# ISG15/GRAIL1/CD3 axis influences survival of patients with esophageal adenocarcinoma

**DOI:** 10.1172/jci.insight.179315

**Published:** 2024-05-23

**Authors:** Dyke P. McEwen, Paramita Ray, Derek J. Nancarrow, Zhuwen Wang, Srimathi Kasturirangan, Saeed Abdullah, Ayushi Balan, Rishi Hoskeri, Dafydd Thomas, Theodore S. Lawrence, David G. Beer, Kiran H. Lagisetty, Dipankar Ray

**Affiliations:** 1 Department of Surgery, Section of Thoracic Surgery;; 2 Department of Radiation Oncology; and; 3 Department of Pathology, University of Michigan, Ann Arbor, Michigan, USA.

**Keywords:** Gastroenterology, Immunology, Cancer

## Abstract

Immunosuppression is a common feature of esophageal adenocarcinoma (EAC) and has been linked to poor overall survival (OS). We hypothesized that upstream factors might negatively influence CD3 levels and T cell activity, thus promoting immunosuppression and worse survival. We used clinical data and patient samples of those who progressed from Barrett’s to dysplasia to EAC, investigated gene (RNA-Seq) and protein (tissue microarray) expression, and performed cell biology studies to delineate a pathway impacting CD3 protein stability that might influence EAC outcome. We showed that the loss of both CD3-ε expression and CD3^+^ T cell number correlated with worse OS in EAC. The gene related to anergy in lymphocytes isoform 1 (GRAIL1), which is the prominent isoform in EACs, degraded (ε, γ, δ) CD3s and inactivated T cells. In contrast, isoform 2 (GRAIL2), which is reduced in EACs, stabilized CD3s. Further, GRAIL1-mediated CD3 degradation was facilitated by interferon-stimulated gene 15 (ISG15), a ubiquitin-like protein. Consequently, the overexpression of a ligase-dead GRAIL1, *ISG15* knockdown, or the overexpression of a conjugation-defective ISG15–leucine-arginine-glycine-glycine mutant could increase CD3 levels. Together, we identified an ISG15/GRAIL1/mutant p53 amplification loop negatively influencing CD3 levels and T cell activity, thus promoting immunosuppression in EAC.

## Abstract 2

CD3 loss in T cells decreases the overall survival of esophageal adenocarcinoma patients and is due to decreased protein stability influenced by GRAIL1.

## Graphical abstract

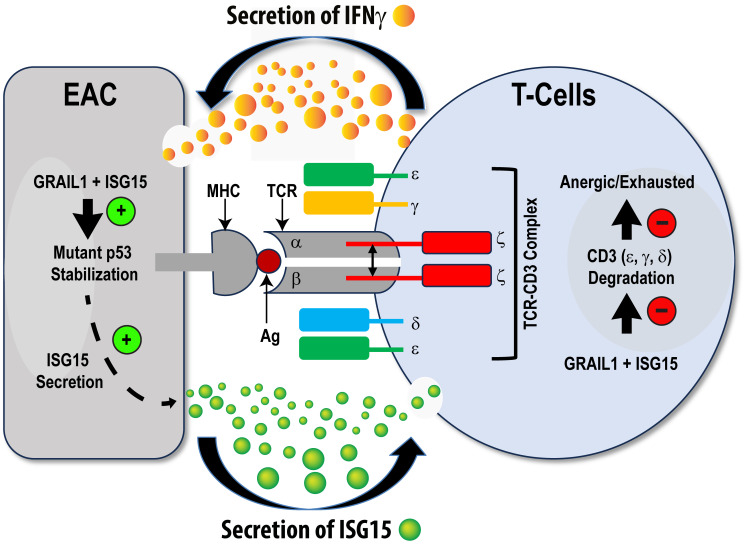

## Introduction

Immune dysregulation is a key determinant of failed response to immunotherapy in esophageal adenocarcinoma (EAC) (1, 2). Despite the advancements in immunotherapy, durable treatment responses have been limited (3, 4). Our previous work demonstrated that Barrett’s (BE) progression to EAC is associated with alterations in chemokines and cytokines with loss of effector T cell function (2). Each step along the progression of BE to EAC is correlated with changes within the immune microenvironment, but the cause of these alterations is largely unknown.

Improving our understanding of how the tumor microenvironment (TME) affects treatment outcome is critically important for improving the outcome of treatment. Immunotherapy using anti– programmed cell death 1 (anti–PD-1), programmed cell death ligand 1 (PD-L1), or cytotoxic T lymphocyte antigen-4 (CTLA-4) agents has demonstrated only modest efficacy for EAC in the adjuvant setting, with little known about which patients may most benefit from these treatment options (3, 5, 6). One known driver of response to immunotherapy is the proliferation of CD8^+^ T cells (7, 8). Emerging evidence indicates that the reduced infiltration or exhaustion of CD8^+^ T cells is one of the causes of poor response (9). Lack of an effector T cell response has been linked to reduced CD3 and T cell receptor (TCR) complex formation, which in turn drives downstream signaling necessary to potentiate immune response following antigen recognition (10). One common mediator of immune dysregulation is mutant p53 (11–13). The majority (>70%) of patients with EAC carry a mutation in the *TP53* tumor suppressor gene that directly increases treatment resistance, as well as influences the surrounding TME to favor tumor growth (14). A plausible explanation of mutant p53 impact on the TME is its influence on altering tumor cell secretome (15). The EAC TME is regulated by a host of mediators, including inhibitory immune checkpoints, chemokines, cytokines, and regulatory immune cells (16). Inhibitory immune checkpoints such as PD-1, PD-L1, and CTLA-4 inhibit immune responses through direct tumor-immune cell interaction as well as via exosome secretion (17, 18). Furthermore, the EAC TME is impacted by cytokines such as TGF-β, IL-6, and IL-10, which aid in the recruitment of inhibitory immune cells such as Tregs and cancer-associated fibroblasts (CAFs) (19). The EAC TME is directly altered via cellular interactions with Tregs and CAFs, both associated with poor survival in EAC (20, 21).

The TCR-CD3 complex serves as the signal initiator for T cell activation (22). Therefore, loss of CD3 has been associated with reduced effector CD8^+^ T cell function (23). The CD3 complex consists of 4 different CD3 polypeptide chains (ε, γ, δ, and ζ) that form ζζ homo- or εγ/εδ hetero-dimers to complex with α and β chains of the TCR (10). Besides transcriptional and posttranscriptional regulation, CD3 is modulated by ubiquitination-mediated degradation (24). RNF128, an E3 ligase, commonly called GRAIL (gene related to anergy in lymphocytes), is a well-known degrader of CD3 (25). *Grail*-deficient mice are resistant to immune tolerance induction and are susceptible to autoimmune disease, suggesting the presence of hyperactive T cells (26). In contrast, Grail upregulation was noted in infiltrated T cells of transplanted lymphomas in mice, whereas Grail loss improved tumor control, suggesting a clinical relevance of the GRAIL-CD3 interaction (26). Our group reported that GRAIL1 levels increase with concomitant loss of isoform 2 during BE progression to EAC (27). Importantly, we demonstrated that this isoform switch was linked to mutant p53 stabilization in EAC cells. However, the isoform-specific role of GRAIL on CD3 polypeptide degradation as well as the correlation of CD3 expression with EAC survival are currently unknown.

Here, we used our cohort of precancer (BE and dysplasia) and EAC cancer tissues from surgically resected patients to identify the impact of immunological changes on patients’ outcome. Furthermore, we sought to understand how the progression associated with GRAIL isoform switch and cooperativity with mutant p53 are impacting CD3 protein stability and tumor cell secretome, respectively, to influence overall survival (OS) in EAC.

## Results

### Lower CD3-ε expression within the tumor and the surrounding stroma is associated with BE progression to EAC.

**Figure 1:**
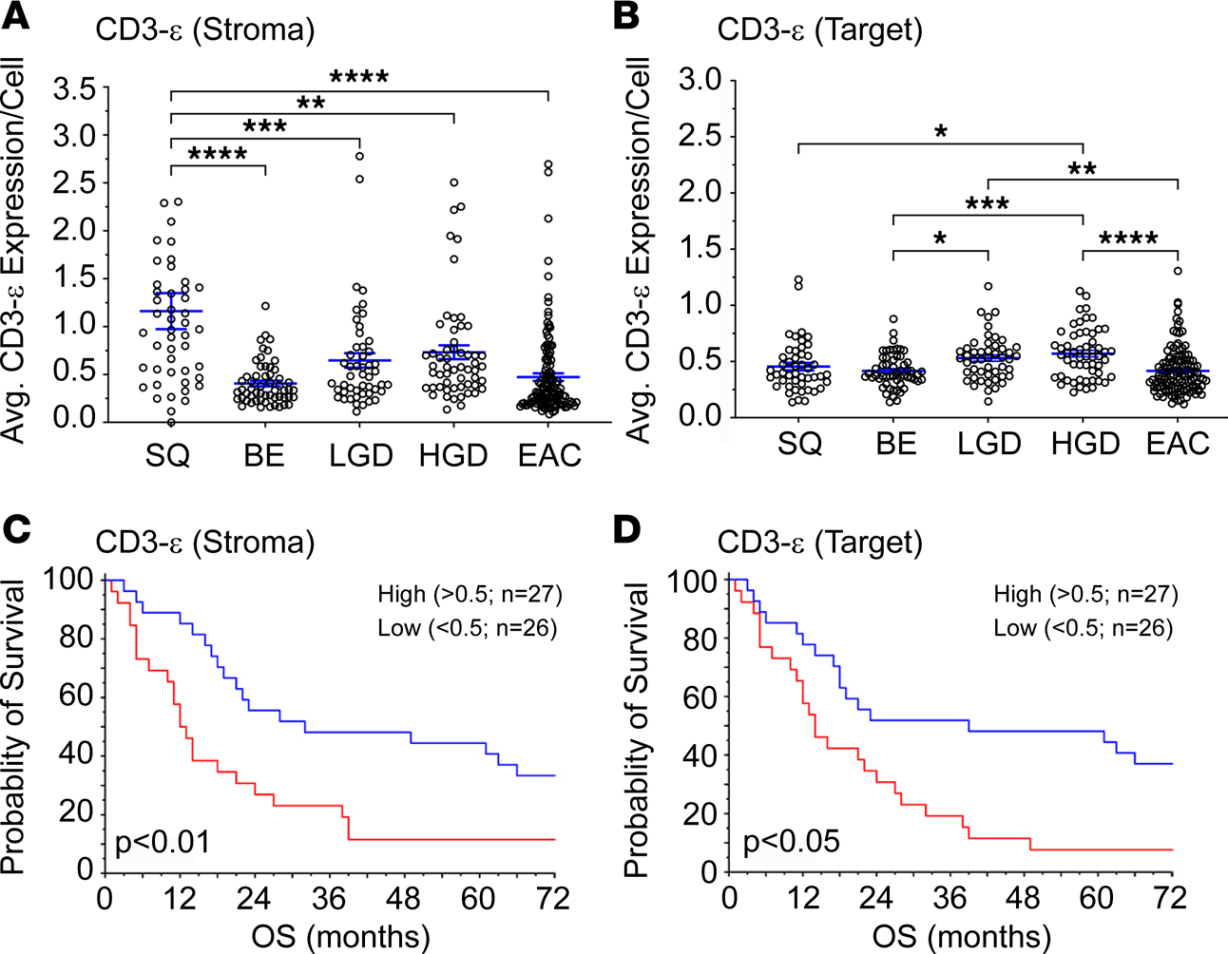
CD3-ε expression during progression from BE to EAC and effects on OS. A TMA consisting of patients with treatment-naive EAC was stained via mIHC for a combination of CD3-ε, CD8, CD163, FoxP3, PanCK, and PD-L1. Tissue sections were classified as squamous tissue (SQ), Barrett’s esophagus (BE), low-grade dysplasia (LGD), high-grade dysplasia (HGD), or esophageal adenocarcinoma (EAC) by an independent pathologist. Stroma is defined as PanCK-negative tissue, while Target indicates PanCK-positive tissue. (**A**) CD3-ε expression in stromal tissue increases from BE to HGD. However, CD3-ε expression decreases from HGD to EAC. (**B**) Similarly, in target tissue, CD3-ε increases during progression from BE to HGD. EAC tissues are immune poor and lack CD3-ε expression. (**C** and **D**) Survival analysis looking at the effects of CD3-ε expression on OS. EACs from panels **A** and **B** were binned into high- and low-expressing populations. For both isoforms, patients with low CD3 expression had worse OS than patients with high CD3-ε expression, regardless of tissue location. For histograms, significance was determined by 1-way ANOVA test with Tukey’s multiple comparison. For statistical significance, *P < 0.05, **P < 0.01, ***P < 0.005, ****P < 0.001. Survival curve differences were determined using Mantel-Cox regression analysis.

Utilizing multiplex immunohistochemistry (mIHC), tissue microarrays (TMAs) representing a cohort of BE, low-grade dysplasia (LGD), high-grade dysplasia (HGD), and EAC were stained for CD3-ε (T cell), CD3-ζ (T cell), CD8 (effector T cell), CD163 (macrophages), FoxP3 (Tregs), pan-Cytokeratin (PanCK: tumor “target” cells), and PD-L1 antibodies. Baseline patient demographics are included in Table 1. Utilizing Akoya’s InForm software, we quantified expression of various markers within cell types as well as the percentage of cells positive for various markers. Cells were categorized as positive if thresholds for a specific marker were above background levels of expression. We further separated expression and cell counts based on tumor (target; panCK positive) versus stromal expression. Our analysis demonstrated that CD3-ε expression within the stroma (Figure 1A) was decreased compared with normal squamous epithelium across all stages of dysplasia and cancer. Within the target tissue, CD3-ε expression increased during BE progression to HGD; however, there was a significant decline in CD3-ε expression during EAC transformation (Figure 1B). To evaluate the potential impact of other CD3 isoforms on progression, we stained for the CD3-ζ isoform, which demonstrated increased levels of CD3-ζ expression within the EAC tissues (Supplemental Figure 1A; supplemental material available online with this article; https://doi.org/10.1172/jci.insight.179315DS1). Our analysis demonstrated a stepwise increase in the expression of effector T cells and Tregs during progression to HGD; however, during HGD transformation to EAC there was a significant reduction (Supplemental Figure 2, A–D). This reduction in effector T cells and Tregs was seen in both the target and stromal compartments. We further analyzed the expression of CD163^+^ macrophages and PD-L1^+^ cells to gain further insight into the expression of antigen-presenting cells (APCs) and inhibitory immune checkpoints within the tumor and TME. Like effector T cell and Treg populations, we noted a rise in macrophages and PD-L1^+^ cell populations from BE to HGD but a decline during HGD progression to EAC (Supplemental Figure 3, A–D). Together, these data indicate that, in general, EAC tissues are immune poor.

### Lower levels of CD3-ε expression are correlated with poor overall EAC patient survival.

Using our clinical database, we assessed the impact of cell surface expression of cellular markers on patient survival. We dichotomized expression into low- and high-expression groups within tumor tissue. Patients with high CD3-ε expression demonstrated a significant improvement in OS compared with patients with low CD3-ε expression. This finding was significant for both tumor and stromal expression of CD3-ε (Figure 1, C and D). When OS was compared between the CD3-ε and CD3-ζ isoforms, only differences in the CD3-ε isoform expression levels were found to significantly affect survival (Supplemental Figure 1B, *P* = 0.05 for CD3-ε compared with *P* = 0.075 for CD3-ζ). When evaluating effector T cell or Treg populations, we found no significant differences in OS (Supplemental Figure 2, E–H). Further, CD163^+^ macrophages and PD-L1^+^ cell populations within the tumor or stroma were not associated with changes in OS (Supplemental Figure 3, E–H).

### BE to EAC progression is associated with lower CD3 engagement with antigen presentation and is associated with poor OS.

**Figure 2:**
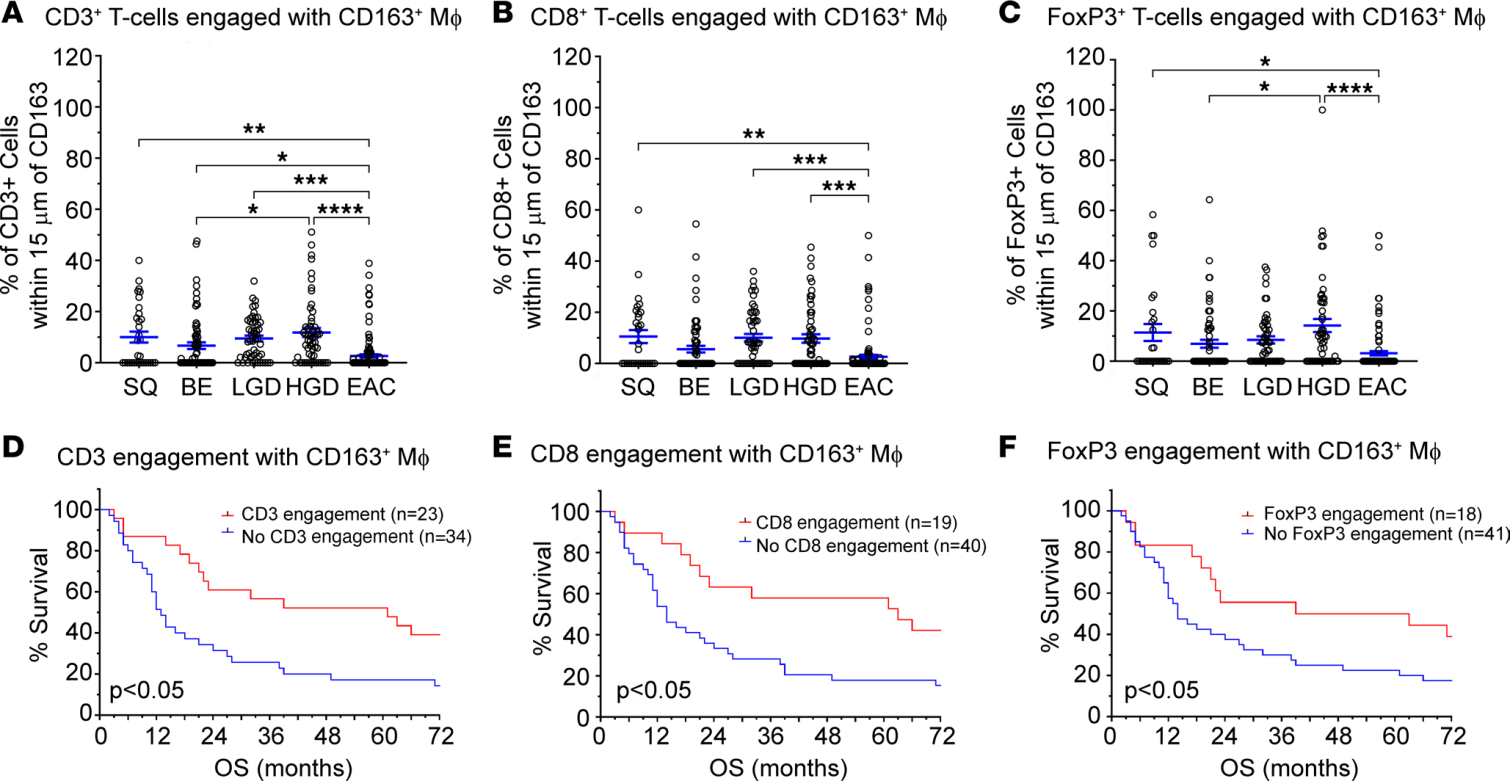
Loss of T cell engagement with CD163^+^ macrophages indicates worse overall patient survival. A TMA from patients with treatment-naive EAC was stained for different T cell markers: CD3 (global T cells), CD8 (cytotoxic T cells), and FoxP3 (Tregs), while CD163 was used as a macrophage marker. InForm software analysis was used to determine the percentage of CD3^+^, CD8^+^, or FoxP3^+^ cells within 15 μm of CD163^+^ macrophages, as an indication of cell-cell engagement. (**A**–**C**) T cell engagement with CD163^+^ macrophages increases during progression from BE to HGD but is nearly absent in EAC tissue for CD3^+^ (global; **A**), CD8^+^ (cytotoxic; **B**), and FoxP3^+^ (regulatory; **C**) T cells. (**D**–**F**) Survival analysis indicates that a lack of engagement of T cells with CD163^+^ macrophages indicates worse OS compared with high T cell engagement with macrophages, regardless of the T cell population. For histograms, significance was determined by 1-way ANOVA test with Tukey’s multiple comparison. For statistical significance, *P < 0.05, **P < 0.01, ***P < 0.005, ****P < 0.001. Survival curve differences were determined using Mantel-Cox regression analysis.

To understand the clinical impact of the immune-poor TME of EAC tissue, we investigated engagement of T cells with APCs and their effects on OS of patients with EAC. Using Akoya’s InForm software, we determined the percentage of CD3^+^ T cells that were within 15 μm of cells staining for cytokeratin, CD163, or PD-L1 (Supplemental Figure 4), an accepted way of analyzing cellular engagement data (28). We used CD163 as a marker for a subset of macrophages within the TME. Interestingly, engagement analysis demonstrated an increase of CD3^+^, CD8^+^, and FoxP3^+^ cellular engagement with APCs during BE progression to HGD. However, our data demonstrated that there was lower CD3^+^ T cell engagement with CD163^+^ APCs in EAC (Figure 2A). This was also seen when evaluating CD8^+^ T cell and FoxP3^+^ Treg engagement with APCs in EAC (Figure 2, B and C). The clinical importance of this finding was demonstrated using survival analysis. Patients with tumors showing loss of T cell engagement with CD163^+^ macrophages had worse OS compared with patients with higher levels of T cell engagement (Figure 2, D–F). This finding was consistent even when analyzed for CD8^+^ and FoxP3^+^ cellular engagement with PD-L1^+^ APCs (Supplemental Figure 5, A–F). Engagement of any T cell with PanCK^+^ epithelium had no effect on OS (data not shown).

### GRAIL isoforms differentially degrade CD3 isoforms.

**Figure 3:**
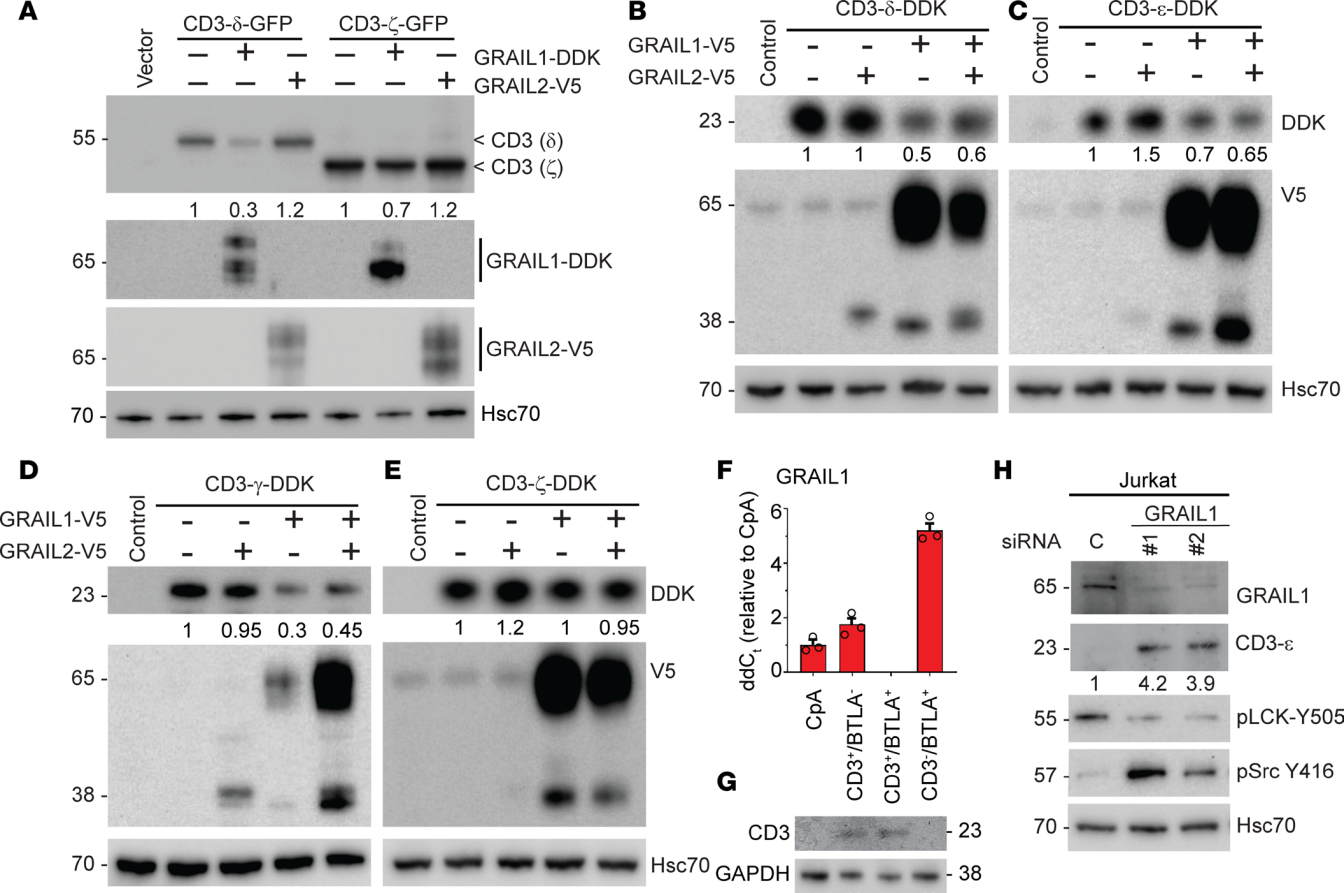
GRAIL1 downregulates, but GRAIL2 upregulates, CD3 isoforms. (**A**) In HeLa cells we overexpressed GFP-tagged CD3-δ and ζ in the presence and absence of either DDK-tagged GRAIL1 or V5-tagged GRAIL2. Cell lysates were prepared 24 hours after transfection and subjected to immunoblotting as indicated. (**B**–**E**) DDK-tagged CD3 isoforms (ε, γ, δ, ζ) were co-overexpressed in the presence of V5-tagged GRAIL1, GRAIL2, or both isoforms. Cell lysates were then subjected to immunoblotting. (**F** and **G**) Patient PBMCs were flow-sorted to isolate T cells, which were then exposed to αCD3/CD28 antibodies for 10 days. Cells were FACS-sorted based on CD3 and BTLA expression and subjected to either quantitative reverse transcriptase PCR or immunoblotting. ddC, ∆∆Ct. (**H**) Human T cell leukemia (Jurkat) cells were subjected to transfection using either control or 2 independent *GRAIL1*-specific siRNAs. Forty-eight hours after transfection, cell lysates were prepared and subjected to immunoblotting using indicated antibodies.

Previous studies have identified RNF128/GRAIL as a ubiquitin ligase for CD3 ubiquitination and degradation (25). As our prior work identified the differential effects of GRAIL isoforms on mutant p53 (27), we asked whether GRAIL isoforms (GRAIL1 and GRAIL2) have differential effects on different CD3 polypeptides (ε, δ, γ, and ζ). The CD3 isoforms showed minimal (10%–15%) amino acid sequence homology (Supplemental Figure 6), with CD3-δ and -γ proteins being the most similar (~36%). We initiated our studies by overexpressing GFP-tagged CD3-δ and -ζ constructs either alone or co-overexpressed with GRAIL1-DDK or GRAIL2-V5 constructs using HeLa cells as an overexpression system. As shown in Figure 3A and Supplemental Figure 7, GRAIL1-DDK was effective in reducing CD3-δ levels with marginal impact on CD3-ζ. By comparison, GRAIL2-V5 overexpression increased both CD3-δ and CD-ζ levels. These results indicate a stark contrast with the effects of GRAIL isoforms we noted on p53, where GRAIL1 stabilizes but GRAIL2 degrades mutant p53 (27). As the above CD3 constructs were GFP tagged (a bigger tag that may influence protein stability), we repeated similar experiments using DDK-tagged CD3 constructs (ε, γ, δ, ζ) either in the presence or in the absence of V5-tagged GRAIL1 or GRAIL2. As shown in Figure 3, B–E, overexpression of GRAIL1-V5 downregulated CD3-ε, -δ, and -γ; however, the effect on CD3-ζ was minimal. In comparison, overexpression of GRAIL2-V5 increased CD3-ε and -ζ levels. Immunofluorescence and immunoblot analysis produced similar results (Supplemental Figure 8).

In these studies, GRAIL1 showed dominant effects on CD3 levels when co-overexpressed with GRAIL2. To test the physiological relevance of our observation, we isolated T cells from patient peripheral blood mononuclear cells (PBMCs) and then induced them to exhaustion by treating with αCD3/αCD28 Dynabeads for 10 days. BTLA was used as a marker of exhausted T cell populations. Cells were sorted into 4 clusters based on CD3 and BTLA expression levels (Supplemental Figure 9A). We observed increased *GRAIL1* gene expression in the anergic (CD3^–^BTLA^+^) population (Figure 3F and Supplemental Figure 9B), which expressed low levels of *GRAIL2* (Supplemental Figure 9C). As expected, CD3 levels were undetectable in the CD3^–^BTLA^+^ T cell population expressing higher GRAIL1, suggesting an inverse correlation (Figure 3G). We then performed isoform-specific siRNA-mediated *GRAIL1* and *GRAIL2* silencing using 2 different siRNAs (27) in the Jurkat cell line (a human leukemia T cell lymphoblast). As expected, loss of *GRAIL1* increased CD3-ε levels, and loss of *GRAIL2* reduced it (Figure 3H and Supplemental Figure 9D). We also checked the CD3 downstream signaling (Figure 3H) and noted reduced pLCK Y505 phosphorylation with a corresponding increase in pSrc Y416 phosphorylation, suggesting T cell activation in this system. Together, we show an isoform-specific effect of GRAIL on different CD3 levels (ε, δ, and γ) influencing downstream signaling.

### GRAIL1 promotes polyubiquitination-mediated CD3 degradation.

**Figure 4:**
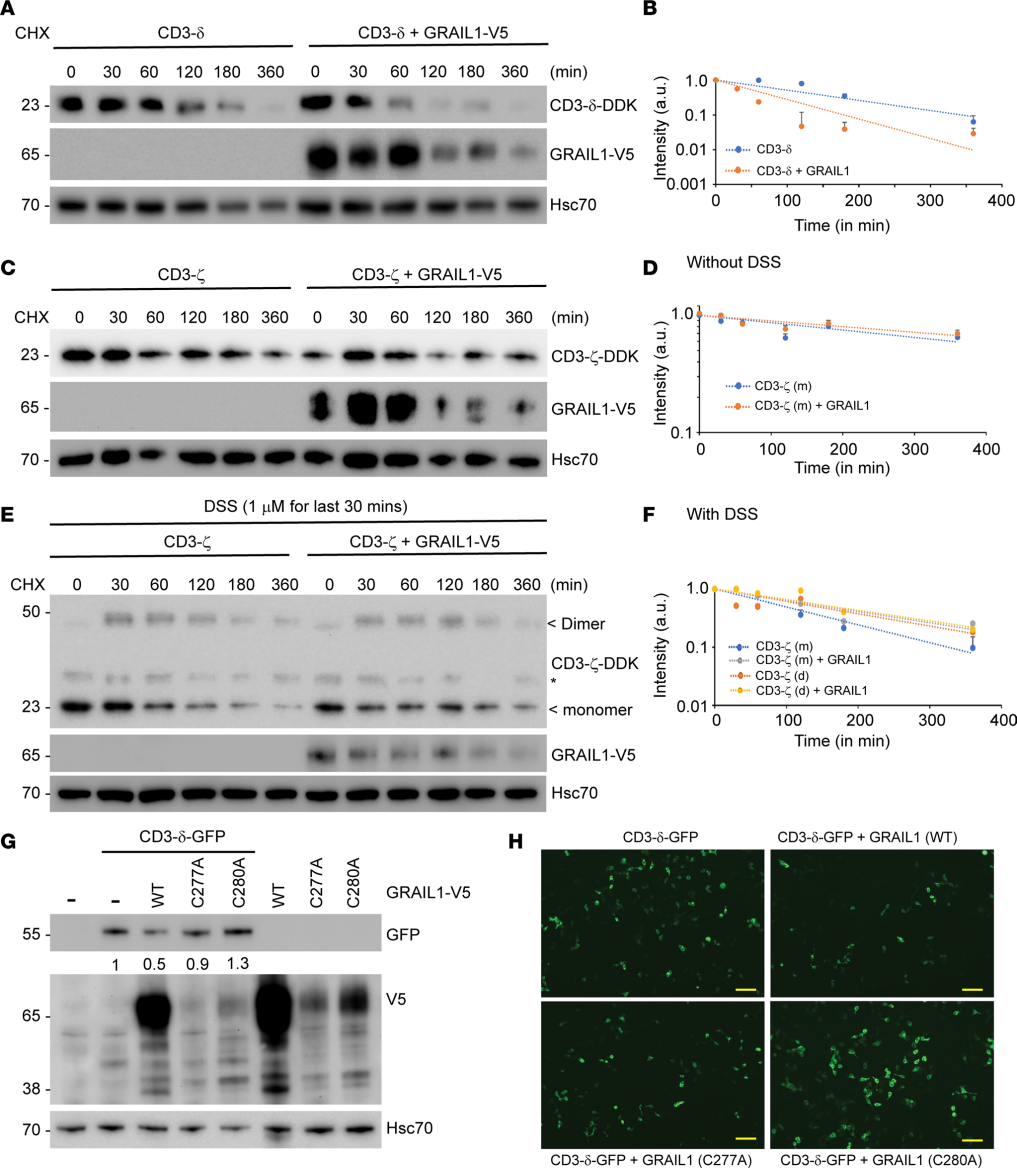
GRAIL isoform 1 promotes CD3-ε degradation in a catalytic activity–dependent manner. (**A** and **B**) DDK-tagged CD3-ε was overexpressed in HEK193 cells in the presence or absence of V5-tagged GRAIL1. Twenty-four hours after transfection, cells were treated with cycloheximide (50 μg/mL); lysates were prepared at indicated time points and subjected to immunoblotting. To determine protein half-life, band intensities were measured using ImageJ (NIH) and normalized by considering 0-hour time point band intensity as “1” arbitrary unit (a.u.). (**C** and **D**) Similar experiments were performed using DDK-tagged CD3-ζ and half-life was calculated in the presence and absence of GRAIL1 overexpression. (**E** and **F**) This experiment was performed similarly to panel **C**, except to restore dimeric CD3-ζ, cells were treated with DSS (1 μg/mL) for the final 30 minutes prior to harvest. Half-lives were calculated for the CD3-ζ monomer and dimer as described above. (**G**) GFP-tagged CD3-δ was overexpressed either alone or in the presence of wild type (WT) GRAIL1 or its catalytically inactive C277A and C280A mutants. Twenty-four hours after transfection, cell lysates were prepared and subjected to immunoblotting as indicated. (**H**) Live cell image of CD3-δ-GFP–expressing cells either alone or in the presence of WT, C277A, and C280A GRAIL1 mutants. Scale bar: 50 mm.

Previous studies have indicated CD3-ζ can be polyubiquitinated by Casitas B-lineage lymphoma (CBL-b), another RING finger family ubiquitin ligase (29). Such posttranslational modification is nondegrading, but it impacts receptor endocytosis to alter downstream signaling. To test whether GRAIL1-mediated downregulation of CD3 polypeptides is due to protein degradation, we performed protein half-life studies using cycloheximide, an inhibitor of eukaryotic protein translation. As shown in Figure 4A and quantified in Figure 4B, overexpression of GRAIL1 reduced CD3-δ half-life from approximately 140 minutes to 60 minutes, indicative of protein degradation. We noted minimal changes in CD3-ζ half-life upon GRAIL1 overexpression (Figure 4, C and D). As CD3-ζ is known to form homodimers, we performed CD3-ζ protein half-life studies in the presence of disodium sulfate (DSS), a cross-linking reagent. As shown in Figure 4E and quantified in Figure 4F, GRAIL1 overexpression had no major impact on either monomeric or dimeric CD3-ζ, suggesting CD3-ζ is not a GRAIL1 substrate. To show the importance of GRAIL1 ubiquitin ligase activity on promoting specific CD3 isoforms, we created catalytic site mutants by mutating Cys277 and Cys280 to alanine (C277A, C280A). As shown in Figure 4, G and H, between the 2 mutants, the C280A GRAIL1 mutant lost the ability to promote CD3-δ degradation and instead showed a dominant-negative effect to increase levels, demonstrating the importance of GRAIL1 ligase activity on CD3-δ degradation. The C277A mutant showed a similar loss of activity; however, the impact was lower, suggesting C280 is one of the key residues involved in GRAIL1 catalytic activity.

**Figure 5:**
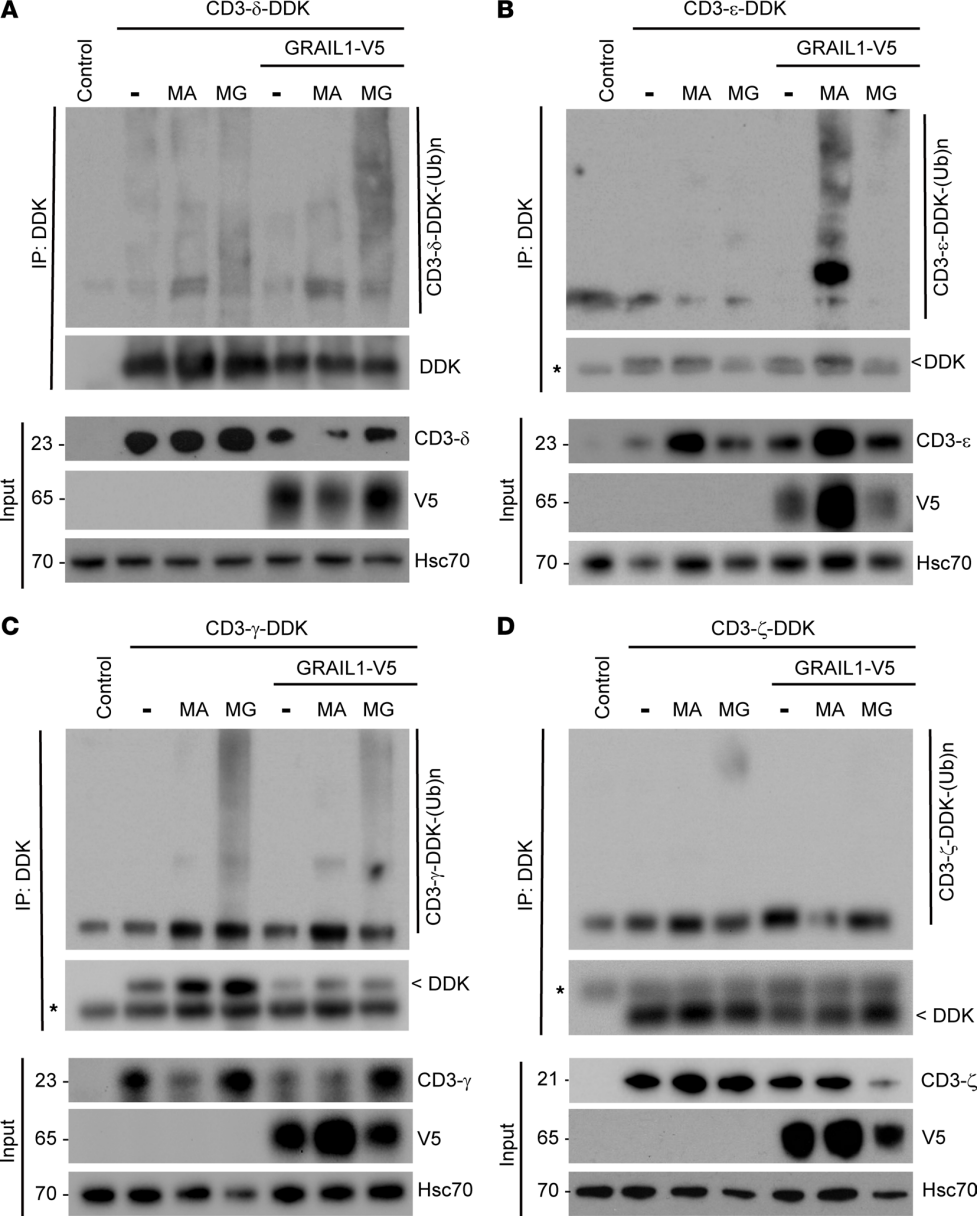
GRAIL1 promotes CD3-ε, -γ, and -δ polyubiquitination but has no effects on CD-ζ. (**A**–**D**) DDK-tagged CD3-δ, CD3-ε, CD3-γ, and CD3-ζ, respectively, was expressed alone or in combination with V5-tagged GRAIL1. Twenty hours after transfection, cells were treated with either a lysosomal (25 mM of MA) or proteasomal (2 μM of MG132 [MG]) inhibitor as indicated. Four hours following inhibitor treatment, cell lysates were prepared and subjected to immunoprecipitation using Affi-FLAG beads and immunoblotted using indicated antibodies.

Once we determined that GRAIL1 mediates CD3-ε, -δ, and -γ degradation, we used proteasomal (MG132) and lysosomal (methylamine hydrochloride, MA) inhibitors to understand the importance of polyubiquitination in the process. As shown in Figure 5, A–D, GRAIL1-mediated CD3-ε loss was found to be lysosome mediated (increased polyubiquitinated species in the presence of MA), but the CD3-δ and -γ degradation was at the proteasome (increased ubiquitinated species when treated with MG132). As expected, MG132 and MA had no effects on CD3-ζ or its ubiquitinated species. Additionally, when we performed immunofluorescence studies, CD3-ε expression showed punctate localization, and such puncta were colocalized with LAMP1 (a lysosomal marker) when co-overexpressed with GRAIL1 (Supplemental Figure 10).

### ISGylation is necessary for GRAIL1-mediated CD3 degradation.

**Figure 6:**
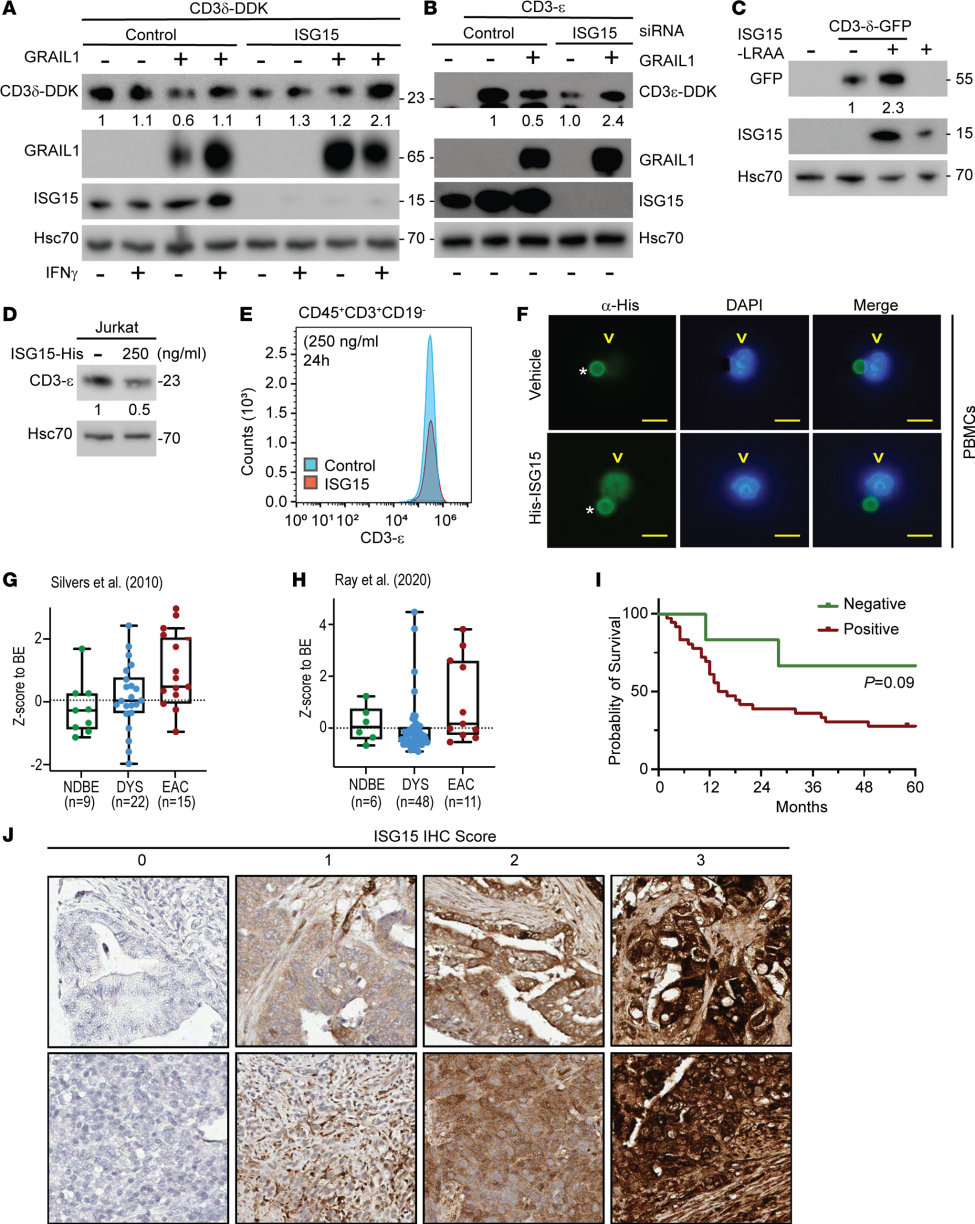
ISG15 is required in GRAIL1-mediated CD3 degradation, and higher ISG15 expression is correlated with poor OS in EAC. (**A** and **B**) CD3-ε and CD3-δ were overexpressed in the presence or absence of GRAIL1 in HeLa cells following transfection of either control or *ISG15* siRNA. For panel **A**, cells were also treated with or without IFN-γ (10 ng/mL) for 6 hours, as indicated, prior to protein isolation. Cell lysates were subjected to immunoblotting using indicated antibodies. (**C**) GFP-tagged CD3-δ was transfected alone or along with a conjugation-deficient ISG15 LRAA mutant, as indicated. Twenty-four hours after transfection, cell lysates were prepared and subjected to immunoblotting. (**D**) Jurkat cells were treated with His-tagged ISG15 (250 ng/mL) for 24 hours, and cell lysates were subjected to immunoblotting. (**E**) PBMCs were treated with recombinant ISG15, and 24 hours after treatment cells were analyzed for surface CD3-ε expression using FACS. (**F**) PBMCs were treated with recombinant ISG15 for 15 minutes. Cells were then washed and subjected to immunofluorescence using anti-His antibody. Anti-CD3/CD28 beads used for T cell activation, showing nonspecific staining (*) in both vehicle control– and ISG15-treated samples. This was used for immunofluorescence intensity normalization, and His-ISG15 specific staining is shown by arrowhead (v). Scale bar, 10 μm. (**G** and **H**) *ISG15* gene expression levels in EACs were compared with nondysplastic Barrett’s (NDBE) and dysplasia (DYS) based on previously published RNA-Seq data sets (57, 27). Box plots show the interquartile range, median (line), and minimum and maximum (whiskers). (**I**) Kaplan-Meier curves showing OS of patients with EAC expressing either negative (*n* = 6) or positive (*n* = 40) ISG15 expression (*P* = 0.09) based on log-rank Mantel-Cox test). (**J**) Representative EAC TMA cores showing numbers with no (scored 0) and different levels (scale of 1–3 as we defined) of ISG15 expression. Pictures were captured at 20× original magnification.

To identify the regulators of GRAIL1 functionality in promoting CD3 degradation, we performed immunoprecipitation followed by mass spectrometry analysis. Interferon-stimulating gene 15 (ISG15) was found to be a key interactor of GRAIL1 (Supplemental Table 1). ISG15 is a ubiquitin-like (UBL) molecule that can be conjugated to a target protein (a process called ISGylation) to modulate protein stability and functionality. We previously reported that IFN-γ signaling is increased during BE to EAC progression (27). As ISG15 is an IFN-γ–inducible factor, we hypothesized that GRAIL1 interaction with ISG15 may be relevant to its stability/functionality. As expected, loss of *ISG15* compromised GRAIL1-mediated CD3-δ degradation, showing the critical importance of ISGylation in this process (Figure 6A). A partial rescue of GRAIL1-mediated CD3 downregulation was noted when cells were treated with excess IFN-γ (10 ng/mL). Similarly, loss of GRAIL1 functionality promoted CD3-ε degradation, supporting our observation (Figure 6B). To verify the importance of ISGylation in promoting CD3 degradation, we overexpressed a functionally defective leucine-arginine-alanine-alanine (LRAA) mutant of ISG15, which promotes dominant-negative effects on ISGylation (30). As shown in Figure 6C, overexpression of ISG15-LRAA mutant increased CD3-δ levels, suggesting ISGylation is important in promoting CD3 degradation. In contrast, treatment of either Jurkat (T cell lymphocytic leukemia) cells or PBMCs with exogenous recombinant and active (leucine-arginine-glycine-glycine exposed), histidine-tagged (His-tagged) ISG15 protein downregulated CD3-ε within 24 hours of treatment (Figure 6, D and E), as measured using immunoblotting and flow cytometry, respectively. Using immunofluorescence, we detected the entry of His-tagged ISG15 in PBMCs within 15 minutes of addition of the recombinant protein (Figure 6F).

### High levels of ISG15 are correlated with poor OS in EAC.

To understand the clinical relevance of ISG15 in EAC, we used our published and publicly available RNA-Seq data (NCBI Gene Expression Omnibus series GSE37203 and GSE193946). As shown in Figure 6G, we noted increased ISG15 expression in EAC samples compared with nondysplastic and dysplastic BE samples. Results from 2 previously published EAC-related bulk RNA-Seq cohorts demonstrate that both ISG15 and markers for IFN response are increased in EAC, relative to normal esophagus or NDBE from patients with EAC, suggesting that higher ISG15 levels in cancer tissue may be a result of fortified IFN signaling from local T cell population(s). Additionally, we performed immunohistochemical (IHC) staining of an EAC TMA (*n* = 46) showing patients expressing ISG15 (*n* = 40) showed a trend of correlation with poor OS (*P* = 0.09) compared with nonexpressors (*n* = 6; ~12% of all EACs) (Figure 6, H and I, and Table 2), suggesting a critical role of ISG15 expression in EAC.

### Mutant p53 influences ISG15 secretion.

**Figure 7:**
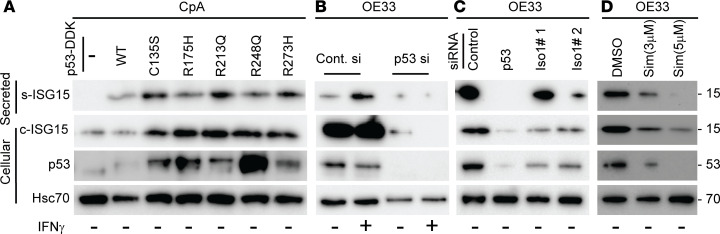
Mutant p53 influences ISG15 secretion. (**A**) Wild-type (WT) p53–containing nondysplastic CpA cells were either left untransfected or transfected with DDK-tagged WT or different p53 mutants (C135S, R175H, R213Q, R248Q, R273H) as indicated. Forty-eight hours after transfection, cell culture supernatants were collected, concentrated (see Methods), and subjected to immunoblotting to quantify secreted ISG15 (s-ISG15) levels. Cellular ISG15 (c-ISG15) levels were determined using immunoblotting. Panels also show the levels of p53 expression, and Hsc70 was used as loading control. (**B** and **C**) OE33 cells were transfected using control, *TP53*, or 2 different *GRAIL1* siRNAs, and 48 hours after transfection, supernatant and cell lysates were prepared and immunoblotted as above. In the indicated lanes, cells were also treated with IFN-γ (10 ng/mL) for the last 6 hours prior to harvest. (**D**) OE33 cells were treated with DMSO or different concentrations (3 and 5 μM) of simvastatin. Forty-eight hours after treatment, samples (secreted and cellular) were prepared as above and immunoblotted.

Wild-type (WT) p53 is a known transcriptional activator of ISG15, which in turn can posttranslationally modify p53 to impact p53 transcriptional ability, suggesting the presence of a positive feedback loop (31). Importantly, mutant p53 is known to influence cellular secretome (15). We analyzed a nondysplastic, WT p53–containing BE cell line (CpA). Although we detected significant amounts of intracellular ISG15, CpA cells did not secrete ISG15 in culture supernatant (Figure 7A). Interestingly, following overexpression of different p53 mutants (e.g., C135S, R175H, R213Q, R248H, and R273H) in CpA cells, we found increased secreted ISG15 (s-ISG15) (Figure 7A). In contrast, the knockdown of mutant *TP53* (Figure 7B) or *GRAIL1* (Figure 7C) reduced s-ISG15 in mutant p53–driven OE33 cells. Thus, in tumor cells, while ISG15 influences GRAIL1 functionality to stabilize mutant p53 (27), in turn stabilized mutant p53 can facilitate the secretion of s-ISG15 by the tumor cells to create an immunosuppressive TME by negatively influencing T cells. As we and others have previously reported that simvastatin, a cholesterol-lowering drug, can reduce mutant p53 levels in EAC cells (27), we then tested simvastatin’s effects on ISG15 secretion. In OE33 cells, simvastatin reduced both cellular and s-ISG15 levels in a dose-dependent manner, and the reduced ISG15 secretion was correlated with the corresponding loss of mutant p53 (Figure 7D).

## Discussion

**Figure 8:**
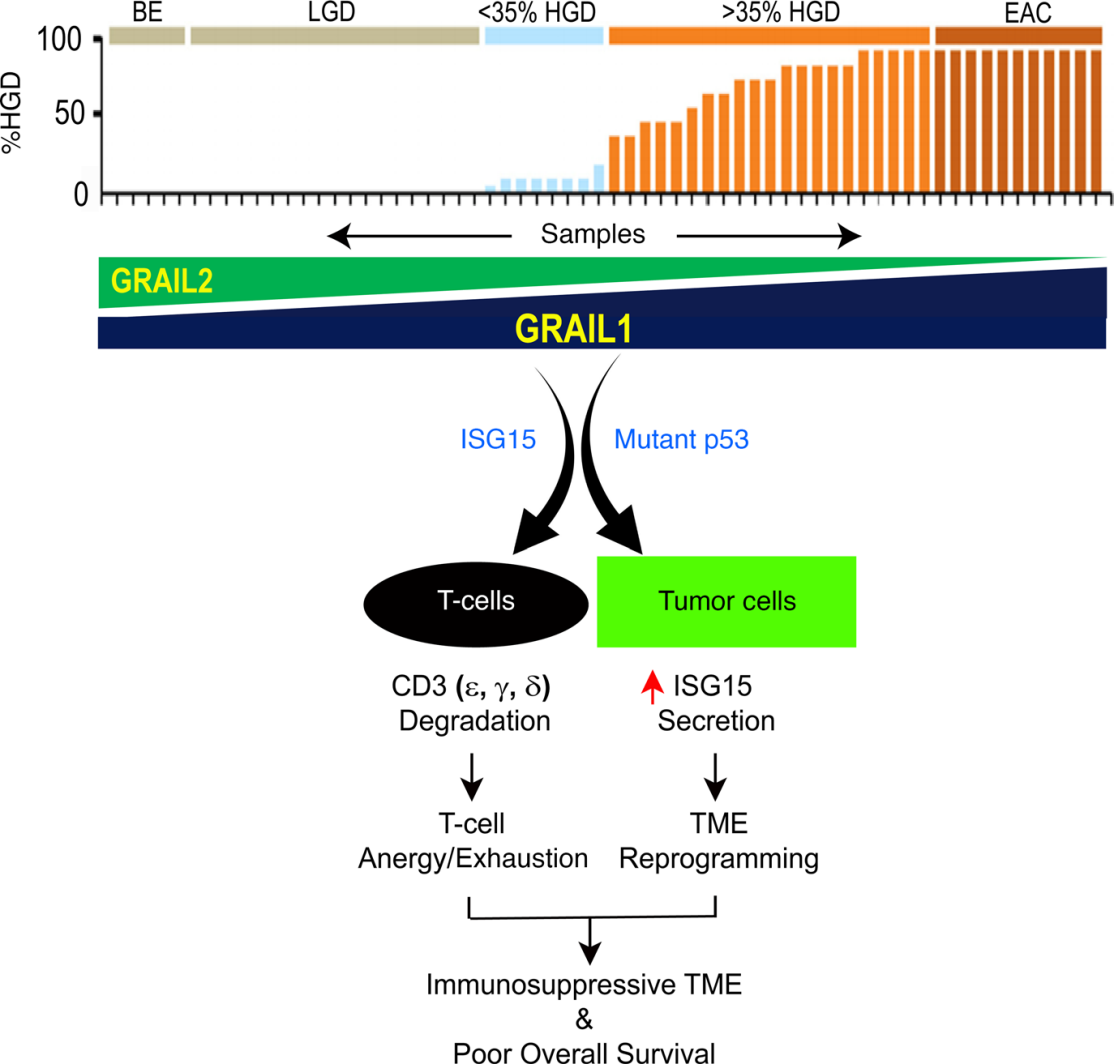
Schematic model showing involvements of ISG15/GRAIL1/mutant p53 axis influencing the TME in patients with EAC. Using BE → dysplasia → EAC patient samples, we previously reported the enrichment of GRAIL1 and loss of GRAIL2 isoform expression (27). Here we show that in T cells, GRAIL1 can cooperate with ISG15 to efficiently ubiquitinate and degrade CD3-ε, -γ, and -δ isoforms. In tumor cells, we show GRAIL1 cooperates with mutant p53, and we find that such cooperativity influences the secretion of ISG15 (s-ISG15), a known paracrine factor reported to reprogram the TME. Clinically, we show that either the reduced CD3 expression or higher ISG15 levels are linked to poor OS in EAC.

In this paper, we have found that EAC tissues present with a loss of CD3 expression as well as a reduction in the number of CD3^+^ T cells and are associated with poor OS. Patients with poor OS are further characterized by TME showing loss of engagement between T cells and APCs. GRAIL1, which we previously reported to be enriched in EACs, targets different CD3 isoforms (ε, γ, and δ) expressed in T cells via ubiquitination-mediated lysosomal/proteasomal degradation. We show that an interferon-stimulated UBL protein, called ISG15, is required to optimize GRAIL1 ubiquitin ligase’s ability to degrade CD3. Treatment of T cells with exogenous recombinant ISG15 protein can downregulate CD3-ε levels. Consequently, EACs expressing higher levels of ISG15 correlate with worse OS than nonexpressors. Additionally, we show that mutant p53, which is the key driver of the majority (>70%) of EACs, partners with GRAIL1 to influence the tumor cell secretome, enhancing the release of ISG15 by tumor cells (Figure 8), a paracrine factor reported to reprogram the TME. Together, we show that the ISG15/GRAIL1/mutant p53 amplification loop is a potentially novel signaling axis that negatively influences T cell activity, thus creating an immunosuppressive TME responsible for poor OS in EAC. Approximately 75% of all p53 mutations in EACs are missense type (32–38), suggesting the importance of the ISG15/GRAIL1/mutant p53 signaling axis in the majority of patients with EAC.

Our study is consistent with previous reports of correlations between the levels and numbers of CD3^+^ T cells and OS across several cancer types. For example, increased presence of CD3^+^ and CD8^+^ infiltrating T cells was shown to prolong survival of patients with glioblastoma (39). Similarly, increased CD3 levels in tumor infiltrating lymphocytes (TILs) in patients with HPV^+^ head and neck cancer demonstrated improved disease-specific survival (40). In hepatocellular carcinoma, high levels of both CD3^+^ and CD8^+^ T cells were associated with a low rate of recurrence and prolonged relapse-free survival (RFS) (41). In esophageal squamous cell carcinoma (ESCC), higher numbers of CD3^+^ TILs correlated with longer median RFS and OS (42). In a separate study, CD3^hi^ + PD-L1^hi^ subgroups of ESCC also showed beneficial outcomes, including overall survival, disease-free survival, and disease-specific survival (43). Our previous work evaluating the immune determinants of BE progression to EAC highlighted the loss of CD8^+^ T cells within the peritumoral environment (2). In that study, we were able to demonstrate the clinical importance of T cell loss as well as how T cell loss led to decreased APC engagement. Here, utilizing a TMA established from patients with treatment-naive EAC and isoform-specific (ε and ζ) CD3 antibodies, we have found a statistically significant correlation between lower CD3-ε levels and poor OS compared with CD3-ζ. Our clinical data are limited by the lack of follow-up regarding receipt of adjuvant chemotherapy for patients with recurrent disease. Assuming the rates of receipt of adjuvant therapy are consistent among patients, we do not believe this limitation affects the results of our study.

It is important to emphasize that we found GRAIL1 as an effective degrader of CD3-ε, but not CD3-ζ, which may explain differences noted between levels of different CD3 isoforms and OS. This was further supported from data shown in Supplemental Figure 9D, where siRNA-mediated knockdown of *GRAIL1* caused CD3-ε accumulation, yet only marginal changes in CD3-ζ were noted. A study reported CBL-b as another E3 ligase that promotes CD3-ζ K33-linked polyubiquitination, which is important in regulating T cell activation (29). Genetic loss of either *Grail* or *Cbl-b* in mice showed similar hyperactive T cell phenotypes, and these knockout mice were found to be highly susceptible to autoimmunity, suggesting overlapping functionality (25, 44). In humans, genetic polymorphism of CBL-b has been linked to autoimmunity (45), and targeting CBL-b can improve antitumor immunity (46). Similarly, patients with lymphoma showed higher GRAIL expression in CD8^+^ T cells compared with T cells isolated from healthy individuals (26). Such data clearly suggest the clinical importance of GRAIL and CBL-b in T cell inactivation, which target distinct CD3 isoforms (GRAIL1 impacts CD3-ε, -γ, and -δ protein stability, whereas CBL-b influences CD3-ζ downstream signaling by impacting receptor endocytosis).

ISG15 is a UBL protein stimulated upon IFN treatment and largely studied for controlling viral infection (47). Furthermore, ISG15 has also been implicated in cancers such as breast, pancreas, and ovary, where it controls various pathways, including autophagy, exosome secretion, DNA replication stress response, and immune regulation (48–51). Our group has previously reported alteration in IFN-γ signaling during BE to EAC progression, and this altered signaling partly causes the GRAIL isoform switch (27). Additionally, we noted that GRAIL1 and -2 have differential impacts on mutant p53 protein stability in epithelial cancer cells, where isoform 1 stabilizes mutant p53 protein while isoform 2 is a potent mutant p53 degrader. As GRAIL1 was enriched and isoform 2 was lost in EACs, we proposed the importance of GRAIL isoform switch as a critical event facilitating nonmalignant BE progression into EAC. Here we additionally show that in T cells, GRAIL1 can degrade CD3 to dampen downstream signaling, resulting in T cell inactivation/anergy. While understanding the differential effects of the 2 GRAIL isoforms on p53 and CD3, we unexpectedly identified ISG15 as one of the previously unknown interactors. ISG15, like ubiquitin, can either be present in its free form, which can be secreted to the TME to influence tumor adjacent cells (52), or be covalently conjugated to the lysine (K) residues of target proteins using specific E1 (UBE1L), E2 (UBCH8), and E3 (TRIM family members, etc.) enzymes by a process termed ISGylation (53). Like ubiquitination, ISGylation is known to alter protein stability and functionality, and it is also known to compete with ubiquitination to maintain cellular homeostasis. As shown in Figure 6, while siRNA-mediated loss of *ISG15* increased GRAIL1 steady-state levels, it compromised GRAIL1 functionality and failed to promote CD3 degradation. Similarly, overexpression of a dominant-negative ISG15-LRAA mutant increased CD3 levels, suggesting the importance of ISGylation in the maintenance of CD3 expression levels. To our surprise, in *ISG15*-knockdown cells, treatment with IFN-γ (10 ng/mL) resulted in the loss of GRAIL1 with consequent accumulation of CD3 protein, whose mechanism is unclear. Due to our initial noted correlation between CD3 levels and EAC OS, we performed ISG15 IHC using a treatment-naive EAC TMA. As shown in Figure 6I, increased expression of ISG15 was correlated with poor OS. In our patient cohort, we identified about 12% (6/46) of patients showing no detectable ISG15, and these patients had better OS, suggesting ISG15 expression may be a good prognostic marker. It is important to emphasize that about 10%–15% of EACs respond to the current standard-of-care chemoradiation treatment, and future studies may be geared toward establishing a correlation between ISG15 levels and therapy response or resistance.

Our findings identify ISGylation as a critical posttranslational protein modification regulating T cell functionality by affecting CD3 protein abundance. Our data indicate that either physical (siRNA) or functional (overexpression of ISG15-LRAA mutant) loss of ISG15 can stabilize CD3 proteins, which may help in overcoming T cell anergy. Thus, targeting ISG15 or ISGylation may be an unexplored approach to restore immune response in patients with cancer. However, there are no agents currently available capable of targeting ISG15/ISGylation. Our work emphasizes the need to develop a first-in-class small molecule capable of inhibiting ISGylation that may improve immunotherapy efficacy.

Despite the above discussed clinical importance, we recognize certain limitations of our work. First, our survival data are based on a retrospective cohort of patients, who may have received adjuvant therapy impacting OS. In addition, BE, LGD, and HGD specimens were taken from patients who progressed to EAC, and each tissue section was verified by a pathologist. Our analysis did not include specific CD8^+^ and CD4^+^ T cell subsets, which may further characterize the specific T cell subpopulation where GRAIL exerts its effects. Our current data indicate Cys280 as a critical catalytic cysteine residue essential for carrying out the CD3-degrading ability of the GRAIL1. As GRAIL is a C3H2C3 zinc finger motif containing E3 ligase, future analysis is required for better understanding. In addition, it may also be worthwhile to identify the lysine (K) residue(s) in GRAIL1 that undergo ISGylation to impact functionality. We located 2 probable ISGylation sites (197-KK-198 and 252-KK-253), but future studies may be necessary to better understand the mechanisms of GRAIL1-mediated CD3 degradation. Finally, although our study identifies a mutant p53/GRAIL axis positively influencing the secretion of s-ISG15, which like a cytokine can reprogram the immune microenvironment (52), the exact mechanism of how mutant p53 influences exosome-mediated ISG15 release (54) by tumor cells and amplifies oncogenic signal remains unclear. Our data show that exogenously added recombinant ISG15 can promote CD3 degradation, possibly upon internalization (Figure 6, D–F). However, like tumor cells, T cells also produce significant amounts of ISG15; thus the extent of cell-autonomous versus cell-nonautonomous impact of ISG15 on T cell functionality requires further understanding.

In summary, low levels of CD3 and higher ISG15 expressions are found to be critical indicators predicting survival of patients with EAC. Our data suggest that in this mutant p53–centric cancer progression process, the GRAIL/ISG15 axis acts as a critical mediator of how tumor cells diminish T cell attack. Future work should focus on developing GRAIL1-targeting strategies, via physical or functional loss (via ISG15 targeting) of GRAIL1 activity, that may restore CD3 levels in T cells and improve immunotherapy efficacy in patients with EAC.

## Methods

### Sex as a biological variable.

Our patient cohort consists of both men and women. Given the low frequency of EACs among women, we do not consider sex as a biological variable.

### Cell lines and reagents.

Human embryonic kidney (HEK293), cervical adenocarcinoma (HeLa), human T cell leukemia (Jurkat), and human esophageal adenocarcinoma (OE33) cells were acquired from the ATCC. HEK293 and HeLa cells were grown in DMEM supplemented with 10% cosmic calf serum, while Jurkat and OE33 cells were grown in RPMI-1640 medium supplemented with 10% fetal bovine serum (FBS). Cells were routinely tested for any unwanted pathogen infection and genotyped for authenticity at the University of Michigan Advanced Genomics Core. For the majority of our experiments, cells were plated a day before the treatment. Plasmid transfections were performed using either calcium phosphate method (HEK293 cells) as described earlier (55) or Lipofectamine 2000 (Invitrogen) according to the manufacturer’s protocol. For knockdown studies using siRNA, we used Lipofectamine RNAiMAX (Invitrogen) as described earlier (56).

Antibodies against different CD3 isoforms were obtained as follows: CD3-ε (catalog 317326 from BioLegend and catalog 4443T from Cell Signaling Technology); CD3-δ (catalog 16044) and CD3-ζ (catalog 243874) were from Abcam. Various other antibodies included GFP (catalog 632381) from Takara and DDK/FLAG antibodies from MilliporeSigma (catalog F1804), Origene (catalog TA50011), or Cell Signaling Technology (catalog 14793S). ISG15 (catalog MA5-28371) and V5 (catalog 46-0705) antibodies were purchased from Invitrogen. Ubiquitin antibody (P4D1) and anti-Hsc70 (catalog sc-7298) were purchased from Santa Cruz Biotechnology. GAPDH (catalog 2118S) and phospho-specific antibodies against LCK-Y505 (catalog 2751T) and Src-Y416 (6943T) were purchased from Cell Signaling Technology. Affi-FLAG (M2) (catalog A2220) and V5 agarose beads (catalog A7345) were purchased from MilliporeSigma. His-tagged recombinant and active LRGG was purchased from Abcam (catalog ab268685). The CD3 microbeads were purchased from Miltenyi Biotec (catalog 130-097-043), and anti-CD3/CD28 beads were purchased from Dynabeads (catalog 11161D). The 7-AAD (catalog 420403), CD45-FITC (catalog 304005), CD3-APC/Fire 750 (catalog 300469), and CD19-BV750 (catalog 302261) were purchased from BioLegend. For immunofluorescence detection of recombinant His-tagged ISG15, we used we used 6x-His tag antibody (catalog MA1-21315) from Thermo Fisher Scientific.

### Constructs.

DDK-tagged CD3 constructs (catalog RC220512, RC214725, RC208276, and RC210010) were purchased from Origene. V5-tagged GRAIL1 and DDK-tagged GRAIL2 were reconstructed in the lab for optimum expression as reported earlier (27). DDK-tagged ISG15 construct (catalog RC206585) was purchased from Origene. CD3-δ-YFP was a gift from Nico Dantuma (Addgene plasmid 11951; http://n2t.net/addgene:11951; RRID:Addgene_11951). CD3-ζ-GFP was a gift from Matthew Krummel (Addgene plasmid 38298; http://n2t.net/addgene:38298; RRID:Addgene_38298). pCMV6-Neo-ISG15-LRAA was a gift from Isidoro Martinez (Addgene plasmid 80405; http://n2t.net/addgene:80405; RRID: Addgene_80405).

### Patients.

The samples are from patients who have stage 1 to stage 3 EAC with associated BE and low- or high-grade dysplasia. Patients with HGD who underwent esophagectomy are also included. For RNA-Seq analysis, tissue samples were collected from 65 chemo-naive patients undergoing curative resection for EAC or HGD at the University of Michigan Health System, as previously described (2) and available in Gene Expression Omnibus series GSE193946. For HG-U133A expression microarray analysis, tissue samples were collected from 46 chemo-naive patients undergoing curative resection for EAC or HGD at the University of Michigan Health System as previously described (57). For TMA to perform IHC analysis, esophageal tissues were collected at the time of curative resection surgery from a separate cohort of more than 350 patients diagnosed with HGD or EAC and undergoing curative cancer surgery at the University of Michigan Health System.

### Preparation of esophageal TMAs.

BE, LGD, HGD, and EAC samples drawn from the above cohort were paraffin-embedded for sectioning, as previously described (2). All chosen squamous epithelium, BE, LGD, and HGD tissues were acquired from patients presenting with HGD or EAC and were acquired from a region within 6 cm of the tumor. H&E-stained sections prepared by the University of Michigan Department of Pathology were used to verify disease pathology. To create the TMA, regions of the original tissue block were identified as areas of interest according to tissue pathology. From these regions, 2 to 3 cores were made for each patient’s tissue sample. Once generated, the TMA was sectioned and baked at 50°C for 24 hours to adhere the tissue sections.

### mIHC.

mIHC was performed using the Opal 7 Solid Tumor Immunology Kit (Akoya Biosciences) according to manufacturer’s instruction, as previously described (2, 28). TMA slides composed of esophageal, column-derived dysplasia and EAC were sectioned and baked 60°C for 1 hour. Slides were deparaffinized with 3 changes of 100% xylene, followed by rehydration in a series of graded ethanol to distilled water. Slides were then dipped in neutral buffered formalin to increase tissue section adherence prior to the first antigen retrieval step. Primary antibody sources and dilutions are listed below. Slides were counterstained with DAPI to visualize nuclei prior to mounting using ProLong Diamond (Thermo Fisher Scientific).

### Visualization and quantitation of mIHC slides.

Slides were imaged using either a Mantra Quantitative Pathology Imaging System (QPIS) or a PhenoImager HT (Akoya Biosciences). For Mantra QPIS imaging, 1 to 2 images per core were acquired using 20× original magnification. For the PhenoImager HT, entire slides were scanned at high resolution. Filters for DAPI, CY3, CY5, CY7, Texas red, and Qdot were applied to each image set for each core. To spectrally unmix and quantitate the individual fluorescence channels, a multispectral library was generated, which was used to unmix the image and identify specific staining for each antigen. Image files were analyzed using Phenochart 1.1.0 software followed by InForm 2.2.1 image analysis software, as previously described (2, 28). Background autofluorescence, defined as signal from unstained esophagus tissue, was subtracted per image. Spectral DAPI was used to identify nuclei. Target epithelial and stroma tissues were identified as previously described (2). Fluorescence intensities were used to identify and segment cell types into CD3-ε–, CD3-ζ–, CD8-, CD163-, FoxP3-, GRAIL1-, and PD-L1–positive cells. Tissue categories were identified separately by a pathologist as previously described (2).

### Antibodies for mIHC staining.

Primary antibodies used for mIHC are as follows at the indicated dilutions: Anti–CD3-ε (1:400; Agilent Technologies, catalog A045229-2), Anti–CD3-ζ (1:200; Abcam, catalog 243874), Anti-CD8 (1:400; Spring Bio, catalog M5390), Anti-CD163 (1:400; Leica, catalog NCL-L-CD163), Anti-FoxP3 (1:400, Cell Signaling Technology, catalog 12653S), GRAIL1 (1:1,000, R&D Systems, catalog AF6234), Anti–PD-L1 (1:200, Cell Signaling Technology, catalog 13684S), and Anti–Pan-Cytokeratin (1:500, Agilent Technologies, catalog M351529-2). Secondary Opal antibodies and TSA Fluorophore reagents were supplied in the Opal 7 Solid Tumor Immunology Kit and used at the recommended dilutions.

### Protein isolation and analyses.

Protein isolation was performed as described earlier (56). Briefly, for lysis, we used a buffer containing 50 mM HEPES/KOH, 150 mM NaCl, 1 mM EDTA, 2.5 mM EGTA, 1 mM N-ethylmaleimide, 1 mM NaF, 100 μM sodium orthovandate, 10% glycerol, 10 mM β-glycerophosphate, 0.1% NP-40, and protease inhibitor cocktail. All steps in protein isolation were performed on ice. Following a brief incubation (5 minutes), samples were sonicated and centrifuged at 15,500*g* for 5 minutes, and supernatants were transferred to a new tube. Protein estimation was performed using the Bradford method. A 4× loading buffer containing 250 mM Tris (pH 6.8), 40% glycerol, 4% SDS, 12.5 mM EDTA, 10% β-mercaptoethanol, and 0.08% bromophenol blue was added to each sample and boiled for 5 minutes before snap-freezing. Equal amounts (mg) of proteins were then subjected to immunoblotting using 4%–12% precast Bis-Tris gel (Invitrogen). For immunoblotting, different dilutions of the primary antibodies were used, membranes were incubated overnight at 4°C, and bound antibody was detected using an enhanced chemiluminescence reagent.

### Quantification of s-ISG15 in culture supernatant.

For these studies, cells were grown in low-serum-containing (0.5%–1%) medium to reduce IgG heavy chain contamination signal at the time of immunoblotting. On the day of harvest, supernatants were collected in a microfuge tube (Thermo Fisher Scientific)and spun down at 9,200*g* for 1 minute to remove cellular debris. A total of 500 μL of cleared supernatant was then transferred into the filter column (Amicon, Ultracel-0.5, 3 kDa centrifugal filters; UFC500396, Merck Millipore), which was placed inside the collection tube. It was then centrifuged at 13,200*g* for 20 minutes at 4°C. The flow-through was discarded and the remaining concentrate (50–60 μL) was then subjected to immunoblotting for s-ISG15 quantification.

### Site-directed mutagenesis.

To create site-specific mutations in GRAIL1, we used Quick Change mutagenesis kit from Stratagene according to manufacturer’s protocol. To confirm mutations, isolated plasmids were sequenced using Sanger sequencing at the GENEWIZ from Azenta Life Sciences and analyzed.

### Mass spectrometry.

To determine GRAIL1 interactors, we used CpA and CpD cells and transfected them with either vehicle control or V5-tagged GRAIL1. Twenty-four hours after transfection, cell lysates were prepared and subjected to immunoprecipitation as described above. Following pulldown using V5 beads and washing with 0.1% NP-40 containing lysis buffer and a final wash using PBS, the beads were resuspended in 50 mL of 0.1 M ammonium bicarbonate buffer (pH ~8). Cysteines were reduced by adding 50 mL of 10 mM DTT and incubating at 45°C for 30 minutes. Samples were cooled to room temperature and alkylation of cysteines was achieved by incubating with 65 mM 2-Chloroacetamide, under darkness, for 30 minutes at room temperature. An overnight (~16 hours) digestion with 1 μg sequencing-grade, modified trypsin was carried out at 37°C with constant shaking in an Eppendorf Thermomixer. Digestion was stopped by acidification and peptides were desalted using SepPak C18 cartridges using manufacturer’s protocol (Waters). Samples were completely dried using vacufuge. Resulting peptides were dissolved in 8 mL of 0.1% formic acid, 2% acetonitrile solution, and 2 mL of the peptide solution was resolved on a nano-capillary reverse-phase column (Acclaim PepMap C18, 2 micron, 50 cm, Thermo Fisher Scientific) using a 0.1% formic acid, 2% acetonitrile (buffer A), and 0.1% formic acid, 95% acetonitrile (buffer B), gradient at 300 nL/min over 180 minutes (2%–22% buffer B for 110 minutes, 22%–40% for 25 minutes, 40%–90% for 5 minutes followed by holding at 90% buffer B for 5 minutes and re-equilibration with buffer A for 25 minutes). Eluent was directly introduced into Orbitrap Fusion Tribrid mass spectrometer (Thermo Fisher Scientific) using an EasySpray source. MS1 scans were acquired at 120,000 resolution (automatic gain control target (AGC) = 2 × 10^5^; max intensity (IT) = 100 ms). Data-dependent high-energy C-trap dissociation MS/MS spectra were acquired using top speed method (3 seconds) following each MS1 scan (normalized collision energy ~32%; AGC target 5 × 10^4^; max IT 50 ms, 15,000 resolution).

Proteins were identified by searching the MS/MS data against *H*. *sapiens* database (UniProt; 28476 reviewed entries; downloaded on August 29, 2018) using Proteome Discoverer (v2.1, Thermo Fisher Scientific). Search parameters included MS1 mass tolerance of 10 parts per million and fragment tolerance of 0.2 Da; 2 missed cleavages were allowed; carbamidimethylation of cysteine was considered fixed modification; and oxidation of methionine and deamidation of aspergine and glutamine were considered potential modifications. False discovery rate (FDR) was determined using Percolator, and proteins/peptides with an FDR of ≤1% were retained for further analysis.

### Immunofluorescence studies.

Immunofluorescence staining was performed as described previously (56). Briefly, fixed cells were washed in PBS, permeabilized with 0.2% Triton X-100 on ice for 5 minutes, blocked for 1 hour, and then incubated with primary antibody at 4°C overnight in a humidified chamber. The slides were then washed again in PBS-Tween, incubated with the fluorescence-conjugated secondary antibody for 45 minutes, rewashed, and prepared with a coverslip after a drop of ProLong Gold anti-fade reagent with DAPI (Molecular Probes) was added to each sample. Fluorescence images were acquired using an Olympus 1X-71 microscope.

### Effect of exogenous ISG15 on CD3 levels.

For this study we used a recombinant N-terminal His-tagged human active ISG15 (C-terminal LRGG sequence exposed). Cells (either Jurkat or PBMCs) were plated 1 day in advance in complete media. Cells were then treated with recombinant His-ISG15 (250 ng/mL) for different periods. Cells were then subjected to either immunoblotting or immunofluorescence as described above.

PBMCs were isolated and processed from the peripheral human blood as described previously (58). CD3^+^ T cells were isolated using CD3 microbeads. As previously described, the frequency was assessed 24 hours after activation using anti-CD3/CD28 beads (59). Stainings were performed in accordance with the manufacturer’s instructions (BioLegend). Briefly, after dead cell exclusion through 7-AAD viability staining solution, cells were stained for surface markers and incubated for 20 minutes at 4°C with a cocktail of CD45 FITC, CD3 APC/Fire 750, and CD19 BV750 to exclude biphenotypic CD3^+^ T cells (60), in FACS buffer (PBS, 2% FBS). Cells were washed twice with FACS buffer before running on the flow cytometer. Samples were analyzed following a 24-hour culture in a complete medium at 37°C, in the presence of ISG15 (250 ng/mL) or PBS control. Samples were acquired on the 4-laser Cytek Aurora and analyzed with FlowJo software, version 10 (TreeStar).

### Protein half-life studies.

Either HeLa or HEK293 cells were transfected with different CD3 isoforms in the presence and absence of GRAIL1 and incubated overnight. Twenty-four hours after transfection, cycloheximide (50 mg/mL) was added, and cells were harvested at the indicated time points. To determine CD3-ζ dimer half-life, cells were treated with DSS (1 μg/mL) for the last 30 minutes prior to harvest. Immunoblotting was performed for DDK and Hsp70 to analyze the protein half-life of CD3-ε, -γ, -δ, and -ζ. The approximate CD3 protein half-life (t_1/2_) in the absence and presence of GRAIL1 was calculated using data (mean ± SEM) from 3 independent experiments by plotting relative band density (arbitrary units) and time (h) in log-linear scales.

### Statistics.

For mIHC analysis all graphs were generated and data analyzed using GraphPad Prism v 9.3.1. Statistical analysis of cell count data for each immunologic marker was performed using 1-way ANOVA followed by Tukey’s multiple-comparison test to determine significant differences among the different cell types. Survival curves were generated using GraphPad Prism software. Statistical significance was determined using a log-rank Mantel-Cox regression test. For all analyses, *P* < 0.05 was considered statistically significantly different. All histograms are presented as the mean value ± SEM.

### Study approval.

All patients provided written informed consent, and all experimental protocols were approved by the University of Michigan Institutional Review Board and Ethics Committee. The methods were carried out in accordance with approved guidelines.

### Data availability.

The data generated in this study are available within the article and its supplemental and Supporting Data Values files.

## Author contributions

DPM, PR, DJN, DT, DGB, KHL, and DR were responsible for experimental design and study concept. DPM, PR, ZW, DT, SK, SA, AB, RH, KHL, and DR were responsible for development of methodology. DGB and KHL provided patient sample material and clinical information. DPM, PR, DJN, DGB, SA, KHL, and DR were responsible for statistical analysis and data interpretation. DGB, KHL, and DR were responsible for study supervision. DPM, PR, DJN, RH, TSL, DGB, KHL, and DR were responsible for manuscript preparation.

## Supplementary Material

Supplemental data

Unedited blot and gel images

Supporting data values

## Figures and Tables

**Table 1:**
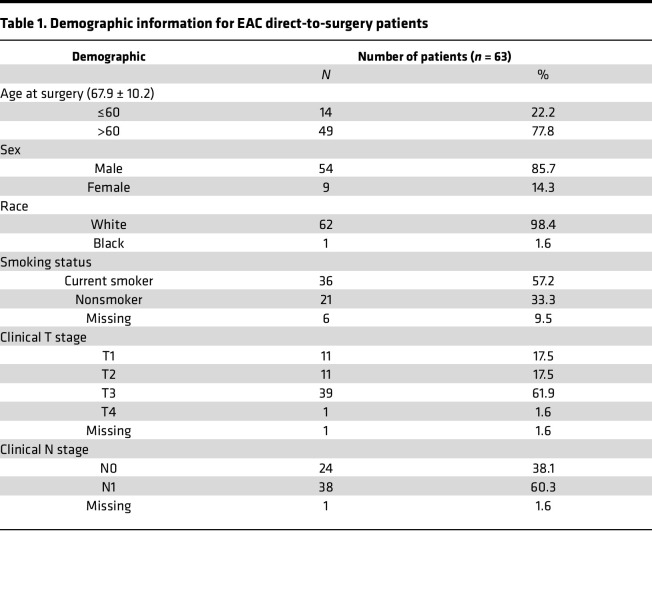
Demographic information for EAC direct-to-surgery patients

**Table 2:**
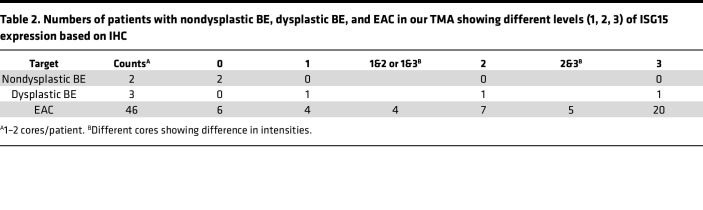
Numbers of patients with nondysplastic BE, dysplastic BE, and EAC in our TMA showing different levels (1, 2, 3) of ISG15 expression based on IHC

## Version 1 05/23/2024

In-Press Preview

## Version 2 07/08/2024

Electronic publication
