# Supplementary material for: ISG15/GRAIL1/CD3 axis influences survival of patients with esophageal adenocarcinoma
Source: JCI Insight. 2024 May 23;9(13):e179315. doi: 10.1172/jci.insight.179315 (PMC11383178; doi:10.1172/jci.insight.179315)
Supplement: Supplemental data [file jciinsight-9-179315-s076.pdf]

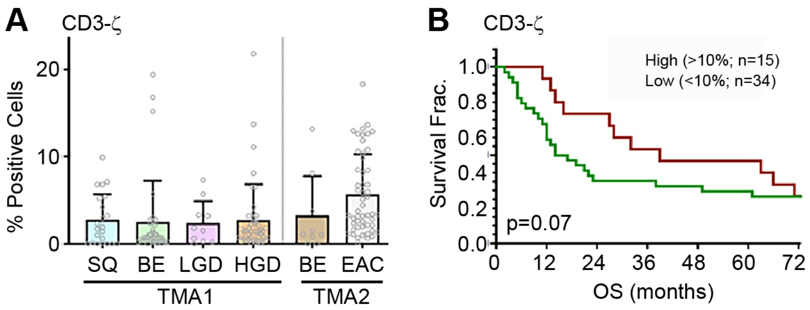

Supplemental Figure 1. CD3ζ expression increases during progression from BE to EAC, with only minimal effects on patient OS. (A) BE, LGD, HGD, or EAC tissue was incorporated into a TMA and stained for CD3ζ. Positive cells per core were counted ad expressed as a percentage of the total cell number. (**B)** Survival analysis shows no significant effects of CD3ζ high vs. low expressing cell on overall patient survival. Survival curves differences were determined using Mantel-Cox regression analysis.

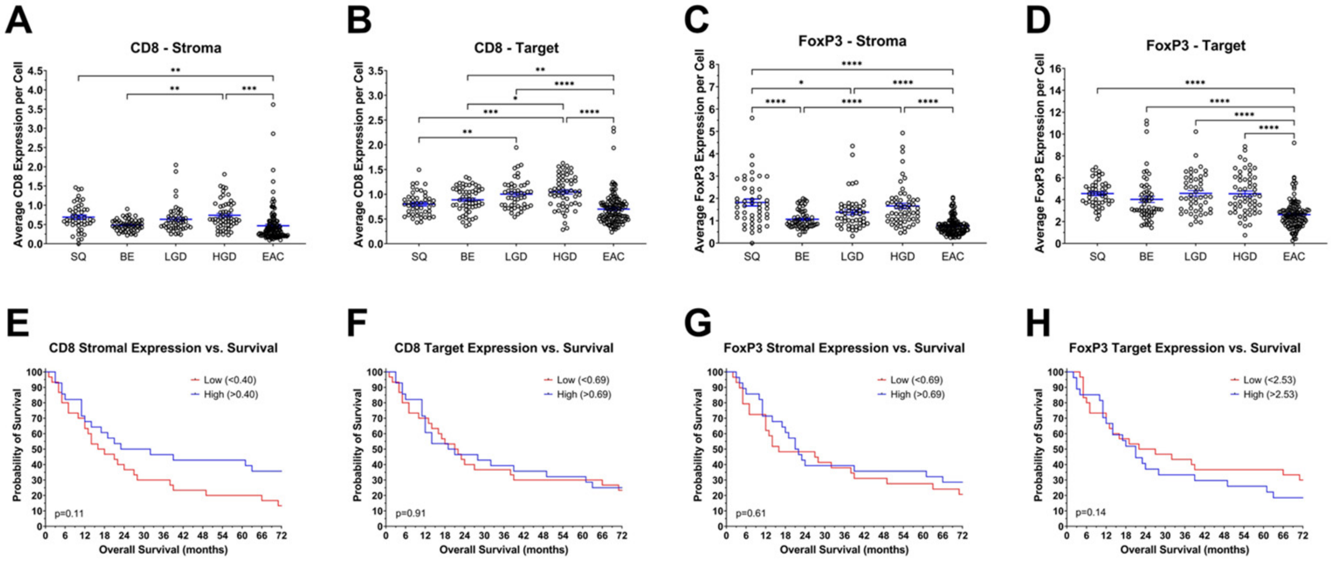

Supplemental Figure 2. Effector and Regulatory T-cell populations changes during progression from BE to EAC and their effects on overall patient survival. A-B, Effector T-cell populations were identified as $CD8^+$ cell populations and the average expression per cell was determined using the Akoya InForm software. Target tissue was identified as CK^+ tissue. $CD8^+$ T-cell populations increase incrementally during progression from BE to HGD in Stroma (A) and Target (B) tissue, respectively. However, EAC tissues are immune poor for effector T-cells. **C-D,** Similarly, FoxP3 expression in T-regulatory cells increase during progression from BE to HGD. However, EAC tissues show low levels of FoxP3+ T-cell populations in either Stroma (C) or Target (D) tissues, respectively. **E-H,** Survival analysis shows no significant effects of either $CD8^+$ high vs. low expressing cell populations or FoxP3+ high vs. low expression cell populations on overall patient survival. Survival curves differences were determined using Mantel-Cox regression analysis.

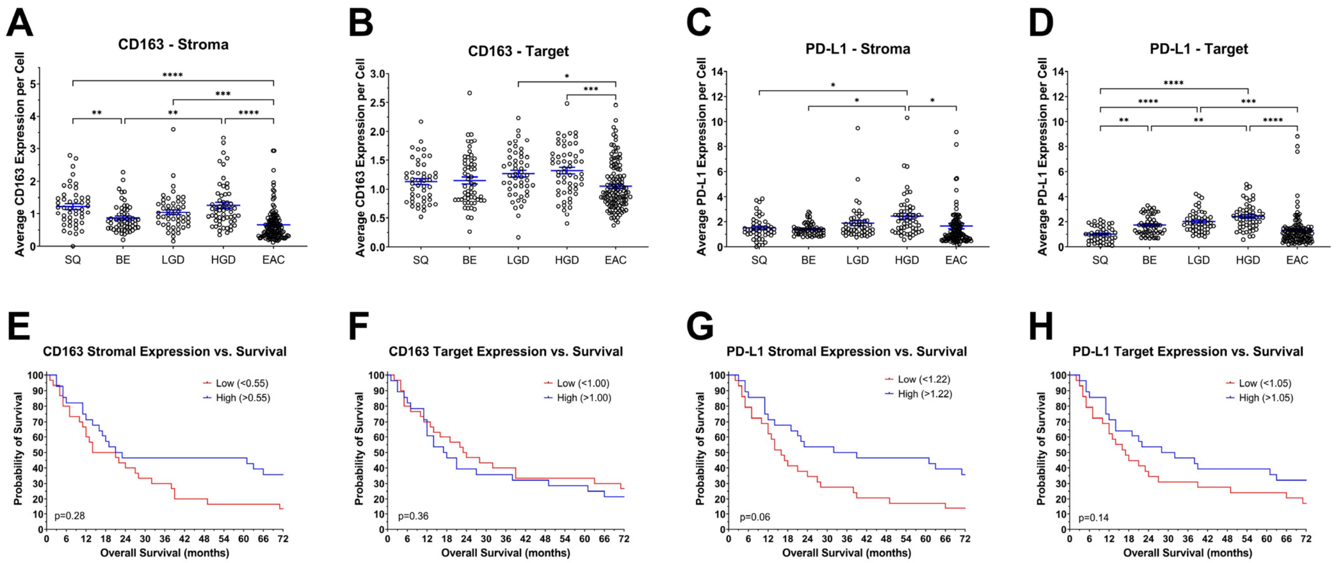

Supplemental Figure 3. Antigen-presenting cell populations change during progression from BE to EAC and their effects on overall patient survival. (A-B) Macrophages were identified as $CD163^{+}$ cell populations and the average expression per cell was determined using the Akoya InForm software. Target tissue was identified as CK^{+} tissue. $CD163^{+}$ macrophages increase incrementally during progression from BE to HGD in Stroma (A) and Target (B) tissue, respectively. However, EAC tissues are immune poor for macrophages. (**C-D)** Similarly, PD-L1 expression in APCs increases during progression from BE to HGD. However, EAC tissues show low levels of PD-$L1^{+}$ APCs in either Stroma (C) or Target (D) tissues, respectively. (**E-H)** Survival analysis shows no significant effects of either $CD163^{+}$ high vs. low expressing cell populations or PD-L1+ high vs. low expression cell populations on overall patient survival. Survival curves differences were determined using Mantel-Cox regression analysis.

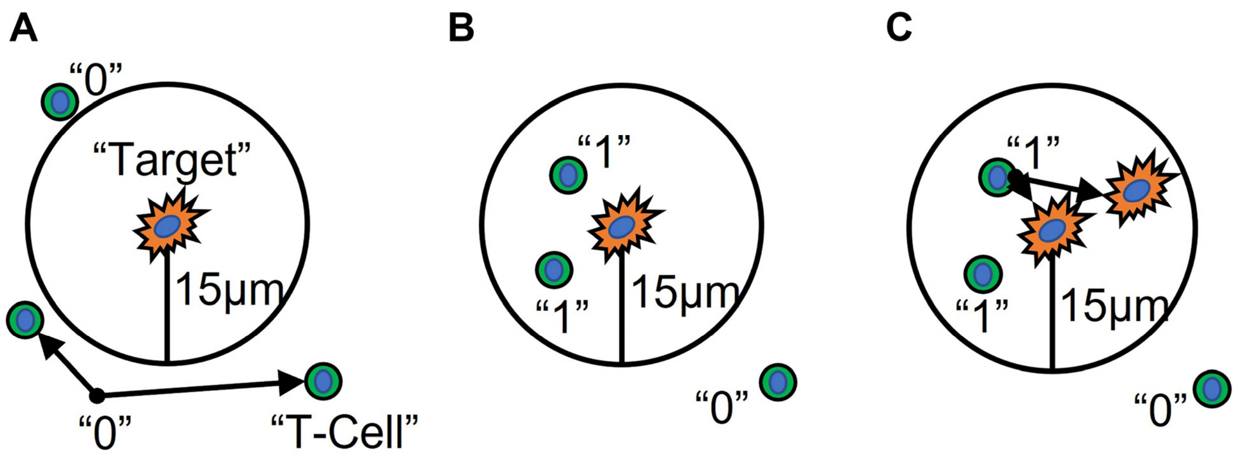

Supplemental Figure 4. Cell Engagement Analysis for T-Cell engagement with epithelium or APCs. (A-C) Multiplex stained images were analyzed using Akoya InForm cell analysis software. Cartoon representation of engagement analysis. PanCK+ epithelial cells, CD163+ macrophages, or PD-L1+ APCs were identified by positive staining as target cells. From each positive target cell, the analysis software determined whether CD3+, CD8+, or FoxP3+ T-cells were within a 15µm radius of the target cell and considered "engaged". A cell outside the radius was given a "0" value as a non-engaged cell (A). Any T-cell within the 15µm radius was given a value of "1" and scored as "engaged" (B). This scoring system prevented T-cells from being counted multiple times, as shown in (C).

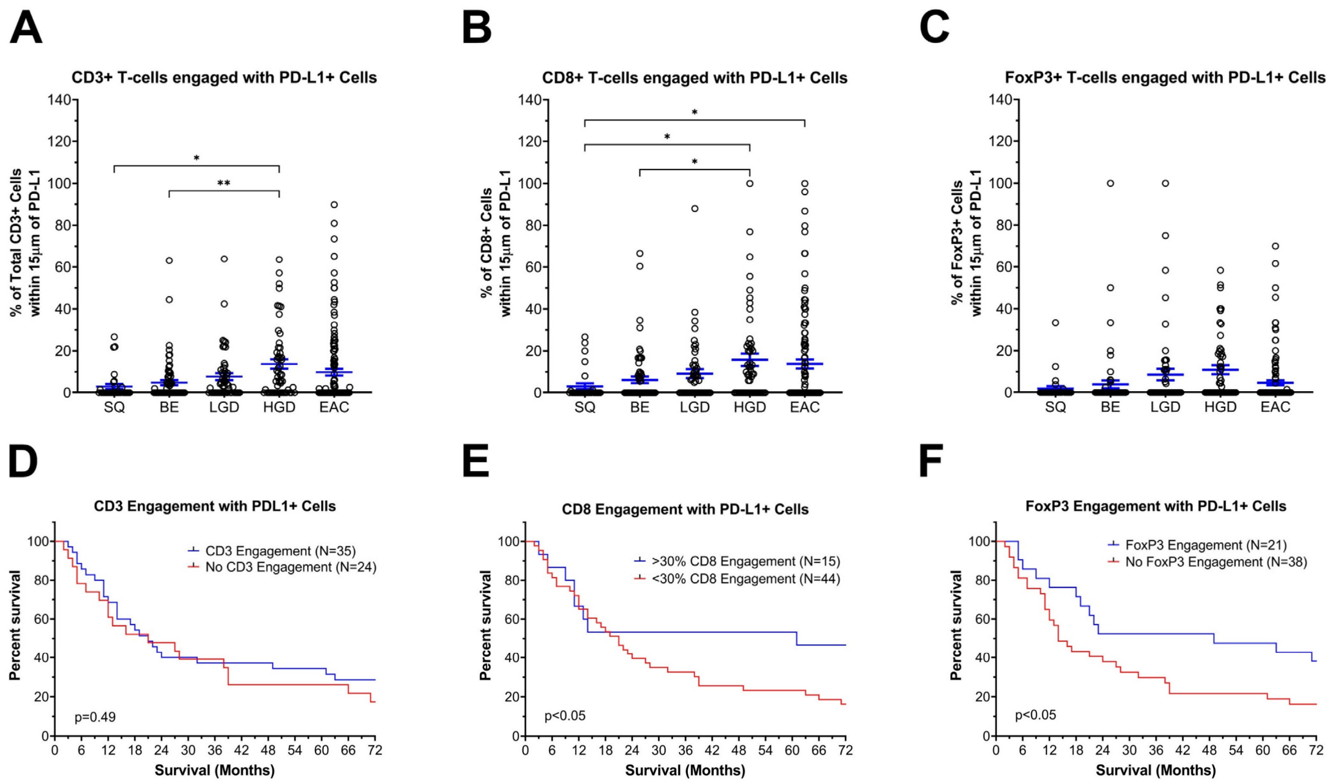

Supplemental Figure 5. T-cell engagement with PD-L1+ APCs minimally affects patient OS. (A) CD3+ T-cells within 15µm of a PD-L1+ APC were counted as "engaged" with the epithelium. The percentage of engaged cells was determined by dividing the number of CD3+ engaged cells by the total number of CD3+ T-cells and multiplying by 100. T-cell engagement increased during progression from BE to HGD. However, CD3+ T-cell engagement within EAC tissue decreased compared to HGD. (**B)** CD8+ effector T-cells within 15µm of a PD-L1+ APC were counted as "engaged" with the epithelium. The percentage of engaged cells was determined by dividing the number of CD8+ engaged cells by the total number of CD8+ T-cells and multiplying by 100. Effector T-cell engagement increased during progression from BE to HGD. However, CD8+ T-cell engagement within EAC tissue decreased compared to HGD. **(C)** FoxP3+ T-regulatory cells within 15µm of a PD-L1+ APC were counted as "engaged" with the epithelium. The percentage of engaged cells was determined by dividing the number of FoxP3+ engaged cells by the total number of FoxP3+ T-cells and multiplying by 100. T-regulatory cell engagement increased during progression from BE to HGD. However, FoxP3+ T-regulatory cell engagement within EAC tissue decreased compared to HGD. (**D-F)** Survival analysis shows no significant effects of $CD3^{+}$ **(D)** on OS. However, high $CD8^{+}$ **(E)**, or $FoxP3^{+}$ **(F)** high engaged tissues had marginally better overall patient survival compared to low engaged tissues. Survival curves differences were determined using Mantel-Cox regression analysis.

```
Sequence 1: Zeta        164 aa
Sequence 2: Delta       171 aa
Sequence 3: Epsilon     207 aa
Sequence 4: Gamma       182 aa

Sequences (1:2) Aligned. Score: 11.5854
Sequences (1:3) Aligned. Score: 11.5854
Sequences (1:4) Aligned. Score: 14.0244
Sequences (2:3) Aligned. Score: 15.2047
Sequences (2:4) Aligned. Score: 36.2573
Sequences (3:4) Aligned. Score: 15.3846


Delta          ---MEHSTFLSGLVLATLLSQVSP---------FKIPIEELEDRVFVNCN----TSITWV
Gamma          ---MEQGKGLAVLILAIILLQGTLAQSIKGNHLVKVYDYQEDGSVLLTCDAE-AKNITWF
Epsilon        MQSGTHWRVLGLCLLSVGVWGQDGNEEMGGITQTPYKVSISGTTVILTCPQYPGSEILWQ
Zeta           -------MKWKALFTAAILQAQLP----------ITEAQSFGLLDPKLCYLL--DGILFI
                            . :  :                              *       * :

Delta          EG--TVGTLLSDITRLDLG-------KRILDPRGIYRCNGTDIY-KDKESTVQVHYRMCQ
Gamma          KDGKMIGFLTEDKKKWNLG-------SNAKDPRGMYQCKGS----QNKSKPLQVYYRMCQ
Epsilon        HNDKNIGGDEDDKNIGSDEDHLSLKEFSELEQSGYYVCYPRGSKPEDANFYLYLRARVCE
Zeta           YGVILTALFLRVKFSRSAD--------APAYQQGQNQLYNELNLGRREEYDVLDKRRGRD
                .    .         .                *           .  .  :    *  :

Delta          SCVELDPATVAGIIVTDVIATLLLALGVFCFAGH-------------ETGRLSGAADTQA
Gamma          NCIELNAATISGFLFAEIVSIFVLAVGVYFIAGQ-------------DGVRQSRASDKQT
Delta          LLRNDQVYQPLRDRDDAQYSHLGGNWARNK 'WSKNRKAKAKPVTRGAGAGGRQRGQNKERP
Gamma          LLPNDQLYQPLKDREDDQYSHLQGNQLRRN,'SEIG------------------MKGERRR
Epsilon        PPVPNPDYEPIRKGQRDLYSGLNQRRI---
                                                                      . :
Zeta           GKGHDGLYQGLSTATKDTYDALHMQALPPR
                   :  *: :        *. *   .
```

Supplemental Figure 6. Pairwise amino acid sequence alignments between different CD3 isoforms (ε, δ, γ, ζ) performed using CLASTALW multiple sequence alignment tool. Top panel includes alignment score showing CD3-δ and CD3-γ with highest alignment score of ~36%. '*' indicates that the residues are identical.

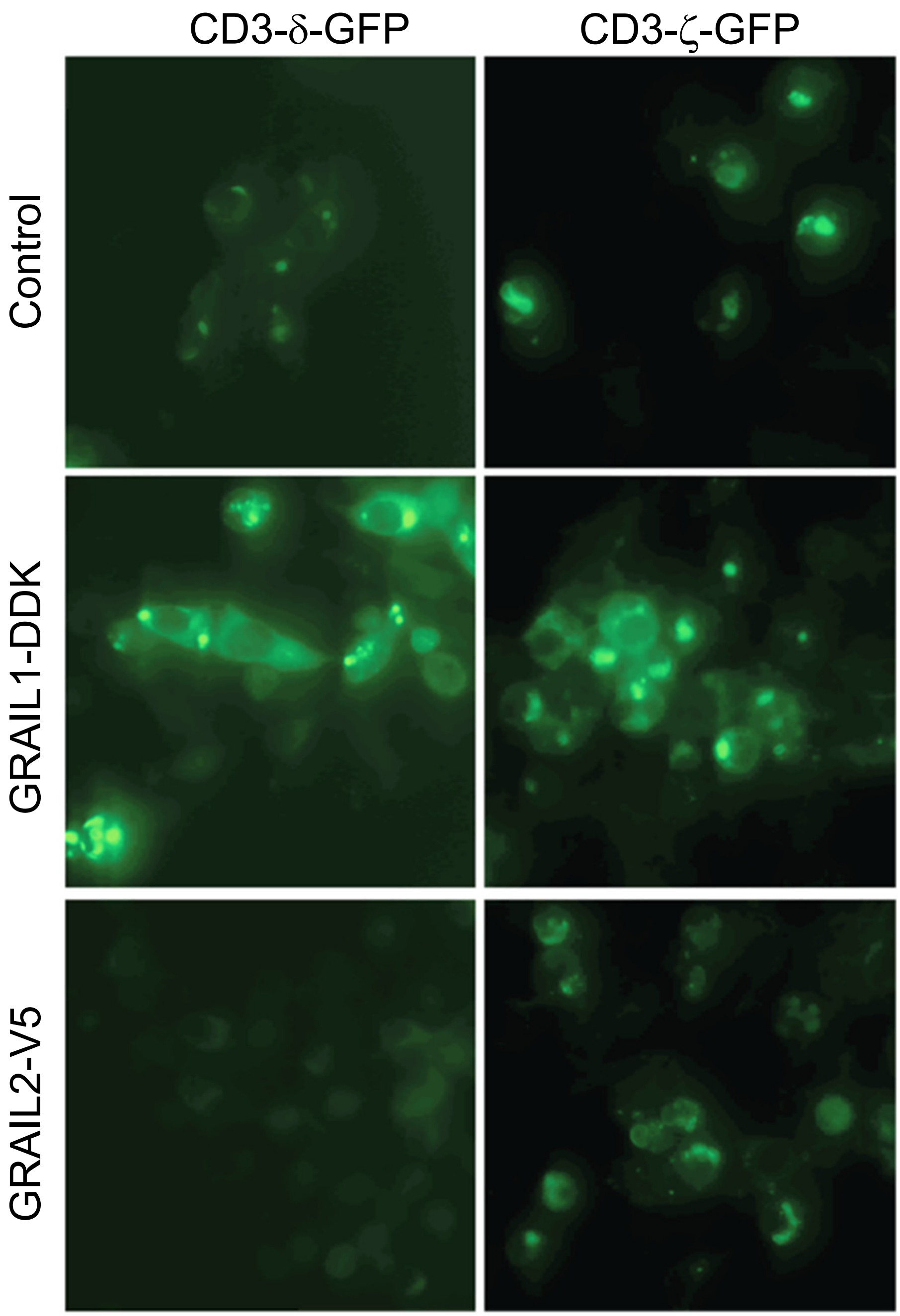

Supplemental Figure 7. Live cell imaging of HeLa cells overexpressing either GFP-tagged CD3-δ or CD3-ζ in the presence or absence of GRAIL1-DDK or GRAIL2-V5. For this study, cells were plated one day in advance followed by transfection using indicated plasmids. Images were taken 24 hours post-transfection.

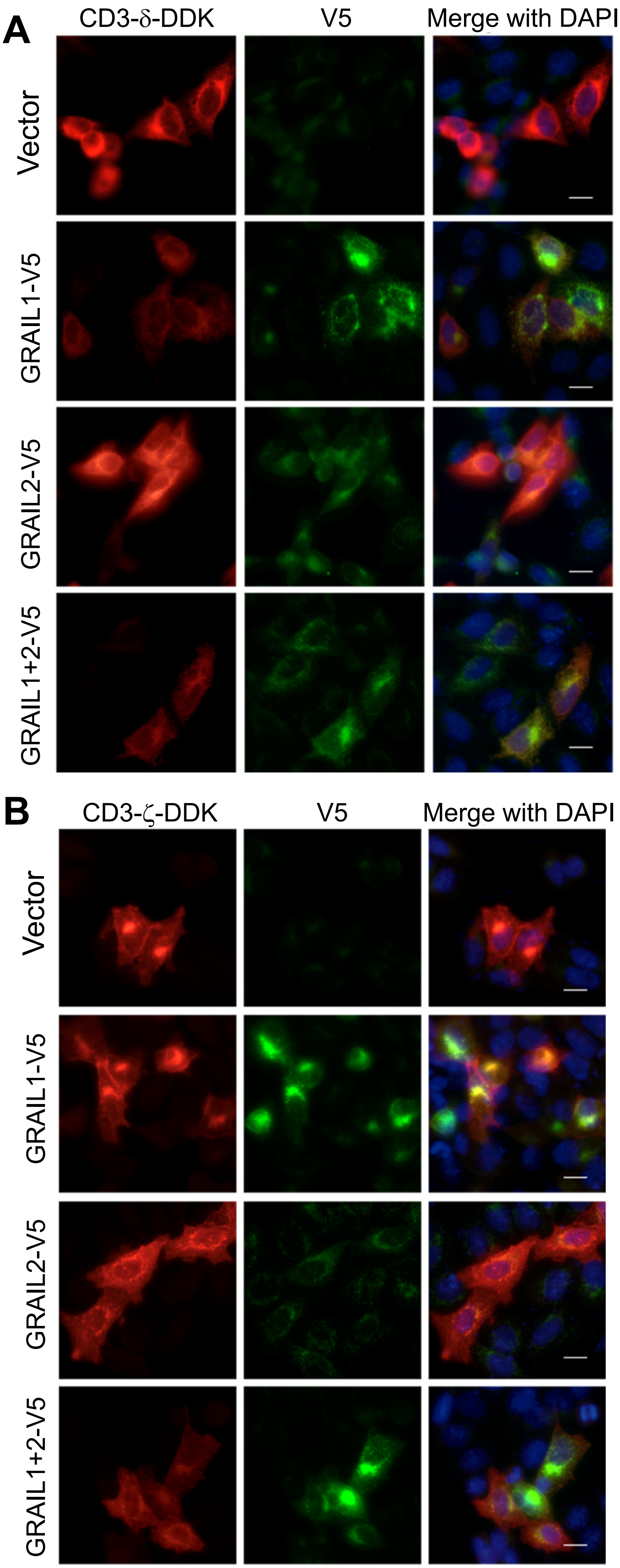

Supplemental Figure 8. Immunofluorescence (IF) staining of HeLa cells **(A)** DDK-tagged CD3-δ and **(B)** CD3-ζ in the presence and absence of either V5-tagged GRAIL1, GRAIL2, or two together as labeled. Twenty-four following transfection, cells were fixed with 10% buffered formalin and subjected to IF staining using DDK antibody (for CD3) or V5 antibody (for GRAIL). DAPI was used for nuclear staining. Scale bar, 10 μm.

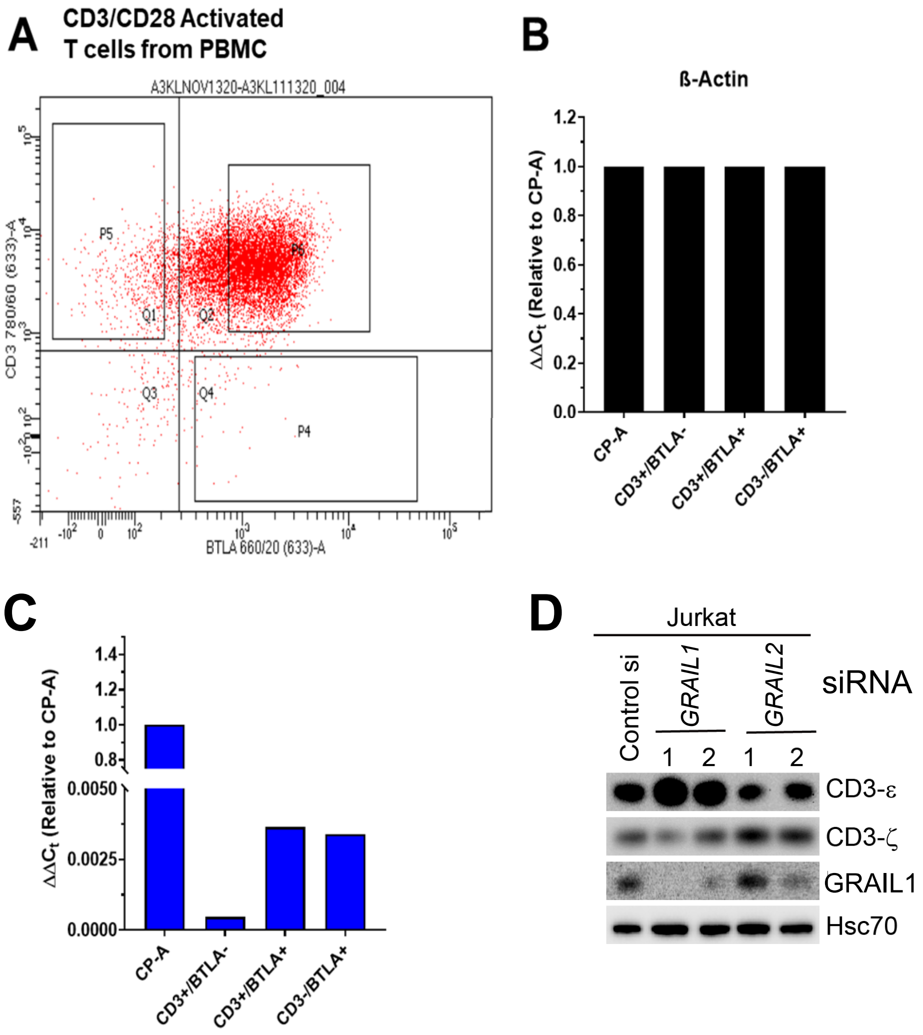

Supplemental Figure 9. Sorted T-cells from patient PBMCs were induced with αCD3-CD28 Dynabeads and were cultured for 10 days to promote T-cell exhaustion. **(A)** Cells were subjected to FACs analysis based on CD3 and BTLA expression. **(B, C)** Sorted cells as indicated were used to isolate RNAs to quantify GRAIL expression and b-actin was used as a loading control. **(D)** Jurkat cells were either transfected with control siRNA or 2 different sets of siRNA for *GRAIL1* and *GRAIL2* as indicated. Forty-eight hours post transfection, cell lysates were prepared and subjected to immunoblotting using indicated antibodies. Hsp70 was used as a loading control.

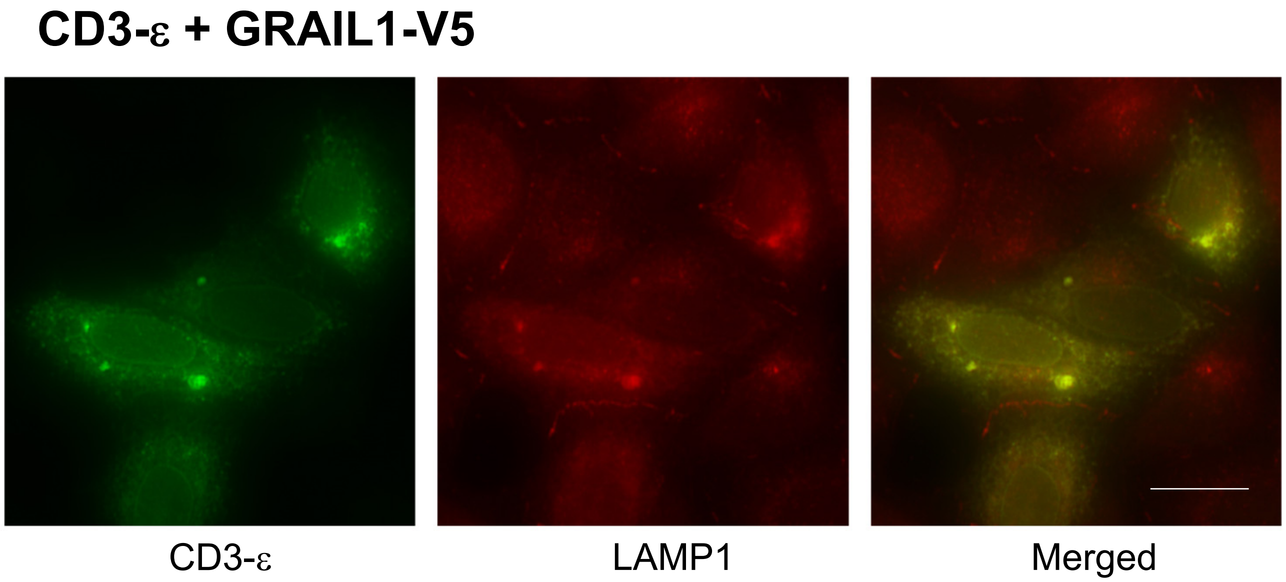

Supplemental Figure 10. DDK tagged CD3-ε when overexpressed along with V5-tagged GRAIL1, showed punctate CD3 staining. Immunofluorescence images showing CD3-ε and LAMP1 colocalization when overexpressed with GRAIL1, suggesting GRAIL mediated CD3-ε degradation is at the lysosome. Scale bar, 10 μm.

CP-A

Cp-D

Protein FDR Confidence	Accession	SYMBOL	Description	Exp. q-value
High	Q15149	' PLEC	Plectin OS=Homo sapiens OX=9606 GN=PLEC PE=1 SV=3	0
High	Q15149-4	' PLEC	Isoform 4 of Plectin OS=Homo sapiens OX=9606 GN=PLEC	0
High	Q15149-3	' PLEC	Isoform 3 of Plectin OS=Homo sapiens OX=9606 GN=PLEC	0
High	Q15149-8	' PLEC	Isoform 8 of Plectin OS=Homo sapiens OX=9606 GN=PLEC	0
High	P35579	' MYH9	Myosin-9 OS=Homo sapiens OX=9606 GN=MYH9 PE=1 SV=4	0
High	Q13813	' SPTAN1	Spectrin alpha chain, non-erythrocytic 1 OS=Homo sapiens OX=9606	0
High	Q13813-2	' SPTAN1	Isoform 2 of Spectrin alpha chain, non-erythrocytic 1 OS=Homo sapi	0
High	Q01082	' SPTBN1	Spectrin beta chain, non-erythrocytic 1 OS=Homo sapiens OX=9606	0
High	Q01082-3	' SPTBN1	Isoform 2 of Spectrin beta chain, non-erythrocytic 1 OS=Homo sapie	0
High	Q09666	' AHNAK	Neuroblast differentiation-associated protein AHNAK OS=Homo sap	0
High	O75369	' FLNB	Filamin-B OS=Homo sapiens OX=9606 GN=FLNB PE=1 SV=2	0
High	O75369-8	' FLNB	Isoform 8 of Filamin-B OS=Homo sapiens OX=9606 GN=FLNB	0
High	P15924	' DSP	Desmoplakin OS=Homo sapiens OX=9606 GN=DSP PE=1 SV=3	0
High	O43707	' ACTN4	Alpha-actinin-4 OS=Homo sapiens OX=9606 GN=ACTN4 PE=1 SV=2	0
High	P46940	' IQGAP1	Ras GTPase-activating-like protein IQGAP1 OS=Homo sapiens OX=96	0
High	P49327	' FASN	Fatty acid synthase OS=Homo sapiens OX=9606 GN=FASN PE=1 SV=	0
High	P35580-3	' MYH10	Isoform 3 of Myosin-10 OS=Homo sapiens OX=9606 GN=MYH10	0
High	P21333	' FLNA	Filamin-A OS=Homo sapiens OX=9606 GN=FLNA PE=1 SV=4	0
High	Q14764	' MVP	Major vault protein OS=Homo sapiens OX=9606 GN=MVP PE=1 SV=4	0
High	P12814	' ACTN1	Alpha-actinin-1 OS=Homo sapiens OX=9606 GN=ACTN1 PE=1 SV=2	0
High	Q92614	' MYO18A	Unconventional myosin-XVIIIa OS=Homo sapiens OX=9606 GN=MYO	0
High	Q14204	' DYNC1H1	Cytoplasmic dynein 1 heavy chain 1 OS=Homo sapiens OX=9606 GN	0
High	P46013	' MKI67	Proliferation marker protein Ki-67 OS=Homo sapiens OX=9606 GN=	0
High	P35527	' KRT9	Keratin, type I cytoskeletal 9 OS=Homo sapiens OX=9606 GN=KRT9	0
High	P78527	' PRKDC	DNA-dependent protein kinase catalytic subunit OS=Homo sapiens	0
High	P02545	' LMNA	Prelamin-A/C OS=Homo sapiens OX=9606 GN=LMNA PE=1 SV=1	0
High	P04264	' KRT1	Keratin, type II cytoskeletal 1 OS=Homo sapiens OX=9606 GN=KRT1	0
High	P09874	' PARP1	Poly [ADP-ribose] polymerase 1 OS=Homo sapiens OX=9606 GN=PA	0
High	P60709	' ACTB	Actin, cytoplasmic 1 OS=Homo sapiens OX=9606 GN=ACTB PE=1 SV=	0
High	P08727	' KRT19	Keratin, type I cytoskeletal 19 OS=Homo sapiens OX=9606 GN=KRT1	0
High	P05787-2	' KRT8	Isoform 2 of Keratin, type II cytoskeletal 8 OS=Homo sapiens OX=96	0
High	P13645	' KRT10	Keratin, type I cytoskeletal 10 OS=Homo sapiens OX=9606 GN=KRT1	0
High	P35908	' KRT2	Keratin, type II cytoskeletal 2 epidermal OS=Homo sapiens OX=9606	0
High	P05783	' KRT18	Keratin, type I cytoskeletal 18 OS=Homo sapiens OX=9606 GN=KRT1	0
High	Q00610	' CLTC	Clathrin heavy chain 1 OS=Homo sapiens OX=9606 GN=CLTC PE=1 S	0
High	P16144-2	' ITGB4	Isoform Beta-4A of Integrin beta-4 OS=Homo sapiens OX=9606 GN=	0
High	Q16787-1	' LAMA3	Isoform 1 of Laminin subunit alpha-3 OS=Homo sapiens OX=9606 G	0
High	P02545-2	' LMNA	Isoform C of Prelamin-A/C OS=Homo sapiens OX=9606 GN=LMNA	0
High	P11388-4	' TOP2A	Isoform 4 of DNA topoisomerase 2-alpha OS=Homo sapiens OX=960	0
High	Q9UPN3	' MACF1	Microtubule-actin cross-linking factor 1, isoforms 1/2/3/5 OS=Homo	0
High	P14618	' PKM	Pyruvate kinase PKM OS=Homo sapiens OX=9606 GN=PKM PE=1 SV	0
High	Q9NYF8	' BCLAF1	Bcl-2-associated transcription factor 1 OS=Homo sapiens OX=9606 G	0
High	O14974	' PPP1R12A	Protein phosphatase 1 regulatory subunit 12A OS=Homo sapiens OX	0
High	Q9NYF8-3	' BCLAF1	Isoform 3 of Bcl-2-associated transcription factor 1 OS=Homo sapier	0
High	Q13751	' LAMB3	Laminin subunit beta-3 OS=Homo sapiens OX=9606 GN=LAMB3 PE=	0
High	O95425-4	' SVIL	Isoform SV4 of Supervillin OS=Homo sapiens OX=9606 GN=SVIL	0
High	O14974-4	' PPP1R12A	Isoform 4 of Protein phosphatase 1 regulatory subunit 12A OS=Hom	0
High	P14618-2	' PKM	Isoform M1 of Pyruvate kinase PKM OS=Homo sapiens OX=9606 GN	0
High	Q13753	' LAMC2	Laminin subunit gamma-2 OS=Homo sapiens OX=9606 GN=LAMC2 F	0
High	Q9UQ35	' SRRM2	Serine/arginine repetitive matrix protein 2 OS=Homo sapiens OX=96	0
High	P07996	' THBS1	Thrombospondin-1 OS=Homo sapiens OX=9606 GN=THBS1 PE=1 SV	0
High	P53621	' COPA	Coatomer subunit alpha OS=Homo sapiens OX=9606 GN=COPA PE=	0
High	Q9P2E9	' RRBP1	Ribosome-binding protein 1 OS=Homo sapiens OX=9606 GN=RRBP1	0
High	Q9BY89	' KIAA1671	Uncharacterized protein KIAA1671 OS=Homo sapiens OX=9606 GN=	0
High	P02538	' KRT6A	Keratin, type II cytoskeletal 6A OS=Homo sapiens OX=9606 GN=KRT	0
High	Q9C0C2	' TNKS1BP1	182 kDa tankyrase-1-binding protein OS=Homo sapiens OX=9606 GN	0
High	O94832	' MYO1D	Unconventional myosin-Id OS=Homo sapiens OX=9606 GN=MYO1D	0
High	P04259	' KRT6B	Keratin, type II cytoskeletal 6B OS=Homo sapiens OX=9606 GN=KRT	0
High	Q6WCQ1-	' MPRIP	Isoform 2 of Myosin phosphatase Rho-interacting protein OS=Homo	0
High	Q9Y4I1	' MYO5A	Unconventional myosin-Va OS=Homo sapiens OX=9606 GN=MYO5A	0
High	P07437	' TUBB	Tubulin beta chain OS=Homo sapiens OX=9606 GN=TUBB PE=1 SV=2	0
High	P13647	' KRT5	Keratin, type II cytoskeletal 5 OS=Homo sapiens OX=9606 GN=KRT5	0
High	P08729	' KRT7	Keratin, type II cytoskeletal 7 OS=Homo sapiens OX=9606 GN=KRT7	0
High	Q9UKK3	' PARP4	Poly [ADP-ribose] polymerase 4 OS=Homo sapiens OX=9606 GN=PA	0
High	Q5K651	' SAMD9	Sterile alpha motif domain-containing protein 9 OS=Homo sapiens (	0
High	P68371	' TUBB4B	Tubulin beta-4B chain OS=Homo sapiens OX=9606 GN=TUBB4B PE=	0
High	Q13045	' FLII	Protein flightless-1 homolog OS=Homo sapiens OX=9606 GN=FLII PE	0
High	Q9Y2W1	' THRAP3	Thyroid hormone receptor-associated protein 3 OS=Homo sapiens (	0
High	Q04695	' KRT17	Keratin, type I cytoskeletal 17 OS=Homo sapiens OX=9606 GN=KRT1	0
High	O75643	' SNRNP200	U5 small nuclear ribonucleoprotein 200 kDa helicase OS=Homo sapi	0
High	Q92499	' DDX1	ATP-dependent RNA helicase DDX1 OS=Homo sapiens OX=9606 GN=	0
High	Q6P2Q9	' PRPF8	Pre-mRNA-processing-splicing factor 8 OS=Homo sapiens OX=9606 (	0
High	P21980	' TGM2	Protein-glutamine gamma-glutamyltransferase 2 OS=Homo sapiens	0
High	Q13045-2	' FLII	Isoform 2 of Protein flightless-1 homolog OS=Homo sapiens OX=960	0
High	Q9UHB6	' LIMA1	LIM domain and actin-binding protein 1 OS=Homo sapiens OX=9606	0
High	Q6ZRV2	' FAM83H	Protein FAM83H OS=Homo sapiens OX=9606 GN=FAM83H PE=1 SV=	0
High	Q9UHB6-4	' LIMA1	Isoform 4 of LIM domain and actin-binding protein 1 OS=Homo sapi	0
High	P12956	' XRCC6	X-ray repair cross-complementing protein 6 OS=Homo sapiens OX=9	0
High	P04406	' GAPDH	Glyceraldehyde-3-phosphate dehydrogenase OS=Homo sapiens OX=	0
High	P11142	' HSPA8	Heat shock cognate 71 kDa protein OS=Homo sapiens OX=9606 GN=	0
High	Q9UPQ9-	' TNRC6B	Isoform 2 of Trinucleotide repeat-containing gene 6B protein OS=Ho	0
High	O43795-2	' MYO1B	Isoform 2 of Unconventional myosin-Ib OS=Homo sapiens OX=9606	0
High	Q16666	' IFI16	Gamma-interferon-inducible protein 16 OS=Homo sapiens OX=9606	0
High	O15231	' ZNF185	Zinc finger protein 185 OS=Homo sapiens OX=9606 GN=ZNF185 PE=	0
High	P13010	' XRCC5	X-ray repair cross-complementing protein 5 OS=Homo sapiens OX=9	0
High	P02533	' KRT14	Keratin, type I cytoskeletal 14 OS=Homo sapiens OX=9606 GN=KRT1	0
High	P23246	' SFPQ	Splicing factor, proline- and glutamine-rich OS=Homo sapiens OX=96	0
High	Q9Y5B9	' SUPT16H	FACT complex subunit SPT16 OS=Homo sapiens OX=9606 GN=SUPT	0
High	P08238	' HSP90AB1	Heat shock protein HSP 90-beta OS=Homo sapiens OX=9606 GN=HS	0
High	Q9BYK8	' HELZ2	Helicase with zinc finger domain 2 OS=Homo sapiens OX=9606 GN=	0
High	Q9BUJ2	' HNRNPUL1	Heterogeneous nuclear ribonucleoprotein U-like protein 1 OS=Hom	0
High	Q69YQ0	' SPECC1L	Cytospin-A OS=Homo sapiens OX=9606 GN=SPECC1L PE=1 SV=2	0
High	Q14247	' CTTN	Src substrate cortactin OS=Homo sapiens OX=9606 GN=CTTN PE=1 S	0
High	O00571	' DDX3X	ATP-dependent RNA helicase DDX3X OS=Homo sapiens OX=9606 GN	0
High	Q15424-3	' SAFB	Isoform 3 of Scaffold attachment factor B1 OS=Homo sapiens OX=96	0
High	Q02880	' TOP2B	DNA topoisomerase 2-beta OS=Homo sapiens OX=9606 GN=TOP2B	0
High	Q08211	' DHX9	ATP-dependent RNA helicase A OS=Homo sapiens OX=9606 GN=DH	0
High	Q00839	' HNRNPU	Heterogeneous nuclear ribonucleoprotein U OS=Homo sapiens OX=	0
High	P14923	' JUP	Junction plakoglobin OS=Homo sapiens OX=9606 GN=JUP PE=1 SV=	0
High	Q8IY21	' DDX60	Probable ATP-dependent RNA helicase DDX60 OS=Homo sapiens OX	0
High	Q14151	' SAFB2	Scaffold attachment factor B2 OS=Homo sapiens OX=9606 GN=SAFB	0
High	Q14966	' ZNF638	Zinc finger protein 638 OS=Homo sapiens OX=9606 GN=ZNF638 PE=	0
High	P17844	' DDX5	Probable ATP-dependent RNA helicase DDX5 OS=Homo sapiens OX=	0
High	P20700	' LMNB1	Lamin-B1 OS=Homo sapiens OX=9606 GN=LMNB1 PE=1 SV=2	0
High	Q13885	' TUBB2A	Tubulin beta-2A chain OS=Homo sapiens OX=9606 GN=TUBB2A PE=	0
High	P08779	' KRT16	Keratin, type I cytoskeletal 16 OS=Homo sapiens OX=9606 GN=KRT1	0
High	O95171	' SCEL	Sciellin OS=Homo sapiens OX=9606 GN=SCEL PE=1 SV=2	0
High	O14646	' CHD1	Chromodomain-helicase-DNA-binding protein 1 OS=Homo sapiens (	0
High	Q00839-2	' HNRNPU	Isoform 2 of Heterogeneous nuclear ribonucleoprotein U OS=Homo	0
High	P52272	' HNRNPM	Heterogeneous nuclear ribonucleoprotein M OS=Homo sapiens OX=	0
High	Q71U36	' TUBA1A	Tubulin alpha-1A chain OS=Homo sapiens OX=9606 GN=TUBA1A PE	0
High	Q14573	' ITPR3	Inositol 1,4,5-trisphosphate receptor type 3 OS=Homo sapiens OX=9	0
High	Q07157	' TJP1	Tight junction protein ZO-1 OS=Homo sapiens OX=9606 GN=TJP1 PE	0
High	P11021	' HSPA5	Endoplasmic reticulum chaperone BiP OS=Homo sapiens OX=9606 G	0
High	O95171-2	' SCEL	Isoform 2 of Sciellin OS=Homo sapiens OX=9606 GN=SCEL	0
High	Q01813	' PFKP	ATP-dependent 6-phosphofructokinase, platelet type OS=Homo sap	0
High	Q07157-2	' TJP1	Isoform Short of Tight junction protein ZO-1 OS=Homo sapiens OX=	0
High	P02787	' TF	Serotransferrin OS=Homo sapiens OX=9606 GN=TF PE=1 SV=3	0
High	O14647	' CHD2	Chromodomain-helicase-DNA-binding protein 2 OS=Homo sapiens (	0
High	Q99959-2	' PKP2	Isoform 1 of Plakophilin-2 OS=Homo sapiens OX=9606 GN=PKP2	0
High	Q9UPN3-	' MACF1	Isoform 4 of Microtubule-actin cross-linking factor 1, isoforms 1/2/3	0
High	Q92841	' DDX17	Probable ATP-dependent RNA helicase DDX17 OS=Homo sapiens OX	0
High	Q7Z2W4	' ZC3HAV1	Zinc finger CCCH-type antiviral protein 1 OS=Homo sapiens OX=960	0
High	Q9UKV3	' ACIN1	Apoptotic chromatin condensation inducer in the nucleus OS=Homo	0
High	O15523	' DDX3Y	ATP-dependent RNA helicase DDX3Y OS=Homo sapiens OX=9606 GN	0
High	Q9Y4K1	' CRYBG1	Beta/gamma crystallin domain-containing protein 1 OS=Homo sapie	0
High	Q16643	' DBN1	Drebrin OS=Homo sapiens OX=9606 GN=DBN1 PE=1 SV=4	0
High	O75533	' SF3B1	Splicing factor 3B subunit 1 OS=Homo sapiens OX=9606 GN=SF3B1	0
High	Q14157-5	' UBAP2L	Isoform 5 of Ubiquitin-associated protein 2-like OS=Homo sapiens C	0
High	Q86V48	' LUZP1	Leucine zipper protein 1 OS=Homo sapiens OX=9606 GN=LUZP1 PE=	0
High	Q13283	' G3BP1	Ras GTPase-activating protein-binding protein 1 OS=Homo sapiens (	0
High	Q14157-1	' UBAP2L	Isoform 2 of Ubiquitin-associated protein 2-like OS=Homo sapiens C	0
High	Q9BQE3	' TUBA1C	Tubulin alpha-1C chain OS=Homo sapiens OX=9606 GN=TUBA1C PE	0
High	Q9UM54-	' MYO6	Isoform 6 of Unconventional myosin-VI OS=Homo sapiens OX=9606	0
High	P68104	' EEF1A1	Elongation factor 1-alpha 1 OS=Homo sapiens OX=9606 GN=EEF1A1	0
High	P07900-2	' HSP90AA1	Isoform 2 of Heat shock protein HSP 90-alpha OS=Homo sapiens OX	0
High	P38646	' HSPA9	Stress-70 protein, mitochondrial OS=Homo sapiens OX=9606 GN=HS	0
High	O60716-3	' CTNND1	Isoform 1AC of Catenin delta-1 OS=Homo sapiens OX=9606 GN=CTN	0
High	Q9Y2J4-2	' AMOTL2	Isoform 2 of Angiomotin-like protein 2 OS=Homo sapiens OX=9606 (	0
High	P55072	' VCP	Transitional endoplasmic reticulum ATPase OS=Homo sapiens OX=9	0
High	P78371	' CCT2	T-complex protein 1 subunit beta OS=Homo sapiens OX=9606 GN=C	0
High	Q9Y2J4-4	' AMOTL2	Isoform 4 of Angiomotin-like protein 2 OS=Homo sapiens OX=9606 (	0
High	Q14980	' NUMA1	Nuclear mitotic apparatus protein 1 OS=Homo sapiens OX=9606 GN	0
High	P22626	' HNRNPA2B1	Heterogeneous nuclear ribonucleoproteins A2/B1 OS=Homo sapien	0
High	Q8WWI1-	' LMO7	Isoform 3 of LIM domain only protein 7 OS=Homo sapiens OX=9606	0
High	P11387	' TOP1	DNA topoisomerase 1 OS=Homo sapiens OX=9606 GN=TOP1 PE=1 S	0
High	P13639	' EEF2	Elongation factor 2 OS=Homo sapiens OX=9606 GN=EEF2 PE=1 SV=4	0
High	P68032	' ACTC1	Actin, alpha cardiac muscle 1 OS=Homo sapiens OX=9606 GN=ACTC	0
High	P06396-2	' GSN	Isoform 2 of Gelsolin OS=Homo sapiens OX=9606 GN=GSN	0
High	O15020	' SPTBN2	Spectrin beta chain, non-erythrocytic 2 OS=Homo sapiens OX=9606	0
High	Q9BZF9	' UACA	Uveal autoantigen with coiled-coil domains and ankyrin repeats OS=	0
High	P16615	' ATP2A2	Sarcoplasmic/endoplasmic reticulum calcium ATPase 2 OS=Homo sa	0
High	P09651	' HNRNPA1	Heterogeneous nuclear ribonucleoprotein A1 OS=Homo sapiens OX	0
High	Q12906	' ILF3	Interleukin enhancer-binding factor 3 OS=Homo sapiens OX=9606 G	0
High	Q9Y3I0	' RTCB	tRNA-splicing ligase RtcB homolog OS=Homo sapiens OX=9606 GN=	0
High	P49750	' YLPM1	YLP motif-containing protein 1 OS=Homo sapiens OX=9606 GN=YLP	0
High	P07814	' EPRS	Bifunctional glutamate/proline--tRNA ligase OS=Homo sapiens OX=9	0
High	Q03252	' LMNB2	Lamin-B2 OS=Homo sapiens OX=9606 GN=LMNB2 PE=1 SV=4	0
High	P68366	' TUBA4A	Tubulin alpha-4A chain OS=Homo sapiens OX=9606 GN=TUBA4A PE	0
High	Q16555	' DPYSL2	Dihydropyrimidinase-related protein 2 OS=Homo sapiens OX=9606 (	0
High	Q9UKV3-5	' ACIN1	Isoform 4 of Apoptotic chromatin condensation inducer in the nucle	0
High	O15231-2	' ZNF185	Isoform 2 of Zinc finger protein 185 OS=Homo sapiens OX=9606 GN	0
High	P06576	' ATP5F1B	ATP synthase subunit beta, mitochondrial OS=Homo sapiens OX=96	0
High	P0DMV9	' HSPA1B	Heat shock 70 kDa protein 1B OS=Homo sapiens OX=9606 GN=HSPA	0
High	P35221	' CTNNA1	Catenin alpha-1 OS=Homo sapiens OX=9606 GN=CTNNA1 PE=1 SV=	0
High	Q9UIG0	' BAZ1B	Tyrosine-protein kinase BAZ1B OS=Homo sapiens OX=9606 GN=BAZ	0
High	O75367	' H2AFY	Core histone macro-H2A.1 OS=Homo sapiens OX=9606 GN=H2AFY	0
High	Q9NWH9	' SLTM	SAFB-like transcription modulator OS=Homo sapiens OX=9606 GN=	0
High	P68133	' ACTA1	Actin, alpha skeletal muscle OS=Homo sapiens OX=9606 GN=ACTA1	0
High	P02751-1	' FN1	Isoform 15 of Fibronectin OS=Homo sapiens OX=9606 GN=FN1	0
High	Q13509	' TUBB3	Tubulin beta-3 chain OS=Homo sapiens OX=9606 GN=TUBB3 PE=1 S	0
High	P11940	' PABPC1	Polyadenylate-binding protein 1 OS=Homo sapiens OX=9606 GN=PA	0
High	Q15233	' NONO	Non-POU domain-containing octamer-binding protein OS=Homo sa	0
High	Q12906-7	' ILF3	Isoform 7 of Interleukin enhancer-binding factor 3 OS=Homo sapien	0
High	P35637	' FUS	RNA-binding protein FUS OS=Homo sapiens OX=9606 GN=FUS PE=1	0
High	P39023	' RPL3	60S ribosomal protein L3 OS=Homo sapiens OX=9606 GN=RPL3 PE=	0
High	Q9Y446-2	' PKP3	Isoform PKP3b of Plakophilin-3 OS=Homo sapiens OX=9606 GN=PKP	0
High	P06733	' ENO1	Alpha-enolase OS=Homo sapiens OX=9606 GN=ENO1 PE=1 SV=2	0
High	Q9UQB8	' BAIAP2	Brain-specific angiogenesis inhibitor 1-associated protein 2 OS=Hom	0
High	O60264	' SMARCA5	SWI/SNF-related matrix-associated actin-dependent regulator of ch	0
High	P20591	' MX1	Interferon-induced GTP-binding protein Mx1 OS=Homo sapiens OX=	0
High	O15021	' MAST4	Microtubule-associated serine/threonine-protein kinase 4 OS=Hom	0
High	P23229-6	' ITGA6	Isoform Alpha-6X1X2A of Integrin alpha-6 OS=Homo sapiens OX=96	0
High	Q9UQB8-	' BAIAP2	Isoform 5 of Brain-specific angiogenesis inhibitor 1-associated prote	0
High	Q9NYL9	' TMOD3	Tropomodulin-3 OS=Homo sapiens OX=9606 GN=TMOD3 PE=1 SV=1	0
High	Q03001	' DST	Dystonin OS=Homo sapiens OX=9606 GN=DST PE=1 SV=4	0
High	P31943	' HNRNPH1	Heterogeneous nuclear ribonucleoprotein H OS=Homo sapiens OX=	0
High	Q07065	' CKAP4	Cytoskeleton-associated protein 4 OS=Homo sapiens OX=9606 GN=	0
High	Q86YZ3	' HRNR	Hornerin OS=Homo sapiens OX=9606 GN=HRNR PE=1 SV=2	0
High	Q13435	' SF3B2	Splicing factor 3B subunit 2 OS=Homo sapiens OX=9606 GN=SF3B2	0
High	P19474	' TRIM21	E3 ubiquitin-protein ligase TRIM21 OS=Homo sapiens OX=9606 GN=	0
High	O00515	' LAD1	Ladinin-1 OS=Homo sapiens OX=9606 GN=LAD1 PE=1 SV=2	0
High	Q9UQB8-	' BAIAP2	Isoform 6 of Brain-specific angiogenesis inhibitor 1-associated prote	0
High	O94901-9	' SUN1	Isoform 9 of SUN domain-containing protein 1 OS=Homo sapiens OX	0
High	P49368	' CCT3	T-complex protein 1 subunit gamma OS=Homo sapiens OX=9606 GN	0
High	Q12965	' MYO1E	Unconventional myosin-Ie OS=Homo sapiens OX=9606 GN=MYO1E	0
High	P42704	' LRPPRC	Leucine-rich PPR motif-containing protein, mitochondrial OS=Homo	0
High	Q6NYC8	' PPP1R18	Phostensin OS=Homo sapiens OX=9606 GN=PPP1R18 PE=1 SV=1	0
High	P58107	' EPPK1	Epiplakin OS=Homo sapiens OX=9606 GN=EPPK1 PE=1 SV=3	0
High	P22314	' UBA1	Ubiquitin-like modifier-activating enzyme 1 OS=Homo sapiens OX=9	0
High	P36578	' RPL4	60S ribosomal protein L4 OS=Homo sapiens OX=9606 GN=RPL4 PE=	0
High	Q15582	' TGFBI	Transforming growth factor-beta-induced protein ig-h3 OS=Homo s	0
High	Q92734	' TFG	Protein TFG OS=Homo sapiens OX=9606 GN=TFG PE=1 SV=2	0
High	P31942	' HNRNPH3	Heterogeneous nuclear ribonucleoprotein H3 OS=Homo sapiens OX	0
High	P20592	' MX2	Interferon-induced GTP-binding protein Mx2 OS=Homo sapiens OX=	0
High	Q8WYP5-	' AHCTF1	Isoform 2 of Protein ELYS OS=Homo sapiens OX=9606 GN=AHCTF1	0
High	Q7KZF4	' SND1	Staphylococcal nuclease domain-containing protein 1 OS=Homo sap	0
High	Q8WWM	' ATXN2L	Ataxin-2-like protein OS=Homo sapiens OX=9606 GN=ATXN2L PE=1	0
High	P55265-4	' ADAR	Isoform 4 of Double-stranded RNA-specific adenosine deaminase O	0
High	P17987	' TCP1	T-complex protein 1 subunit alpha OS=Homo sapiens OX=9606 GN=	0
High	Q16891	' IMMT	MICOS complex subunit MIC60 OS=Homo sapiens OX=9606 GN=IMM	0
High	Q9NR30	' DDX21	Nucleolar RNA helicase 2 OS=Homo sapiens OX=9606 GN=DDX21 PE	0
High	P60842	' EIF4A1	Eukaryotic initiation factor 4A-I OS=Homo sapiens OX=9606 GN=EIF	0
High	Q92804	' TAF15	TATA-binding protein-associated factor 2N OS=Homo sapiens OX=96	0
High	Q9NZB2-6	' FAM120A	Isoform F of Constitutive coactivator of PPAR-gamma-like protein 1	0
High	Q9Y490	' TLN1	Talin-1 OS=Homo sapiens OX=9606 GN=TLN1 PE=1 SV=3	0
High	Q14444	' CAPRIN1	Caprin-1 OS=Homo sapiens OX=9606 GN=CAPRIN1 PE=1 SV=2	0
High	Q8IVT2	' MISP	Mitotic interactor and substrate of PLK1 OS=Homo sapiens OX=9606	0
High	Q99590	' SCAF11	Protein SCAF11 OS=Homo sapiens OX=9606 GN=SCAF11 PE=1 SV=2	0
High	P11216	' PYGB	Glycogen phosphorylase, brain form OS=Homo sapiens OX=9606 GN	0
High	Q16891-4	' IMMT	Isoform 4 of MICOS complex subunit MIC60 OS=Homo sapiens OX=9	0
High	P06748	' NPM1	Nucleophosmin OS=Homo sapiens OX=9606 GN=NPM1 PE=1 SV=2	0
High	Q9NR12	' PDLIM7	PDZ and LIM domain protein 7 OS=Homo sapiens OX=9606 GN=PDL	0
High	Q9Y608	' LRRFIP2	Leucine-rich repeat flightless-interacting protein 2 OS=Homo sapien	0
High	Q8N3C0	' ASCC3	Activating signal cointegrator 1 complex subunit 3 OS=Homo sapien	0
High	O60488	' ACSL4	Long-chain-fatty-acid--CoA ligase 4 OS=Homo sapiens OX=9606 GN=	0
High	Q96PK6	' RBM14	RNA-binding protein 14 OS=Homo sapiens OX=9606 GN=RBM14 PE=	0
High	P60660-2	' MYL6	Isoform Smooth muscle of Myosin light polypeptide 6 OS=Homo sap	0
High	P43243	' MATR3	Matrin-3 OS=Homo sapiens OX=9606 GN=MATR3 PE=1 SV=2	0
High	P08670	' VIM	Vimentin OS=Homo sapiens OX=9606 GN=VIM PE=1 SV=4	0
High	P50990	' CCT8	T-complex protein 1 subunit theta OS=Homo sapiens OX=9606 GN=	0
High	P19338	' NCL	Nucleolin OS=Homo sapiens OX=9606 GN=NCL PE=1 SV=3	0
High	P25705	' ATP5F1A	ATP synthase subunit alpha, mitochondrial OS=Homo sapiens OX=96	0
High	Q99973	' TEP1	Telomerase protein component 1 OS=Homo sapiens OX=9606 GN=T	0
High	O14639	' ABLIM1	Actin-binding LIM protein 1 OS=Homo sapiens OX=9606 GN=ABLIM	0
High	O43143	' DHX15	Pre-mRNA-splicing factor ATP-dependent RNA helicase DHX15 OS=H	0
High	Q9P2J5	' LARS	Leucine--tRNA ligase, cytoplasmic OS=Homo sapiens OX=9606 GN=L	0
High	P07355-2	' ANXA2	Isoform 2 of Annexin A2 OS=Homo sapiens OX=9606 GN=ANXA2	0
High	O95573	' ACSL3	Long-chain-fatty-acid--CoA ligase 3 OS=Homo sapiens OX=9606 GN=	0
High	P61978-2	' HNRNPK	Isoform 2 of Heterogeneous nuclear ribonucleoprotein K OS=Homo	0
High	Q14676	' MDC1	Mediator of DNA damage checkpoint protein 1 OS=Homo sapiens O	0
High	Q9BUF5	' TUBB6	Tubulin beta-6 chain OS=Homo sapiens OX=9606 GN=TUBB6 PE=1 S	0
High	P19367-3	' HK1	Isoform 3 of Hexokinase-1 OS=Homo sapiens OX=9606 GN=HK1	0
High	P55265-2	' ADAR	Isoform 2 of Double-stranded RNA-specific adenosine deaminase O	0
High	P49411	' TUFM	Elongation factor Tu, mitochondrial OS=Homo sapiens OX=9606 GN	0
High	Q15029	' EFTUD2	116 kDa U5 small nuclear ribonucleoprotein component OS=Homo	0
High	Q14498-2	' RBM39	Isoform 2 of RNA-binding protein 39 OS=Homo sapiens OX=9606 GN	0
High	P35222	' CTNNB1	Catenin beta-1 OS=Homo sapiens OX=9606 GN=CTNNB1 PE=1 SV=1	0
High	P17858	' PFKL	ATP-dependent 6-phosphofructokinase, liver type OS=Homo sapien	0
High	P31942-2	' HNRNPH3	Isoform 2 of Heterogeneous nuclear ribonucleoprotein H3 OS=Hom	0
High	P14625	' HSP90B1	Endoplasmin OS=Homo sapiens OX=9606 GN=HSP90B1 PE=1 SV=1	0
High	Q08J23	' NSUN2	tRNA (cytosine(34)-C(5))-methyltransferase OS=Homo sapiens OX=9	0
High	Q8WWQ0	' PHIP	PH-interacting protein OS=Homo sapiens OX=9606 GN=PHIP PE=1 S	0
High	Q9P0K7-2	' RAI14	Isoform 2 of Ankycorbin OS=Homo sapiens OX=9606 GN=RAI14	0
High	Q14126	' DSG2	Desmoglein-2 OS=Homo sapiens OX=9606 GN=DSG2 PE=1 SV=2	0
High	P21589	' NT5E	5'-nucleotidase OS=Homo sapiens OX=9606 GN=NT5E PE=1 SV=1	0
High	Q15393	' SF3B3	Splicing factor 3B subunit 3 OS=Homo sapiens OX=9606 GN=SF3B3	0
High	P41252	' IARS	Isoleucine--tRNA ligase, cytoplasmic OS=Homo sapiens OX=9606 GN	0
High	Q9NYL2	' MAP3K20	Mitogen-activated protein kinase kinase kinase 20 OS=Homo sapien	0
High	Q9UJV9	' DDX41	Probable ATP-dependent RNA helicase DDX41 OS=Homo sapiens OX	0
High	Q9NQH7	' XPNPEP3	Xaa-Pro aminopeptidase 3 OS=Homo sapiens OX=9606 GN=XPNPEP	0
High	P00558	' PGK1	Phosphoglycerate kinase 1 OS=Homo sapiens OX=9606 GN=PGK1 PE	0
High	P61978-3	' HNRNPK	Isoform 3 of Heterogeneous nuclear ribonucleoprotein K OS=Homo	0
High	Q7L2E3-2	' DHX30	Isoform 2 of Putative ATP-dependent RNA helicase DHX30 OS=Hom	0
High	O00159-3	' MYO1C	Isoform 3 of Unconventional myosin-Ic OS=Homo sapiens OX=9606	0
High	P62424	' RPL7A	60S ribosomal protein L7a OS=Homo sapiens OX=9606 GN=RPL7A P	0
High	O43390	' HNRNPR	Heterogeneous nuclear ribonucleoprotein R OS=Homo sapiens OX=	0
High	Q8IYB3	' SRRM1	Serine/arginine repetitive matrix protein 1 OS=Homo sapiens OX=96	0
High	P53618	' COPB1	Coatomer subunit beta OS=Homo sapiens OX=9606 GN=COPB1 PE=	0
High	Q99832	' CCT7	T-complex protein 1 subunit eta OS=Homo sapiens OX=9606 GN=CC	0
High	P60660	' MYL6	Myosin light polypeptide 6 OS=Homo sapiens OX=9606 GN=MYL6 P	0
High	P48643	' CCT5	T-complex protein 1 subunit epsilon OS=Homo sapiens OX=9606 GN	0
High	P43490	' NAMPT	Nicotinamide phosphoribosyltransferase OS=Homo sapiens OX=960	0
High	A1L390	' PLEKHG3	Pleckstrin homology domain-containing family G member 3 OS=Hon	0
High	P50991	' CCT4	T-complex protein 1 subunit delta OS=Homo sapiens OX=9606 GN=	0
High	Q8WUF5	' PPP1R13L	RelA-associated inhibitor OS=Homo sapiens OX=9606 GN=PPP1R13L	0
High	P13646	' KRT13	Keratin, type I cytoskeletal 13 OS=Homo sapiens OX=9606 GN=KRT1	0
High	P40939	' HADHA	Trifunctional enzyme subunit alpha, mitochondrial OS=Homo sapier	0
High	P27708	' CAD	CAD protein OS=Homo sapiens OX=9606 GN=CAD PE=1 SV=3	0
High	P61158	' ACTR3	Actin-related protein 3 OS=Homo sapiens OX=9606 GN=ACTR3 PE=1	0
High	O43776	' NARS	Asparagine--tRNA ligase, cytoplasmic OS=Homo sapiens OX=9606 G	0
High	Q8NCA5	' FAM98A	Protein FAM98A OS=Homo sapiens OX=9606 GN=FAM98A PE=1 SV=	0
High	Q05639	' EEF1A2	Elongation factor 1-alpha 2 OS=Homo sapiens OX=9606 GN=EEF1A2	0
High	P10809	' HSPD1	60 kDa heat shock protein, mitochondrial OS=Homo sapiens OX=960	0
High	Q13310-3	' PABPC4	Isoform 3 of Polyadenylate-binding protein 4 OS=Homo sapiens OX=	0

High	Q5M775	' SPECC1	Cytospin-B OS=Homo sapiens OX=9606 GN=SPECC1 PE=1 SV=1	0	122.833	37.07865	34	118	34	1068	118.5	6.7		2.566
High	Q15365	' PCBP1	Poly(rC)-binding protein 1 OS=Homo sapiens OX=9606 GN=PCBP1 P	0	121.991	74.1573	17	117	12	356	37.5	7.09		25.827
High	O14746	' TERT	Telomerase reverse transcriptase OS=Homo sapiens OX=9606 GN=T	0	121.67	35.60071	30	90	30	1132	126.9	10.52		1.916
High	O60506	' SYNCRIP	Heterogeneous nuclear ribonucleoprotein Q OS=Homo sapiens OX=	0	121.545	54.57464	28	122	21	623	69.6	8.59		5.31
High	Q08380	' LGALS3BP	Galectin-3-binding protein OS=Homo sapiens OX=9606 GN=LGALS3E	0	121.379	40.68376	21	134	21	585	65.3	5.27		6.055
High	P35606	' COPB2	Coatomer subunit beta' OS=Homo sapiens OX=9606 GN=COPB2 PE=	0	121.363	48.45475	30	99	30	906	102.4	5.27		3.047
High	Q9Y2D5-6	' AKAP2	Isoform 4 of A-kinase anchor protein 2 OS=Homo sapiens OX=9606	0	121.323	33.11927	25	86	25	1090	120.6	5.05	Phospho	2.884
High	Q9UHD8	' SEPT9	Septin-9 OS=Homo sapiens OX=9606 GN=SEPT9 PE=1 SV=2	0	121.089	60.75085	28	138	28	586	65.4	8.97		6.943
High	Q9NUQ6-	' SPATS2L	Isoform 3 of SPATS2-like protein OS=Homo sapiens OX=9606 GN=SP	0	120.643	48.63946	24	88	2	588	65.1	9.66		3.819
High	Q86VP6	' CAND1	Cullin-associated NEDD8-dissociated protein 1 OS=Homo sapiens O	0	120.486	32.19512	31	79	31	1230	136.3	5.78		1.783
High	P08237-3	' PFKM	Isoform 3 of ATP-dependent 6-phosphofructokinase, muscle type O	0	120.375	43.24324	31	107	28	851	93.2	7.97		3.715
High	P62805	' HIST1H4A	Histone H4 OS=Homo sapiens OX=9606 GN=HIST1H4A PE=1 SV=2	0	120.098	67.96117	18	307	18	103	11.4	11.36		99999
High	Q69YN4	' VIRMA	Protein virilizer homolog OS=Homo sapiens OX=9606 GN=VIRMA PE	0	120.071	23.34437	31	87	31	1812	201.9	5.01	Phospho	0.966
High	Q92900	' UPF1	Regulator of nonsense transcripts 1 OS=Homo sapiens OX=9606 GN	0	119.247	30.82374	30	87	30	1129	124.3	6.61		2.406
High	Q7Z417	' NUFIP2	Nuclear fragile X mental retardation-interacting protein 2 OS=Homo	0	119.133	51.51079	26	137	26	695	76.1	8.7		9
High	O14950	' MYL12B	Myosin regulatory light chain 12B OS=Homo sapiens OX=9606 GN=M	0	119.108	80.23256	13	195	6	172	19.8	4.84		186.382
High	Q92616	' GCN1	eIF-2-alpha kinase activator GCN1 OS=Homo sapiens OX=9606 GN=	0	118.931	19.54324	39	73	39	2671	292.6	7.47		0.661
High	Q7Z2W4-	' ZC3HAV1	Isoform 3 of Zinc finger CCCH-type antiviral protein 1 OS=Homo sap	0	118.8	52.08333	25	143	1	624	67.5	8.72		4.337
High	Q14669-2	' TRIP12	Isoform 2 of E3 ubiquitin-protein ligase TRIP12 OS=Homo sapiens O	0	118.791	22.02469	32	66	32	2025	223.8	8.4		0.82
High	A0MZ66-	' SHTN1	Isoform 3 of Shootin-1 OS=Homo sapiens OX=9606 GN=SHTN1	0	118.733	54.69954	32	95	32	649	73.6	5.45		5.579
High	O15144	' ARPC2	Actin-related protein 2/3 complex subunit 2 OS=Homo sapiens OX=	0	118.612	71	21	115	21	300	34.3	7.36		19.153
High	O14639-5	' ABLIM1	Isoform 5 of Actin-binding LIM protein 1 OS=Homo sapiens OX=960	0	118.429	68.79121	24	134	1	455	52	9.19	Phospho	11.69
High	Q9UN86-	' G3BP2	Isoform B of Ras GTPase-activating protein-binding protein 2 OS=Ho	0	117.614	59.02004	20	150	1	449	50.8	5.38	Phospho	13.065
High	Q08945	' SSRP1	FACT complex subunit SSRP1 OS=Homo sapiens OX=9606 GN=SSRP1	0	116.397	40.47955	23	80	23	709	81	6.87	Phospho	4.957
High	Q86SQ0	' PHLDB2	Pleckstrin homology-like domain family B member 2 OS=Homo sapi	0	115.911	34.79649	39	102	39	1253	142.1	7.43	Phospho	2.36
High	P06753-2	' TPM3	Isoform 2 of Tropomyosin alpha-3 chain OS=Homo sapiens OX=9606	0	115.727	68.54839	30	142	5	248	29	4.78		56.797
High	P14866	' HNRNPL	Heterogeneous nuclear ribonucleoprotein L OS=Homo sapiens OX=	0	115.603	51.44312	21	152	20	589	64.1	8.22		9
High	Q01844-5	' EWSR1	Isoform 5 of RNA-binding protein EWS OS=Homo sapiens OX=9606	0	115.569	22.84418	15	184	15	661	68.9	9.29		11.217
High	O00468-6	' AGRN	Isoform 6 of Agrin OS=Homo sapiens OX=9606 GN=AGRN	0	115.189	25.47677	33	75	33	2045	214.7	6.4		1.186
High	Q9UQE7	' SMC3	Structural maintenance of chromosomes protein 3 OS=Homo sapier	0	115.131	34.34675	33	90	33	1217	141.5	7.18		1.456
High	P62701	' RPS4X	40S ribosomal protein S4, X isoform OS=Homo sapiens OX=9606 GN	0	114.717	56.65399	19	263	11	263	29.6	10.15		24.119
High	P00533	' EGFR	Epidermal growth factor receptor OS=Homo sapiens OX=9606 GN=E	0	114.476	35.12397	28	85	28	1210	134.2	6.68		1.916
High	P40227	' CCT6A	T-complex protein 1 subunit zeta OS=Homo sapiens OX=9606 GN=C	0	114.248	51.22411	22	126	22	531	58	6.68		6.43
High	O15195	' VILL	Villin-like protein OS=Homo sapiens OX=9606 GN=VILL PE=2 SV=3	0	114.1	32.00935	25	68	25	856	95.8	7.02		2.444
High	Q9Y678	' COPG1	Coatomer subunit gamma-1 OS=Homo sapiens OX=9606 GN=COPG1	0	113.701	43.59268	27	95	23	874	97.7	5.47		2.612
High	Q99459	' CDC5L	Cell division cycle 5-like protein OS=Homo sapiens OX=9606 GN=CD	0	113.657	42.5187	25	87	25	802	92.2	8.18		3.885
High	Q9HCD5	' NCOA5	Nuclear receptor coactivator 5 OS=Homo sapiens OX=9606 GN=NCO	0	113.378	59.06736	21	135	21	579	65.5	9.6		9
High	P38159	' RBMX	RNA-binding motif protein, X chromosome OS=Homo sapiens OX=96	0	113.108	47.05882	22	288	8	391	42.3	10.05	Phospho	10.498
High	P54136	' RARS	Arginine--tRNA ligase, cytoplasmic OS=Homo sapiens OX=9606 GN=	0	113.033	51.06061	32	112	32	660	75.3	6.68		4.549
High	O94929	' ABLIM3	Actin-binding LIM protein 3 OS=Homo sapiens OX=9606 GN=ABLIM3	0	112.886	47.58419	25	92	3	683	77.8	8.54	Phospho	3.348
High	P13797	' PLS3	Plastin-3 OS=Homo sapiens OX=9606 GN=PLS3 PE=1 SV=4	0	112.802	50.15873	25	80	19	630	70.8	5.6		3.903
High	P05023	' ATP1A1	Sodium/potassium-transporting ATPase subunit alpha-1 OS=Homo s	0	112.532	33.52884	27	125	27	1023	112.8	5.49		2.455
High	P11586	' MTHFD1	C-1-tetrahydrofolate synthase, cytoplasmic OS=Homo sapiens OX=9	0	112.433	37.96791	29	114	28	935	101.5	7.3		2.415
High	Q9Y6N5	' SQOR	Sulfide:quinone oxidoreductase, mitochondrial OS=Homo sapiens O	0	112.298	61.55556	23	102	23	450	49.9	9.11		4.817
High	Q9NUQ6-	' SPATS2L	Isoform 2 of SPATS2-like protein OS=Homo sapiens OX=9606 GN=SP	0	112.258	53.57873	23	85	1	489	54.1	9.63		4.908
High	P33993	' MCM7	DNA replication licensing factor MCM7 OS=Homo sapiens OX=9606	0	112.251	43.81085	24	100	24	719	81.3	6.46		2.907
High	Q9Y6K5	' OAS3	2'-5'-oligoadenylate synthase 3 OS=Homo sapiens OX=9606 GN=OAS	0	112.216	33.48666	31	97	31	1087	121.1	8.4		2.769
High	Q86UX7	' FERMT3	Fermitin family homolog 3 OS=Homo sapiens OX=9606 GN=FERMT3	0	112.061	56.07196	26	85	26	667	75.9	6.98		3.732
High	O43166	' SIPA1L1	Signal-induced proliferation-associated 1-like protein 1 OS=Homo sa	0	112.04	27.49446	35	73	35	1804	199.9	8.19		1.056
High	P42167	' TMPO	Lamina-associated polypeptide 2, isoforms beta/gamma OS=Homo s	0	111.819	55.94714	18	104	10	454	50.6	9.38		8.152
High	P28799	' GRN	Granulins OS=Homo sapiens OX=9606 GN=GRN PE=1 SV=2	0	111.612	49.57841	20	110	20	593	63.5	6.83		9.857
High	Q9Y3Z3	' SAMHD1	Deoxynucleoside triphosphate triphosphohydrolase SAMHD1 OS=H	0	111.511	49.52077	26	62	26	626	72.2	7.14	Phospho	4.391
High	Q92620	' DHX38	Pre-mRNA-splicing factor ATP-dependent RNA helicase PRP16 OS=H	0	111.49	27.13936	26	69	26	1227	140.4	6.54		1.48
High	P09914	' IFIT1	Interferon-induced protein with tetratricopeptide repeats 1 OS=Hor	0	111.217	59.20502	24	71	24	478	55.3	7.2		5.579
High	P49916	' LIG3	DNA ligase 3 OS=Homo sapiens OX=9606 GN=LIG3 PE=1 SV=2	0	111.187	33.20119	30	75	30	1009	112.8	9.01		2.162
High	P67936-2	' TPM4	Isoform 2 of Tropomyosin alpha-4 chain OS=Homo sapiens OX=9606	0	110.83	61.61972	25	102	6	284	32.7	4.73		17.233
High	P24821	' TNC	Tenascin OS=Homo sapiens OX=9606 GN=TNC PE=1 SV=3	0	110.811	20.53612	33	70	33	2201	240.7	4.89		0.925
High	O00151	' PDLIM1	PDZ and LIM domain protein 1 OS=Homo sapiens OX=9606 GN=PDL	0	110.759	69.60486	20	101	20	329	36	7.02		24.119
High	P42166	' TMPO	Lamina-associated polypeptide 2, isoform alpha OS=Homo sapiens	0	110.446	43.08357	19	99	11	694	75.4	7.66		2.758
High	P51991	' HNRNPA3	Heterogeneous nuclear ribonucleoprotein A3 OS=Homo sapiens OX	0	110.099	44.70899	20	119	19	378	39.6	9.01	Phospho	18.953
High	Q9UN86	' G3BP2	Ras GTPase-activating protein-binding protein 2 OS=Homo sapiens	0	109.756	52.90456	20	96	1	482	54.1	5.55		8.261
High	Q9UDY2-	' TJP2	Isoform 7 of Tight junction protein ZO-2 OS=Homo sapiens OX=9606	0	108.979	28.99263	30	85	29	1221	137.3	8.05		1.339
High	O14879	' IFIT3	Interferon-induced protein with tetratricopeptide repeats 3 OS=Hor	0	108.636	58.97959	19	74	19	490	56	5.2		5.404
High	O94929-3	' ABLIM3	Isoform 3 of Actin-binding LIM protein 3 OS=Homo sapiens OX=960	0	107.967	54.2517	24	83	1	588	67	8.66	Phospho	4.309
High	P55795	' HNRNPH2	Heterogeneous nuclear ribonucleoprotein H2 OS=Homo sapiens OX	0	107.567	49.66592	17	141	9	449	49.2	6.3		9
High	P38919	' EIF4A3	Eukaryotic initiation factor 4A-III OS=Homo sapiens OX=9606 GN=EI	0	107.565	49.63504	22	97	20	411	46.8	6.73		9.965
High	O95793	' STAU1	Double-stranded RNA-binding protein Staufen homolog 1 OS=Homo	0	107.486	54.76603	23	94	21	577	63.1	9.44		5.225
High	Q02878	' RPL6	60S ribosomal protein L6 OS=Homo sapiens OX=9606 GN=RPL6 PE=	0	107.274	53.125	20	165	20	288	32.7	10.58		22.357
High	O43852-3	' CALU	Isoform 3 of Calumenin OS=Homo sapiens OX=9606 GN=CALU	0	106.842	66.56347	19	99	19	323	38	4.63	Phospho	23.484
High	Q9BZE4	' GTPBP4	Nucleolar GTP-binding protein 1 OS=Homo sapiens OX=9606 GN=GT	0	106.754	48.26498	26	61	26	634	73.9	9.5		3.394
High	Q86WJ1	' CHD1L	Chromodomain-helicase-DNA-binding protein 1-like OS=Homo sapie	0	106.454	36.45485	26	87	26	897	100.9	6.9		2.099
High	Q14258	' TRIM25	E3 ubiquitin/ISG15 ligase TRIM25 OS=Homo sapiens OX=9606 GN=T	0	106.345	45.2381	25	120	25	630	70.9	8.09		4.623
High	P35251	' RFC1	Replication factor C subunit 1 OS=Homo sapiens OX=9606 GN=RFC1	0	106.153	31.18467	30	78	30	1148	128.2	9.36		1.949
High	P49756	' RBM25	RNA-binding protein 25 OS=Homo sapiens OX=9606 GN=RBM25 PE=	0	106.078	36.89205	25	127	25	843	100.1	6.32	Phospho	7.733
High	P78344	' EIF4G2	Eukaryotic translation initiation factor 4 gamma 2 OS=Homo sapiens	0	105.989	37.70673	28	122	28	907	102.3	7.14		2.562
High	P52907	' CAPZA1	F-actin-capping protein subunit alpha-1 OS=Homo sapiens OX=9606	0	105.654	72.02797	15	136	13	286	32.9	5.69		19.535
High	Q9UMS4	' PRPF19	Pre-mRNA-processing factor 19 OS=Homo sapiens OX=9606 GN=PRI	0	105.33	64.0873	18	102	18	504	55.1	6.61		12.503
High	Q9UG63-	' ABCF2	Isoform 2 of ATP-binding cassette sub-family F member 2 OS=Homo	0	105.224	36.2776	22	96	22	634	72.4	7.49		4.796
High	P04844	' RPN2	Dolichyl-diphosphooligosaccharide--protein glycosyltransferase sub	0	104.759	43.10618	16	84	16	631	69.2	5.69		3.758
High	P04843	' RPN1	Dolichyl-diphosphooligosaccharide--protein glycosyltransferase sub	0	104.551	53.70675	25	103	25	607	68.5	6.38		3.556
High	Q96HC4	' PDLIM5	PDZ and LIM domain protein 5 OS=Homo sapiens OX=9606 GN=PDL	0	104.225	48.15436	20	101	20	596	63.9	8.21		4.722
High	Q14966-6	' ZNF638	Isoform 6 of Zinc finger protein 638 OS=Homo sapiens OX=9606 GN	0	103.802	37.14597	26	76	1	918	104	9.63		2.444
High	O94929-2	' ABLIM3	Isoform 2 of Actin-binding LIM protein 3 OS=Homo sapiens OX=960	0	103.631	56.98529	23	82	1	544	61.5	8.48	Phospho	4.456
High	P06753-3	' TPM3	Isoform 3 of Tropomyosin alpha-3 chain OS=Homo sapiens OX=9606	0	103.07	58.2996	26	125	1	247	28.9	4.81		27.48
High	P36871	' PGM1	Phosphoglucomutase-1 OS=Homo sapiens OX=9606 GN=PGM1 PE=	0	102.867	52.4911	19	74	19	562	61.4	6.76		3.281
High	P63010-2	' AP2B1	Isoform 2 of AP-2 complex subunit beta OS=Homo sapiens OX=9606	0	102.842	37.32913	24	98	16	951	105.6	5.34		2.802
High	P04792	' HSPB1	Heat shock protein beta-1 OS=Homo sapiens OX=9606 GN=HSPB1 P	0	102.794	84.87805	15	127	15	205	22.8	6.4	Phospho	57.78
High	P26358-2	' DNMT1	Isoform 2 of DNA (cytosine-5)-methyltransferase 1 OS=Homo sapier	0	102.504	24.14216	31	92	31	1632	184.7	7.85		1.172
High	P61247	' RPS3A	40S ribosomal protein S3a OS=Homo sapiens OX=9606 GN=RPS3A P	0	102.49	67.80303	24	182	24	264	29.9	9.73		41.813
High	P33176	' KIF5B	Kinesin-1 heavy chain OS=Homo sapiens OX=9606 GN=KIF5B PE=1 S	0	102.481	33.54102	25	87	25	963	109.6	6.51		1.738
High	P27824-2	' CANX	Isoform 2 of Calnexin OS=Homo sapiens OX=9606 GN=CANX	0	102.464	35.56619	24	107	24	627	71.5	4.7		7.003
High	Q96E39	' RBMXL1	RNA binding motif protein, X-linked-like-1 OS=Homo sapiens OX=96	0	102.103	44.87179	20	228	6	390	42.1	9.89		6.686
High	Q9Y520-7	' PRRC2C	Isoform 7 of Protein PRRC2C OS=Homo sapiens OX=9606 GN=PRRC2	0	101.964	15.49344	31	80	30	2898	316.9	9.13		0.802
High	P18124	' RPL7	60S ribosomal protein L7 OS=Homo sapiens OX=9606 GN=RPL7 PE=	0	100.85	54.03226	20	154	20	248	29.2	10.65		99
High	Q1KMD3	' HNRNPUL2	Heterogeneous nuclear ribonucleoprotein U-like protein 2 OS=Hom	0	100.837	41.90094	28	99	28	747	85.1	4.91	Phospho	4.179
High	P61160	' ACTR2	Actin-related protein 2 OS=Homo sapiens OX=9606 GN=ACTR2 PE=1	0	100.761	48.22335	13	113	13	394	44.7	6.74		10.103
High	Q15654	' TRIP6	Thyroid receptor-interacting protein 6 OS=Homo sapiens OX=9606	0	100.719	57.35294	16	82	16	476	50.3	7.37		4.179
High	P15880	' RPS2	40S ribosomal protein S2 OS=Homo sapiens OX=9606 GN=RPS2 PE=	0	100.643	50.17065	14	200	14	293	31.3	10.24		9
High	P42224	' STAT1	Signal transducer and activator of transcription 1-alpha/beta OS=Ho	0	100.527	35.46667	21	66	21	750	87.3	6.05		2.162
High	P62917	' RPL8	60S ribosomal protein L8 OS=Homo sapiens OX=9606 GN=RPL8 PE=	0	100.457	63.81323	15	124	15	257	28	11.03		42.94
High	Q5T200	' ZC3H13	Zinc finger CCCH domain-containing protein 13 OS=Homo sapiens O	0	100.249	18.28537	31	71	31	1668	196.5	9.42		1.456
High	Q9BY44	' EIF2A	Eukaryotic translation initiation factor 2A OS=Homo sapiens OX=960	0	99.423	54.18803	22	92	22	585	64.9	8.87		3.739
High	P41091	' EIF2S3	Eukaryotic translation initiation factor 2 subunit 3 OS=Homo sapiens	0	99.325	48.51695	18	102	18	472	51.1	8.4		9
High	P11413-2	' G6PD	Isoform Long of Glucose-6-phosphate 1-dehydrogenase OS=Homo s	0	99.173	57.041	28	92	28	561	63.8	6.89		4.623
High	P36952	' SERPINB5	Serpin B5 OS=Homo sapiens OX=9606 GN=SERPINB5 PE=1 SV=2	0	99.138	62.13333	20	101	20	375	42.1	6.05		11.328
High	Q15293	' RCN1	Reticulocalbin-1 OS=Homo sapiens OX=9606 GN=RCN1 PE=1 SV=1	0	99.048	66.16314	19	93	19	331	38.9	5		47.697
High	P47756-2	' CAPZB	Isoform 2 of F-actin-capping protein subunit beta OS=Homo sapiens	0	98.004	69.48529	20	121	20	272	30.6	6		28.764
High	Q15366-3	' PCBP2	Isoform 3 of Poly(rC)-binding protein 2 OS=Homo sapiens OX=9606	0	97.963	60.49724	15	105	1	362	38.2	6.79	Phospho	18.953
High	P26368	' U2AF2	Splicing factor U2AF 65 kDa subunit OS=Homo sapiens OX=9606 GN	0	97.932	43.15789	13	89	13	475	53.5	9.09		7.031
High	Q9H0A0	' NAT10	RNA cytidine acetyltransferase OS=Homo sapiens OX=9606 GN=NAT	0	97.891	29.85366	24	47	24	1025	115.7	8.27		1.668
High	P52597	' HNRNPF	Heterogeneous nuclear ribonucleoprotein F OS=Homo sapiens OX=	0	97.853	51.80723	14	100	12	415	45.6	5.58		8.047
High	P67936	' TPM4	Tropomyosin alpha-4 chain OS=Homo sapiens OX=9606 GN=TPM4 F	0	97.752	56.45161	21	97	4	248	28.5	4.69		16.302
High	Q6KB66	' KRT80	Keratin, type II cytoskeletal 80 OS=Homo sapiens OX=9606 GN=KRT	0	97.549	57.52212	26	266	24	452	50.5	5.67		5.31
High	O15371	' EIF3D	Eukaryotic translation initiation factor 3 subunit D OS=Homo sapien	0	97.154	38.13869	17	59	17	548	63.9	6.05		4.995
High	P22061-2	' PCMT1	Isoform 2 of Protein-L-isoaspartate(D-aspartate) O-methyltransfera	0	97.144	66.66667	14	90	14	228	24.7	6.52		27.943
High	Q9H583	' HEATR1	HEAT repeat-containing protein 1 OS=Homo sapiens OX=9606 GN=H	0	97.039	16.32463	29	42	29	2144	242.2	6.54		0.63
High	Q9NVI7-2	' ATAD3A	Isoform 2 of ATPase family AAA domain-containing protein 3A OS=H	0	96.981	45.39249	24	113	10	586	66.2	9.25		3.479
High	P33992	' MCM5	DNA replication licensing factor MCM5 OS=Homo sapiens OX=9606	0	96.778	40.73569	22	95	22	734	82.2	8.37		2.014
High	O60292	' SIPA1L3	Signal-induced proliferation-associated 1-like protein 3 OS=Homo sa	0	96.371	22.96463	27	57	27	1781	194.5	8.32		0.778
High	P21281	' ATP6V1B2	V-type proton ATPase subunit B, brain isoform OS=Homo sapiens O	0	96.197	50.48924	21	68	15	511	56.5	5.81		6.356
High	O95782	' AP2A1	AP-2 complex subunit alpha-1 OS=Homo sapiens OX=9606 GN=AP2A	0	96.05	37.66633	28	86	20	977	107.5	7.03		2.103
High	P07237	' P4HB	Protein disulfide-isomerase OS=Homo sapiens OX=9606 GN=P4HB P	0	95.698	57.08661	24	99	24	508	57.1	4.87		3.732
High	P62136	' PPP1CA	Serine/threonine-protein phosphatase PP1-alpha catalytic subunit C	0	95.531	66.06061	19	104	5	330	37.5	6.33		10.22
High	P0DP25	' CALM3	Calmodulin-3 OS=Homo sapiens OX=9606 GN=CALM3 PE=1 SV=1	0	95.194	65.77181	13	113	12	149	16.8	4.22		124.893
High	Q96T37	' RBM15	RNA-binding protein 15 OS=Homo sapiens OX=9606 GN=RBM15 PE=	0	95.164	35.82399	25	75	24	977	107.1	10.08	Phospho	1.424
High	Q96M96	' FGD4	FYVE, RhoGEF and PH domain-containing protein 4 OS=Homo sapier	0	94.751	42.81984	25	75	25	766	86.6	6.13		2.525
High	P04075-2	' ALDOA	Isoform 2 of Fructose-bisphosphate aldolase A OS=Homo sapiens O	0	94.669	56.69856	19	132	19	418	45.2	8.25		11.115
High	P21796	' VDAC1	Voltage-dependent anion-selective channel protein 1 OS=Homo sap	0	94.483	68.55124	14	117	13	283	30.8	8.54		13.678
High	O00567	' NOP56	Nucleolar protein 56 OS=Homo sapiens OX=9606 GN=NOP56 PE=1 S	0	94.292	48.48485	25	49	25	594	66	9.19	Phospho	4.995
High	Q9ULV4-3	' CORO1C	Isoform 3 of Coronin-1C OS=Homo sapiens OX=9606 GN=CORO1C	0	94.051	39.46869	17	64	17	527	58.9	7.75		4.012
High	P51114	' FXR1	Fragile X mental retardation syndrome-related protein 1 OS=Homo	0	94.048	46.21578	23	63	20	621	69.7	6.15		3.642
High	Q12797	' ASPH	Aspartyl/asparaginyl beta-hydroxylase OS=Homo sapiens OX=9606	0	93.811	31.00264	22	72	22	758	85.8	5.01		3.281
High	O14617-5	' AP3D1	Isoform 5 of AP-3 complex subunit delta-1 OS=Homo sapiens OX=96	0	93.78	24.52675	23	66	23	1215	136.6	7.64		1.458
High	Q6ZUT6	' CCDC9B	Coiled-coil domain-containing protein 9B OS=Homo sapiens OX=960	0	93.677	58.05243	23	79	23	534	57.3	9.13	Phospho	6.055
High	Q14690	' PDCD11	Protein RRP5 homolog OS=Homo sapiens OX=9606 GN=PDCD11 PE=	0	93.455	21.16515	29	48	29	1871	208.6	8.87		0.77
High	Q8N884	' CGAS	Cyclic GMP-AMP synthase OS=Homo sapiens OX=9606 GN=CGAS PE	0	93.453	46.93487	22	75	22	522	58.8	9.48		5.225
High	Q15366-4	' PCBP2	Isoform 4 of Poly(rC)-binding protein 2 OS=Homo sapiens OX=9606	0	93.268	70.69486	15	102	1	331	34.9	8		17.33
High	Q9NQW6	' ANLN	Anillin OS=Homo sapiens OX=9606 GN=ANLN PE=1 SV=2	0	92.192	28.46975	29	98	29	1124	124.1	8.07		1.61
High	P26641-2	' EEF1G	Isoform 2 of Elongation factor 1-gamma OS=Homo sapiens OX=9606	0	92.139	47.43326	21	111	21	487	56.1	7.72		7.003
High	P62140	' PPP1CB	Serine/threonine-protein phosphatase PP1-beta catalytic subunit O	0	91.997	59.93884	17	97	4	327	37.2	6.19		7.913
High	O60841	' EIF5B	Eukaryotic translation initiation factor 5B OS=Homo sapiens OX=960	0	91.816	23.19672	19	66	19	1220	138.7	5.49		1.102
High	Q7Z478	' DHX29	ATP-dependent RNA helicase DHX29 OS=Homo sapiens OX=9606 GN	0	91.36	25.42001	25	48	25	1369	155.1	8.09		0.916
High	P23634	' ATP2B4	Plasma membrane calcium-transporting ATPase 4 OS=Homo sapiens	0	91.277	24.57695	24	72	17	1241	137.8	6.6		1.477
High	P04083	' ANXA1	Annexin A1 OS=Homo sapiens OX=9606 GN=ANXA1 PE=1 SV=2	0	91.076	65.31792	21	82	21	346	38.7	7.02		10.159
High	Q9UHX1-	' PUF60	Isoform 2 of Poly(U)-binding-splicing factor PUF60 OS=Homo sapien	0	90.991	57.74908	23	100	23	542	58.1	5.35		4.52
High	Q8WUM4	' PDCD6IP	Programmed cell death 6-interacting protein OS=Homo sapiens OX=	0	90.719	36.29032	25	71	25	868	96	6.52		2.097
High	Q16531	' DDB1	DNA damage-binding protein 1 OS=Homo sapiens OX=9606 GN=DDI	0	90.676	25.4386	24	73	24	1140	126.9	5.26		1.569
High	Q14974	' KPNB1	Importin subunit beta-1 OS=Homo sapiens OX=9606 GN=KPNB1 PE=	0	90.615	31.27854	19	77	19	876	97.1	4.78		2.511
High	Q14978-2	' NOLC1	Isoform Beta of Nucleolar and coiled-body phosphoprotein 1 OS=Ho	0	90.534	43.58251	25	63	25	709	74.7	9.47		2.942
High	Q96C19	' EFHD2	EF-hand domain-containing protein D2 OS=Homo sapiens OX=9606	0	90.436	71.25	17	118	17	240	26.7	5.2		21.539
High	P53396	' ACLY	ATP-citrate synthase OS=Homo sapiens OX=9606 GN=ACLY PE=1 SV	0	89.788	29.15531	24	72	24	1101	120.8	7.33		1.361
High	Q9Y2X3	' NOP58	Nucleolar protein 58 OS=Homo sapiens OX=9606 GN=NOP58 PE=1 S	0	89.706	45.74669	18	54	18	529	59.5	8.92		2.981
High	P22090	' RPS4Y1	40S ribosomal protein S4, Y isoform 1 OS=Homo sapiens OX=9606 G	0	89.61	52.09125	15	133	7	263	29.4	10.24		15.238
High	P13489	' RNH1	Ribonuclease inhibitor OS=Homo sapiens OX=9606 GN=RNH1 PE=1	0	89.415	48.80694	17	94	17	461	49.9	4.82		3.642
High	P48444	' ARCN1	Coatomer subunit delta OS=Homo sapiens OX=9606 GN=ARCN1 PE=	0	89.384	50.48924	24	111	24	511	57.2	6.21		6.356
High	P18669	' PGAM1	Phosphoglycerate mutase 1 OS=Homo sapiens OX=9606 GN=PGAM1	0	89.244	53.93701	14	81	10	254	28.8	7.18		28.286
High	Q92974	' ARHGEF2	Rho guanine nucleotide exchange factor 2 OS=Homo sapiens OX=96	0	89.203	29.10751	24	70	24	986	111.5	7.27		1.34
High	P26640	' VARS	Valine--tRNA ligase OS=Homo sapiens OX=9606 GN=VARS PE=1 SV=	0	89.196	20.88608	19	87	19	1264	140.4	7.59		0.868
High	Q14152	' EIF3A	Eukaryotic translation initiation factor 3 subunit A OS=Homo sapien	0	89.185	21.34588	24	68	24	1382	166.5	6.79		0.8
High	P54652	' HSPA2	Heat shock-related 70 kDa protein 2 OS=Homo sapiens OX=9606 GN	0	88.864	28.6385	18	133	6	639	70	5.74		2.849
High	P49915	' GMPS	GMP synthase [glutamine-hydrolyzing] OS=Homo sapiens OX=9606	0	88.854	47.90765	24	75	24	693	76.7	6.87		2.981
High	Q96MU7	' YTHDC1	YTH domain-containing protein 1 OS=Homo sapiens OX=9606 GN=Y	0	88.733	36.03851	22	87	22	727	84.6	6.23	Phospho	4.043
High	P24844	' MYL9	Myosin regulatory light polypeptide 9 OS=Homo sapiens OX=9606 G	0	88.635	50.5814	9	132	2	172	19.8	4.92		42.288
High	P46777	' RPL5	60S ribosomal protein L5 OS=Homo sapiens OX=9606 GN=RPL5 PE=	0	88.588	43.77104	14	80	14	297	34.3	9.72		13.678
High	Q7Z406-2	' MYH14	Isoform 2 of Myosin-14 OS=Homo sapiens OX=9606 GN=MYH14	0	88.367	7.121807	17	189	4	2036	231.9	5.6		0.535
High	Q9NZM1	' MYOF	Myoferlin OS=Homo sapiens OX=9606 GN=MYOF PE=1 SV=1	0	88.226	16.64241	27	62	27	2061	234.6	6.18		0.612
High	O15143	' ARPC1B	Actin-related protein 2/3 complex subunit 1B OS=Homo sapiens OX	0	88.175	48.3871	14	80	13	372	40.9	8.35		15.238
High	P46063	' RECQL	ATP-dependent DNA helicase Q1 OS=Homo sapiens OX=9606 GN=R	0	87.998	42.52696	24	87	24	649	73.4	7.88		3.125
High	P23396	' RPS3	40S ribosomal protein S3 OS=Homo sapiens OX=9606 GN=RPS3 PE=	0	87.895	74.4856	19	205	19	243	26.7	9.66	Phospho	15.238
High	P49023-3	' PXN	Isoform Gamma of Paxillin OS=Homo sapiens OX=9606 GN=PXN	0	86.926	49.09091	16	68	16	605	66.2	6.37	Phospho	3.532
High	P12268	' IMPDH2	Inosine-5'-monophosphate dehydrogenase 2 OS=Homo sapiens OX=	0	86.839	39.88327	19	69	18	514	55.8	6.9		4.995
High	P68400	' CSNK2A1	Casein kinase II subunit alpha OS=Homo sapiens OX=9606 GN=CSNK	0	86.655	53.19693	16	57	15	391	45.1	7.74		7.031
High	Q13263	' TRIM28	Transcription intermediary factor 1-beta OS=Homo sapiens OX=960	0	86.481	34.61078	20	90	20	835	88.5	5.77		2.849
High	Q14683	' SMC1A	Structural maintenance of chromosomes protein 1A OS=Homo sapie	0	86.405	25.46634	27	80	27	1233	143.1	7.64		1.027
High	P55084	' HADHB	Trifunctional enzyme subunit beta, mitochondrial OS=Homo sapiens	0	86.322	58.43882	21	90	21	474	51.3	9.41		4.52
High	Q99613	' EIF3C	Eukaryotic translation initiation factor 3 subunit C OS=Homo sapien	0	86.304	24.75356	23	90	23	913	105.3	5.68		2.481
High	P08708	' RPS17	40S ribosomal protein S17 OS=Homo sapiens OX=9606 GN=RPS17 P	0	86.296	60	9	65	9	135	15.5	9.85		1466.799
High	Q7L014	' DDX46	Probable ATP-dependent RNA helicase DDX46 OS=Homo sapiens O	0	86.154	29.38894	27	95	27	1031	117.3	9.29		1.878
High	Q9UH99	' SUN2	SUN domain-containing protein 2 OS=Homo sapiens OX=9606 GN=S	0	86.132	48.11715	20	53	20	717	80.3	6.73		3.184
High	Q14839-2	' CHD4	Isoform 2 of Chromodomain-helicase-DNA-binding protein 4 OS=Ho	0	85.808	17.06186	26	57	26	1940	220.7	5.97		0.798
High	Q07666	' KHDRBS1	KH domain-containing, RNA-binding, signal transduction-associated	0	85.796	33.63431	15	90	13	443	48.2	8.66		6.499
High	Q8IZW8	' TNS4	Tensin-4 OS=Homo sapiens OX=9606 GN=TNS4 PE=1 SV=3	0	85.78	48.11189	25	112	25	715	76.7	7.34		3.642
High	Q13200	' PSMD2	26S proteasome non-ATPase regulatory subunit 2 OS=Homo sapien	0	85.714	32.48899	22	57	22	908	100.1	5.2		1.759
High	Q96SB3	' PPP1R9B	Neurabin-2 OS=Homo sapiens OX=9606 GN=PPP1R9B PE=1 SV=2	0	85.7	30.79755	19	62	19	815	89.1	4.97		3.375
High	P0CG12	' CHTF8	Chromosome transmission fidelity protein 8 homolog isoform 2 OS=	0	85.636	61.64122	17	72	17	524	51.4	12.41		5.529
High	Q9UKV8	' AGO2	Protein argonaute-2 OS=Homo sapiens OX=9606 GN=AGO2 PE=1 SV	0	85.55	32.47963	21	62	15	859	97.1	9.19		1.512
High	Q9BQG0-	' MYBBP1A	Isoform 2 of Myb-binding protein 1A OS=Homo sapiens OX=9606 GI	0	85.204	23.12312	23	56	23	1332	149.3	9.28		1.054
High	P07910-2	' HNRNPC	Isoform C1 of Heterogeneous nuclear ribonucleoproteins C1/C2 OS=	0	85.114	44.3686	15	85	15	293	32.3	5.08		7.185
High	Q86V81	' ALYREF	THO complex subunit 4 OS=Homo sapiens OX=9606 GN=ALYREF PE=	0	85.033	52.91829	9	98	9	257	26.9	11.15		14.849
High	P26038	' MSN	Moesin OS=Homo sapiens OX=9606 GN=MSN PE=1 SV=3	0	84.983	45.40728	27	91	19	577	67.8	6.4		4.391
High	Q9BQ39	' DDX50	ATP-dependent RNA helicase DDX50 OS=Homo sapiens OX=9606 GI	0	84.841	31.88602	22	67	20	737	82.5	9.17		2.849
High	P42765	' ACAA2	3-ketoacyl-CoA thiolase, mitochondrial OS=Homo sapiens OX=9606	0	84.547	58.69018	17	53	17	397	41.9	8.09		7.185
High	P36873-2	' PPP1CC	Isoform 2 of Serine/threonine-protein phosphatase PP1-gamma cat	0	84.514	46.2908	16	87	2	337	38.5	6.13		5.579
High	P20042	' EIF2S2	Eukaryotic translation initiation factor 2 subunit 2 OS=Homo sapiens	0	84.492	55.85586	18	90	18	333	38.4	5.8		7.111
High	Q13451	' FKBP5	Peptidyl-prolyl cis-trans isomerase FKBP5 OS=Homo sapiens OX=960	0	84.335	54.92341	22	76	22	457	51.2	5.9		5.813
High	Q13572	' ITPK1	Inositol-tetrakisphosphate 1-kinase OS=Homo sapiens OX=9606 GN	0	84.198	63.76812	15	72	15	414	45.6	6.16		10.288
High	P47755	' CAPZA2	F-actin-capping protein subunit alpha-2 OS=Homo sapiens OX=9606	0	84.008	69.93007	13	101	11	286	32.9	5.85		14.849
High	Q14134	' TRIM29	Tripartite motif-containing protein 29 OS=Homo sapiens OX=9606 G	0	83.905	40.98639	22	72	22	588	65.8	7.15		3.467
High	P17480	' UBTF	Nucleolar transcription factor 1 OS=Homo sapiens OX=9606 GN=UB	0	83.844	28.01047	18	60	18	764	89.4	5.81		1.738
High	P19012	' KRT15	Keratin, type I cytoskeletal 15 OS=Homo sapiens OX=9606 GN=KRT1	0	83.778	39.47368	19	269	7	456	49.2	4.77		3.85
High	P08195-4	' SLC3A2	Isoform 4 of 4F2 cell-surface antigen heavy chain OS=Homo sapiens	0	83.723	36.15734	19	59	19	661	71.1	4.97		2.765
High	Q15678	' PTPN14	Tyrosine-protein phosphatase non-receptor type 14 OS=Homo sapie	0	83.582	31.33951	24	63	24	1187	135.2	8.31		1.839
High	Q12959-2	' DLG1	Isoform 2 of Disks large homolog 1 OS=Homo sapiens OX=9606 GN=	0	82.992	34.66523	21	54	21	926	103.3	5.92		2.415
High	P55884-2	' EIF3B	Isoform 2 of Eukaryotic translation initiation factor 3 subunit B OS=	0	82.933	31.04238	19	72	19	873	99	5.08		1.721
High	P08865	' RPSA	40S ribosomal protein SA OS=Homo sapiens OX=9606 GN=RPSA PE=	0	82.238	52.20339	10	71	10	295	32.8	4.87		9
High	P62263	' RPS14	40S ribosomal protein S14 OS=Homo sapiens OX=9606 GN=RPS14 P	0	82.16	69.53642	11	77	11	151	16.3	10.05		267.27
High	Q9H307	' PNN	Pinin OS=Homo sapiens OX=9606 GN=PNN PE=1 SV=5	0	82.073	35.84379	22	72	22	717	81.6	7.14		2.899
High	Q04637-9	' EIF4G1	Isoform 9 of Eukaryotic translation initiation factor 4 gamma 1 OS=	0	82.036	18.86675	23	69	23	1606	176.1	5.34		1.05
High	P23528	' CFL1	Cofilin-1 OS=Homo sapiens OX=9606 GN=CFL1 PE=1 SV=3	0	81.731	72.89157	14	72	11	166	18.5	8.09		25.102
High	P51659	' HSD17B4	Peroxisomal multifunctional enzyme type 2 OS=Homo sapiens OX=9	0	81.408	35.46196	18	66	18	736	79.6	8.84		2.001
High	P31153	' MAT2A	S-adenosylmethionine synthase isoform type-2 OS=Homo sapiens O	0	81.223	52.65823	19	76	19	395	43.6	6.48		11.217
High	P05388	' RPLP0	60S acidic ribosomal protein P0 OS=Homo sapiens OX=9606 GN=RPI	0	81.081	57.72871	14	82	14	317	34.3	5.97		5.952
High	P62906	' RPL10A	60S ribosomal protein L10a OS=Homo sapiens OX=9606 GN=RPL10A	0	80.784	55.29954	15	97	15	217	24.8	9.94		24.119
High	Q9Y224	' RTRAF	RNA transcription, translation and transport factor protein OS=Hom	0	80.573	73.36066	17	91	17	244	28.1	6.65		10.365
High	P30876	' POLR2B	DNA-directed RNA polymerase II subunit RPB2 OS=Homo sapiens O	0	80.303	26.14991	24	54	24	1174	133.8	6.87		1.271
High	Q14195-2	' DPYSL3	Isoform LCRMP-4 of Dihydropyrimidinase-related protein 3 OS=Hom	0	80.093	38.15789	17	65	14	684	73.9	6.35		2.35
High	P01834	' IGKC	Immunoglobulin kappa constant OS=Homo sapiens OX=9606 GN=IG	0	79.745	82.24299	8	100	2	107	11.8	6.52		1583.893
High	Q12904-2	' AIMP1	Isoform 2 of Aminoacyl tRNA synthase complex-interacting multifun	0	79.514	65.47619	14	82	14	336	37	8.65		6.197
High	P62753	' RPS6	40S ribosomal protein S6 OS=Homo sapiens OX=9606 GN=RPS6 PE=	0	79.445	42.97189	15	103	15	249	28.7	10.84		27.943
High	P09493-3	' TPM1	Isoform 3 of Tropomyosin alpha-1 chain OS=Homo sapiens OX=9606	0	79.444	55.6338	23	80	8	284	32.9	4.78		13.925
High	Q96I24	' FUBP3	Far upstream element-binding protein 3 OS=Homo sapiens OX=960	0	79.332	43.70629	17	92	16	572	61.6	8.38		2.57
High	P63244	' RACK1	Receptor of activated protein C kinase 1 OS=Homo sapiens OX=9606	0	79.3	63.7224	16	65	16	317	35.1	7.69		7.111
High	Q15646	' OASL	2'-5'-oligoadenylate synthase-like protein OS=Homo sapiens OX=96	0	79.255	47.66537	21	77	21	514	59.2	7.87		4.843
High	Q9P2M7	' CGN	Cingulin OS=Homo sapiens OX=9606 GN=CGN PE=1 SV=2	0	79.229	20.96909	20	43	20	1197	136.3	5.54		0.691
High	Q9UGU0	' TCF20	Transcription factor 20 OS=Homo sapiens OX=9606 GN=TCF20 PE=1	0	79.116	23.36735	25	45	25	1960	211.6	9.04		0.788
High	P26599-3	' PTBP1	Isoform 3 of Polypyrimidine tract-binding protein 1 OS=Homo sapie	0	78.974	39.31777	13	77	11	557	59.6	9.16		3.786
High	P16403	' HIST1H1C	Histone H1.2 OS=Homo sapiens OX=9606 GN=HIST1H1C PE=1 SV=2	0	78.96	32.39437	15	203	4	213	21.4	10.93	Phospho	27.943
High	P23526	' AHCY	Adenosylhomocysteinase OS=Homo sapiens OX=9606 GN=AHCY PE=	0	78.626	39.35185	18	88	16	432	47.7	6.34		6.586
High	B1AK53	' ESPN	Espin OS=Homo sapiens OX=9606 GN=ESPN PE=1 SV=1	0	78.553	24.59016	17	64	17	854	91.7	6.93		2.728
High	Q03001-3	' DST	Isoform 3 of Dystonin OS=Homo sapiens OX=9606 GN=DST	0	78.411	12.60853	28	60	7	2649	306.6	6.32		0.402
High	Q9Y4L1	' HYOU1	Hypoxia up-regulated protein 1 OS=Homo sapiens OX=9606 GN=HY	0	78.404	25.02503	19	47	19	999	111.3	5.22		1.239
High	O75694	' NUP155	Nuclear pore complex protein Nup155 OS=Homo sapiens OX=9606	0	78.184	22.57369	20	71	20	1391	155.1	6.16		1.081
High	P06899	' HIST1H2BJ	Histone H2B type 1-J OS=Homo sapiens OX=9606 GN=HIST1H2BJ PE	0	77.962	57.93651	9	464	2	126	13.9	10.32		34.112
High	P63104	' YWHAZ	14-3-3 protein zeta/delta OS=Homo sapiens OX=9606 GN=YWHAZ P	0	77.823	56.73469	15	99	11	245	27.7	4.79		7.859
High	P06753	' TPM3	Tropomyosin alpha-3 chain OS=Homo sapiens OX=9606 GN=TPM3 F	0	77.817	40.70175	17	96	2	285	32.9	4.72		6.499
High	Q5QNW6	' HIST2H2BF	Isoform 2 of Histone H2B type 2-F OS=Homo sapiens OX=9606 GN=I	0	77.752	54.47761	9	506	2	134	14.8	10.35		25.102
High	Q14694-2	' USP10	Isoform 2 of Ubiquitin carboxyl-terminal hydrolase 10 OS=Homo sap	0	77.707	30.49645	19	57	19	846	92.5	5.69	Phospho	2.162
High	P55786	' NPEPPS	Puromycin-sensitive aminopeptidase OS=Homo sapiens OX=9606 GI	0	77.345	25.0272	20	53	20	919	103.2	5.72		1.432
High	P22102	' GART	Trifunctional purine biosynthetic protein adenosine-3 OS=Homo sap	0	76.943	24.35644	19	74	19	1010	107.7	6.7		1.154
High	P19525	' EIF2AK2	Interferon-induced, double-stranded RNA-activated protein kinase	0	76.75	37.56806	17	77	17	551	62.1	8.4		4.38
High	Q8NE71	' ABCF1	ATP-binding cassette sub-family F member 1 OS=Homo sapiens OX=	0	76.576	30.29586	20	68	20	845	95.9	6.8		1.664
High	P60174	' TPI1	Triosephosphate isomerase OS=Homo sapiens OX=9606 GN=TPI1 PE	0	76.434	70.97902	14	66	14	286	30.8	5.92		7.859
High	O75044	' SRGAP2	SLIT-ROBO Rho GTPase-activating protein 2 OS=Homo sapiens OX=9	0	76.414	27.73109	22	53	22	1071	120.8	6.7		1.637
High	Q13501	' SQSTM1	Sequestosome-1 OS=Homo sapiens OX=9606 GN=SQSTM1 PE=1 SV=	0	76.406	62.27273	13	56	13	440	47.7	5.22		7.859
High	Q9Y263	' PLAA	Phospholipase A-2-activating protein OS=Homo sapiens OX=9606 GI	0	76.309	32.07547	21	76	21	795	87.1	6.37		1.825
High	Q13247	' SRSF6	Serine/arginine-rich splicing factor 6 OS=Homo sapiens OX=9606 GN	0	75.881	36.33721	15	104	10	344	39.6	11.43		9
High	Q9HCE1	' MOV10	Putative helicase MOV-10 OS=Homo sapiens OX=9606 GN=MOV10	0	75.761	30.20937	24	46	24	1003	113.6	8.82		1.438
High	Q3KQU3	' MAP7D1	MAP7 domain-containing protein 1 OS=Homo sapiens OX=9606 GN=	0	75.168	37.3365	24	98	24	841	92.8	10.11		2.318
High	A0FGR8-6	' ESYT2	Isoform 6 of Extended synaptotagmin-2 OS=Homo sapiens OX=9606	0	74.587	30.25478	20	57	20	942	104.6	9.33		1.666
High	P10412	' HIST1H1E	Histone H1.4 OS=Homo sapiens OX=9606 GN=HIST1H1E PE=1 SV=2	0	74.518	42.92237	15	209	4	219	21.9	11.03	Phospho	37.312
High	P09913	' IFIT2	Interferon-induced protein with tetratricopeptide repeats 2 OS=Hor	0	74.282	41.73729	15	34	15	472	54.6	6.76		2.511
High	P62241	' RPS8	40S ribosomal protein S8 OS=Homo sapiens OX=9606 GN=RPS8 PE=	0	74.275	58.65385	12	159	12	208	24.2	10.32		49.119
High	O76094	' SRP72	Signal recognition particle subunit SRP72 OS=Homo sapiens OX=960	0	74.236	33.38301	18	48	18	671	74.6	9.26		1.834
High	Q9Y265	' RUVBL1	RuvB-like 1 OS=Homo sapiens OX=9606 GN=RUVBL1 PE=1 SV=1	0	74.054	42.54386	15	64	15	456	50.2	6.42		3.642
High	P22087	' FBL	rRNA 2'-O-methyltransferase fibrillarin OS=Homo sapiens OX=9606	0	74.005	55.45171	16	54	16	321	33.8	10.18		5.062
High	P40926	' MDH2	Malate dehydrogenase, mitochondrial OS=Homo sapiens OX=9606	0	73.984	49.40828	13	79	13	338	35.5	8.68		3.365
High	Q9UGI8	' TES	Testin OS=Homo sapiens OX=9606 GN=TES PE=1 SV=1	0	73.945	49.88124	22	92	22	421	48	7.68		6.197
High	O76021	' RSL1D1	Ribosomal L1 domain-containing protein 1 OS=Homo sapiens OX=96	0	73.827	35.91837	21	48	21	490	54.9	10.13		5.422
High	O75083	' WDR1	WD repeat-containing protein 1 OS=Homo sapiens OX=9606 GN=WI	0	73.734	39.27393	17	56	17	606	66.2	6.65		2.257
High	P49588-2	' AARS	Isoform 2 of Alanine--tRNA ligase, cytoplasmic OS=Homo sapiens O	0	73.73	25.80645	18	47	18	992	109.2	5.57		1.302
High	Q8IXT5	' RBM12B	RNA-binding protein 12B OS=Homo sapiens OX=9606 GN=RBM12B	0	73.336	21.27872	20	58	20	1001	118	6.81		0.753
High	P48735	' IDH2	Isocitrate dehydrogenase [NADP], mitochondrial OS=Homo sapiens	0	73.314	46.23894	20	71	19	452	50.9	8.69		4.52
High	P05387	' RPLP2	60S acidic ribosomal protein P2 OS=Homo sapiens OX=9606 GN=RPI	0	73.278	95.65217	10	77	9	115	11.7	4.54	Phospho	214.443
High	Q02413	' DSG1	Desmoglein-1 OS=Homo sapiens OX=9606 GN=DSG1 PE=1 SV=2	0	72.903	22.40229	17	77	17	1049	113.7	5.03		2.162
High	Q96QK1	' VPS35	Vacuolar protein sorting-associated protein 35 OS=Homo sapiens O	0	72.845	31.78392	19	67	19	796	91.6	5.49		1.512
High	Q9Y230	' RUVBL2	RuvB-like 2 OS=Homo sapiens OX=9606 GN=RUVBL2 PE=1 SV=3	0	72.768	45.57235	18	59	18	463	51.1	5.64		3.52
High	P0DOX7	' NA	Immunoglobulin kappa light chain OS=Homo sapiens OX=9606 PE=1	0	72.401	49.53271	8	87	2	214	23.4	7.17		22.101
High	P27694	' RPA1	Replication protein A 70 kDa DNA-binding subunit OS=Homo sapien	0	72.197	41.88312	18	74	18	616	68.1	7.21		2.472
High	Q13838-2	' DDX39B	Isoform 2 of Spliceosome RNA helicase DDX39B OS=Homo sapiens	0	72.052	45.37246	16	59	6	443	50.6	5.88		3.52
High	Q15007	' WTAP	Pre-mRNA-splicing regulator WTAP OS=Homo sapiens OX=9606 GN=	0	72.023	58.58586	19	56	19	396	44.2	5.19		4.878
High	P16402	' HIST1H1D	Histone H1.3 OS=Homo sapiens OX=9606 GN=HIST1H1D PE=1 SV=2	0	71.983	38.46154	15	193	6	221	22.3	11.02	Phospho	27.943
High	P16401	' HIST1H1B	Histone H1.5 OS=Homo sapiens OX=9606 GN=HIST1H1B PE=1 SV=3	0	71.609	33.18584	13	184	10	226	22.6	10.92		23.245
High	P34932	' HSPA4	Heat shock 70 kDa protein 4 OS=Homo sapiens OX=9606 GN=HSPA4	0	71.596	27.2619	18	49	16	840	94.3	5.19		1.043
High	Q5QJE6	' DNTTIP2	Deoxynucleotidyltransferase terminal-interacting protein 2 OS=Hom	0	71.499	29.62963	15	45	15	756	84.4	6.16		1.54
High	Q86UP2	' KTN1	Kinectin OS=Homo sapiens OX=9606 GN=KTN1 PE=1 SV=1	0	71.37	20.78113	22	54	22	1357	156.2	5.64		0.746
High	Q9NZL9	' MAT2B	Methionine adenosyltransferase 2 subunit beta OS=Homo sapiens	0	71.293	51.1976	18	39	18	334	37.5	7.36		9
High	P15311	' EZR	Ezrin OS=Homo sapiens OX=9606 GN=EZR PE=1 SV=4	0	71.205	35.83618	21	86	13	586	69.4	6.27		2.888
High	Q99623	' PHB2	Prohibitin-2 OS=Homo sapiens OX=9606 GN=PHB2 PE=1 SV=2	0	71.14	59.86622	17	95	17	299	33.3	9.83		3.924
High	Q8WUI4-	' HDAC7	Isoform 8 of Histone deacetylase 7 OS=Homo sapiens OX=9606 GN=	0	70.705	33.33333	20	67	20	1014	108.9	8.51		1.7
High	O75400	' PRPF40A	Pre-mRNA-processing factor 40 homolog A OS=Homo sapiens OX=9	0	70.648	25.28736	22	69	2	957	108.7	7.56		2.849
High	P23381	' WARS	Tryptophan--tRNA ligase, cytoplasmic OS=Homo sapiens OX=9606 G	0	70.641	41.40127	15	56	15	471	53.1	6.23		2.687
High	Q9UMD9	' COL17A1	Collagen alpha-1(XVII) chain OS=Homo sapiens OX=9606 GN=COL17	0	70.581	17.50167	20	52	20	1497	150.3	8.79		1.154
**High**	**Q8TEB7**	**' RNF128**	**E3 ubiquitin-protein ligase RNF128 OS=Homo sapiens OX=9606 GN**	**0**	**70.561**	**25.23364**	**9**	**44**	**4**	**428**	**46.5**	**6.35**		**7.733**
High	Q96CW1	' AP2M1	AP-2 complex subunit mu OS=Homo sapiens OX=9606 GN=AP2M1 F	0	70.537	45.74713	18	64	18	435	49.6	9.54		3.894
High	Q15019-2	' SEPT2	Isoform 2 of Septin-2 OS=Homo sapiens OX=9606 GN=SEPT2	0	70.18	47.22222	12	60	12	396	45.4	6.89	Phospho	2.981
High	Q14240-2	' EIF4A2	Isoform 2 of Eukaryotic initiation factor 4A-II OS=Homo sapiens OX=	0	69.981	41.17647	14	87	5	408	46.5	5.48		4.109
High	P50402	' EMD	Emerin OS=Homo sapiens OX=9606 GN=EMD PE=1 SV=1	0	69.755	70.86614	15	89	15	254	29	5.5	Phospho	14.849
High	P30101	' PDIA3	Protein disulfide-isomerase A3 OS=Homo sapiens OX=9606 GN=PDI/	0	69.73	47.72277	19	72	19	505	56.7	6.35		3.037
High	Q02539	' HIST1H1A	Histone H1.1 OS=Homo sapiens OX=9606 GN=HIST1H1A PE=1 SV=3	0	69.515	32.09302	13	110	8	215	21.8	10.99		34.112
High	P67809	' YBX1	Nuclease-sensitive element-binding protein 1 OS=Homo sapiens OX	0	69.44	50.30864	13	85	8	324	35.9	9.88		15.379
High	Q7KZI7-1	' MARK2	Isoform 16 of Serine/threonine-protein kinase MARK2 OS=Homo sa	0	69.307	33.83978	19	53	15	724	81.2	9.57		1.703
High	P31939	' ATIC	Bifunctional purine biosynthesis protein PURH OS=Homo sapiens O	0	69.295	38.34459	17	58	17	592	64.6	6.71		2.262
High	Q6NZI2	' CAVIN1	Caveolae-associated protein 1 OS=Homo sapiens OX=9606 GN=CAV	0	69.184	33.33333	12	61	12	390	43.5	5.6		5.813
High	Q07955	' SRSF1	Serine/arginine-rich splicing factor 1 OS=Homo sapiens OX=9606 GN	0	69.009	53.22581	17	170	17	248	27.7	10.36		14.849
High	P20020	' ATP2B1	Plasma membrane calcium-transporting ATPase 1 OS=Homo sapiens	0	68.934	18.36248	15	40	8	1258	138.7	6.04		0.764
High	Q2KHR2	' RFX7	DNA-binding protein RFX7 OS=Homo sapiens OX=9606 GN=RFX7 PE	0	68.874	21.86354	20	61	20	1363	146.8	6.76	Phospho	1.154
High	Q5SSJ5	' HP1BP3	Heterochromatin protein 1-binding protein 3 OS=Homo sapiens OX	0	68.799	32.91139	16	52	16	553	61.2	9.67		2.511
High	P24752	' ACAT1	Acetyl-CoA acetyltransferase, mitochondrial OS=Homo sapiens OX=	0	68.749	50.58548	16	68	16	427	45.2	8.85		3.262
High	P30838	' ALDH3A1	Aldehyde dehydrogenase, dimeric NADP-preferring OS=Homo sapie	0	68.708	44.81236	16	51	15	453	50.4	6.54		4.623

High	Q8IXB1	' DNAJC10	DnaJ homolog subfamily C member 10 OS=Homo sapiens OX=9606	0	68.623	27.86889	18	67	18	793	91	7.18		2.665
High	P62937	' PPIA	Peptidyl-prolyl cis-trans isomerase A OS=Homo sapiens OX=9606 GN	0	68.384	49.09091	8	113	8	165	18	7.81		10.788
High	Q92896-2	' GLG1	Isoform 2 of Golgi apparatus protein 1 OS=Homo sapiens OX=9606	0	68.201	21.03076	18	30	18	1203	137.1	6.9		0.821
High	O60437	' PPL	Periplakin OS=Homo sapiens OX=9606 GN=PPL PE=1 SV=4	0	68.158	15.60364	23	42	23	1756	204.6	5.6		0.48
High	O43790	' KRT86	Keratin, type II cuticular Hb6 OS=Homo sapiens OX=9606 GN=KRT86	0	68.119	50.41152	20	62	8	486	53.5	5.66		3.541
High	Q562R1	' ACTBL2	Beta-actin-like protein 2 OS=Homo sapiens OX=9606 GN=ACTBL2 PE	0	67.906	33.7766	9	495	4	376	42	5.59		4.109
High	P63241-2	' EIF5A	Isoform 2 of Eukaryotic translation initiation factor 5A-1 OS=Homo s	0	67.845	42.93478	10	56	10	184	20.2	7.01		18.953
High	O75400-3	' PRPF40A	Isoform 3 of Pre-mRNA-processing factor 40 homolog A OS=Homo s	0	67.765	23.85517	21	68	1	939	106.8	7.43		2.758
High	P22695	' UQCRC2	Cytochrome b-c1 complex subunit 2, mitochondrial OS=Homo sapie	0	67.561	33.77483	11	41	11	453	48.4	8.63		3.365
High	Q9NYT0	' PLEK2	Pleckstrin-2 OS=Homo sapiens OX=9606 GN=PLEK2 PE=1 SV=1	0	67.211	46.45892	15	41	15	353	39.9	9.41		5.062
High	P61313	' RPL15	60S ribosomal protein L15 OS=Homo sapiens OX=9606 GN=RPL15 P	0	67.165	50	11	98	11	204	24.1	11.62		15.379
High	P26639-2	' TARS	Isoform 2 of Threonine--tRNA ligase, cytoplasmic OS=Homo sapiens	0	67.012	33.46561	21	58	21	756	86.8	6.77		1.683
High	O00154	' ACOT7	Cytosolic acyl coenzyme A thioester hydrolase OS=Homo sapiens OX	0	66.932	37.36842	12	64	12	380	41.8	8.54		3.806
High	P50479	' PDLIM4	PDZ and LIM domain protein 4 OS=Homo sapiens OX=9606 GN=PDL	0	66.855	71.81818	17	68	17	330	35.4	7.91	Phospho	7.962
High	P09493-5	' TPM1	Isoform 5 of Tropomyosin alpha-1 chain OS=Homo sapiens OX=9606	0	66.669	42.85714	16	68	3	245	28.4	4.77		7.111
High	O00429-6	' DNM1L	Isoform 6 of Dynamin-1-like protein OS=Homo sapiens OX=9606 GN	0	66.669	31.10814	16	42	16	749	83.3	7.08		1.093
High	Q5T749	' KPRP	Keratinocyte proline-rich protein OS=Homo sapiens OX=9606 GN=K	0	66.637	30.91537	12	77	12	579	64.1	8.27		7.185
High	P53992	' SEC24C	Protein transport protein Sec24C OS=Homo sapiens OX=9606 GN=S	0	66.635	20.01828	17	69	16	1094	118.2	7.06		1.371
High	P27348	' YWHAQ	14-3-3 protein theta OS=Homo sapiens OX=9606 GN=YWHAQ PE=1	0	66.494	60	17	76	12	245	27.7	4.78		7.799
High	P14868	' DARS	Aspartate--tRNA ligase, cytoplasmic OS=Homo sapiens OX=9606 GN	0	66.363	46.50699	20	57	20	501	57.1	6.55		2.162
High	P61011	' SRP54	Signal recognition particle 54 kDa protein OS=Homo sapiens OX=96	0	66.33	41.66667	19	45	19	504	55.7	8.75		2.262
High	Q92882	' OSTF1	Osteoclast-stimulating factor 1 OS=Homo sapiens OX=9606 GN=OST	0	66.26	55.14019	11	40	11	214	23.8	5.68		9
High	P17655	' CAPN2	Calpain-2 catalytic subunit OS=Homo sapiens OX=9606 GN=CAPN2	0	66.197	28.85714	17	55	17	700	79.9	4.98		2.162
High	Q8TF72	' SHROOM3	Protein Shroom3 OS=Homo sapiens OX=9606 GN=SHROOM3 PE=1 S	0	66.104	17.23447	24	46	22	1996	216.7	7.8		0.609
High	Q99700	' ATXN2	Ataxin-2 OS=Homo sapiens OX=9606 GN=ATXN2 PE=1 SV=2	0	66.054	15.15613	15	56	13	1313	140.2	9.57		1.089
High	P04899-4	' GNAI2	Isoform sGi2 of Guanine nucleotide-binding protein G(i) subunit alp	0	66.028	41.80328	13	58	8	366	41.5	5.99		4.78
High	P46087-4	' NOP2	Isoform 4 of Probable 28S rRNA (cytosine(4447)-C(5))-methyltransfe	0	65.972	26.50888	18	46	18	845	92.8	9.19		1.512
High	P08240	' SRPRA	Signal recognition particle receptor subunit alpha OS=Homo sapiens	0	65.966	31.34796	16	58	16	638	69.8	8.95		1.748
High	Q9NVP1	' DDX18	ATP-dependent RNA helicase DDX18 OS=Homo sapiens OX=9606 G	0	65.945	31.19403	15	37	15	670	75.4	9.5		2.371
High	P18621-3	' RPL17	Isoform 3 of 60S ribosomal protein L17 OS=Homo sapiens OX=9606	0	65.791	45.61404	11	114	11	228	26.4	10.1		10.659
High	P49792	' RANBP2	E3 SUMO-protein ligase RanBP2 OS=Homo sapiens OX=9606 GN=RA	0	65.697	11.22829	23	56	23	3224	358	6.2		0.268
High	Q9H0J9	' PARP12	Poly [ADP-ribose] polymerase 12 OS=Homo sapiens OX=9606 GN=P	0	65.495	29.81455	19	90	18	701	79	8.51		2.332
High	P05114	' HMGN1	Non-histone chromosomal protein HMG-14 OS=Homo sapiens OX=	0	65.401	59	12	55	12	100	10.7	9.6		680.292
High	Q9UJZ1	' STOML2	Stomatin-like protein 2, mitochondrial OS=Homo sapiens OX=9606	0	65.242	46.06742	12	52	12	356	38.5	7.39		3.467
High	P32969	' RPL9	60S ribosomal protein L9 OS=Homo sapiens OX=9606 GN=RPL9 PE=	0	64.923	61.97917	10	54	10	192	21.9	9.95		17.738
High	Q12905	' ILF2	Interleukin enhancer-binding factor 2 OS=Homo sapiens OX=9606 G	0	64.533	42.82051	13	56	13	390	43	5.26		3.642
High	Q9NSD9	' FARSB	Phenylalanine--tRNA ligase beta subunit OS=Homo sapiens OX=960	0	64.512	32.93718	18	72	18	589	66.1	6.84		1.537
High	Q86UE4	' MTDH	Protein LYRIC OS=Homo sapiens OX=9606 GN=MTDH PE=1 SV=2	0	64.408	36.59794	16	49	16	582	63.8	9.32		2.981
High	Q8NC56	' LEMD2	LEM domain-containing protein 2 OS=Homo sapiens OX=9606 GN=L	0	64.402	38.9662	15	54	15	503	56.9	9		2.054
High	Q9H0S4	' DDX47	Probable ATP-dependent RNA helicase DDX47 OS=Homo sapiens OX	0	64.188	36.7033	14	47	14	455	50.6	9.1	Phospho	2.775
High	O75955	' FLOT1	Flotillin-1 OS=Homo sapiens OX=9606 GN=FLOT1 PE=1 SV=3	0	64.112	45.19906	15	32	15	427	47.3	7.49		2.282
High	Q13151	' HNRNPA0	Heterogeneous nuclear ribonucleoprotein A0 OS=Homo sapiens OX	0	64.109	47.86885	11	61	10	305	30.8	9.29		7.577
High	P49761	' CLK3	Dual specificity protein kinase CLK3 OS=Homo sapiens OX=9606 GN	0	63.962	30.09404	16	46	16	638	73.5	9.92		1.598
High	Q14257-2	' RCN2	Isoform 2 of Reticulocalbin-2 OS=Homo sapiens OX=9606 GN=RCN2	0	63.936	42.08955	13	42	13	335	39.1	4.45		5.31
High	Q86UW9	' DTX2	Probable E3 ubiquitin-protein ligase DTX2 OS=Homo sapiens OX=96	0	63.804	38.26367	13	44	3	622	67.2	8.47		2.857
High	Q9H7E2-3	' TDRD3	Isoform 3 of Tudor domain-containing protein 3 OS=Homo sapiens	0	63.313	32.52688	18	40	18	744	83.1	9		1.371
High	Q8N1G4	' LRRC47	Leucine-rich repeat-containing protein 47 OS=Homo sapiens OX=96	0	63.274	42.19554	17	51	17	583	63.4	8.28		2.06
High	Q8IY33	' MICALL2	MICAL-like protein 2 OS=Homo sapiens OX=9606 GN=MICALL2 PE=1	0	63.145	28.31858	17	36	16	904	97.4	9.57		0.987
High	P62714	' PPP2CB	Serine/threonine-protein phosphatase 2A catalytic subunit beta iso	0	63.119	50.16181	12	58	12	309	35.6	5.43		5.158
High	Q2TAY7	' SMU1	WD40 repeat-containing protein SMU1 OS=Homo sapiens OX=9606	0	63.019	36.64717	16	56	16	513	57.5	7.18		3.037
High	Q14697	' GANAB	Neutral alpha-glucosidase AB OS=Homo sapiens OX=9606 GN=GAN	0	62.832	29.76695	20	52	20	944	106.8	6.14		1.31
High	Q15084-2	' PDIA6	Isoform 2 of Protein disulfide-isomerase A6 OS=Homo sapiens OX=9	0	62.8	42.88618	14	57	14	492	53.9	5.33		2.162
High	P0C0S8	' HIST1H2AG	Histone H2A type 1 OS=Homo sapiens OX=9606 GN=HIST1H2AG PE	0	62.797	68.46154	9	267	1	130	14.1	10.9		192.07
High	P12532	' CKMT1A	Creatine kinase U-type, mitochondrial OS=Homo sapiens OX=9606	0	62.705	39.56835	12	43	12	417	47	8.34		2.03
High	P23258	' TUBG1	Tubulin gamma-1 chain OS=Homo sapiens OX=9606 GN=TUBG1 PE=	0	62.669	37.47228	12	77	12	451	51.1	6.14		3.806
High	Q8NFW8	' CMAS	N-acylneuraminate cytidylyltransferase OS=Homo sapiens OX=9606	0	62.643	39.40092	16	50	16	434	48.3	7.93		2.687
High	P19784	' CSNK2A2	Casein kinase II subunit alpha' OS=Homo sapiens OX=9606 GN=CSN	0	62.567	55.42857	15	35	14	350	41.2	8.56		3.962
High	Q6IQ23-2	' PLEKHA7	Isoform 2 of Pleckstrin homology domain-containing family A memb	0	62.502	25.66845	19	44	19	1122	127.2	9.35		0.905
High	Q96R06	' SPAG5	Sperm-associated antigen 5 OS=Homo sapiens OX=9606 GN=SPAG5	0	62.455	23.3026	20	39	20	1193	134.3	5		0.913
High	Q9HAU0-	' PLEKHA5	Isoform 6 of Pleckstrin homology domain-containing family A memb	0	62.424	17.70671	14	40	14	1282	146.8	7.65		0.612
High	P35232	' PHB	Prohibitin OS=Homo sapiens OX=9606 GN=PHB PE=1 SV=1	0	62.39	63.60294	14	68	14	272	29.8	5.76		5.813
High	P24928	' POLR2A	DNA-directed RNA polymerase II subunit RPB1 OS=Homo sapiens O	0	62.288	14.56853	21	52	21	1970	217	7.37		0.559
High	P31689	' DNAJA1	DnaJ homolog subfamily A member 1 OS=Homo sapiens OX=9606 G	0	62.241	46.85139	12	51	12	397	44.8	7.08		4.337
High	Q15717-2	' ELAVL1	Isoform 2 of ELAV-like protein 1 OS=Homo sapiens OX=9606 GN=EL	0	62.188	43.34278	12	66	12	353	39	9.45		5.31
High	Q6PKG0	' LARP1	La-related protein 1 OS=Homo sapiens OX=9606 GN=LARP1 PE=1 SV	0	62.023	20.62044	17	47	17	1096	123.4	8.82		0.764
High	Q16658	' FSCN1	Fascin OS=Homo sapiens OX=9606 GN=FSCN1 PE=1 SV=3	0	61.983	40.16227	16	61	16	493	54.5	7.24		2.857
High	Q8NI36	' WDR36	WD repeat-containing protein 36 OS=Homo sapiens OX=9606 GN=W	0	61.943	26.91903	17	46	17	951	105.3	7.53		1.254
High	P61163	' ACTR1A	Alpha-centractin OS=Homo sapiens OX=9606 GN=ACTR1A PE=1 SV=	0	61.942	45.74468	12	54	6	376	42.6	6.64		3.062
High	P78386	' KRT85	Keratin, type II cuticular Hb5 OS=Homo sapiens OX=9606 GN=KRT85	0	61.936	37.86982	17	57	5	507	55.8	6.55		2.472
High	O00425	' IGF2BP3	Insulin-like growth factor 2 mRNA-binding protein 3 OS=Homo sapie	0	61.559	36.09672	16	52	13	579	63.7	8.87		2.06
High	P27635	' RPL10	60S ribosomal protein L10 OS=Homo sapiens OX=9606 GN=RPL10 P	0	61.397	42.99065	10	95	10	214	24.6	10.08		13.251
High	P18583-5	' SON	Isoform D of Protein SON OS=Homo sapiens OX=9606 GN=SON	0	61.382	14.72143	20	47	20	2459	266.9	5.73		0.722
High	Q06830	' PRDX1	Peroxiredoxin-1 OS=Homo sapiens OX=9606 GN=PRDX1 PE=1 SV=1	0	61.358	73.86935	15	84	12	199	22.1	8.13		21.758
High	Q52LJ0-2	' FAM98B	Isoform 2 of Protein FAM98B OS=Homo sapiens OX=9606 GN=FAM	0	61.203	35.79677	10	62	8	433	45.5	8.69		1.807
High	P45880-1	' VDAC2	Isoform 1 of Voltage-dependent anion-selective channel protein 2	0	61.165	43.04207	11	74	11	309	33.4	7.59		5.952
High	O94973-2	' AP2A2	Isoform 2 of AP-2 complex subunit alpha-2 OS=Homo sapiens OX=9	0	61.149	25.42553	18	51	10	940	104	6.86		1.096
High	Q9Y383	' LUC7L2	Putative RNA-binding protein Luc7-like 2 OS=Homo sapiens OX=960	0	61.049	36.22449	14	83	8	392	46.5	10.01		5.952
High	P61106	' RAB14	Ras-related protein Rab-14 OS=Homo sapiens OX=9606 GN=RAB14	0	60.943	73.48837	13	61	12	215	23.9	6.21		6.356
High	O00231-2	' PSMD11	Isoform 2 of 26S proteasome non-ATPase regulatory subunit 11 OS=	0	60.909	41.37116	15	56	15	423	47.5	6.48		2.398
High	P16104	' H2AFX	Histone H2AX OS=Homo sapiens OX=9606 GN=H2AFX PE=1 SV=2	0	60.764	58.74126	8	182	1	143	15.1	10.74		34.938
High	P53814-6	' SMTN	Isoform B3 of Smoothelin OS=Homo sapiens OX=9606 GN=SMTN	0	60.755	22.97872	16	57	16	940	101.7	8.78		0.867
High	Q9Y4P3	' TBL2	Transducin beta-like protein 2 OS=Homo sapiens OX=9606 GN=TBL2	0	60.706	42.72931	16	44	16	447	49.8	9.44		2.535
High	Q8IY81	' FTSJ3	pre-rRNA processing protein FTSJ3 OS=Homo sapiens OX=9606 GN=	0	60.637	25.38371	15	38	15	847	96.5	8.4		1.31
High	Q9UJS0-2	' SLC25A13	Isoform 2 of Calcium-binding mitochondrial carrier protein Aralar2	0	60.609	38.16568	18	56	13	676	74.3	8.62		2.162
High	P61586	' RHOA	Transforming protein RhoA OS=Homo sapiens OX=9606 GN=RHOA	0	60.604	72.02073	12	74	4	193	21.8	6.1		16.013
High	Q9Y6M1	' IGF2BP2	Insulin-like growth factor 2 mRNA-binding protein 2 OS=Homo sapie	0	60.559	33.55593	15	42	13	599	66.1	8.46		1.865
High	P98175-5	' RBM10	Isoform 5 of RNA-binding protein 10 OS=Homo sapiens OX=9606 GN	0	60.506	26.53266	19	38	18	995	110.3	6.28		1.184
High	Q15436	' SEC23A	Protein transport protein Sec23A OS=Homo sapiens OX=9606 GN=S	0	60.383	27.5817	16	55	13	765	86.1	7.08		1.683
High	O94905	' ERLIN2	Erlin-2 OS=Homo sapiens OX=9606 GN=ERLIN2 PE=1 SV=1	0	60.284	49.85251	15	56	13	339	37.8	5.62		3.217
High	Q92888-3	' ARHGEF1	Isoform 3 of Rho guanine nucleotide exchange factor 1 OS=Homo sa	0	60.023	22.65372	16	29	16	927	104	5.62		1.031
High	Q13409-2	' DYNC1I2	Isoform 2B of Cytoplasmic dynein 1 intermediate chain 2 OS=Homo	0	59.987	35.12658	13	35	13	632	70.6	5.24	Phospho	2.433
High	Q8N1F7	' NUP93	Nuclear pore complex protein Nup93 OS=Homo sapiens OX=9606 G	0	59.986	33.21123	23	53	23	819	93.4	5.72		1.36
High	P20810-6	' CAST	Isoform 6 of Calpastatin OS=Homo sapiens OX=9606 GN=CAST	0	59.715	36.40961	14	23	14	791	84.9	5.36		1.462
High	P61204	' ARF3	ADP-ribosylation factor 3 OS=Homo sapiens OX=9606 GN=ARF3 PE=	0	59.681	54.69613	9	88	4	181	20.6	7.43		13.678
High	O94979-8	' SEC31A	Isoform 8 of Protein transport protein Sec31A OS=Homo sapiens OX	0	59.664	16.54501	18	48	18	1233	134.4	6.73		1.283
High	Q93077	' HIST1H2AC	Histone H2A type 1-C OS=Homo sapiens OX=9606 GN=HIST1H2AC P	0	59.448	68.46154	9	248	1	130	14.1	11.05		137.95
High	Q8IUE6	' HIST2H2AB	Histone H2A type 2-B OS=Homo sapiens OX=9606 GN=HIST2H2AB P	0	59.288	70	9	140	3	130	14	10.89		99
High	Q15323	' KRT31	Keratin, type I cuticular Ha1 OS=Homo sapiens OX=9606 GN=KRT31	0	59.195	45.67308	16	51	5	416	47.2	4.88		3.262
High	Q9NUU7	' DDX19A	ATP-dependent RNA helicase DDX19A OS=Homo sapiens OX=9606	0	59.125	43.72385	16	41	3	478	53.9	6.58		1.955
High	P55060	' CSE1L	Exportin-2 OS=Homo sapiens OX=9606 GN=CSE1L PE=1 SV=3	0	59.119	24.09887	19	66	19	971	110.3	5.77		1.212
High	Q8N1N4	' KRT78	Keratin, type II cytoskeletal 78 OS=Homo sapiens OX=9606 GN=KRT	0	59.057	35.38462	17	70	14	520	56.8	6.02		2.472
High	P30153	' PPP2R1A	Serine/threonine-protein phosphatase 2A 65 kDa regulatory subuni	0	59.033	31.06961	14	47	14	589	65.3	5.11		1.285
High	O00148	' DDX39A	ATP-dependent RNA helicase DDX39A OS=Homo sapiens OX=9606	0	58.998	38.1733	13	56	3	427	49.1	5.68		2.728
High	Q8TEB7-2	' RNF128	Isoform 2 of E3 ubiquitin-protein ligase RNF128 OS=Homo sapiens	0	58.936	20.14925	7	33	2	402	44.6	6.1		9
High	O60610	' DIAPH1	Protein diaphanous homolog 1 OS=Homo sapiens OX=9606 GN=DIA	0	58.791	16.3522	19	43	19	1272	141.3	5.41		0.913
High	P33121	' ACSL1	Long-chain-fatty-acid--CoA ligase 1 OS=Homo sapiens OX=9606 GN=	0	58.715	32.37822	18	43	16	698	77.9	7.15		2.07
High	P00338-3	' LDHA	Isoform 3 of L-lactate dehydrogenase A chain OS=Homo sapiens OX	0	58.663	53.73961	16	50	15	361	39.8	8.43		2.728
High	Q9NRR4	' DROSHA	Ribonuclease 3 OS=Homo sapiens OX=9606 GN=DROSHA PE=1 SV=2	0	58.489	16.01164	17	43	17	1374	159.2	7.87		0.81
High	Q15942	' ZYX	Zyxin OS=Homo sapiens OX=9606 GN=ZYX PE=1 SV=1	0	58.304	34.61538	13	67	13	572	61.2	6.67		2.675
High	P31946	' YWHAB	14-3-3 protein beta/alpha OS=Homo sapiens OX=9606 GN=YWHAB	0	58.077	58.94309	13	76	7	246	28.1	4.83		6.627
High	Q01085-2	' TIAL1	Isoform 2 of Nucleolysin TIAR OS=Homo sapiens OX=9606 GN=TIAL1	0	58.046	36.73469	12	56	8	392	43.4	7.99		3.16
High	P49790	' NUP153	Nuclear pore complex protein Nup153 OS=Homo sapiens OX=9606	0	58.034	20.61017	20	56	20	1475	153.8	8.73		0.968
High	O14579	' COPE	Coatomer subunit epsilon OS=Homo sapiens OX=9606 GN=COPE PE	0	58.03	60.71429	10	44	10	308	34.5	5.12		3.281
High	Q14103	' HNRNPD	Heterogeneous nuclear ribonucleoprotein D0 OS=Homo sapiens OX	0	58.001	34.08451	13	60	12	355	38.4	7.81		6.356
High	Q9UQ80	' PA2G4	Proliferation-associated protein 2G4 OS=Homo sapiens OX=9606 GN	0	57.897	42.63959	15	77	15	394	43.8	6.55		3.786
High	P37268	' FDFT1	Squalene synthase OS=Homo sapiens OX=9606 GN=FDFT1 PE=1 SV=	0	57.807	41.00719	15	40	15	417	48.1	6.54		3.642
High	P62841	' RPS15	40S ribosomal protein S15 OS=Homo sapiens OX=9606 GN=RPS15 P	0	57.712	54.48276	6	53	6	145	17	10.39		36.276
High	P17812	' CTPS1	CTP synthase 1 OS=Homo sapiens OX=9606 GN=CTPS1 PE=1 SV=2	0	57.665	34.51777	15	45	13	591	66.6	6.46		1.683
High	Q12788	' TBL3	Transducin beta-like protein 3 OS=Homo sapiens OX=9606 GN=TBL3	0	57.637	26.36139	16	29	16	808	89	6.9		1.121
High	Q9H223	' EHD4	EH domain-containing protein 4 OS=Homo sapiens OX=9606 GN=EH	0	57.571	29.02033	14	60	13	541	61.1	6.76		1.581
High	Q9P2R6	' RERE	Arginine-glutamic acid dipeptide repeats protein OS=Homo sapiens	0	57.406	17.75223	18	45	18	1566	172.3	7.69		1.154
High	P17096	' HMGA1	High mobility group protein HMG-I/HMG-Y OS=Homo sapiens OX=9	0	57.295	56.07477	9	79	4	107	11.7	10.32		145.78
High	P84103	' SRSF3	Serine/arginine-rich splicing factor 3 OS=Homo sapiens OX=9606 GN	0	57.216	40.2439	9	117	8	164	19.3	11.65		24.119
High	P31947	' SFN	14-3-3 protein sigma OS=Homo sapiens OX=9606 GN=SFN PE=1 SV=	0	57.179	55.24194	13	45	9	248	27.8	4.74		5.813
High	O43175	' PHGDH	D-3-phosphoglycerate dehydrogenase OS=Homo sapiens OX=9606	0	57.106	29.64353	12	55	12	533	56.6	6.71		2.455
High	P62081	' RPS7	40S ribosomal protein S7 OS=Homo sapiens OX=9606 GN=RPS7 PE=	0	57.073	64.94845	14	85	14	194	22.1	10.1		34.112
High	P16152	' CBR1	Carbonyl reductase [NADPH] 1 OS=Homo sapiens OX=9606 GN=CBR	0	57.061	52.34657	12	37	12	277	30.4	8.32		4.995
High	O75131	' CPNE3	Copine-3 OS=Homo sapiens OX=9606 GN=CPNE3 PE=1 SV=1	0	56.97	30.54004	15	51	15	537	60.1	5.85		2.162
High	O14979	' HNRNPDL	Heterogeneous nuclear ribonucleoprotein D-like OS=Homo sapiens	0	56.957	21.66667	12	65	11	420	46.4	9.57		2.831
High	O94925-3	' GLS	Isoform 3 of Glutaminase kidney isoform, mitochondrial OS=Homo s	0	56.956	36.28763	14	43	14	598	65.4	7.88		1.61
High	P62258	' YWHAE	14-3-3 protein epsilon OS=Homo sapiens OX=9606 GN=YWHAE PE=	0	56.934	46.66667	12	68	10	255	29.2	4.74		4.995
High	Q15417	' CNN3	Calponin-3 OS=Homo sapiens OX=9606 GN=CNN3 PE=1 SV=1	0	56.872	50.15198	10	36	9	329	36.4	6.05		4.456
High	P05198	' EIF2S1	Eukaryotic translation initiation factor 2 subunit 1 OS=Homo sapien	0	56.859	57.14286	15	51	15	315	36.1	5.08		4.926
High	Q9H4M9	' EHD1	EH domain-containing protein 1 OS=Homo sapiens OX=9606 GN=EH	0	56.697	36.14232	14	28	12	534	60.6	6.83		1.683
High	P62888	' RPL30	60S ribosomal protein L30 OS=Homo sapiens OX=9606 GN=RPL30 P	0	56.599	66.95652	8	96	8	115	12.8	9.63		55.234
High	O15230	' LAMA5	Laminin subunit alpha-5 OS=Homo sapiens OX=9606 GN=LAMA5 PE	0	56.549	8.064953	19	32	19	3695	399.5	7.02		0.246
High	P39656	' DDOST	Dolichyl-diphosphooligosaccharide--protein glycosyltransferase 48	0	56.514	41.66667	12	56	12	456	50.8	6.55		2.631
High	P48047	' ATP5O	ATP synthase subunit O, mitochondrial OS=Homo sapiens OX=9606	0	56.513	62.9108	10	44	10	213	23.3	9.96		5.105
High	Q86UW9-	' DTX2	Isoform 2 of Probable E3 ubiquitin-protein ligase DTX2 OS=Homo sa	0	56.299	36.34783	11	40	1	575	62.3	7.77		2.775
High	P59998-3	' ARPC4	Isoform 3 of Actin-related protein 2/3 complex subunit 4 OS=Homo	0	56.274	51.3369	9	57	9	187	21.6	8.59		9
High	P01130	' LDLR	Low-density lipoprotein receptor OS=Homo sapiens OX=9606 GN=L	0	56.187	21.62791	15	36	15	860	95.3	5.05		1.054
High	P19013	' KRT4	Keratin, type II cytoskeletal 4 OS=Homo sapiens OX=9606 GN=KRT4	0	56.066	23.78277	14	306	5	534	57.3	6.61		2.511
High	Q02790	' FKBP4	Peptidyl-prolyl cis-trans isomerase FKBP4 OS=Homo sapiens OX=96	0	56.048	42.2658	16	58	16	459	51.8	5.43		2.808
High	P23743	' DGKA	Diacylglycerol kinase alpha OS=Homo sapiens OX=9606 GN=DGKA P	0	56.027	25.71429	15	40	15	735	82.6	6.73		1.085
High	Q9H3U1	' UNC45A	Protein unc-45 homolog A OS=Homo sapiens OX=9606 GN=UNC45A	0	55.723	18.96186	14	52	14	944	103	6.07		0.73
High	P39060	' COL18A1	Collagen alpha-1(XVIII) chain OS=Homo sapiens OX=9606 GN=COL1	0	55.682	12.37172	14	31	14	1754	178.1	6.01		0.566
High	Q8TDN6	' BRIX1	Ribosome biogenesis protein BRX1 homolog OS=Homo sapiens OX=	0	55.624	45.60907	12	29	12	353	41.4	9.92		4.623
High	P25205-2	' MCM3	Isoform 2 of DNA replication licensing factor MCM3 OS=Homo sapie	0	55.315	23.56389	16	52	16	853	95.8	5.77		0.9
High	P18031	' PTPN1	Tyrosine-protein phosphatase non-receptor type 1 OS=Homo sapier	0	55.292	33.7931	12	46	12	435	49.9	6.27		1.829
High	Q9UHB9	' SRP68	Signal recognition particle subunit SRP68 OS=Homo sapiens OX=960	0	55.228	29.98405	16	39	16	627	70.7	8.56		1.356
High	P29728	' OAS2	2'-5'-oligoadenylate synthase 2 OS=Homo sapiens OX=9606 GN=OA	0	55.223	21.83588	15	33	15	719	82.4	8.25		1.818
High	Q15437	' SEC23B	Protein transport protein Sec23B OS=Homo sapiens OX=9606 GN=S	0	55.21	22.0339	13	51	10	767	86.4	6.89		1.322
High	P62995	' TRA2B	Transformer-2 protein homolog beta OS=Homo sapiens OX=9606 G	0	55.209	40.97222	11	67	8	288	33.6	11.25	Phospho	27.48
High	Q15397	' PUM3	Pumilio homolog 3 OS=Homo sapiens OX=9606 GN=PUM3 PE=1 SV=	0	55.16	27.16049	13	20	13	648	73.5	9.64		1.154
High	O75494-3	' SRSF10	Isoform 3 of Serine/arginine-rich splicing factor 10 OS=Homo sapien	0	54.999	54.64481	11	59	11	183	22.2	10.33		13.678
High	Q92597	' NDRG1	Protein NDRG1 OS=Homo sapiens OX=9606 GN=NDRG1 PE=1 SV=1	0	54.98	42.63959	10	43	10	394	42.8	5.82		4.08
High	Q5T9A4	' ATAD3B	ATPase family AAA domain-containing protein 3B OS=Homo sapiens	0	54.895	27.62346	17	67	3	648	72.5	9.2		1.703
High	O95985	' TOP3B	DNA topoisomerase 3-beta-1 OS=Homo sapiens OX=9606 GN=TOP3	0	54.768	24.01392	17	29	17	862	96.6	8.12		1.188
High	P07951-2	' TPM2	Isoform 2 of Tropomyosin beta chain OS=Homo sapiens OX=9606 G	0	54.725	33.4507	12	52	1	284	33	4.67		3.489
High	P39019	' RPS19	40S ribosomal protein S19 OS=Homo sapiens OX=9606 GN=RPS19 P	0	54.601	70.34483	15	83	15	145	16.1	10.32		30.623
High	P30520	' ADSS	Adenylosuccinate synthetase isozyme 2 OS=Homo sapiens OX=9606	0	54.579	25.87719	10	45	10	456	50.1	6.55		1.913
High	Q8IYM9	' TRIM22	E3 ubiquitin-protein ligase TRIM22 OS=Homo sapiens OX=9606 GN=	0	54.339	36.54618	14	34	14	498	56.9	7.72		1.738
High	P62269	' RPS18	40S ribosomal protein S18 OS=Homo sapiens OX=9606 GN=RPS18 P	0	54.299	53.28947	13	141	13	152	17.7	10.99		25.102
High	Q99729-3	' HNRNPAB	Isoform 3 of Heterogeneous nuclear ribonucleoprotein A/B OS=Hom	0	54.284	44.5614	13	80	12	285	30.6	7.91		9
High	P31040	' SDHA	Succinate dehydrogenase [ubiquinone] flavoprotein subunit, mitoch	0	54.189	25.90361	13	39	13	664	72.6	7.39		1.39
High	P34897	' SHMT2	Serine hydroxymethyltransferase, mitochondrial OS=Homo sapiens	0	53.935	31.34921	12	29	11	504	56	8.53		1.412
High	Q9BWD1	' ACAT2	Acetyl-CoA acetyltransferase, cytosolic OS=Homo sapiens OX=9606	0	53.927	37.53149	11	58	11	397	41.3	6.92		3.329
High	P02790	' HPX	Hemopexin OS=Homo sapiens OX=9606 GN=HPX PE=1 SV=2	0	53.849	38.31169	12	58	12	462	51.6	7.02		2.455
High	P62191	' PSMC1	26S proteasome regulatory subunit 4 OS=Homo sapiens OX=9606 G	0	53.841	45.90909	15	36	14	440	49.2	6.21		2.433
High	Q8N556-2	' AFAP1	Isoform 2 of Actin filament-associated protein 1 OS=Homo sapiens	0	53.601	20.14742	17	54	17	814	89.7	8.57	Phospho	1.748
High	P46782	' RPS5	40S ribosomal protein S5 OS=Homo sapiens OX=9606 GN=RPS5 PE=	0	53.469	50	13	75	13	204	22.9	9.72		24.119
High	Q6YHK3	' CD109	CD109 antigen OS=Homo sapiens OX=9606 GN=CD109 PE=1 SV=2	0	53.443	17.43945	19	43	19	1445	161.6	5.85		0.752
High	P60953	' CDC42	Cell division control protein 42 homolog OS=Homo sapiens OX=9606	0	53.44	62.82723	10	54	9	191	21.2	6.55		18.953
High	Q9UNX4	' WDR3	WD repeat-containing protein 3 OS=Homo sapiens OX=9606 GN=W	0	53.438	20.36055	14	23	14	943	106	6.64		0.833
High	Q53GQ0	' HSD17B12	Very-long-chain 3-oxoacyl-CoA reductase OS=Homo sapiens OX=960	0	53.373	44.55128	11	47	11	312	34.3	9.32		4.08
High	Q27J81	' INF2	Inverted formin-2 OS=Homo sapiens OX=9606 GN=INF2 PE=1 SV=2	0	53.317	17.3739	16	55	16	1249	135.5	5.38		0.812
High	Q00325-2	' SLC25A3	Isoform B of Phosphate carrier protein, mitochondrial OS=Homo sap	0	53.258	38.50416	15	80	15	361	39.9	9.36		5.449
High	Q16181	' SEPT7	Septin-7 OS=Homo sapiens OX=9606 GN=SEPT7 PE=1 SV=2	0	53.22	46.22426	15	49	14	437	50.6	8.63		3.262
High	P62879	' GNB2	Guanine nucleotide-binding protein G(I)/G(S)/G(T) subunit beta-2 O	0	53.141	43.52941	12	44	6	340	37.3	6		6.499
High	Q96HS1	' PGAM5	Serine/threonine-protein phosphatase PGAM5, mitochondrial OS=H	0	53.109	46.7128	11	56	11	289	32	8.68		3.833
High	Q8TCB0	' IFI44	Interferon-induced protein 44 OS=Homo sapiens OX=9606 GN=IFI44	0	53.053	37.38739	16	32	16	444	50.5	6.89		2.398
High	O15042	' U2SURP	U2 snRNP-associated SURP motif-containing protein OS=Homo sapi	0	52.922	19.72789	15	52	15	1029	118.2	8.47		1.154
High	Q01518	' CAP1	Adenylyl cyclase-associated protein 1 OS=Homo sapiens OX=9606 G	0	52.835	31.78947	14	43	14	475	51.9	8.06		2.054
High	O00622	' CYR61	Protein CYR61 OS=Homo sapiens OX=9606 GN=CYR61 PE=1 SV=1	0	52.807	48.29396	14	35	14	381	42	8.21		2.433
High	P84085	' ARF5	ADP-ribosylation factor 5 OS=Homo sapiens OX=9606 GN=ARF5 PE=	0	52.779	56.66667	10	87	5	180	20.5	6.79		9
High	Q13242	' SRSF9	Serine/arginine-rich splicing factor 9 OS=Homo sapiens OX=9606 GN	0	52.751	50.22624	12	79	12	221	25.5	8.65	Phospho	10.548
High	Q71DI3	' HIST2H3A	Histone H3.2 OS=Homo sapiens OX=9606 GN=HIST2H3A PE=1 SV=3	0	52.712	64.70588	9	92	1	136	15.4	11.27		70.969
High	Q01081	' U2AF1	Splicing factor U2AF 35 kDa subunit OS=Homo sapiens OX=9606 GN	0	52.674	32.91667	8	79	8	240	27.9	8.81		11.115
High	Q14692	' BMS1	Ribosome biogenesis protein BMS1 homolog OS=Homo sapiens OX=	0	52.664	15.36661	14	20	14	1282	145.7	6.44		0.618
High	Q9NR56	' MBNL1	Muscleblind-like protein 1 OS=Homo sapiens OX=9606 GN=MBNL1	0	52.549	29.89691	8	50	2	388	41.8	8.9		5.31
High	P78347	' GTF2I	General transcription factor II-I OS=Homo sapiens OX=9606 GN=GTF	0	52.523	22.94589	18	40	18	998	112.3	6.39		0.951
High	P01891	' HLA-A	HLA class I histocompatibility antigen, A-68 alpha chain OS=Homo sa	0	52.497	41.36986	11	38	3	365	40.9	6.7		3.806
High	P09382	' LGALS1	Galectin-1 OS=Homo sapiens OX=9606 GN=LGALS1 PE=1 SV=2	0	52.451	77.77778	8	118	8	135	14.7	5.5		45.416
High	P05141	' SLC25A5	ADP/ATP translocase 2 OS=Homo sapiens OX=9606 GN=SLC25A5 PE	0	52.381	45.30201	15	99	5	298	32.8	9.69		4.78
High	Q13724	' MOGS	Mannosyl-oligosaccharide glucosidase OS=Homo sapiens OX=9606	0	52.362	23.29749	13	29	13	837	91.9	8.9		0.945
High	Q9BYX7	' POTEKP	Putative beta-actin-like protein 3 OS=Homo sapiens OX=9606 GN=P	0	52.34	13.06667	5	385	1	375	42	6.33		2.325
High	P49419	' ALDH7A1	Alpha-aminoadipic semialdehyde dehydrogenase OS=Homo sapiens	0	52.24	29.12801	10	38	10	539	58.5	7.99		1.512
High	P08621	' SNRNP70	U1 small nuclear ribonucleoprotein 70 kDa OS=Homo sapiens OX=9	0	52.088	38.2151	14	62	14	437	51.5	9.94		5.31
High	Q9UBU9	' NXF1	Nuclear RNA export factor 1 OS=Homo sapiens OX=9606 GN=NXF1	0	52.016	28.75606	15	31	15	619	70.1	8.51		1.966
High	P60228	' EIF3E	Eukaryotic translation initiation factor 3 subunit E OS=Homo sapien	0	52.015	39.10112	14	46	14	445	52.2	6.04		1.943
High	Q14847	' LASP1	LIM and SH3 domain protein 1 OS=Homo sapiens OX=9606 GN=LAS	0	52.005	56.32184	14	100	13	261	29.7	7.05		7.799
High	Q13573	' SNW1	SNW domain-containing protein 1 OS=Homo sapiens OX=9606 GN=	0	51.931	32.83582	13	27	13	536	61.5	9.52		1.762
High	Q9UMR2	' DDX19B	ATP-dependent RNA helicase DDX19B OS=Homo sapiens OX=9606	0	51.855	36.11691	14	37	1	479	53.9	6.3		1.738
High	O43242	' PSMD3	26S proteasome non-ATPase regulatory subunit 3 OS=Homo sapien	0	51.833	32.02247	13	44	13	534	60.9	8.44		1.154
High	Q6PL18	' ATAD2	ATPase family AAA domain-containing protein 2 OS=Homo sapiens	0	51.723	18.34532	17	35	17	1390	158.5	6.32		0.685
High	Q9P206-2	' KIAA1522	Isoform 2 of Uncharacterized protein KIAA1522 OS=Homo sapiens	0	51.578	18.46435	14	37	14	1094	113	10.02		0.968
High	P17301	' ITGA2	Integrin alpha-2 OS=Homo sapiens OX=9606 GN=ITGA2 PE=1 SV=1	0	51.553	14.30991	13	30	13	1181	129.2	5.31		0.723
High	P18085	' ARF4	ADP-ribosylation factor 4 OS=Homo sapiens OX=9606 GN=ARF4 PE=	0	51.477	60.55556	9	74	5	180	20.5	7.14		7.377
High	P51532	' SMARCA4	Transcription activator BRG1 OS=Homo sapiens OX=9606 GN=SMAR	0	51.378	12.14329	17	33	9	1647	184.5	7.88		0.679
High	P62249	' RPS16	40S ribosomal protein S16 OS=Homo sapiens OX=9606 GN=RPS16 P	0	51.326	63.0137	13	109	13	146	16.4	10.21		20.544
High	Q9H9G7	' AGO3	Protein argonaute-3 OS=Homo sapiens OX=9606 GN=AGO3 PE=1 SV	0	51.305	22.90698	14	49	6	860	97.3	9.11		0.759
High	Q14525	' KRT33B	Keratin, type I cuticular Ha3-II OS=Homo sapiens OX=9606 GN=KRT3	0	51.176	40.59406	14	49	2	404	46.2	4.84		2.433
High	Q99873	' PRMT1	Protein arginine N-methyltransferase 1 OS=Homo sapiens OX=9606	0	51.122	41.50943	13	48	13	371	42.4	5.35		5.31
High	P26373	' RPL13	60S ribosomal protein L13 OS=Homo sapiens OX=9606 GN=RPL13 P	0	51.067	45.0237	13	99	13	211	24.2	11.65		10.788
High	Q14203	' DCTN1	Dynactin subunit 1 OS=Homo sapiens OX=9606 GN=DCTN1 PE=1 SV	0	51.042	13.61502	13	33	13	1278	141.6	5.81		0.565
High	O43896	' KIF1C	Kinesin-like protein KIF1C OS=Homo sapiens OX=9606 GN=KIF1C PE	0	50.957	19.12965	16	43	12	1103	122.9	6.9		0.656
High	P46781	' RPS9	40S ribosomal protein S9 OS=Homo sapiens OX=9606 GN=RPS9 PE=	0	50.948	58.24742	17	125	17	194	22.6	10.65		21.758
High	P15170-3	' GSPT1	Isoform 3 of Eukaryotic peptide chain release factor GTP-binding su	0	50.844	27.47253	15	40	15	637	68.7	5.33		2.054
High	P14324	' FDPS	Farnesyl pyrophosphate synthase OS=Homo sapiens OX=9606 GN=F	0	50.838	23.86635	7	43	7	419	48.2	6.15		2.008
High	Q96T23	' RSF1	Remodeling and spacing factor 1 OS=Homo sapiens OX=9606 GN=R	0	50.82	14.43442	17	39	17	1441	163.7	5.01		0.631
High	Q12792	' TWF1	Twinfilin-1 OS=Homo sapiens OX=9606 GN=TWF1 PE=1 SV=3	0	50.775	33.71429	12	24	12	350	40.3	6.96		3.062
High	Q13595	' TRA2A	Transformer-2 protein homolog alpha OS=Homo sapiens OX=9606	0	50.758	46.09929	12	52	9	282	32.7	11.27	Phospho	9
High	Q96CS3	' FAF2	FAS-associated factor 2 OS=Homo sapiens OX=9606 GN=FAF2 PE=1	0	50.662	31.68539	10	36	10	445	52.6	5.62		1.783
High	Q96GQ7	' DDX27	Probable ATP-dependent RNA helicase DDX27 OS=Homo sapiens OX	0	50.645	23.61809	18	40	18	796	89.8	9.28		1.512
High	P08134	' RHOC	Rho-related GTP-binding protein RhoC OS=Homo sapiens OX=9606	0	50.524	65.80311	10	66	2	193	22	6.58		11.328
High	P07737	' PFN1	Profilin-1 OS=Homo sapiens OX=9606 GN=PFN1 PE=1 SV=2	0	50.413	72.14286	11	94	11	140	15	8.27		26.826
High	P62277	' RPS13	40S ribosomal protein S13 OS=Homo sapiens OX=9606 GN=RPS13 P	0	50.412	50.99338	11	83	11	151	17.2	10.54		17.738
High	P10321	' HLA-C	HLA class I histocompatibility antigen, Cw-7 alpha chain OS=Homo s	0	50.398	33.06011	9	37	2	366	40.6	6.04		4.456
High	Q5JTH9	' RRP12	RRP12-like protein OS=Homo sapiens OX=9606 GN=RRP12 PE=1 SV=	0	50.37	18.27294	17	29	17	1297	143.6	8.75		0.674
High	Q9H9B4	' SFXN1	Sideroflexin-1 OS=Homo sapiens OX=9606 GN=SFXN1 PE=1 SV=4	0	50.35	50.31056	11	35	9	322	35.6	9.07		3.467
High	P46783	' RPS10	40S ribosomal protein S10 OS=Homo sapiens OX=9606 GN=RPS10 P	0	50.304	58.78788	11	74	11	165	18.9	10.15		10.938
High	P11498	' PC	Pyruvate carboxylase, mitochondrial OS=Homo sapiens OX=9606 GN	0	50.253	19.94907	17	34	17	1178	129.6	6.84		0.71
High	Q13427	' PPIG	Peptidyl-prolyl cis-trans isomerase G OS=Homo sapiens OX=9606 GN	0	49.955	20.02653	14	51	14	754	88.6	10.29		2.282
High	P07339	' CTSD	Cathepsin D OS=Homo sapiens OX=9606 GN=CTSD PE=1 SV=1	0	49.816	33.98058	12	57	12	412	44.5	6.54		3.062
High	P12004	' PCNA	Proliferating cell nuclear antigen OS=Homo sapiens OX=9606 GN=P	0	49.76	50.19157	11	65	11	261	28.8	4.69		3.833
High	P61019	' RAB2A	Ras-related protein Rab-2A OS=Homo sapiens OX=9606 GN=RAB2A	0	49.677	49.0566	8	44	8	212	23.5	6.54		4.412
High	P38606	' ATP6V1A	V-type proton ATPase catalytic subunit A OS=Homo sapiens OX=960	0	49.608	31.11831	14	46	14	617	68.3	5.52		1.371
High	P62195	' PSMC5	26S proteasome regulatory subunit 8 OS=Homo sapiens OX=9606 G	0	49.599	38.42365	12	44	11	406	45.6	7.55		1.683
High	Q5JTV8	' TOR1AIP1	Torsin-1A-interacting protein 1 OS=Homo sapiens OX=9606 GN=TOR	0	49.422	28.64494	13	47	13	583	66.2	8.18		1.352
High	Q9BTC0	' DIDO1	Death-inducer obliterator 1 OS=Homo sapiens OX=9606 GN=DIDO1	0	49.422	9.866071	17	35	17	2240	243.7	7.88		0.336
High	Q5D862	' FLG2	Filaggrin-2 OS=Homo sapiens OX=9606 GN=FLG2 PE=1 SV=1	0	49.36	11.58511	13	42	13	2391	247.9	8.31		0.462
High	Q9UKM9	' RALY	RNA-binding protein Raly OS=Homo sapiens OX=9606 GN=RALY PE=	0	49.152	39.54248	13	50	13	306	32.4	9.17		4.012
High	Q9H2P0	' ADNP	Activity-dependent neuroprotector homeobox protein OS=Homo sa	0	49.132	20.41742	16	35	16	1102	123.5	7.34		0.645
High	Q96TA1	' FAM129B	Niban-like protein 1 OS=Homo sapiens OX=9606 GN=FAM129B PE=	0	49.033	18.09651	9	38	9	746	84.1	6.19		0.848
High	P13807	' GYS1	Glycogen [starch] synthase, muscle OS=Homo sapiens OX=9606 GN=	0	48.92	22.9308	13	36	13	737	83.7	6.18		1.154
High	Q92747	' ARPC1A	Actin-related protein 2/3 complex subunit 1A OS=Homo sapiens OX	0	48.864	45.13514	14	42	13	370	41.5	8.18		3.217
High	P00387-3	' CYB5R3	Isoform 3 of NADH-cytochrome b5 reductase 3 OS=Homo sapiens O	0	48.849	40.11976	9	32	9	334	38.2	6.9		2.981
High	Q14CN4	' KRT72	Keratin, type II cytoskeletal 72 OS=Homo sapiens OX=9606 GN=KRT	0	48.774	16.82975	11	89	3	511	55.8	6.89	Phospho	1.39
High	Q7Z794	' KRT77	Keratin, type II cytoskeletal 1b OS=Homo sapiens OX=9606 GN=KRT	0	48.727	20.76125	12	234	9	578	61.9	5.99		1.683
High	O75874	' IDH1	Isocitrate dehydrogenase [NADP] cytoplasmic OS=Homo sapiens OX	0	48.695	35.99034	12	46	11	414	46.6	7.01		1.929
High	P00352	' ALDH1A1	Retinal dehydrogenase 1 OS=Homo sapiens OX=9606 GN=ALDH1A1	0	48.587	21.35729	12	66	10	501	54.8	6.73		2.03
High	P62829	' RPL23	60S ribosomal protein L23 OS=Homo sapiens OX=9606 GN=RPL23 P	0	48.551	56.42857	9	76	9	140	14.9	10.51		11.589
High	P29317	' EPHA2	Ephrin type-A receptor 2 OS=Homo sapiens OX=9606 GN=EPHA2 PE	0	48.533	18.44262	14	35	13	976	108.2	6.23		0.743
High	Q9NZN4	' EHD2	EH domain-containing protein 2 OS=Homo sapiens OX=9606 GN=EH	0	48.532	28.72928	12	27	11	543	61.1	6.46		1.448
High	Q16822	' PCK2	Phosphoenolpyruvate carboxykinase [GTP], mitochondrial OS=Hom	0	48.478	26.875	14	31	14	640	70.7	7.62		1.239
High	Q93052	' LPP	Lipoma-preferred partner OS=Homo sapiens OX=9606 GN=LPP PE=1	0	48.459	30.88235	12	44	12	612	65.7	7.37		2.775
High	Q13418	' ILK	Integrin-linked protein kinase OS=Homo sapiens OX=9606 GN=ILK P	0	48.375	35.17699	14	51	14	452	51.4	8.07		2.433
High	P62910	' RPL32	60S ribosomal protein L32 OS=Homo sapiens OX=9606 GN=RPL32 P	0	48.364	40.74074	8	52	8	135	15.9	11.33		30.623
High	P68431	' HIST1H3A	Histone H3.1 OS=Homo sapiens OX=9606 GN=HIST1H3A PE=1 SV=2	0	48.353	64.70588	9	87	1	136	15.4	11.12		50.795
High	P62280	' RPS11	40S ribosomal protein S11 OS=Homo sapiens OX=9606 GN=RPS11 P	0	48.321	48.73418	11	93	11	158	18.4	10.3		24.119
High	Q02241	' KIF23	Kinesin-like protein KIF23 OS=Homo sapiens OX=9606 GN=KIF23 PE	0	48.219	16.66667	13	33	13	960	110	8.51		0.743
High	Q96AG4	' LRRC59	Leucine-rich repeat-containing protein 59 OS=Homo sapiens OX=96	0	48.216	45.27687	11	68	11	307	34.9	9.57		3.642
High	P07384	' CAPN1	Calpain-1 catalytic subunit OS=Homo sapiens OX=9606 GN=CAPN1	0	48.118	23.80952	15	48	15	714	81.8	5.67		1.276
High	O00139-2	' KIF2A	Isoform 2 of Kinesin-like protein KIF2A OS=Homo sapiens OX=9606	0	48.039	26.9697	13	25	13	660	75	6.4		1.116
High	Q8TCD5	' NT5C	5'(3')-deoxyribonucleotidase, cytosolic type OS=Homo sapiens OX=	0	48.003	52.23881	7	34	7	201	23.4	6.64		4.623
High	O60762	' DPM1	Dolichol-phosphate mannosyltransferase subunit 1 OS=Homo sapie	0	47.693	50	11	53	11	260	29.6	9.57		3.642
High	P32322-3	' PYCR1	Isoform 3 of Pyrroline-5-carboxylate reductase 1, mitochondrial OS=	0	47.656	41.32948	10	22	9	346	36	7.61		3.642
High	Q92769	' HDAC2	Histone deacetylase 2 OS=Homo sapiens OX=9606 GN=HDAC2 PE=1	0	47.542	35.2459	11	44	7	488	55.3	5.91	Phospho	2.433
High	Q13155	' AIMP2	Aminoacyl tRNA synthase complex-interacting multifunctional prote	0	47.526	51.25	11	53	11	320	35.3	8.22		3.467
High	Q9UNQ2	' DIMT1	Probable dimethyladenosine transferase OS=Homo sapiens OX=960	0	47.477	41.21406	11	29	11	313	35.2	9.99		2.325
High	P29401-2	' TKT	Isoform 2 of Transketolase OS=Homo sapiens OX=9606 GN=TKT	0	47.415	33.75594	15	47	15	631	68.8	7.52		1.543
High	Q15459	' SF3A1	Splicing factor 3A subunit 1 OS=Homo sapiens OX=9606 GN=SF3A1	0	47.366	26.1034	16	36	16	793	88.8	5.22		1.404
High	Q99798	' ACO2	Aconitate hydratase, mitochondrial OS=Homo sapiens OX=9606 GN	0	47.299	23.71795	13	39	13	780	85.4	7.61		1.075
High	P16989-2	' YBX3	Isoform 2 of Y-box-binding protein 3 OS=Homo sapiens OX=9606 GN	0	47.211	44.88449	9	51	1	303	31.9	9.66		5.31
High	P28300	' LOX	Protein-lysine 6-oxidase OS=Homo sapiens OX=9606 GN=LOX PE=1	0	47.042	43.40528	11	25	11	417	46.9	8.09		3.16
High	Q15390	' MTFR1	Mitochondrial fission regulator 1 OS=Homo sapiens OX=9606 GN=M	0	46.989	36.33634	11	31	11	333	37	9.01		4.817
High	P84243	' H3F3A	Histone H3.3 OS=Homo sapiens OX=9606 GN=H3F3A PE=1 SV=2	0	46.726	64.70588	10	84	3	136	15.3	11.27		50.795
High	Q92621	' NUP205	Nuclear pore complex protein Nup205 OS=Homo sapiens OX=9606	0	46.7	8.996024	14	24	14	2012	227.8	6.19		0.373
High	P11908-2	' PRPS2	Isoform 2 of Ribose-phosphate pyrophosphokinase 2 OS=Homo sap	0	46.636	37.6947	8	36	3	321	35	6.46		2.36
High	P60891	' PRPS1	Ribose-phosphate pyrophosphokinase 1 OS=Homo sapiens OX=960	0	46.632	38.05031	9	39	4	318	34.8	6.98		2.162
High	Q14527	' HLTF	Helicase-like transcription factor OS=Homo sapiens OX=9606 GN=H	0	46.578	19.22696	14	21	14	1009	113.9	8.6		0.682
High	Q9BXJ9	' NAA15	N-alpha-acetyltransferase 15, NatA auxiliary subunit OS=Homo sapi	0	46.542	16.9746	14	36	14	866	101.2	7.42		0.746
High	Q9H6S0	' YTHDC2	3'-5' RNA helicase YTHDC2 OS=Homo sapiens OX=9606 GN=YTHDC2	0	46.501	12.72727	15	23	15	1430	160.1	8.4		0.456
High	Q9UBQ7	' GRHPR	Glyoxylate reductase/hydroxypyruvate reductase OS=Homo sapiens	0	46.393	40.2439	8	34	8	328	35.6	7.39		2.162
High	P13929	' ENO3	Beta-enolase OS=Homo sapiens OX=9606 GN=ENO3 PE=1 SV=5	0	46.338	23.96313	7	53	3	434	47	7.71		0.887
High	Q9Y3F4-2	' STRAP	Isoform 2 of Serine-threonine kinase receptor-associated protein O	0	46.133	35.81267	10	31	10	363	39.8	5.31		1.873
High	P84098	' RPL19	60S ribosomal protein L19 OS=Homo sapiens OX=9606 GN=RPL19 P	0	46.098	32.14286	9	75	9	196	23.5	11.47		11.915
High	Q9UBS4	' DNAJB11	DnaJ homolog subfamily B member 11 OS=Homo sapiens OX=9606	0	46.008	32.68156	11	43	11	358	40.5	6.18		2.899
High	Q9H0U4	' RAB1B	Ras-related protein Rab-1B OS=Homo sapiens OX=9606 GN=RAB1B	0	45.98	60.69652	10	58	3	201	22.2	5.73		3.437
High	Q5SW79	' CEP170	Centrosomal protein of 170 kDa OS=Homo sapiens OX=9606 GN=CE	0	45.933	14.33081	14	31	14	1584	175.2	7.11		0.389
High	Q15269	' PWP2	Periodic tryptophan protein 2 homolog OS=Homo sapiens OX=9606	0	45.921	17.73667	13	25	13	919	102.4	6.15		0.905
High	P43034	' PAFAH1B1	Platelet-activating factor acetylhydrolase IB subunit alpha OS=Hom	0	45.914	38.78049	13	48	12	410	46.6	7.37		2.594
High	Q9BPX5	' ARPC5L	Actin-related protein 2/3 complex subunit 5-like protein OS=Homo	0	45.858	58.82353	6	53	5	153	16.9	6.6		5.813
High	P35250	' RFC2	Replication factor C subunit 2 OS=Homo sapiens OX=9606 GN=RFC2	0	45.495	36.72316	10	31	10	354	39.1	6.44		1.754
High	P20073	' ANXA7	Annexin A7 OS=Homo sapiens OX=9606 GN=ANXA7 PE=1 SV=3	0	45.469	33.40164	14	29	14	488	52.7	5.68		3.062
High	P50995	' ANXA11	Annexin A11 OS=Homo sapiens OX=9606 GN=ANXA11 PE=1 SV=1	0	45.411	27.72277	12	36	12	505	54.4	7.65		3.329
High	Q96DI7	' SNRNP40	U5 small nuclear ribonucleoprotein 40 kDa protein OS=Homo sapier	0	45.38	44.2577	9	34	9	357	39.3	8.1		3.467
High	Q8TD16-2	' BICD2	Isoform 2 of Protein bicaudal D homolog 2 OS=Homo sapiens OX=96	0	45.32	27.25146	16	21	16	855	96.7	5.44		0.861
High	O14980	' XPO1	Exportin-1 OS=Homo sapiens OX=9606 GN=XPO1 PE=1 SV=1	0	45.308	14.75257	12	24	12	1071	123.3	6.06		0.597

High	O75475	' PSIP1	PC4 and SFRS1-interacting protein OS=Homo sapiens OX=9606 GN=	0	45.233	26.79245	13	32	13	530	60.1	9.13		1.39
High	P12236	' SLC25A6	ADP/ATP translocase 3 OS=Homo sapiens OX=9606 GN=SLC25A6 PE	0	45.202	44.96644	14	79	4	298	32.8	9.74		3.329
High	Q9BSJ8-2	' ESYT1	Isoform 2 of Extended synaptotagmin-1 OS=Homo sapiens OX=9606	0	45.14	12.92639	11	34	11	1114	123.9	5.78		0.548
High	Q7L576	' CYFIP1	Cytoplasmic FMR1-interacting protein 1 OS=Homo sapiens OX=9606	0	45.138	16.20112	17	31	17	1253	145.1	6.9		0.674
High	P62750	' RPL23A	60S ribosomal protein L23a OS=Homo sapiens OX=9606 GN=RPL23A	0	45.097	51.92308	12	92	12	156	17.7	10.45		34.938
High	P67870	' CSNK2B	Casein kinase II subunit beta OS=Homo sapiens OX=9606 GN=CSNK2	0	45.001	46.97674	8	22	8	215	24.9	5.55	Phospho	7.254
High	P28370	' SMARCA1	Probable global transcription activator SNF2L1 OS=Homo sapiens O	0	44.977	13.66224	16	46	2	1054	122.5	8.09		0.719
High	Q01831	' XPC	DNA repair protein complementing XP-C cells OS=Homo sapiens OX	0	44.967	19.04255	11	17	11	940	105.9	8.9		0.8
High	O60832	' DKC1	H/ACA ribonucleoprotein complex subunit DKC1 OS=Homo sapiens	0	44.92	27.23735	11	26	11	514	57.6	9.42		1.106
High	Q8WXX5	' DNAJC9	DnaJ homolog subfamily C member 9 OS=Homo sapiens OX=9606 G	0	44.846	43.46154	12	35	12	260	29.9	5.73		3.642
High	P28838	' LAP3	Cytosol aminopeptidase OS=Homo sapiens OX=9606 GN=LAP3 PE=1	0	44.843	35.06744	13	35	13	519	56.1	7.93		1.297
High	Q9NX63	' CHCHD3	MICOS complex subunit MIC19 OS=Homo sapiens OX=9606 GN=CHC	0	44.809	50.22026	13	52	13	227	26.1	8.28		4.275
High	Q9Y5J1	' UTP18	U3 small nucleolar RNA-associated protein 18 homolog OS=Homo sa	0	44.791	34.71223	12	20	12	556	62	8.76		1.512
High	P07195	' LDHB	L-lactate dehydrogenase B chain OS=Homo sapiens OX=9606 GN=LD	0	44.678	36.52695	11	34	10	334	36.6	6.05		2.728
High	O00303	' EIF3F	Eukaryotic translation initiation factor 3 subunit F OS=Homo sapiens	0	44.615	35.85434	11	41	11	357	37.5	5.45		4.08
High	P35659	' DEK	Protein DEK OS=Homo sapiens OX=9606 GN=DEK PE=1 SV=1	0	44.57	35.2	12	59	12	375	42.6	8.56		2.481
High	Q9BY77	' POLDIP3	Polymerase delta-interacting protein 3 OS=Homo sapiens OX=9606	0	44.528	38.95487	11	53	11	421	46.1	9.99		1.471
High	P16989	' YBX3	Y-box-binding protein 3 OS=Homo sapiens OX=9606 GN=YBX3 PE=1	0	44.474	36.55914	9	51	1	372	40.1	9.77		2.981
High	Q96L91	' EP400	E1A-binding protein p400 OS=Homo sapiens OX=9606 GN=EP400 PE	0	44.379	9.623299	15	34	15	3159	343.3	9.19		0.35
High	P62244	' RPS15A	40S ribosomal protein S15a OS=Homo sapiens OX=9606 GN=RPS15A	0	44.295	73.07692	10	89	10	130	14.8	10.13		14.199
High	Q96QD9	' FYTTD1	UAP56-interacting factor OS=Homo sapiens OX=9606 GN=FYTTD1 P	0	44.208	46.85535	12	28	12	318	35.8	11.78		2.981
High	P23588	' EIF4B	Eukaryotic translation initiation factor 4B OS=Homo sapiens OX=960	0	44.026	27.49591	16	34	16	611	69.1	5.73		1.955
High	P35998	' PSMC2	26S proteasome regulatory subunit 7 OS=Homo sapiens OX=9606 G	0	43.903	38.10624	12	36	12	433	48.6	5.95		1.438
High	O95684	' FGFR1OP	FGFR1 oncogene partner OS=Homo sapiens OX=9606 GN=FGFR1OP	0	43.598	37.34336	11	19	11	399	43	4.81		4.275
High	Q9Y2Z4	' YARS2	Tyrosine--tRNA ligase, mitochondrial OS=Homo sapiens OX=9606 GN	0	43.596	26.83438	9	18	9	477	53.2	8.98		1.326
[illegible]	[illegible]	[illegible]	[illegible]	[illegible]	[illegible]	[illegible]	[illegible]	[illegible]	[illegible]	[illegible]	[illegible]	[illegible]	[illegible]	[illegible]

[illegible]

High	P37802-2	' TAGLN2	Isoform 2 of Transgelin-2 OS=Homo sapiens OX=9606 GN=TAGLN2	0	30.763	52.72727	10	35	10	220	24.4	8.25		3.642	10672331.3	22351661.6	9405278.56	13521754.1	14.36	10.14	11.3	10.62	6	4	5	5
High	P61513	' RPL37A	60S ribosomal protein L37a OS=Homo sapiens OX=9606 GN=RPL37A	0	30.743	51.08696	5	47	5	92	10.3	10.43		13.678	85723802.8	91952487.1	24167084.8	16636105.1	22.13	15.72	5.28	5.22	4	4	3	3
High	Q71UM5	' RPS27L	40S ribosomal protein S27-like OS=Homo sapiens OX=9606 GN=RPS	0	30.666	40.47619	5	38	2	84	9.5	9.45		38.811	37348029.2	44612388.6	25105489.3	21699163.6	20.9	15.44	10.57	11.14	5	4	3	3
High	O60231	' DHX16	Pre-mRNA-splicing factor ATP-dependent RNA helicase DHX16 OS=H	0	30.598	12.00768	10	22	9	1041	119.2	6.8		0.487	5347776.05	4647807.69			16.38	6.93	1.8	1.76	8	3	1	1
High	P80723	' BASP1	Brain acid soluble protein 1 OS=Homo sapiens OX=9606 GN=BASP1	0	30.589	66.51982	8	22	8	227	22.7	4.63		4.878	8853553.78	5048350.63			6.69	2.37			5	2		
High	O43809	' NUDT21	Cleavage and polyadenylation specificity factor subunit 5 OS=Homo	0	30.531	48.89868	8	30	8	227	26.2	8.82		2.652	10071577.4	8305654.15	10018128.3	5833877.16	4.21	9.74	11.47	4.62	3	3	4	2
High	P62333	' PSMC6	26S proteasome regulatory subunit 10B OS=Homo sapiens OX=9606	0	30.522	30.07712	10	22	10	389	44.1	7.49		1.346	5434939.08	7252302.75	1760942.13	3592390.47	9.15	7.77	0	2.68	6	3	1	2
High	O94906	' PRPF6	Pre-mRNA-processing factor 6 OS=Homo sapiens OX=9606 GN=PRPI	0	30.407	13.49628	11	41	11	941	106.9	8.25		0.476	5368306.25	5707806.76	3438942.24	3636145.23	9.64	12.26	7.11	7.09	6	6	5	3
High	Q8N0Y7	' PGAM4	Probable phosphoglycerate mutase 4 OS=Homo sapiens OX=9606 G	0	30.392	22.44094	5	38	1	254	28.8	6.65		2.981	43979537.9	33137257.3	4725351	4795773.38	20	19.96	9.01	14.25	5	4	4	4
High	O94880	' PHF14	PHD finger protein 14 OS=Homo sapiens OX=9606 GN=PHF14 PE=1	0	30.319	11.5991	8	15	8	888	100	5.33		0.468	4810532.38	3447519.31			3.78	3.8			3	3		
High	O95758-4	' PTBP3	Isoform 4 of Polypyrimidine tract-binding protein 3 OS=Homo sapie	0	30.316	15.5914	6	23	4	558	60.4	9.19		0.957	22452748.9	17331103.9			13.11	14.56		1.72	5	3		2
High	O14936	' CASK	Peripheral plasma membrane protein CASK OS=Homo sapiens OX=9	0	30.299	12.74298	9	25	9	926	105.1	6.43		0.429	2192490.61	4326568.13	4036587.61	6186826.21	4.27	4.35	8.85	13.54	3	2	5	6
High	P0C0S5	' H2AFZ	Histone H2A.Z OS=Homo sapiens OX=9606 GN=H2AFZ PE=1 SV=2	0	30.229	31.25	5	148	3	128	13.5	10.58		20.544	2044280804	891850337	298407633	120372747	73	79.05	37.94	18.05	4	5	5	4
High	O75396	' SEC22B	Vesicle-trafficking protein SEC22b OS=Homo sapiens OX=9606 GN=S	0	30.183	38.60465	7	31	7	215	24.6	6.92		2.162	7699298.34	10202145.8	9601181.2	14243801.6	8.6	12	7.6	14.9	4	4	4	6
High	P52926	' HMGA2	High mobility group protein HMGI-C OS=Homo sapiens OX=9606 GN	0	30.174	48.62385	6	33	6	109	11.8	10.62		24.119	190397850	173102318	2829843.88		25.78	21.85	0		6	5	1	
High	P20290-2	' BTF3	Isoform 2 of Transcription factor BTF3 OS=Homo sapiens OX=9606 C	0	30.093	75.92593	8	24	8	162	17.7	7.5		9		2876660.29		3150158.46		3.34		2.4		4		2
High	Q8N5F7	' NKAP	NF-kappa-B-activating protein OS=Homo sapiens OX=9606 GN=NKA	0	30.058	19.51807	7	23	7	415	47.1	10.11		2.36	22728571.8	10926621.3	7056085.29	13272074.6	8.68	4.6	9.88	8.54	2	2	4	5
High	Q66PJ3	' ARL6IP4	ADP-ribosylation factor-like protein 6-interacting protein 4 OS=Hom	0	30.005	16.62708	7	33	7	421	44.9	10.93		1.31	21842916.5	13247747.9	19256501.3	21145841.6	6.27	10.81	19.77	15.93	2	4	7	5
High	P12830	' CDH1	Cadherin-1 OS=Homo sapiens OX=9606 GN=CDH1 PE=1 SV=3	0	30	15.87302	10	14	10	882	97.4	4.73		0.833						2.77				1		
High	Q96FQ6	' S100A16	Protein S100-A16 OS=Homo sapiens OX=9606 GN=S100A16 PE=1 SV	0	29.978	66.99029	6	19	6	103	11.8	6.79		20.544	16655373.2	23106747.8		2873389.25	13.05	10.87		3.11	4	4		1
High	P29590	' PML	Protein PML OS=Homo sapiens OX=9606 GN=PML PE=1 SV=3	0	29.968	18.93424	11	26	11	882	97.5	6.21		0.598	3599133	2364424.92	4197283.86	6555706.05	2.34	1.88	6.37	9.04	1	4	4	4
High	Q5JWF2	' GNAS	Guanine nucleotide-binding protein G(s) subunit alpha isoforms XLa	0	29.879	12.24687	10	48	9	1037	111	5.03		0.983	9125154.13	5230602.31	4604155.8	12822408.9	9.33	5.13	8.55	21.39	6	3	6	8
High	Q13823	' GNL2	Nucleolar GTP-binding protein 2 OS=Homo sapiens OX=9606 GN=GI	0	29.831	14.36389	9	19	9	731	83.6	9.25		0.658	9559925.59	7024394.25			5.73	12.95			3	6		
High	Q9UGP8	' SEC63	Translocation protein SEC63 homolog OS=Homo sapiens OX=9606 G	0	29.811	14.47368	9	21	9	760	87.9	5.31		0.73	7458349.75	5739723.41	4229121.25		7.64	8.64	0		3	5	2	
High	Q05519	' SRSF11	Serine/arginine-rich splicing factor 11 OS=Homo sapiens OX=9606 G	0	29.743	17.14876	6	40	6	484	53.5	10.52		1.581	9879791.22	15519629.3	19911173.7	22827178.6	2.18	6.48	13.59	13.02	2	4	5	5
High	P03989	' HLA-B	HLA class I histocompatibility antigen, B-27 alpha chain OS=Homo sa	0	29.704	26.51934	7	15	1	362	40.4	5.82		1.976				8797591.06			5.11	17.73			2	6
High	P60866-2	' RPS20	Isoform 2 of 40S ribosomal protein S20 OS=Homo sapiens OX=9606	0	29.678	30.98592	6	51	6	142	16	9.32		12.895	210461732	184857903	132918929	148032081	22.53	24.15	22.13	23.18	5	5	6	6
High	P02768	' ALB	Serum albumin OS=Homo sapiens OX=9606 GN=ALB PE=1 SV=2	0	29.592	16.91297	10	46	10	609	69.3	6.28		0.828	134296945	144367674	185751649	180734778	17.87	9.55	13.57	13.13	7	4	5	6
High	Q10570	' CPSF1	Cleavage and polyadenylation specificity factor subunit 1 OS=Homo	0	29.583	8.108108	10	17	10	1443	160.8	6.4		0.342	6439566.34	10822605.3			11.53	2.45			5	3		
High	Q9NZ01	' TECR	Very-long-chain enoyl-CoA reductase OS=Homo sapiens OX=9606 G	0	29.526	24.67532	8	29	8	308	36	9.45		2.162	22081834.9	27368255.3	10852449.4	13023515.1	11.49	11.36	2.87	12.77	4	6	3	5
High	Q4KMQ1	' TPRN	Taperin OS=Homo sapiens OX=9606 GN=TPRN PE=1 SV=2	0	29.426	22.36287	11	20	11	711	75.5	7.25		0.884	4897909.75		27101526	6475525.82	2.96		10.14	12.88	1		6	6
High	P10319	' HLA-B	HLA class I histocompatibility antigen, B-58 alpha chain OS=Homo sa	0	29.368	27.90055	7	18	1	362	40.3	6.3		1.818				3568193.81		2.82	2.31	19.72		1	1	6
High	Q96EP5	' DAZAP1	DAZ-associated protein 1 OS=Homo sapiens OX=9606 GN=DAZAP1 F	0	29.286	25.30713	6	20	6	407	43.4	8.56		2.162	15299742	26476848.2	16583760.9	16948907.3	4.1	10.16	10.19	9.35	1	3	3	4
High	P31483	' TIA1	Nucleolysin TIA-1 isoform p40 OS=Homo sapiens OX=9606 GN=TIA1	0	29.25	22.27979	8	32	4	386	42.9	7.74		1.512	5443001.63	5485601.63	9042963.08	5398042.75	12.54	10.05	12.87	8.66	6	4	6	3
High	P49189	' ALDH9A1	4-trimethylaminobutyraldehyde dehydrogenase OS=Homo sapiens (	0	29.214	19.83806	9	42	9	494	53.8	5.87		1.346	19770790.3	13954413.9	5296579.41	6259433.98	13.57	13.45	12.09	8.54	4	6	7	5
High	Q86X29	' LSR	Lipolysis-stimulated lipoprotein receptor OS=Homo sapiens OX=960	0	29.204	16.17874	8	22	8	649	71.4	7.97		0.778	5457847.28	6921265.39		3645233.6	9.05	13.65		6.71	4	6		5
High	Q9NRW3	' APOBEC3C	DNA dC->dU-editing enzyme APOBEC-3C OS=Homo sapiens OX=960	0	29.196	38.94737	5	10	5	190	22.8	7.59		2.162		26427470.8				13.17			4			
High	O94851	' MICAL2	[F-actin]-monooxygenase MICAL2 OS=Homo sapiens OX=9606 GN=I	0	29.184	11.121	11	19	9	1124	126.6	8.65		0.415			3869861	4515697.49			7.04	4.29			5	6
High	O00487	' PSMD14	26S proteasome non-ATPase regulatory subunit 14 OS=Homo sapie	0	29.159	32.58065	6	19	6	310	34.6	6.52		1.783	9664107.71	12223409.5	4584217.03	7984567.68	9.8	7.69	2.49	5.92	4	3	1	3
High	Q9NTK5	' OLA1	Obg-like ATPase 1 OS=Homo sapiens OX=9606 GN=OLA1 PE=1 SV=2	0	29.038	26.76768	8	23	8	396	44.7	7.81		1.031	11761525.8	11237986.6	3496788.44	5643411.62	12.16	10.78	2.56	11.63	4	3	1	4
High	P84090	' ERH	Enhancer of rudimentary homolog OS=Homo sapiens OX=9606 GN=	0	29.032	46.15385	6	48	6	104	12.3	5.92		25.827	486681816	403443099	99791230.7		27.93	24.29	5.96		5	5	2	
High	Q9Y5P6-2	' GMPPB	Isoform 2 of Mannose-1-phosphate guanyltransferase beta OS=Hon	0	29.017	26.35659	8	32	8	387	42.6	6.29		1.512	32793485.7	21955190	7959642.44	13039793.4	14.71	18.4	5.89	7.08	6	7	3	3
High	Q9UBM7	' DHCR7	7-dehydrocholesterol reductase OS=Homo sapiens OX=9606 GN=DH	0	28.976	18.52632	8	37	8	475	54.5	8.7	Phospho [	2.594	13634774.8	25806946	16270925.2	15348618.7	14.77	13.75	6.72	12.08	6	6	3	5
High	Q9H0H5	' RACGAP1	Rac GTPase-activating protein 1 OS=Homo sapiens OX=9606 GN=RA	0	28.958	21.67722	10	28	10	632	71	8.88		0.805	9119099.06	3431833.53	3005424.57	1346689.94	13.34	6.81	2.02	0	7	4	4	1
High	Q14651	' PLS1	Plastin-1 OS=Homo sapiens OX=9606 GN=PLS1 PE=1 SV=2	0	28.878	15.26232	7	23	3	629	70.2	5.41		0.668	9424466.07	10371323.7			12.13	11.71		0	5	5		1
High	P62851	' RPS25	40S ribosomal protein S25 OS=Homo sapiens OX=9606 GN=RPS25 P	0	28.85	30.4	5	71	5	125	13.7	10.11		30.623	465222539	301516050	103233345	112715433	29.12	24.44	18.37	16.07	5	5	5	4
High	Q99848	' EBNA1BP2	Probable rRNA-processing protein EBP2 OS=Homo sapiens OX=9606	0	28.832	29.08497	8	19	8	306	34.8	10.1		1.783	16167792.8	12977647			6.88	10.34	0		4	4	1	
High	O14530	' TXNDC9	Thioredoxin domain-containing protein 9 OS=Homo sapiens OX=960	0	28.827	25.22124	6	24	6	226	26.5	5.88		2.162	14997484.8	17767008	6952243.24	8881720.04	9.24	9.74	7.48	8.75	3	3	5	3
High	Q15050	' RRS1	Ribosome biogenesis regulatory protein homolog OS=Homo sapiens	0	28.797	28.49315	8	12	8	365	41.2	10.7		1.154	6504977.81	5371629.6			3.56	5.05			1	3		
High	Q5T4S7-2	' UBR4	Isoform 2 of E3 ubiquitin-protein ligase UBR4 OS=Homo sapiens OX-	0	28.794	3.312206	10	15	10	5204	575.6	6.01		0.086	3750560.94	3531825.07			12.06	10.56			5	6		
High	O00566	' MPHOSPH10	U3 small nucleolar ribonucleoprotein protein MPP10 OS=Homo sapi	0	28.761	19.08957	10	13	10	681	78.8	4.86		0.833		5334700.58				3.33			3			
High	P17980	' PSMC3	26S proteasome regulatory subunit 6A OS=Homo sapiens OX=9606 (	0	28.684	33.02961	9	22	9	439	49.2	5.24		1.154	8053881.13	6388159	5092283.09	8963422.35	1.8	3.1	4.85	9.59	1	2	3	3
High	Q13620	' CUL4B	Cullin-4B OS=Homo sapiens OX=9606 GN=CUL4B PE=1 SV=4	0	28.636	13.03399	12	26	6	913	103.9	7.37		0.53	8859214.96	2092410.44			8.3	6.22	0		7	5	1	
High	P30566	' ADSL	Adenylosuccinate lyase OS=Homo sapiens OX=9606 GN=ADSL PE=1	0	28.619	23.76033	8	18	8	484	54.9	7.11		0.951	4001689.29	2793135.59	3110216.91	4089414.79	4.6	3.4	4.99	8.41	3	3	2	4
High	Q04828	' AKR1C1	Aldo-keto reductase family 1 member C1 OS=Homo sapiens OX=960	0	28.546	32.19814	9	19	9	323	36.8	7.88		1.565	6152803.34	6444631.42	6366364.28	5453310.19	3.6	2.05	6.08	6.35	2	2	3	3
High	Q16795	' NDUFA9	NADH dehydrogenase [ubiquinone] 1 alpha subcomplex subunit 9, r	0	28.539	28.11671	8	23	8	377	42.5	9.8		0.931	10358780.9	7636767.91	6203186.71		12.16	13.83	6.76		4	5	4	
High	P20336	' RAB3A	Ras-related protein Rab-3A OS=Homo sapiens OX=9606 GN=RAB3A	0	28.474	41.81818	8	35	6	220	25	5.03		2.162	15413302.2	17989110.8		5126306.31	16.89	14.81	2.29	6.29	6	5	1	3
High	P52735	' VAV2	Guanine nucleotide exchange factor VAV2 OS=Homo sapiens OX=96	0	28.468	10.47836	6	18	6	878	101.2	7.08		0.302	6398278.61	4755608.56	3527605.03		7.39	13.78	2.38		3	5	2	
High	P42766	' RPL35	60S ribosomal protein L35 OS=Homo sapiens OX=9606 GN=RPL35 P	0	28.464	34.95935	6	41	6	123	14.5	11.05		20.544	323896399	222105803	145635151	60057294.8	12.42	17.8	5.31	10.9	3	5	1	3
High	P63173	' RPL38	60S ribosomal protein L38 OS=Homo sapiens OX=9606 GN=RPL38 P	0	28.432	52.85714	7	55	7	70	8.2	10.1		463.159	45599850.3	90783666.1	50425143.9	61648081.1	36	24.72	17.39	17.68	6	5	5	6
[illegible]																										

High	Q15061	' WDR43	WD repeat-containing protein 43 OS=Homo sapiens OX=9606 GN=W	0	20.666	14.62334	7	[illegible]
High	O75569	' PRKRA	Interferon-inducible double-stranded RNA-dependent protein kinas	0	20.659	24.28115	5	[illegible]
High	Q04446	' GBE1	1,4-alpha-glucan-branching enzyme OS=Homo sapiens OX=9606 GN	0	20.644	13.24786	8	[illegible]
High	O43818	' RRP9	U3 small nucleolar RNA-interacting protein 2 OS=Homo sapiens OX=	0	20.614	17.05263	7	[illegible]
High	O15031	' PLXNB2	Plexin-B2 OS=Homo sapiens OX=9606 GN=PLXNB2 PE=1 SV=3	0	20.61	5.005441	6	[illegible]
High	P62070-4	' RRAS2	Isoform 4 of Ras-related protein R-Ras2 OS=Homo sapiens OX=9606	0	20.569	28.57143	5	[illegible]
High	Q12929	' EPS8	Epidermal growth factor receptor kinase substrate 8 OS=Homo sapi	0	20.559	15.08516	7	[illegible]
High	Q07352	' ZFP36L1	mRNA decay activator protein ZFP36L1 OS=Homo sapiens OX=9606	0	20.546	23.9645	7	[illegible]
High	P11166	' SLC2A1	Solute carrier family 2, facilitated glucose transporter member 1 OS	0	20.533	7.113821	4	[illegible]
High	P51665	' PSMD7	26S proteasome non-ATPase regulatory subunit 7 OS=Homo sapiens	0	20.5	28.08642	7	[illegible]
High	Q92766-2	' RREB1	Isoform 2 of Ras-responsive element-binding protein 1 OS=Homo sa	0	20.499	7.405281	8	[illegible]
High	Q86VI3	' IQGAP3	Ras GTPase-activating-like protein IQGAP3 OS=Homo sapiens OX=96	0	20.426	3.556101	5	[illegible]
High	Q96AE4-2	' FUBP1	Isoform 2 of Far upstream element-binding protein 1 OS=Homo sap	0	20.354	11.79173	6	[illegible]
High	P49760	' CLK2	Dual specificity protein kinase CLK2 OS=Homo sapiens OX=9606 GN=	0	20.349	11.02204	5	[illegible]
High	P30041	' PRDX6	Peroxiredoxin-6 OS=Homo sapiens OX=9606 GN=PRDX6 PE=1 SV=3	0	20.341	36.16071	7	[illegible]
High	Q9H5Z1	' DHX35	Probable ATP-dependent RNA helicase DHX35 OS=Homo sapiens OX	0	20.334	13.08677	7	[illegible]
High	O60763-2	' USO1	Isoform 2 of General vesicular transport factor p115 OS=Homo sapi	0	20.312	7.605344	6	[illegible]
High	Q99653	' CHP1	Calcineurin B homologous protein 1 OS=Homo sapiens OX=9606 GN	0	20.241	36.92308	6	[illegible]
High	Q9NQZ2	' UTP3	Something about silencing protein 10 OS=Homo sapiens OX=9606 G	0	20.236	20.04175	6	[illegible]
High	Q13043	' STK4	Serine/threonine-protein kinase 4 OS=Homo sapiens OX=9606 GN=S	0	20.224	18.48049	7	[illegible]
High	P57088	' TMEM33	Transmembrane protein 33 OS=Homo sapiens OX=9606 GN=TMEM3	0	20.192	23.48178	6	[illegible]
High	Q9Y5K5	' UCHL5	Ubiquitin carboxyl-terminal hydrolase isozyme L5 OS=Homo sapiens	0	20.178	22.18845	7	[illegible]
High	Q9ULC4-3	' MCTS1	Isoform 3 of Malignant T-cell-amplified sequence 1 OS=Homo sapien	0	20.145	40.65934	5	[illegible]
High	Q15392	' DHCR24	Delta(24)-sterol reductase OS=Homo sapiens OX=9606 GN=DHCR24	0	20.141	12.9845	6	[illegible]
High	P52789	' HK2	Hexokinase-2 OS=Homo sapiens OX=9606 GN=HK2 PE=1 SV=2	0	20.096	8.833152	7	[illegible]
High	O95786	' DDX58	Probable ATP-dependent RNA helicase DDX58 OS=Homo sapiens OX	0	20.093	10.91892	8	[illegible]
High	Q96P63-2	' SERPINB12	Isoform 2 of Serpin B12 OS=Homo sapiens OX=9606 GN=SERPINB12	0	20.081	19.52941	7	[illegible]
High	Q9NP97	' DYNLRB1	Dynein light chain roadblock-type 1 OS=Homo sapiens OX=9606 GN	0	20.036	51.04167	3	[illegible]
High	Q969X5	' ERGIC1	Endoplasmic reticulum-Golgi intermediate compartment protein 1 O	0	20.021	26.55172	6	[illegible]
High	P07942	' LAMB1	Laminin subunit beta-1 OS=Homo sapiens OX=9606 GN=LAMB1 PE=	0	20.013	5.207167	7	[illegible]
High	Q8TDD1-2	' DDX54	Isoform 2 of ATP-dependent RNA helicase DDX54 OS=Homo sapiens	0	19.995	8.049887	6	[illegible]
High	P61081	' UBE2M	NEDD8-conjugating enzyme Ubc12 OS=Homo sapiens OX=9606 GN=	0	19.93	35.51913	6	[illegible]
High	P60981	' DSTN	Destrin OS=Homo sapiens OX=9606 GN=DSTN PE=1 SV=3	0	19.906	28.48485	5	[illegible]
High	Q68CZ2	' TNS3	Tensin-3 OS=Homo sapiens OX=9606 GN=TNS3 PE=1 SV=2	0	19.871	9.134948	9	[illegible]
High	Q8N3E9	' PLCD3	1-phosphatidylinositol 4,5-bisphosphate phosphodiesterase delta-3	0	19.865	13.68821	6	[illegible]
High	Q9Y2L1	' DIS3	Exosome complex exonuclease RRP44 OS=Homo sapiens OX=9606 G	0	19.856	8.768267	7	[illegible]
High	Q9BUH6	' PAXX	Protein PAXX OS=Homo sapiens OX=9606 GN=PAXX PE=1 SV=2	0	19.83	24.01961	4	[illegible]
High	Q13601	' KRR1	KRR1 small subunit processome component homolog OS=Homo sap	0	19.794	21.25984	6	[illegible]
High	O94874	' UFL1	E3 UFM1-protein ligase 1 OS=Homo sapiens OX=9606 GN=UFL1 PE=	0	19.787	10.20151	7	[illegible]
High	Q9UJU6	' DBNL	Drebrin-like protein OS=Homo sapiens OX=9606 GN=DBNL PE=1 SV=	0	19.776	21.62791	7	[illegible]
High	Q9GZR7	' DDX24	ATP-dependent RNA helicase DDX24 OS=Homo sapiens OX=9606 GN	0	19.77	8.731083	5	[illegible]
High	Q14166	' TTLL12	Tubulin--tyrosine ligase-like protein 12 OS=Homo sapiens OX=9606	0	19.74	10.09317	5	[illegible]
High	Q13619	' CUL4A	Cullin-4A OS=Homo sapiens OX=9606 GN=CUL4A PE=1 SV=3	0	19.732	11.46245	8	[illegible]
High	Q86Y07	' VRK2	Serine/threonine-protein kinase VRK2 OS=Homo sapiens OX=9606 G	0	19.697	18.50394	7	[illegible]
High	Q9BY32	' ITPA	Inosine triphosphate pyrophosphatase OS=Homo sapiens OX=9606	0	19.683	32.47423	5	[illegible]
High	Q9Y6A4	' CFAP20	Cilia- and flagella-associated protein 20 OS=Homo sapiens OX=9606	0	19.682	22.79793	5	[illegible]
High	O15427	' SLC16A3	Monocarboxylate transporter 4 OS=Homo sapiens OX=9606 GN=SLC	0	19.664	10.53763	5	[illegible]
High	Q9ULW0-	' TPX2	Isoform 2 of Targeting protein for Xklp2 OS=Homo sapiens OX=9606	0	19.579	10.47254	7	[illegible]
High	O15213	' WDR46	WD repeat-containing protein 46 OS=Homo sapiens OX=9606 GN=W	0	19.574	11.63934	6	[illegible]
High	Q15370-2	' ELOB	Isoform 2 of Elongin-B OS=Homo sapiens OX=9606 GN=ELOB	0	19.556	33.54037	4	[illegible]
High	Q9H078-2	' CLPB	Isoform 2 of Caseinolytic peptidase B protein homolog OS=Homo sa	0	19.549	10.78287	6	[illegible]
High	Q13185	' CBX3	Chromobox protein homolog 3 OS=Homo sapiens OX=9606 GN=CBX	0	19.516	23.49727	4	[illegible]
High	Q96RP9-2	' GFM1	Isoform 2 of Elongation factor G, mitochondrial OS=Homo sapiens C	0	19.51	10	7	[illegible]
High	P36776	' LONP1	Lon protease homolog, mitochondrial OS=Homo sapiens OX=9606 G	0	19.503	6.047967	5	[illegible]
High	P78406	' RAE1	mRNA export factor OS=Homo sapiens OX=9606 GN=RAE1 PE=1 SV=	0	19.497	22.28261	6	[illegible]
High	P17252	' PRKCA	Protein kinase C alpha type OS=Homo sapiens OX=9606 GN=PRKCA	0	19.487	13.83929	6	[illegible]
High	Q09028	' RBBP4	Histone-binding protein RBBP4 OS=Homo sapiens OX=9606 GN=RBE	0	19.478	18.35294	7	[illegible]
High	P62633	' CNBP	Cellular nucleic acid-binding protein OS=Homo sapiens OX=9606 GN	0	19.436	34.46328	5	[illegible]
High	P31937	' HIBADH	3-hydroxyisobutyrate dehydrogenase, mitochondrial OS=Homo sapi	0	19.428	26.78571	6	[illegible]
High	P62854	' RPS26	40S ribosomal protein S26 OS=Homo sapiens OX=9606 GN=RPS26 P	0	19.367	50.43478	5	[illegible]
High	P13987	' CD59	CD59 glycoprotein OS=Homo sapiens OX=9606 GN=CD59 PE=1 SV=1	0	19.319	25	4	[illegible]
High	Q9NYH9	' UTP6	U3 small nucleolar RNA-associated protein 6 homolog OS=Homo sa	0	19.292	15.41039	7	[illegible]
High	Q12996	' CSTF3	Cleavage stimulation factor subunit 3 OS=Homo sapiens OX=9606 G	0	19.224	12.5523	6	[illegible]
High	Q6P1J9	' CDC73	Parafibromin OS=Homo sapiens OX=9606 GN=CDC73 PE=1 SV=1	0	19.211	12.80603	5	[illegible]
High	Q9NXF1	' TEX10	Testis-expressed protein 10 OS=Homo sapiens OX=9606 GN=TEX10	0	19.204	8.18084	6	[illegible]
High	Q8N3V7-	' SYNPO	Isoform 2 of Synaptopodin OS=Homo sapiens OX=9606 GN=SYNPO	0	19.186	14.17497	9	[illegible]
High	Q969S3	' ZNF622	Zinc finger protein 622 OS=Homo sapiens OX=9606 GN=ZNF622 PE=	0	19.115	17.61006	6	[illegible]
High	O60678	' PRMT3	Protein arginine N-methyltransferase 3 OS=Homo sapiens OX=9606	0	19.095	13.55932	6	[illegible]
High	O14776	' TCERG1	Transcription elongation regulator 1 OS=Homo sapiens OX=9606 GN	0	19.068	6.284153	7	[illegible]
High	P13073	' COX4I1	Cytochrome c oxidase subunit 4 isoform 1, mitochondrial OS=Homo	0	18.986	30.76923	5	[illegible]
High	P53582	' METAP1	Methionine aminopeptidase 1 OS=Homo sapiens OX=9606 GN=MET	0	18.985	20.72539	5	[illegible]
High	P27797	' CALR	Calreticulin OS=Homo sapiens OX=9606 GN=CALR PE=1 SV=1	0	18.932	15.10791	5	[illegible]
High	Q9BUZ4	' TRAF4	TNF receptor-associated factor 4 OS=Homo sapiens OX=9606 GN=TI	0	18.92	14.89362	5	[illegible]
High	Q9BTV4	' TMEM43	Transmembrane protein 43 OS=Homo sapiens OX=9606 GN=TMEM4	0	18.904	15.25	4	[illegible]
High	O75746	' SLC25A12	Calcium-binding mitochondrial carrier protein Aralar1 OS=Homo sap	0	18.893	10.61947	7	[illegible]
High	Q9ULX3	' NOB1	RNA-binding protein NOB1 OS=Homo sapiens OX=9606 GN=NOB1 P	0	18.877	18.68932	7	[illegible]
High	Q9P0J7	' KCMF1	E3 ubiquitin-protein ligase KCMF1 OS=Homo sapiens OX=9606 GN=I	0	18.849	20.47244	5	[illegible]
High	Q9P2N5	' RBM27	RNA-binding protein 27 OS=Homo sapiens OX=9606 GN=RBM27 PE=	0	18.819	5.849057	5	[illegible]
High	Q8TEQ6	' GEMIN5	Gem-associated protein 5 OS=Homo sapiens OX=9606 GN=GEMIN5	0	18.766	8.289125	8	[illegible]
High	Q15181	' PPA1	Inorganic pyrophosphatase OS=Homo sapiens OX=9606 GN=PPA1 P	0	18.759	24.56747	5	[illegible]
High	Q7KZ85	' SUPT6H	Transcription elongation factor SPT6 OS=Homo sapiens OX=9606 GN	0	18.753	7.126304	7	[illegible]
High	Q92522	' H1FX	Histone H1x OS=Homo sapiens OX=9606 GN=H1FX PE=1 SV=1	0	18.751	23.00469	5	[illegible]
High	Q6UN15	' FIP1L1	Pre-mRNA 3'-end-processing factor FIP1 OS=Homo sapiens OX=9606	0	18.737	12.96296	5	[illegible]
High	P45974	' USP5	Ubiquitin carboxyl-terminal hydrolase 5 OS=Homo sapiens OX=9606	0	18.728	9.90676	7	[illegible]
High	Q01780	' EXOSC10	Exosome component 10 OS=Homo sapiens OX=9606 GN=EXOSC10 P	0	18.701	8.248588	5	[illegible]
High	Q05655	' PRKCD	Protein kinase C delta type OS=Homo sapiens OX=9606 GN=PRKCD	0	18.679	11.98225	6	[illegible]
High	P16519	' PCSK2	Neuroendocrine convertase 2 OS=Homo sapiens OX=9606 GN=PCSK	0	18.657	8.777429	4	[illegible]
High	Q13217	' DNAJC3	DnaJ homolog subfamily C member 3 OS=Homo sapiens OX=9606 G	0	18.631	20.2381	7	[illegible]
High	Q9NRL2	' BAZ1A	Bromodomain adjacent to zinc finger domain protein 1A OS=Homo	0	18.612	3.920308	6	[illegible]
High	O00411	' POLRMT	DNA-directed RNA polymerase, mitochondrial OS=Homo sapiens OX	0	18.6	5.772358	4	[illegible]
High	Q8TA86	' RP9	Retinitis pigmentosa 9 protein OS=Homo sapiens OX=9606 GN=RP9	0	18.585	25.79186	7	[illegible]
High	Q9Y2A7-2	' NCKAP1	Isoform 2 of Nck-associated protein 1 OS=Homo sapiens OX=9606 G	0	18.569	6.70194	6	[illegible]
High	P05109	' S100A8	Protein S100-A8 OS=Homo sapiens OX=9606 GN=S100A8 PE=1 SV=1	0	18.562	55.91398	6	[illegible]
High	Q9Y3U8	' RPL36	60S ribosomal protein L36 OS=Homo sapiens OX=9606 GN=RPL36 P	0	18.522	30.47619	5	[illegible]
High	Q6P1M0	' SLC27A4	Long-chain fatty acid transport protein 4 OS=Homo sapiens OX=960	0	18.519	12.44168	6	[illegible]
High	P54619-3	' PRKAG1	Isoform 3 of 5'-AMP-activated protein kinase subunit gamma-1 OS=	0	18.463	17.64706	5	[illegible]
High	P17706	' PTPN2	Tyrosine-protein phosphatase non-receptor type 2 OS=Homo sapien	0	18.443	20.48193	6	[illegible]
High	O14773	' TPP1	Tripeptidyl-peptidase 1 OS=Homo sapiens OX=9606 GN=TPP1 PE=1	0	18.427	11.01243	4	[illegible]
High	P61006	' RAB8A	Ras-related protein Rab-8A OS=Homo sapiens OX=9606 GN=RAB8A	0	18.414	27.53623	6	[illegible]
High	P51570-2	' GALK1	Isoform 2 of Galactokinase OS=Homo sapiens OX=9606 GN=GALK1	0	18.388	19.19431	6	[illegible]
High	Q13557-1	' CAMK2D	Isoform Delta 11 of Calcium/calmodulin-dependent protein kinase t	0	18.366	18.51145	7	[illegible]
High	P07954	' FH	Fumarate hydratase, mitochondrial OS=Homo sapiens OX=9606 GN=	0	18.352	10.78431	3	[illegible]
High	Q9Y512	' SAMM50	Sorting and assembly machinery component 50 homolog OS=Homo	0	18.348	17.91045	6	[illegible]
High	P28331-2	' NDUFS1	Isoform 2 of NADH-ubiquinone oxidoreductase 75 kDa subunit, mitc	0	18.323	12.14575	6	[illegible]
High	Q9Y3E5	' PTRH2	Peptidyl-tRNA hydrolase 2, mitochondrial OS=Homo sapiens OX=96	0	18.306	39.6648	4	[illegible]
High	P22392-2	' NME2	Isoform 3 of Nucleoside diphosphate kinase B OS=Homo sapiens OX	0	18.289	42.69663	7	[illegible]
High	Q96S52	' PIGS	GPI transamidase component PIG-S OS=Homo sapiens OX=9606 GN	0	18.251	14.59459	5	[illegible]
High	O95373	' IPO7	Importin-7 OS=Homo sapiens OX=9606 GN=IPO7 PE=1 SV=1	0	18.246	7.514451	6	[illegible]
High	P11766	' ADH5	Alcohol dehydrogenase class-3 OS=Homo sapiens OX=9606 GN=ADH	0	18.241	19.51872	7	[illegible]
High	O60493	' SNX3	Sorting nexin-3 OS=Homo sapiens OX=9606 GN=SNX3 PE=1 SV=3	0	18.239	37.65432	4	[illegible]
High	P35052	' GPC1	Glypican-1 OS=Homo sapiens OX=9606 GN=GPC1 PE=1 SV=2	0	18.205	15.05376	6	[illegible]
High	P14678-3	' SNRPB	Isoform SM-B1 of Small nuclear ribonucleoprotein-associated prote	0	18.163	20.41522	6	[illegible]
High	Q13228-4	' SELENBP1	Isoform 4 of Methanethiol oxidase OS=Homo sapiens OX=9606 GN=	0	18.138	8.754864	4	[illegible]
High	Q9H7B2	' RPF2	Ribosome production factor 2 homolog OS=Homo sapiens OX=9606	0	18.134	21.56863	6	[illegible]
High	Q7RTV0	' PHF5A	PHD finger-like domain-containing protein 5A OS=Homo sapiens OX	0	18.131	60	6	[illegible]
High	P31151	' S100A7	Protein S100-A7 OS=Homo sapiens OX=9606 GN=S100A7 PE=1 SV=4	0	18.115	54.45545	5	[illegible]
High	P62987	' UBA52	Ubiquitin-60S ribosomal protein L40 OS=Homo sapiens OX=9606 GN	0	18.102	45.3125	5	[illegible]
High	Q8TDB6	' DTX3L	E3 ubiquitin-protein ligase DTX3L OS=Homo sapiens OX=9606 GN=D	0	18.08	10.81081	6	[illegible]
High	O94776	' MTA2	Metastasis-associated protein MTA2 OS=Homo sapiens OX=9606 GN	0	18.079	14.07186	7	[illegible]
High	P40261	' NNMT	Nicotinamide N-methyltransferase OS=Homo sapiens OX=9606 GN=	0	18.074	16.66667	4	[illegible]
High	P15559	' NQO1	NAD(P)H dehydrogenase [quinone] 1 OS=Homo sapiens OX=9606 G	0	18.071	20.80292	5	[illegible]
High	Q96D46	' NMD3	60S ribosomal export protein NMD3 OS=Homo sapiens OX=9606 GN	0	18.051	16.6998	6	[illegible]
High	Q9BVK6	' TMED9	Transmembrane emp24 domain-containing protein 9 OS=Homo sap	0	17.981	22.12766	5	[illegible]
High	Q13363	' CTBP1	C-terminal-binding protein 1 OS=Homo sapiens OX=9606 GN=CTBP1	0	17.979	18.40909	6	[illegible]
High	Q13057-2	' COASY	Isoform 2 of Bifunctional coenzyme A synthase OS=Homo sapiens O	0	17.977	13.99663	6	[illegible]
High	Q92930	' RAB8B	Ras-related protein Rab-8B OS=Homo sapiens OX=9606 GN=RAB8B	0	17.964	27.53623	6	[illegible]
High	Q9BV36-2	' MLPH	Isoform 2 of Melanophilin OS=Homo sapiens OX=9606 GN=MLPH	0	17.957	14.16084	6	[illegible]
High	Q12931	' TRAP1	Heat shock protein 75 kDa, mitochondrial OS=Homo sapiens OX=96	0	17.946	8.380682	4	[illegible]
High	Q53H96	' PYCR3	Pyrroline-5-carboxylate reductase 3 OS=Homo sapiens OX=9606 GN	0	17.883	21.53285	4	[illegible]
High	Q86US8	' SMG6	Telomerase-binding protein EST1A OS=Homo sapiens OX=9606 GN=	0	17.879	5.073996	6	[illegible]
High	Q9C035	' TRIM5	Tripartite motif-containing protein 5 OS=Homo sapiens OX=9606 GN	0	17.865	12.7789	5	[illegible]
High	Q15435	' PPP1R7	Protein phosphatase 1 regulatory subunit 7 OS=Homo sapiens OX=9	0	17.861	14.44444	5	[illegible]
High	Q96EK6	' GNPNAT1	Glucosamine 6-phosphate N-acetyltransferase OS=Homo sapiens OX	0	17.842	31.52174	4	[illegible]
High	P09471-2	' GNAO1	Isoform Alpha-2 of Guanine nucleotide-binding protein G(o) subunit	0	17.823	13.27684	4	[illegible]
High	Q14318-2	' FKBP8	Isoform 2 of Peptidyl-prolyl cis-trans isomerase FKBP8 OS=Homo sa	0	17.801	16.46489	5	[illegible]
High	P31949	' S100A11	Protein S100-A11 OS=Homo sapiens OX=9606 GN=S100A11 PE=1 SV	0	17.792	42.85714	4	[illegible]
High	Q96IF1	' AJUBA	LIM domain-containing protein ajuba OS=Homo sapiens OX=9606 G	0	17.781	16.72862	6	[illegible]
High	P28290	' SSFA2	Sperm-specific antigen 2 OS=Homo sapiens OX=9606 GN=SSFA2 PE=	0	17.768	6.989674	7	[illegible]
High	Q9P2I0	' CPSF2	Cleavage and polyadenylation specificity factor subunit 2 OS=Homo	0	17.711	10.1023	7	[illegible]
High	P61201-2	' COPS2	Isoform 2 of COP9 signalosome complex subunit 2 OS=Homo sapien	0	17.71	14.44444	6	[illegible]
High	P17096-2	' HMGA1	Isoform HMG-Y of High mobility group protein HMG-I/HMG-Y OS=H	0	17.68	51.04167	6	[illegible]
High	P49207	' RPL34	60S ribosomal protein L34 OS=Homo sapiens OX=9606 GN=RPL34 P	0	17.678	36.75214	6	[illegible]
High	Q9NPQ8-	' RIC8A	Isoform 3 of Synembryn-A OS=Homo sapiens OX=9606 GN=RIC8A	0	17.631	11.54562	4	[illegible]
High	P51812	' RPS6KA3	Ribosomal protein S6 kinase alpha-3 OS=Homo sapiens OX=9606 GN	0	17.628	9.72973	6	[illegible]
High	Q9NVU7	' SDAD1	Protein SDA1 homolog OS=Homo sapiens OX=9606 GN=SDAD1 PE=1	0	17.627	12.5182	7	[illegible]
High	O43237	' DYNC1LI2	Cytoplasmic dynein 1 light intermediate chain 2 OS=Homo sapiens O	0	17.621	19.30894	6	[illegible]
High	Q8ND56	' LSM14A	Protein LSM14 homolog A OS=Homo sapiens OX=9606 GN=LSM14A	0	17.616	16.1987	6	[illegible]
High	P32926	' DSG3	Desmoglein-3 OS=Homo sapiens OX=9606 GN=DSG3 PE=1 SV=2	0	17.563	7.507508	5	[illegible]
High	P56545-2	' CTBP2	Isoform 2 of C-terminal-binding protein 2 OS=Homo sapiens OX=960	0	17.561	6.700508	5	[illegible]
High	Q9HC07	' TMEM165	Transmembrane protein 165 OS=Homo sapiens OX=9606 GN=TMEN	0	17.525	19.44444	3	[illegible]
High	Q9UHI6	' DDX20	Probable ATP-dependent RNA helicase DDX20 OS=Homo sapiens OX	0	17.515	9.587379	6	[illegible]
High	Q9P2T1-2	' GMPR2	Isoform 2 of GMP reductase 2 OS=Homo sapiens OX=9606 GN=GMF	0	17.506	19.12568	6	[illegible]
High	O15460	' P4HA2	Prolyl 4-hydroxylase subunit alpha-2 OS=Homo sapiens OX=9606 GN	0	17.498	16.63551	6	[illegible]
High	P24666-2	' ACP1	Isoform 2 of Low molecular weight phosphotyrosine protein phosph	0	17.483	27.21519	5	[illegible]
High	Q9Y6C9	' MTCH2	Mitochondrial carrier homolog 2 OS=Homo sapiens OX=9606 GN=M	0	17.427	22.11221	5	[illegible]
High	Q9H936	' SLC25A22	Mitochondrial glutamate carrier 1 OS=Homo sapiens OX=9606 GN=S	0	17.41	25.387	6	[illegible]
High	O14734	' ACOT8	Acyl-coenzyme A thioesterase 8 OS=Homo sapiens OX=9606 GN=AC	0	17.406	21.00313	4	[illegible]
High	Q13188-2	' STK3	Isoform 2 of Serine/threonine-protein kinase 3 OS=Homo sapiens O	0	17.369	17.53372	7	[illegible]
High	A2AJT9	' BCLAF3	BCLAF1 and THRAP3 family member 3 OS=Homo sapiens OX=9606 G	0	17.329	7.735584	5	[illegible]
High	Q9UNE7	' STUB1	E3 ubiquitin-protein ligase CHIP OS=Homo sapiens OX=9606 GN=STU	0	17.316	16.50165	5	[illegible]
High	O43709-3	' BUD23	Isoform 3 of Probable 18S rRNA (guanine-N(7))-methyltransferase O	0	17.285	17.78523	4	[illegible]
High	Q9Y266	' NUDC	Nuclear migration protein nudC OS=Homo sapiens OX=9606 GN=NU	0	17.256	25.07553	8	[illegible]
High	Q9BXS6	' NUSAP1	Nucleolar and spindle-associated protein 1 OS=Homo sapiens OX=96	0	17.246	12.92517	5	[illegible]
High	Q969V3	' NCLN	Nicalin OS=Homo sapiens OX=9606 GN=NCLN PE=1 SV=2	0	17.245	12.25577	5	[illegible]
High	P10515	' DLAT	Dihydrolipoyllysine-residue acetyltransferase component of pyruvat	0	17.204	15.14683	7	[illegible]
High	P11047	' LAMC1	Laminin subunit gamma-1 OS=Homo sapiens OX=9606 GN=LAMC1 P	0	17.185	6.712244	8	[illegible]
High	Q9P035	' HACD3	Very-long-chain (3R)-3-hydroxyacyl-CoA dehydratase 3 OS=Homo sa	0	17.182	12.98343	4	[illegible]
High	O95155-4	' UBE4B	Isoform 4 of Ubiquitin conjugation factor E4 B OS=Homo sapiens OX	0	17.123	9.386548	7	[illegible]
High	Q8WVV9	' HNRNPLL	Heterogeneous nuclear ribonucleoprotein L-like OS=Homo sapiens O	0	17.119	14.02214	6	[illegible]
High	Q05682	' CALD1	Caldesmon OS=Homo sapiens OX=9606 GN=CALD1 PE=1 SV=3	0	17.111	7.313997	5	[illegible]
High	Q9Y2X9	' ZNF281	Zinc finger protein 281 OS=Homo sapiens OX=9606 GN=ZNF281 PE=	0	17.111	16.31285	6	[illegible]
High	Q15025	' TNIP1	TNFAIP3-interacting protein 1 OS=Homo sapiens OX=9606 GN=TNIP	0	17.105	12.26415	7	[illegible]
High	P09234	' SNRPC	U1 small nuclear ribonucleoprotein C OS=Homo sapiens OX=9606 G	0	17.048	24.5283	3	[illegible]
High	P61421	' ATP6V0D1	V-type proton ATPase subunit d 1 OS=Homo sapiens OX=9606 GN=A	0	17.048	21.08262	6	[illegible]
High	Q9Y6E0	' STK24	Serine/threonine-protein kinase 24 OS=Homo sapiens OX=9606 GN=	0	17.029	10.15801	5	[illegible]
High	P55036	' PSMD4	26S proteasome non-ATPase regulatory subunit 4 OS=Homo sapiens	0	17.009	14.85411	5	[illegible]
High	O75964	' ATP5L	ATP synthase subunit g, mitochondrial OS=Homo sapiens OX=9606 O	0	16.978	46.60194	4	[illegible]
High	Q9Y316-3	' MEMO1	Isoform 3 of Protein MEMO1 OS=Homo sapiens OX=9606 GN=MEM	0	16.977	13.66667	4	[illegible]
High	Q9Y2T2	' AP3M1	AP-3 complex subunit mu-1 OS=Homo sapiens OX=9606 GN=AP3M1	0	16.938	13.63636	4	[illegible]
High	P62314	' SNRPD1	Small nuclear ribonucleoprotein Sm D1 OS=Homo sapiens OX=9606	0	16.889	54.62185	4	[illegible]
High	P09496-2	' CLTA	Isoform Non-brain of Clathrin light chain A OS=Homo sapiens OX=96	0	16.852	24.77064	5	[illegible]
High	Q8N766	' EMC1	ER membrane protein complex subunit 1 OS=Homo sapiens OX=960	0	16.826	6.747231	5	[illegible]
High	Q96IU4	' ABHD14B	Protein ABHD14B OS=Homo sapiens OX=9606 GN=ABHD14B PE=1 S	0	16.814	21.90476	3	[illegible]
High	O95571	' ETHE1	Persulfide dioxygenase ETHE1, mitochondrial OS=Homo sapiens OX=	0	16.81	22.83465	4	[illegible]
High	Q9Y223-2	' GNE	Isoform 2 of Bifunctional UDP-N-acetylglucosamine 2-epimerase/N-	0	16.802	9.561753	7	[illegible]
High	Q9UEY8	' ADD3	Gamma-adducin OS=Homo sapiens OX=9606 GN=ADD3 PE=1 SV=1	0	16.794	7.648725	5	[illegible]
High	Q6NXT2	' H3F3C	Histone H3.3C OS=Homo sapiens OX=9606 GN=H3F3C PE=1 SV=3	0	16.787	49.62963	6	[illegible]
High	P15531-2	' NME1	Isoform 2 of Nucleoside diphosphate kinase A OS=Homo sapiens OX	0	16.77	40.11299	6	[illegible]
High	Q86XP3	' DDX42	ATP-dependent RNA helicase DDX42 OS=Homo sapiens OX=9606 GN	0	16.728	7.675906	6	[illegible]
High	P04632	' CAPNS1	Calpain small subunit 1 OS=Homo sapiens OX=9606 GN=CAPNS1 PE=	0	16.725	25.74627	5	[illegible]
High	Q14571	' ITPR2	Inositol 1,4,5-trisphosphate receptor type 2 OS=Homo sapiens OX=9	0	16.71	2.887819	7	[illegible]
High	P13667	' PDIA4	Protein disulfide-isomerase A4 OS=Homo sapiens OX=9606 GN=PDIA	0	16.707	11.93798	5	[illegible]
High	O00764	' PDXK	Pyridoxal kinase OS=Homo sapiens OX=9606 GN=PDXK PE=1 SV=1	0	16.705	22.75641	5	[illegible]
High	Q9HD33	' MRPL47	39S ribosomal protein L47, mitochondrial OS=Homo sapiens OX=96	0	16.696	20.4	5	[illegible]
High	O14964	' HGS	Hepatocyte growth factor-regulated tyrosine kinase substrate OS=H	0	16.674	6.306306	5	[illegible]
High	P15941-2	' MUC1	Isoform 2 of Mucin-1 OS=Homo sapiens OX=9606 GN=MUC1	0	16.652	3.560127	3	[illegible]
High	Q9BZX2	' UCK2	Uridine-cytidine kinase 2 OS=Homo sapiens OX=9606 GN=UCK2 PE=	0	16.636	20.30651	4	[illegible]
High	O95833	' CLIC3	Chloride intracellular channel protein 3 OS=Homo sapiens OX=9606	0	16.631	36.86441	6	[illegible]
High	Q15418-2	' RPS6KA1	Isoform 2 of Ribosomal protein S6 kinase alpha-1 OS=Homo sapiens	0	16.611	11.82796	7	[illegible]
High	O75351	' VPS4B	Vacuolar protein sorting-associated protein 4B OS=Homo sapiens O	0	16.61	13.06306	6	[illegible]
High	Q01130	' SRSF2	Serine/arginine-rich splicing factor 2 OS=Homo sapiens OX=9606 GN	0	16.545	19.45701	4	[illegible]
High	Q3MHD2-	' LSM12	Isoform 2 of Protein LSM12 homolog OS=Homo sapiens OX=9606 GI	0	16.542	34.34343	5	[illegible]
High	O76031	' CLPX	ATP-dependent Clp protease ATP-binding subunit clpX-like, mitocho	0	16.541	15.79779	7	[illegible]
High	Q9NSE4	' IARS2	Isoleucine--tRNA ligase, mitochondrial OS=Homo sapiens OX=9606 G	0	16.525	7.509881	6	[illegible]
High	Q9BUK6	' MSTO1	Protein misato homolog 1 OS=Homo sapiens OX=9606 GN=MSTO1 P	0	16.484	12.2807	5	[illegible]
High	P55196-1	' AFDN	Isoform 2 of Afadin OS=Homo sapiens OX=9606 GN=AFDN	0	16.481	4.845815	5	[illegible]
High	Q8IY63	' AMOTL1	Angiomotin-like protein 1 OS=Homo sapiens OX=9606 GN=AMOTL1	0	16.46	8.263598	7	[illegible]
High	Q15796	' SMAD2	Mothers against decapentaplegic homolog 2 OS=Homo sapiens OX=	0	16.452	12.20557	4	[illegible]
High	O95433	' AHSA1	Activator of 90 kDa heat shock protein ATPase homolog 1 OS=Homo	0	16.427	18.3432	4	[illegible]
High	Q9H267	' VPS33B	Vacuolar protein sorting-associated protein 33B OS=Homo sapiens O	0	16.389	13.29011	7	[illegible]
High	Q9BZH6	' WDR11	WD repeat-containing protein 11 OS=Homo sapiens OX=9606 GN=W	0	16.375	6.535948	6	[illegible]
High	O95235	' KIF20A	Kinesin-like protein KIF20A OS=Homo sapiens OX=9606 GN=KIF20A	0	16.372	5.617978	4	[illegible]
High	P31948-2	' STIP1	Isoform 2 of Stress-induced-phosphoprotein 1 OS=Homo sapiens OX	0	16.366	9.491525	5	[illegible]
High	P10301	' RRAS	Ras-related protein R-Ras OS=Homo sapiens OX=9606 GN=RRAS PE=	0	16.35	24.77064	4	[illegible]
High	Q9H329-2	' EPB41L4B	Isoform 2 of Band 4.1-like protein 4B OS=Homo sapiens OX=9606 GI	0	16.334	12.16216	6	[illegible]
High	Q9P0L2	' MARK1	Serine/threonine-protein kinase MARK1 OS=Homo sapiens OX=9606	0	16.331	7.54717	5	[illegible]
High	Q12986	' NFX1	Transcriptional repressor NF-X1 OS=Homo sapiens OX=9606 GN=NF	0	16.329	8.571429	6	[illegible]
High	Q9Y3B4	' SF3B6	Splicing factor 3B subunit 6 OS=Homo sapiens OX=9606 GN=SF3B6 P	0	16.328	45.6	5	[illegible]
High	O00635	' TRIM38	E3 ubiquitin-protein ligase TRIM38 OS=Homo sapiens OX=9606 GN=	0	16.319	11.39785	5	[illegible]
High	Q6UXN9	' WDR82	WD repeat-containing protein 82 OS=Homo sapiens OX=9606 GN=W	0	16.317	23.00319	6	[illegible]
High	P12277	' CKB	Creatine kinase B-type OS=Homo sapiens OX=9606 GN=CKB PE=1 SV	0	16.309	11.28609	3	[illegible]
High	O15260	' SURF4	Surfeit locus protein 4 OS=Homo sapiens OX=9606 GN=SURF4 PE=1	0	16.306	14.86989	4	[illegible]
High	P26006-1	' ITGA3	Isoform 2 of Integrin alpha-3 OS=Homo sapiens OX=9606 GN=ITGA3	0	16.273	5.816135	5	[illegible]
High	Q9H269	' VPS16	Vacuolar protein sorting-associated protein 16 homolog OS=Homo s	0	16.271	7.866508	6	[illegible]
High	Q9NXH9	' TRMT1	tRNA (guanine(26)-N(2))-dimethyltransferase OS=Homo sapiens OX	0	16.248	12.89833	6	[illegible]
High	P19388	' POLR2E	DNA-directed RNA polymerases I, II, and III subunit RPABC1 OS=Hon	0	16.242	22.85714	4	[illegible]
High	Q13616	' CUL1	Cullin-1 OS=Homo sapiens OX=9606 GN=CUL1 PE=1 SV=2	0	16.202	8.634021	6	[illegible]
High	Q9UM00	' TMCO1	Calcium load-activated calcium channel OS=Homo sapiens OX=9606	0	16.193	24.46809	4	[illegible]
High	O95292	' VAPB	Vesicle-associated membrane protein-associated protein B/C OS=Ho	0	16.151	15.63786	4	[illegible]
High	P61221	' ABCE1	ATP-binding cassette sub-family E member 1 OS=Homo sapiens OX=	0	16.149	12.52087	6	[illegible]
High	Q658Y4	' FAM91A1	Protein FAM91A1 OS=Homo sapiens OX=9606 GN=FAM91A1 PE=1 S	0	16.141	9.546539	5	[illegible]
High	P56182	' RRP1	Ribosomal RNA processing protein 1 homolog A OS=Homo sapiens O	0	16.133	14.09978	4	[illegible]
High	O60783	' MRPS14	28S ribosomal protein S14, mitochondrial OS=Homo sapiens OX=96	0	16.096	28.90625	3	[illegible]
High	P00568	' AK1	Adenylate kinase isoenzyme 1 OS=Homo sapiens OX=9606 GN=AK1	0	16.06	29.89691	5	[illegible]
High	Q96FZ2	' HMCES	Embryonic stem cell-specific 5-hydroxymethylcytosine-binding prot	0	16.052	21.46893	5	[illegible]
High	O00116	' AGPS	Alkyldihydroxyacetonephosphate synthase, peroxisomal OS=Homo	0	15.989	13.98176	6	[illegible]
High	Q9NWB6	' ARGLU1	Arginine and glutamate-rich protein 1 OS=Homo sapiens OX=9606 G	0	15.968	20.51282	7	[illegible]
High	Q8N4C8	' MINK1	Misshapen-like kinase 1 OS=Homo sapiens OX=9606 GN=MINK1 PE=	0	15.964	6.531532	6	[illegible]
High	Q9NZR1	' TMOD2	Tropomodulin-2 OS=Homo sapiens OX=9606 GN=TMOD2 PE=1 SV=1	0	15.958	19.94302	5	[illegible]
High	P49757	' NUMB	Protein numb homolog OS=Homo sapiens OX=9606 GN=NUMB PE=1	0	15.936	9.21659	5	[illegible]
High	Q8TCT9	' HM13	Minor histocompatibility antigen H13 OS=Homo sapiens OX=9606 G	0	15.912	12.20159	3	[illegible]
High	O14841	' OPLAH	5-oxoprolinase OS=Homo sapiens OX=9606 GN=OPLAH PE=1 SV=3	0	15.888	6.599379	6	[illegible]
High	Q92696	' RABGGTA	Geranylgeranyl transferase type-2 subunit alpha OS=Homo sapiens	0	15.888	10.75838	6	[illegible]
High	Q9BXY0	' MAK16	Protein MAK16 homolog OS=Homo sapiens OX=9606 GN=MAK16 PE	0	15.888	17	4	[illegible]
High	P00403	' MT-CO2	Cytochrome c oxidase subunit 2 OS=Homo sapiens OX=9606 GN=MT	0	15.866	16.29956	3	[illegible]
High	Q96PY5-3	' FMNL2	Isoform 2 of Formin-like protein 2 OS=Homo sapiens OX=9606 GN=F	0	15.857	5.494505	5	[illegible]
High	Q9BQ67	' GRWD1	Glutamate-rich WD repeat-containing protein 1 OS=Homo sapiens O	0	15.83	12.10762	4	[illegible]
High	Q9H5H4	' ZNF768	Zinc finger protein 768 OS=Homo sapiens OX=9606 GN=ZNF768 PE=	0	15.803	11.2963	6	[illegible]
High	P09012	' SNRPA	U1 small nuclear ribonucleoprotein A OS=Homo sapiens OX=9606 G	0	15.792	24.46809	6	[illegible]
High	Q5BKY9	' FAM133B	Protein FAM133B OS=Homo sapiens OX=9606 GN=FAM133B PE=1 S	0	15.748	31.17409	5	[illegible]
High	Q8IY17-4	' PNPLA6	Isoform 4 of Neuropathy target esterase OS=Homo sapiens OX=960	0	15.73	5.527273	4	[illegible]
High	O00629	' KPNA4	Importin subunit alpha-3 OS=Homo sapiens OX=9606 GN=KPNA4 PE	0	15.721	11.32438	4	[illegible]
High	Q96A33	' CCDC47	Coiled-coil domain-containing protein 47 OS=Homo sapiens OX=960	0	15.672	17.18427	5	[illegible]
High	P55263	' ADK	Adenosine kinase OS=Homo sapiens OX=9606 GN=ADK PE=1 SV=2	0	15.663	16.57459	5	[illegible]
High	P55081	' MFAP1	Microfibrillar-associated protein 1 OS=Homo sapiens OX=9606 GN=	0	15.651	15.94533	5	[illegible]
High	P24539	' ATP5F1	ATP synthase F(0) complex subunit B1, mitochondrial OS=Homo sap	0	15.646	22.26563	5	[illegible]
High	Q92843-2	' BCL2L2	Isoform 3 of Bcl-2-like protein 2 OS=Homo sapiens OX=9606 GN=BC	0	15.634	11.71171	5	[illegible]
High	Q7L1Q6-3	' BZW1	Isoform 3 of Basic leucine zipper and W2 domain-containing protein	0	15.612	15.07761	6	[illegible]
High	P63172	' DYNLT1	Dynein light chain Tctex-type 1 OS=Homo sapiens OX=9606 GN=DYN	0	15.602	44.24779	3	[illegible]
High	P35658-5	' NUP214	Isoform 5 of Nuclear pore complex protein Nup214 OS=Homo sapie	0	15.595	4.108935	6	[illegible]
High	Q8TCJ2	' STT3B	Dolichyl-diphosphooligosaccharide--protein glycosyltransferase sub	0	15.577	7.748184	6	[illegible]
High	P13674	' P4HA1	Prolyl 4-hydroxylase subunit alpha-1 OS=Homo sapiens OX=9606 GN	0	15.576	8.052434	4	[illegible]
High	Q9BV20	' MRI1	Methylthioribose-1-phosphate isomerase OS=Homo sapiens OX=96	0	15.576	18.42818	5	[illegible]
High	Q01804	' OTUD4	OTU domain-containing protein 4 OS=Homo sapiens OX=9606 GN=O	0	15.549	6.912029	6	[illegible]
High	O60341-2	' KDM1A	Isoform 2 of Lysine-specific histone demethylase 1A OS=Homo sapie	0	15.548	7.534247	6	[illegible]
High	Q93009	' USP7	Ubiquitin carboxyl-terminal hydrolase 7 OS=Homo sapiens OX=9606	0	15.529	4.627949	5	[illegible]
High	P63151-2	' PPP2R2A	Isoform 2 of Serine/threonine-protein phosphatase 2A 55 kDa regul	0	15.508	10.94092	3	[illegible]
High	Q6R327-3	' RICTOR	Isoform 3 of Rapamycin-insensitive companion of mTOR OS=Homo s	0	15.47	3.464203	4	[illegible]
High	P08758	' ANXA5	Annexin A5 OS=Homo sapiens OX=9606 GN=ANXA5 PE=1 SV=2	0	15.466	15.625	5	[illegible]
High	A8CG34	' POM121C	Nuclear envelope pore membrane protein POM 121C OS=Homo sap	0	15.435	9.194467	7	[illegible]
High	Q53GS9	' USP39	U4/U6.U5 tri-snRNP-associated protein 2 OS=Homo sapiens OX=960	0	15.422	12.38938	6	[illegible]
High	Q8WVX9	' FAR1	Fatty acyl-CoA reductase 1 OS=Homo sapiens OX=9606 GN=FAR1 PE	0	15.398	10.09709	4	[illegible]
High	Q9NX20	' MRPL16	39S ribosomal protein L16, mitochondrial OS=Homo sapiens OX=960	0	15.331	22.31076	5	[illegible]
High	Q96A35	' MRPL24	39S ribosomal protein L24, mitochondrial OS=Homo sapiens OX=960	0	15.325	18.51852	3	[illegible]
High	O75947	' ATP5H	ATP synthase subunit d, mitochondrial OS=Homo sapiens OX=9606 O	0	15.309	40.99379	5	[illegible]
High	Q01650	' SLC7A5	Large neutral amino acids transporter small subunit 1 OS=Homo sap	0	15.301	7.692308	3	[illegible]
High	O75152	' ZC3H11A	Zinc finger CCCH domain-containing protein 11A OS=Homo sapiens	0	15.286	8.765432	4	[illegible]
High	Q2M2I5	' KRT24	Keratin, type I cytoskeletal 24 OS=Homo sapiens OX=9606 GN=KRT2	0	15.273	6.666667	4	[illegible]
High	Q01469	' FABP5	Fatty acid-binding protein 5 OS=Homo sapiens OX=9606 GN=FABP5	0	15.262	47.40741	5	[illegible]
High	Q92979	' EMG1	Ribosomal RNA small subunit methyltransferase NEP1 OS=Homo sa	0	15.258	26.22951	4	[illegible]
High	Q16576-2	' RBBP7	Isoform 2 of Histone-binding protein RBBP7 OS=Homo sapiens OX=9	0	15.237	11.72708	6	[illegible]
High	O00422	' SAP18	Histone deacetylase complex subunit SAP18 OS=Homo sapiens OX=	0	15.221	30.06536	6	[illegible]
High	O43294	' TGFB1I1	Transforming growth factor beta-1-induced transcript 1 protein OS=	0	15.187	9.978308	4	[illegible]
High	Q02750	' MAP2K1	Dual specificity mitogen-activated protein kinase kinase 1 OS=Homo	0	15.132	18.32061	5	[illegible]
High	Q9BQ69	' MACROD1	O-acetyl-ADP-ribose deacetylase MACROD1 OS=Homo sapiens OX=9	0	15.128	14.76923	3	[illegible]
High	Q9UI10-2	' EIF2B4	Isoform 2 of Translation initiation factor eIF-2B subunit delta OS=Ho	0	15.101	15.10129	5	[illegible]
High	Q8IWZ3-6	' ANKHD1	Isoform 6 of Ankyrin repeat and KH domain-containing protein 1 OS	0	15.095	2.560183	5	[illegible]
High	Q9BPW9-	' DHRS9	Isoform 4 of Dehydrogenase/reductase SDR family member 9 OS=Ho	0	15.074	13.72032	4	[illegible]
High	Q9Y333	' LSM2	U6 snRNA-associated Sm-like protein LSm2 OS=Homo sapiens OX=9	0	15.07	48.42105	3	[illegible]
High	Q9H553	' ALG2	Alpha-1,3/1,6-mannosyltransferase ALG2 OS=Homo sapiens OX=960	0	15.032	18.75	6	[illegible]
High	Q9NTJ5	' SACM1L	Phosphatidylinositide phosphatase SAC1 OS=Homo sapiens OX=960	0	15.031	12.26576	6	[illegible]
High	Q9Y5Y2	' NUBP2	Cytosolic Fe-S cluster assembly factor NUBP2 OS=Homo sapiens OX=	0	15.025	22.87823	3	[illegible]
High	Q8N5P1	' ZC3H8	Zinc finger CCCH domain-containing protein 8 OS=Homo sapiens OX	0	15.017	20.9622	4	[illegible]
High	Q9ULA0	' DNPEP	Aspartyl aminopeptidase OS=Homo sapiens OX=9606 GN=DNPEP PE	0	15.017	14.73684	4	[illegible]
High	O95407	' TNFRSF6B	Tumor necrosis factor receptor superfamily member 6B OS=Homo s	0	14.988	23.66667	5	[illegible]
High	P68402	' PAFAH1B2	Platelet-activating factor acetylhydrolase IB subunit beta OS=Homo	0	14.984	19.65066	4	[illegible]
High	O43615	' TIMM44	Mitochondrial import inner membrane translocase subunit TIM44 O	0	14.981	13.71681	5	[illegible]
High	Q9NXG2	' THUMPD1	THUMP domain-containing protein 1 OS=Homo sapiens OX=9606 GI	0	14.966	20.96317	4	[illegible]
High	P35240-2	' NF2	Isoform 2 of Merlin OS=Homo sapiens OX=9606 GN=NF2	0	14.962	8.709677	5	[illegible]

High	Q9H3P7	'ACBD3	Golgi resident protein GCP60 OS=Homo sapiens OX=9606 GN=ACBD	0	14.937	12.87879	5	13	4	528	60.6	5.06		0.492
High	Q96GA3	'LTV1	Protein LTV1 homolog OS=Homo sapiens OX=9606 GN=LTV1 PE=1 S	0	14.926	14.31579	5	8	5	475	54.8	4.91		0.509
High	Q9NP72	'RAB18	Ras-related protein Rab-18 OS=Homo sapiens OX=9606 GN=RAB18	0	14.919	27.18447	5	17	5	206	23	5.24		1.276
High	Q96TA2	'YME1L1	ATP-dependent zinc metalloprotease YME1L1 OS=Homo sapiens OX	0	14.918	10.60802	5	12	5	773	86.4	8.76		0.253
High	P29353-6	'SHC1	Isoform 6 of SHC-transforming protein 1 OS=Homo sapiens OX=960	0	14.879	14.72603	4	20	4	584	62.9	6.44		0.585
High	Q5BKZ1	'ZNF326	DBIRD complex subunit ZNF326 OS=Homo sapiens OX=9606 GN=ZN	0	14.873	12.54296	5	14	5	582	65.6	5.15		0.403
High	Q12834	'CDC20	Cell division cycle protein 20 homolog OS=Homo sapiens OX=9606 G	0	14.868	18.23647	5	10	5	499	54.7	9.23		0.557
High	Q92520	'FAM3C	Protein FAM3C OS=Homo sapiens OX=9606 GN=FAM3C PE=1 SV=1	0	14.861	22.02643	4	5	4	227	24.7	8.29		0.778
High	Q13162	'PRDX4	Peroxiredoxin-4 OS=Homo sapiens OX=9606 GN=PRDX4 PE=1 SV=1	0	14.823	18.45018	5	23	3	271	30.5	6.29		1.154
High	P55010	'EIF5	Eukaryotic translation initiation factor 5 OS=Homo sapiens OX=9606	0	14.822	10.44084	4	11	4	431	49.2	5.58		0.407
High	P55735-3	'SEC13	Isoform 3 of Protein SEC13 homolog OS=Homo sapiens OX=9606 GN	0	14.811	15.48913	4	8	4	368	40.7	5.85		0.848
High	P29034	'S100A2	Protein S100-A2 OS=Homo sapiens OX=9606 GN=S100A2 PE=1 SV=3	0	14.811	27.55102	5	22	5	98	11.1	4.78		14.849
High	Q14790-4	'CASP8	Isoform 4 of Caspase-8 OS=Homo sapiens OX=9606 GN=CASP8	0	14.784	9.879032	5	14	5	496	57.7	5.43		0.487
High	Q15056	'EIF4H	Eukaryotic translation initiation factor 4H OS=Homo sapiens OX=960	0	14.774	20.16129	4	7	4	248	27.4	7.23		0.551
High	P61619	'SEC61A1	Protein transport protein Sec61 subunit alpha isoform 1 OS=Homo s	0	14.629	7.773109	4	21	4	476	52.2	8.06		0.778
High	Q5JPE7	'NOMO2	Nodal modulator 2 OS=Homo sapiens OX=9606 GN=NOMO2 PE=1 S	0	14.612	8.12944	7	11	7	1267	139.4	5.76		0.243
High	Q8TDX7	'NEK7	Serine/threonine-protein kinase Nek7 OS=Homo sapiens OX=9606 G	0	14.611	22.18543	5	11	5	302	34.5	8.25		0.833
High	Q14165	'MLEC	Malectin OS=Homo sapiens OX=9606 GN=MLEC PE=1 SV=1	0	14.593	19.52055	5	12	5	292	32.2	5.41		0.778
High	O15160	'POLR1C	DNA-directed RNA polymerases I and III subunit RPAC1 OS=Homo sa	0	14.592	16.18497	4	6	4	346	39.2	5.5		0.778
High	Q3LXA3	'TKFC	Triokinase/FMN cyclase OS=Homo sapiens OX=9606 GN=TKFC PE=1	0	14.591	10.95652	4	10	4	575	58.9	7.49		0.417
High	O43837	'IDH3B	Isocitrate dehydrogenase [NAD] subunit beta, mitochondrial OS=Ho	0	14.588	12.20779	4	7	4	385	42.2	8.46		0.492
High	Q92997	'DVL3	Segment polarity protein dishevelled homolog DVL-3 OS=Homo sapi	0	14.587	7.122905	4	11	2	716	78	6.65		0.334
High	Q92544	'TM9SF4	Transmembrane 9 superfamily member 4 OS=Homo sapiens OX=96	0	14.536	9.813084	4	5	4	642	74.5	6.54		0.425
High	P39880-3	'CUX1	Isoform 3 of Homeobox protein cut-like 1 OS=Homo sapiens OX=96	0	14.463	5.738786	7	8	7	1516	165.6	5.86		0.214
High	Q6NUM9	'RETSAT	All-trans-retinol 13,14-reductase OS=Homo sapiens OX=9606 GN=R	0	14.461	12.29508	5	5	5	610	66.8	8.28		0.365
High	Q9Y613	'FHOD1	FH1/FH2 domain-containing protein 1 OS=Homo sapiens OX=9606 G	0	14.443	7.044674	5	5	5	1164	126.5	6.39		0.191
High	Q9NWT1	'PAK1IP1	p21-activated protein kinase-interacting protein 1 OS=Homo sapien	0	14.433	10.71429	5	6	5	392	43.9	8.91		0.551
High	P57740	'NUP107	Nuclear pore complex protein Nup107 OS=Homo sapiens OX=9606	0	14.431	8.540541	5	6	5	925	106.3	5.43		0.22
High	O95747	'OXSR1	Serine/threonine-protein kinase OSR1 OS=Homo sapiens OX=9606	0	14.403	9.677419	5	17	4	527	58	6.43		0.487
High	P62861	'FAU	40S ribosomal protein S30 OS=Homo sapiens OX=9606 GN=FAU PE=	0	14.399	33.89831	4	27	4	59	6.6	12.15		45.416
High	Q9HAZ1	'CLK4	Dual specificity protein kinase CLK4 OS=Homo sapiens OX=9606 GN	0	14.387	9.97921	3	8	2	481	57.5	8.68		0.292
High	P51608-2	'MECP2	Isoform B of Methyl-CpG-binding protein 2 OS=Homo sapiens OX=9	0	14.368	18.4739	7	10	7	498	53.3	9.88		0.817
High	O75489	'NDUFS3	NADH dehydrogenase [ubiquinone] iron-sulfur protein 3, mitochond	0	14.332	20.07576	4	10	4	264	30.2	7.5		0.585
High	P17931	'LGALS3	Galectin-3 OS=Homo sapiens OX=9606 GN=LGALS3 PE=1 SV=5	0	14.326	18.8	4	11	4	250	26.1	8.56		1.848
High	Q9BRJ6	'C7orf50	Uncharacterized protein C7orf50 OS=Homo sapiens OX=9606 GN=C	0	14.325	29.38144	4	18	4	194	22.1	9.64		1.848
High	P62857	'RPS28	40S ribosomal protein S28 OS=Homo sapiens OX=9606 GN=RPS28 P	0	14.292	46.37681	3	13	3	69	7.8	10.7		9
High	P61604	'HSPE1	10 kDa heat shock protein, mitochondrial OS=Homo sapiens OX=96	0	14.277	42.15686	4	5	4	102	10.9	8.92		1.512
High	O43747-2	'AP1G1	Isoform 2 of AP-1 complex subunit gamma-1 OS=Homo sapiens OX=	0	14.256	7.151515	5	13	5	825	91.7	6.8		0.292
High	P61970	'NUTF2	Nuclear transport factor 2 OS=Homo sapiens OX=9606 GN=NUTF2 P	0	14.249	51.1811	4	7	4	127	14.5	5.38		3.217
High	O60333-3	'KIF1B	Isoform 3 of Kinesin-like protein KIF1B OS=Homo sapiens OX=9606	0	14.235	5.810928	5	13	2	1153	130.3	8.43		0.166
High	P06730-2	'EIF4E	Isoform 2 of Eukaryotic translation initiation factor 4E OS=Homo sap	0	14.199	20.56452	5	14	5	248	28.8	6.42		0.968
High	O76041-2	'NEBL	Isoform 2 of Nebulette OS=Homo sapiens OX=9606 GN=NEBL	0	14.166	28.51852	5	13	4	270	31.2	8.31		0.778
High	Q93050-3	'ATP6V0A1	Isoform 3 of V-type proton ATPase 116 kDa subunit a isoform 1 OS=	0	14.151	6.324582	4	7	4	838	96.5	6.64		0.245
High	Q9UBX3-2	'SLC25A10	Isoform 2 of Mitochondrial dicarboxylate carrier OS=Homo sapiens	0	14.135	18.24324	4	8	4	296	32.1	9.35		0.624
High	Q9H0D6	'XRN2	5'-3' exoribonuclease 2 OS=Homo sapiens OX=9606 GN=XRN2 PE=1	0	14.134	7.157895	5	17	5	950	108.5	7.47		0.224
High	P49755	'TMED10	Transmembrane emp24 domain-containing protein 10 OS=Homo sa	0	14.113	14.61187	3	19	3	219	25	7.44		0.701
High	Q16762	'TST	Thiosulfate sulfurtransferase OS=Homo sapiens OX=9606 GN=TST P	0	14.112	13.80471	4	11	4	297	33.4	7.25		0.585
High	Q14739	'LBR	Lamin-B receptor OS=Homo sapiens OX=9606 GN=LBR PE=1 SV=2	0	14.093	7.479675	4	9	4	615	70.7	9.36		0.389
High	Q04206	'RELA	Transcription factor p65 OS=Homo sapiens OX=9606 GN=RELA PE=1	0	14.079	9.618875	4	6	4	551	60.2	5.68		0.407
High	Q8WUM0	'NUP133	Nuclear pore complex protein Nup133 OS=Homo sapiens OX=9606	0	14.055	6.487889	6	11	6	1156	128.9	5.1		0.237
High	O00264	'PGRMC1	Membrane-associated progesterone receptor component 1 OS=Hom	0	14.043	30.25641	5	12	4	195	21.7	4.7		1.848
High	Q9NV31	'IMP3	U3 small nucleolar ribonucleoprotein protein IMP3 OS=Homo sapie	0	14.018	23.36957	3	7	3	184	21.8	9.5		0.874
High	Q13671	'RIN1	Ras and Rab interactor 1 OS=Homo sapiens OX=9606 GN=RIN1 PE=1	0	14.017	6.896552	4	6	4	783	84	8.02		0.324
High	Q96G21	'IMP4	U3 small nucleolar ribonucleoprotein protein IMP4 OS=Homo sapie	0	14.015	18.90034	4	7	4	291	33.7	9.47		0.719
High	Q6Y7W6-	'GIGYF2	Isoform 2 of GRB10-interacting GYF protein 2 OS=Homo sapiens OX	0	14.008	4.469697	4	7	4	1320	152.3	5.63		0.16
High	P10606	'COX5B	Cytochrome c oxidase subunit 5B, mitochondrial OS=Homo sapiens	0	13.985	19.37984	4	21	4	129	13.7	8.81		2.162
High	P04183	'TK1	Thymidine kinase, cytosolic OS=Homo sapiens OX=9606 GN=TK1 PE	0	13.981	27.35043	5	8	5	234	25.5	8.51		1.054
High	Q9HCJ6	'VAT1L	Synaptic vesicle membrane protein VAT-1 homolog-like OS=Homo s	0	13.965	11.93317	4	10	4	419	45.9	5.06		0.468
High	Q12769	'NUP160	Nuclear pore complex protein Nup160 OS=Homo sapiens OX=9606	0	13.955	4.038997	5	8	5	1436	162	5.5		0.179
High	P30837	'ALDH1B1	Aldehyde dehydrogenase X, mitochondrial OS=Homo sapiens OX=96	0	13.955	11.21857	5	11	3	517	57.2	6.8		0.487
High	P07199	'CENPB	Major centromere autoantigen B OS=Homo sapiens OX=9606 GN=C	0	13.924	9.51586	4	14	4	599	65.1	4.55		0.425
High	Q93008	'USP9X	Probable ubiquitin carboxyl-terminal hydrolase FAF-X OS=Homo sap	0	13.92	2.29572	5	6	5	2570	292.1	5.8		0.084
High	Q96KR1	'ZFR	Zinc finger RNA-binding protein OS=Homo sapiens OX=9606 GN=ZF	0	13.917	6.610801	6	8	6	1074	116.9	9.04		0.326
High	Q6PD62	'CTR9	RNA polymerase-associated protein CTR9 homolog OS=Homo sapie	0	13.898	4.177323	4	5	4	1173	133.4	6.77		0.143
High	Q969N2	'PIGT	GPI transamidase component PIG-T OS=Homo sapiens OX=9606 GN	0	13.884	11.07266	5	8	5	578	65.7	8.38		0.433
High	Q6SJ93	'FAM111B	Protein FAM111B OS=Homo sapiens OX=9606 GN=FAM111B PE=1 S	0	13.865	6.40327	4	4	4	734	84.6	8.59		0.212
High	P82914	'MRPS15	28S ribosomal protein S15, mitochondrial OS=Homo sapiens OX=96	0	13.84	19.06615	5	9	5	257	29.8	10.48		0.833
High	Q9Y6G9	'DYNC1LI1	Cytoplasmic dynein 1 light intermediate chain 1 OS=Homo sapiens	0	13.829	14.14914	5	6	5	523	56.5	6.42		0.45
High	Q9Y3T9	'NOC2L	Nucleolar complex protein 2 homolog OS=Homo sapiens OX=9606 G	0	13.81	5.607477	4	5	4	749	84.9	5.62		0.292
High	Q6P158	'DHX57	Putative ATP-dependent RNA helicase DHX57 OS=Homo sapiens OX	0	13.786	5.844156	6	9	6	1386	155.5	7.71		0.186
High	P29466	'CASP1	Caspase-1 OS=Homo sapiens OX=9606 GN=CASP1 PE=1 SV=1	0	13.785	14.10891	4	5	4	404	45.1	5.91		0.551
High	Q8IWC1	'MAP7D3	MAP7 domain-containing protein 3 OS=Homo sapiens OX=9606 GN	0	13.751	7.990868	6	9	6	876	98.4	9.32		0.269
High	Q96L92	'SNX27	Sorting nexin-27 OS=Homo sapiens OX=9606 GN=SNX27 PE=1 SV=2	0	13.74	14.2329	6	11	6	541	61.2	6.49		0.562
High	P50502	'ST13	Hsc70-interacting protein OS=Homo sapiens OX=9606 GN=ST13 PE=	0	13.724	13.27913	4	11	4	369	41.3	5.27		0.668
High	Q9NUL7	'DDX28	Probable ATP-dependent RNA helicase DDX28 OS=Homo sapiens OX	0	13.704	15.55556	5	5	5	540	59.5	10.42		0.377
High	Q9NW64	'RBM22	Pre-mRNA-splicing factor RBM22 OS=Homo sapiens OX=9606 GN=R	0	13.688	12.14286	5	14	5	420	46.9	8.54		0.557
High	P06132	'UROD	Uroporphyrinogen decarboxylase OS=Homo sapiens OX=9606 GN=U	0	13.681	15.53134	4	6	4	367	40.8	6.14		0.624
High	Q9H3N1	'TMX1	Thioredoxin-related transmembrane protein 1 OS=Homo sapiens OX	0	13.666	19.28571	5	15	5	280	31.8	4.98		1.61
High	Q15326	'ZMYND11	Zinc finger MYND domain-containing protein 11 OS=Homo sapiens	0	13.659	10.46512	4	11	4	602	70.9	8.53		0.403
High	Q9BRL6	'SRSF8	Serine/arginine-rich splicing factor 8 OS=Homo sapiens OX=9606 GN	0	13.658	16.66667	4	20	2	282	32.3	11.72		0.931
High	Q14241	'ELOA	Elongin-A OS=Homo sapiens OX=9606 GN=ELOA PE=1 SV=2	0	13.654	5.764411	4	8	4	798	89.9	9.57		0.227
High	O15162	'PLSCR1	Phospholipid scramblase 1 OS=Homo sapiens OX=9606 GN=PLSCR1	0	13.651	22.64151	5	9	5	318	35	4.94		1.848
High	Q99985	'SEMA3C	Semaphorin-3C OS=Homo sapiens OX=9606 GN=SEMA3C PE=2 SV=2	0	13.65	9.720373	5	7	5	751	85.2	8.69		0.265
High	Q15773	'MLF2	Myeloid leukemia factor 2 OS=Homo sapiens OX=9606 GN=MLF2 PE	0	13.648	18.54839	3	6	3	248	28.1	6.9		0.778
High	P81605-2	'DCD	Isoform 2 of Dermcidin OS=Homo sapiens OX=9606 GN=DCD	0	13.634	20.66116	3	11	3	121	12.4	7.97		2.162
High	Q03405	'PLAUR	Urokinase plasminogen activator surface receptor OS=Homo sapien	0	13.619	14.32836	3	4	3	335	37	6.65		0.389
High	P60903	'S100A10	Protein S100-A10 OS=Homo sapiens OX=9606 GN=S100A10 PE=1 SV	0	13.602	27.83505	2	23	2	97	11.2	7.37		5.31
High	Q5UIP0	'RIF1	Telomere-associated protein RIF1 OS=Homo sapiens OX=9606 GN=R	0	13.599	2.588997	5	8	5	2472	274.3	5.52		0.084
High	Q15262-4	'PTPRK	Isoform 4 of Receptor-type tyrosine-protein phosphatase kappa OS=	0	13.594	4.309166	4	12	4	1462	164.5	5.83		0.124
High	Q9NRF8	'CTPS2	CTP synthase 2 OS=Homo sapiens OX=9606 GN=CTPS2 PE=1 SV=1	0	13.588	13.31058	5	7	3	586	65.6	6.9		0.389
High	Q15386	'UBE3C	Ubiquitin-protein ligase E3C OS=Homo sapiens OX=9606 GN=UBE3C	0	13.567	6.832872	5	11	5	1083	123.8	6.71		0.201
High	Q9Y4Z0	'LSM4	U6 snRNA-associated Sm-like protein LSm4 OS=Homo sapiens OX=9	0	13.536	20.14388	3	7	3	139	15.3	9.99		0.874
High	Q9ULX6	'AKAP8L	A-kinase anchor protein 8-like OS=Homo sapiens OX=9606 GN=AKA	0	13.529	8.049536	3	8	3	646	71.6	5.07		0.359
High	P09669	'COX6C	Cytochrome c oxidase subunit 6C OS=Homo sapiens OX=9606 GN=C	0	13.525	37.33333	4	17	4	75	8.8	10.39		5.813
High	P05026	'ATP1B1	Sodium/potassium-transporting ATPase subunit beta-1 OS=Homo sa	0	13.524	16.83168	4	9	4	303	35	8.53		0.719
High	Q9UL25	'RAB21	Ras-related protein Rab-21 OS=Homo sapiens OX=9606 GN=RAB21	0	13.445	12	2	12	2	225	24.3	7.94		0.585
High	O14545	'TRAFD1	TRAF-type zinc finger domain-containing protein 1 OS=Homo sapien	0	13.428	7.560137	4	7	4	582	64.8	5.29		0.374
High	P32321-2	'DCTD	Isoform 2 of Deoxycytidylate deaminase OS=Homo sapiens OX=960	0	13.399	29.62963	5	9	5	189	21	7.53		1.61
High	O75390	'CS	Citrate synthase, mitochondrial OS=Homo sapiens OX=9606 GN=CS	0	13.372	11.58798	5	11	5	466	51.7	8.32		0.65
High	O95396	'MOCS3	Adenylyltransferase and sulfurtransferase MOCS3 OS=Homo sapien	0	13.361	11.52174	3	7	3	460	49.6	6.21		0.292
High	Q07866-9	'KLC1	Isoform I of Kinesin light chain 1 OS=Homo sapiens OX=9606 GN=KL	0	13.361	11.26761	5	7	5	639	72.4	7.49		0.324
High	P18859-2	'ATP5J	Isoform 2 of ATP synthase-coupling factor 6, mitochondrial OS=Hom	0	13.358	41.37931	3	9	3	116	13.4	9.07		1.371
High	P78362-2	'SRPK2	Isoform 2 of SRSF protein kinase 2 OS=Homo sapiens OX=9606 GN=	0	13.325	10.58655	6	13	3	699	79	5.22		0.52
High	Q15102	'PAFAH1B3	Platelet-activating factor acetylhydrolase IB subunit gamma OS=Hor	0	13.308	19.04762	5	17	5	231	25.7	6.84		1.054
High	Q96QC0	'PPP1R10	Serine/threonine-protein phosphatase 1 regulatory subunit 10 OS=H	0	13.269	5	4	6	4	940	99	9.17		0.212
High	Q6YN16	'HSDL2	Hydroxysteroid dehydrogenase-like protein 2 OS=Homo sapiens OX	0	13.266	14.35407	4	4	4	418	45.4	7.99		0.389
High	Q9UMY1	'NOL7	Nucleolar protein 7 OS=Homo sapiens OX=9606 GN=NOL7 PE=1 SV=	0	13.245	20.23346	4	7	4	257	29.4	9.67		1.31
High	Q9Y580	'RBM7	RNA-binding protein 7 OS=Homo sapiens OX=9606 GN=RBM7 PE=1	0	13.24	22.93233	4	12	4	266	30.5	9.57		0.968
High	P40121	'CAPG	Macrophage-capping protein OS=Homo sapiens OX=9606 GN=CAPG	0	13.236	13.50575	4	9	4	348	38.5	6.19		0.778
High	Q15434	'RBMS2	RNA-binding motif, single-stranded-interacting protein 2 OS=Homo	0	13.212	15.97052	5	18	3	407	43.9	9.07		0.896
High	P21127	'CDK11B	Cyclin-dependent kinase 11B OS=Homo sapiens OX=9606 GN=CDK1	0	13.151	6.163522	5	7	5	795	92.6	5.54		0.292
High	Q15836	'VAMP3	Vesicle-associated membrane protein 3 OS=Homo sapiens OX=9606	0	13.15	33	2	7	2	100	11.3	8.79		1.512
High	Q9BWJ5	'SF3B5	Splicing factor 3B subunit 5 OS=Homo sapiens OX=9606 GN=SF3B5	0	13.144	55.81395	4	12	4	86	10.1	6.35		3.642
High	Q96AC1-3	'FERMT2	Isoform 3 of Fermitin family homolog 2 OS=Homo sapiens OX=9606	0	13.14	6.695779	3	11	2	687	78.6	6.7		0.239
High	P31944	'CASP14	Caspase-14 OS=Homo sapiens OX=9606 GN=CASP14 PE=1 SV=2	0	13.136	16.52893	3	9	3	242	27.7	5.58		0.468
High	Q8TF05	'PPP4R1	Serine/threonine-protein phosphatase 4 regulatory subunit 1 OS=H	0	13.129	4.842105	5	8	5	950	106.9	4.77		0.278
High	Q9BUQ8	'DDX23	Probable ATP-dependent RNA helicase DDX23 OS=Homo sapiens OX	0	13.127	7.926829	5	9	5	820	95.5	9.55		0.243
High	P14314	'PRKCSH	Glucosidase 2 subunit beta OS=Homo sapiens OX=9606 GN=PRKCSH	0	13.108	8.712121	4	9	4	528	59.4	4.41		0.52
High	O43169	'CYB5B	Cytochrome b5 type B OS=Homo sapiens OX=9606 GN=CYB5B PE=1	0	13.09	31.50685	2	3	2	146	16.3	4.97		1.154
High	P61758	'VBP1	Prefoldin subunit 3 OS=Homo sapiens OX=9606 GN=VBP1 PE=1 SV=	0	13.081	25.38071	4	8	4	197	22.6	7.11		0.848
High	Q8NFH4	'NUP37	Nucleoporin Nup37 OS=Homo sapiens OX=9606 GN=NUP37 PE=1 SV	0	13.077	15.64417	4	7	4	326	36.7	5.92		0.719
High	P05455	'SSB	Lupus La protein OS=Homo sapiens OX=9606 GN=SSB PE=1 SV=2	0	13.052	10.04902	3	4	3	408	46.8	7.12		0.269
High	Q9UBE0	'SAE1	SUMO-activating enzyme subunit 1 OS=Homo sapiens OX=9606 GN	0	13.042	19.9422	4	8	4	346	38.4	5.3		0.65
High	O14640	'DVL1	Segment polarity protein dishevelled homolog DVL-1 OS=Homo sapi	0	13.033	7.769784	4	8	3	695	75.1	7.81		0.301
High	Q9HD20	'ATP13A1	Manganese-transporting ATPase 13A1 OS=Homo sapiens OX=9606	0	13.017	6.146179	5	6	5	1204	132.9	8.13		0.208
High	Q13428-4	'TCOF1	Isoform 4 of Treacle protein OS=Homo sapiens OX=9606 GN=TCOF1	0	13.012	4.330709	5	10	5	1524	155.8	8.95		0.145
High	P11182	'DBT	Lipoamide acyltransferase component of branched-chain alpha-keto	0	12.989	7.261411	3	6	3	482	53.5	8.51		0.269
High	Q86V15	'CASZ1	Zinc finger protein castor homolog 1 OS=Homo sapiens OX=9606 GN	0	12.955	4.206936	4	8	4	1759	189.9	7.03		0.112
High	Q9BVQ7	'SPATA5L1	Spermatogenesis-associated protein 5-like protein 1 OS=Homo sapi	0	12.934	6.507304	4	7	4	753	80.7	8.09		0.233
High	P08574	'CYC1	Cytochrome c1, heme protein, mitochondrial OS=Homo sapiens OX	0	12.907	10.76923	3	8	3	325	35.4	9		0.438
High	Q9BZJ0	'CRNKL1	Crooked neck-like protein 1 OS=Homo sapiens OX=9606 GN=CRNKL	0	12.894	6.721698	5	5	5	848	100.4	8		0.228
High	Q5VWZ2	'LYPLAL1	Lysophospholipase-like protein 1 OS=Homo sapiens OX=9606 GN=L	0	12.88	16.4557	4	11	4	237	26.3	7.84		0.719
High	Q9H000	'MKRN2	Probable E3 ubiquitin-protein ligase makorin-2 OS=Homo sapiens O	0	12.852	11.05769	5	10	5	416	46.9	7.61		0.532
High	Q8IXI1	'RHOT2	Mitochondrial Rho GTPase 2 OS=Homo sapiens OX=9606 GN=RHOT	0	12.833	8.252427	4	16	4	618	68.1	5.86		0.359
High	Q9NWU5	'MRPL22	39S ribosomal protein L22, mitochondrial OS=Homo sapiens OX=96	0	12.787	29.12621	5	11	4	206	23.6	9.94		1.154
High	Q15005	'SPCS2	Signal peptidase complex subunit 2 OS=Homo sapiens OX=9606 GN	0	12.784	19.46903	4	9	4	226	25	8.47		1.031
High	Q05048	'CSTF1	Cleavage stimulation factor subunit 1 OS=Homo sapiens OX=9606 G	0	12.75	10.67285	4	7	4	431	48.3	6.58		0.52
High	A1L0T0	'ILVBL	Acetolactate synthase-like protein OS=Homo sapiens OX=9606 GN=	0	12.724	14.08228	4	8	4	632	67.8	8.15		0.322
High	P43304	'GPD2	Glycerol-3-phosphate dehydrogenase, mitochondrial OS=Homo sap	0	12.693	4.676754	2	5	2	727	80.8	7.69		0.148
High	Q9BVJ7	'DUSP23	Dual specificity protein phosphatase 23 OS=Homo sapiens OX=9606	0	12.679	36	5	8	5	150	16.6	8.21		1.61
High	Q9BT78	'COPS4	COP9 signalosome complex subunit 4 OS=Homo sapiens OX=9606 G	0	12.678	11.57635	4	5	4	406	46.2	5.83		0.389
High	Q9UBQ0-	'VPS29	Isoform 2 of Vacuolar protein sorting-associated protein 29 OS=Hon	0	12.67	23.65591	4	11	4	186	20.9	7.05		1.31
High	P84022	'SMAD3	Mothers against decapentaplegic homolog 3 OS=Homo sapiens OX=	0	12.666	13.88235	4	14	2	425	48.1	7.15		0.551
High	P82930	'MRPS34	28S ribosomal protein S34, mitochondrial OS=Homo sapiens OX=96	0	12.659	25.68807	5	11	5	218	25.6	9.98		1.154
High	P82673	'MRPS35	28S ribosomal protein S35, mitochondrial OS=Homo sapiens OX=96	0	12.644	21.36223	4	10	4	323	36.8	8.24		0.551
High	Q96BJ8-3	'ELMO3	Isoform 3 of Engulfment and cell motility protein 3 OS=Homo sapier	0	12.639	6.468305	4	7	4	773	87	7.68		0.227
High	Q9HBL7	'PLGRKT	Plasminogen receptor (KT) OS=Homo sapiens OX=9606 GN=PLGRKT	0	12.62	27.21088	4	13	4	147	17.2	9.58		2.594
High	Q15645	'TRIP13	Pachytene checkpoint protein 2 homolog OS=Homo sapiens OX=96	0	12.567	10.41667	4	11	4	432	48.5	6.09		0.445
High	Q13488	'TCIRG1	V-type proton ATPase 116 kDa subunit a isoform 3 OS=Homo sapier	0	12.562	5.783133	4	9	4	830	92.9	7.12		0.233
High	P36969	'GPX4	Phospholipid hydroperoxide glutathione peroxidase OS=Homo sapie	0	12.506	22.33503	3	7	3	197	22.2	8.37		0.585
High	P53350	'PLK1	Serine/threonine-protein kinase PLK1 OS=Homo sapiens OX=9606 G	0	12.491	10.11609	4	10	4	603	68.2	8.91		0.283
High	Q9Y696	'CLIC4	Chloride intracellular channel protein 4 OS=Homo sapiens OX=9606	0	12.473	26.48221	4	10	4	253	28.8	5.59		0.719
High	P07602-3	'PSAP	Isoform Sap-mu-9 of Prosaposin OS=Homo sapiens OX=9606 GN=PS	0	12.472	8.159393	4	10	4	527	58.4	5.14		0.311
High	Q8TB61	'SLC35B2	Adenosine 3'-phospho 5'-phosphosulfate transporter 1 OS=Homo sa	0	12.435	10.18519	5	11	5	432	47.5	9.16		0.73
High	O75151	'PHF2	Lysine-specific demethylase PHF2 OS=Homo sapiens OX=9606 GN=P	0	12.4	6.113139	5	10	5	1096	120.7	9.17		0.228
High	Q9NP77	'SSU72	RNA polymerase II subunit A C-terminal domain phosphatase SSU72	0	12.394	20.10309	3	7	3	194	22.6	5.33		0.874
High	O76003	'GLRX3	Glutaredoxin-3 OS=Homo sapiens OX=9606 GN=GLRX3 PE=1 SV=2	0	12.394	18.20896	5	11	5	335	37.4	5.39		0.833
High	P82921	'MRPS21	28S ribosomal protein S21, mitochondrial OS=Homo sapiens OX=96	0	12.389	39.08046	3	12	3	87	10.7	9.92		2.162
High	Q8IWT6	'LRRC8A	Volume-regulated anion channel subunit LRRC8A OS=Homo sapiens	0	12.385	6.790123	4	6	4	810	94.1	7.94		0.212
High	P50281	'MMP14	Matrix metalloproteinase-14 OS=Homo sapiens OX=9606 GN=MMP	0	12.37	8.934708	4	10	4	582	65.9	7.77		0.389
High	Q14677-3	'CLINT1	Isoform 3 of Clathrin interactor 1 OS=Homo sapiens OX=9606 GN=C	0	12.361	7.931571	4	6	4	643	70.3	6.58		0.425
High	Q8N9B5	'JMY	Junction-mediating and -regulatory protein OS=Homo sapiens OX=9	0	12.331	4.757085	4	8	4	988	111.4	6.18		0.145
High	Q14C86-6	'GAPVD1	Isoform 6 of GTPase-activating protein and VPS9 domain-containing	0	12.322	5.110962	5	10	5	1487	166.1	5.21		0.153
High	Q96I25	'RBM17	Splicing factor 45 OS=Homo sapiens OX=9606 GN=RBM17 PE=1 SV=1	0	12.307	13.21696	5	10	5	401	44.9	5.97		0.585
High	P80748	'IGLV3-21	Immunoglobulin lambda variable 3-21 OS=Homo sapiens OX=9606	0	12.295	23.07692	2	10	2	117	12.4	5.29		2.981
High	Q5XKP0	'MIC13	MICOS complex subunit MIC13 OS=Homo sapiens OX=9606 GN=MIC	0	12.263	49.15254	3	18	3	118	13.1	9.42		2.728
High	Q6P5R6	'RPL22L1	60S ribosomal protein L22-like 1 OS=Homo sapiens OX=9606 GN=RP	0	12.255	39.34426	3	15	3	122	14.6	9.38		2.162
High	O43660	'PLRG1	Pleiotropic regulator 1 OS=Homo sapiens OX=9606 GN=PLRG1 PE=1	0	12.235	6.614786	2	4	2	514	57.2	9.17		0.259
High	Q92878-2	'RAD50	Isoform 2 of DNA repair protein RAD50 OS=Homo sapiens OX=9606	0	12.222	4.400607	6	11	6	1318	154.5	6.89		0.151
High	Q86Y79	'PTRH1	Probable peptidyl-tRNA hydrolase OS=Homo sapiens OX=9606 GN=I	0	12.208	23.36449	4	6	4	214	22.9	10.56		0.931
High	Q96PZ2	'FAM111A	Protein FAM111A OS=Homo sapiens OX=9606 GN=FAM111A PE=1 S	0	12.206	9.328969	3	5	3	611	70.2	8.53		0.292
High	P43307	'SSR1	Translocon-associated protein subunit alpha OS=Homo sapiens OX=	0	12.185	11.88811	3	15	3	286	32.2	4.49		0.778
High	O75306	'NDUFS2	NADH dehydrogenase [ubiquinone] iron-sulfur protein 2, mitochond	0	12.182	7.12743	3	10	3	463	52.5	7.55		0.407
High	P61923-4	'COPZ1	Isoform 4 of Coatomer subunit zeta-1 OS=Homo sapiens OX=9606 G	0	12.17	18.37838	2	8	2	185	21.1	4.88		1.683
High	Q99439	'CNN2	Calponin-2 OS=Homo sapiens OX=9606 GN=CNN2 PE=1 SV=4	0	12.151	15.85761	4	10	3	309	33.7	7.33		0.668
High	P63220	'RPS21	40S ribosomal protein S21 OS=Homo sapiens OX=9606 GN=RPS21 P	0	12.147	44.57831	3	11	3	83	9.1	8.5		1.683
High	P62380	'TBPL1	TATA box-binding protein-like protein 1 OS=Homo sapiens OX=9606	0	12.144	24.19355	3	3	3	186	20.9	9.54		0.874
High	Q13206	'DDX10	Probable ATP-dependent RNA helicase DDX10 OS=Homo sapiens OX	0	12.125	5.714286	4	4	4	875	100.8	8.63		0.202
High	Q13835	'PKP1	Plakophilin-1 OS=Homo sapiens OX=9606 GN=PKP1 PE=1 SV=2	0	12.125	9.236948	5	12	5	747	82.8	9.13		0.233
High	Q8TAD8	'SNIP1	Smad nuclear-interacting protein 1 OS=Homo sapiens OX=9606 GN=	0	12.124	14.64646	4	7	4	396	45.8	9.99	Phospho (	0.52
High	P35610	'SOAT1	Sterol O-acyltransferase 1 OS=Homo sapiens OX=9606 GN=SOAT1 P	0	12.099	7.454545	3	12	3	550	64.7	8.94		0.28
High	O60568	'PLOD3	Procollagen-lysine,2-oxoglutarate 5-dioxygenase 3 OS=Homo sapier	0	12.09	6.097561	3	6	3	738	84.7	6.05		0.158
High	Q9H4M7	'PLEKHA4	Pleckstrin homology domain-containing family A member 4 OS=Hon	0	12.063	8.985879	5	6	5	779	85.3	10.56		0.265
High	Q15008-4	'PSMD6	Isoform 4 of 26S proteasome non-ATPase regulatory subunit 6 OS=H	0	12.05	10.40724	4	4	4	442	51.9	6.74		0.311
High	P52306-5	'RAP1GDS1	Isoform 5 of Rap1 GTPase-GDP dissociation stimulator 1 OS=Homo s	0	12.002	6.085526	3	6	3	608	66.3	5.31		0.184
High	Q9Y4W2	'LAS1L	Ribosomal biogenesis protein LAS1L OS=Homo sapiens OX=9606 GN	0	11.981	6.53951	4	8	4	734	83	4.73		0.239
High	Q70UQ0-	'IKBIP	Isoform 4 of Inhibitor of nuclear factor kappa-B kinase-interacting p	0	11.98	11.93634	4	12	3	377	43.1	4.92		0.445
High	P55809	'OXCT1	Succinyl-CoA:3-ketoacid coenzyme A transferase 1, mitochondrial O	0	11.971	6.730769	2	6	2	520	56.1	7.46		0.172
High	Q9H8Y5	'ANKZF1	Ankyrin repeat and zinc finger domain-containing protein 1 OS=Hon	0	11.944	9.22865	5	10	5	726	80.9	8.41		0.239
High	P49458	'SRP9	Signal recognition particle 9 kDa protein OS=Homo sapiens OX=960	0	11.94	39.53488	4	13	4	86	10.1	7.97		2.728
High	O75038	'PLCH2	1-phosphatidylinositol 4,5-bisphosphate phosphodiesterase eta-2 O	0	11.933	4.096045	4	5	4	1416	154.6	7.84		0.131
High	P22223	'CDH3	Cadherin-3 OS=Homo sapiens OX=9606 GN=CDH3 PE=1 SV=2	0	11.922	6.996381	4	6	4	829	91.4	4.75		0.292
High	P30626	'SRI	Sorcin OS=Homo sapiens OX=9606 GN=SRI PE=1 SV=1	0	11.871	22.72727	5	12	5	198	21.7	5.59		1.61
High	Q29RF7	'PDS5A	Sister chromatid cohesion protein PDS5 homolog A OS=Homo sapie	0	11.868	4.263276	4	8	3	1337	150.7	7.91		0.109
High	Q9NTI5	'PDS5B	Sister chromatid cohesion protein PDS5 homolog B OS=Homo sapie	0	11.858	4.14651	4	7	3	1447	164.6	8.47		0.105
High	P49748-3	'ACADVL	Isoform 3 of Very long-chain specific acyl-CoA dehydrogenase, mito	0	11.847	6.637168	4	9	4	678	72.9	8.75		0.227
High	P09497-2	'CLTB	Isoform Non-brain of Clathrin light chain B OS=Homo sapiens OX=96	0	11.831	16.58768	4	9	4	211	23.2	4.69		1.512
High	P09661	'SNRPA1	U2 small nuclear ribonucleoprotein A' OS=Homo sapiens OX=9606 G	0	11.808	18.03922	4	13	4	255	28.4	8.62		0.848
High	Q9BTT0	'ANP32E	Acidic leucine-rich nuclear phosphoprotein 32 family member E OS=	0	11.807	12.31343	3	10	3	268	30.7	3.85		0.995
High	Q12802-2	'AKAP13	Isoform 2 of A-kinase anchor protein 13 OS=Homo sapiens OX=9606	0	11.803	2.662407	5	9	5	2817	307.7	5.2		0.083
High	O60784-2	'TOM1	Isoform 2 of Target of Myb protein 1 OS=Homo sapiens OX=9606 GN	0	11.796	13.18458	4	4	4	493	53.8	4.74		0.551
High	P27144	'AK4	Adenylate kinase 4, mitochondrial OS=Homo sapiens OX=9606 GN=	0	11.788	17.48879	3	12	3	223	25.3	8.4		0.585
High	Q16831	'UPP1	Uridine phosphorylase 1 OS=Homo sapiens OX=9606 GN=UPP1 PE=	0	11.78	9.677419	3	4	3	310	33.9	7.88		0.413
High	P21266	'GSTM3	Glutathione S-transferase Mu 3 OS=Homo sapiens OX=9606 GN=GS	0	11.766	19.55556	4	14	3	225	26.5	5.54		0.624
High	O75822	'EIF3J	Eukaryotic translation initiation factor 3 subunit J OS=Homo sapiens	0	11.756	16.27907	3	11	3	258	29	4.83		0.54
High	Q15629	'TRAM1	Translocating chain-associated membrane protein 1 OS=Homo sapie	0	11.748	11.49733	3	9	3	374	43	9.63		0.778
High	Q96G03	'PGM2	Phosphoglucomutase-2 OS=Homo sapiens OX=9606 GN=PGM2 PE=	0	11.709	9.313725	5	11	5	612	68.2	6.73		0.324
High	Q96A65	'EXOC4	Exocyst complex component 4 OS=Homo sapiens OX=9606 GN=EXO	0	11.697	6.262834	4	7	4	974	110.4	6.49		0.15
High	Q9UIF9	'BAZ2A	Bromodomain adjacent to zinc finger domain protein 2A OS=Homo	0	11.691	3.989501	4	4	4	1905	211.1	6.64		0.131
High	P07741	'APRT	Adenine phosphoribosyltransferase OS=Homo sapiens OX=9606 GN	0	11.691	31.11111	4	11	4	180	19.6	6.02		0.931
High	Q8N9N2-	'ASCC1	Isoform 2 of Activating signal cointegrator 1 complex subunit 1 OS=	0	11.687	16.52661	5	10	5	357	41.2	5.53		0.688
High	P60983	'GMFB	Glia maturation factor beta OS=Homo sapiens OX=9606 GN=GMFB	0	11.678	26.05634	4	9	4	142	16.7	5.29		1.783
High	P40925-3	'MDH1	Isoform 3 of Malate dehydrogenase, cytoplasmic OS=Homo sapiens	0	11.667	12.78409	3	6	3	352	38.6	7.71		0.318
High	Q96JH7	'VCPIP1	Deubiquitinating protein VCIP135 OS=Homo sapiens OX=9606 GN=V	0	11.644	5.973813	5	6	5	1222	134.2	7.2		0.155
High	P98194-7	'ATP2C1	Isoform 7 of Calcium-transporting ATPase type 2C member 1 OS=Ho	0	11.64	4.008222	3	6	3	973	107.1	6.86		0.134
High	Q8IWR0	'ZC3H7A	Zinc finger CCCH domain-containing protein 7A OS=Homo sapiens O	0	11.622	5.046344	5	10	5	971	110.5	7.3		0.224
High	Q86WR7	'PROSER2	Proline and serine-rich protein 2 OS=Homo sapiens OX=9606 GN=PF	0	11.619	13.56322	5	9	5	435	45.8	7.2		0.65
High	O95478	'NSA2	Ribosome biogenesis protein NSA2 homolog OS=Homo sapiens OX=	0	11.619	18.46154	4	8	4	260	30	10.27		1.031
High	P10398	'ARAF	Serine/threonine-protein kinase A-Raf OS=Homo sapiens OX=9606	0	11.613	7.590759	4	9	2	606	67.5	9.01		0.266
High	Q86W42	'THOC6	THO complex subunit 6 homolog OS=Homo sapiens OX=9606 GN=T	0	11.61	20.2346	5	6	5	341	37.5	7.43		0.778
High	Q9P2R3-4	'ANKFY1	Isoform 4 of Rabankyrin-5 OS=Homo sapiens OX=9606 GN=ANKFY1	0	11.597	4.541701	4	12	4	1211	132.7	6.49		0.147
High	Q9NY12	'GAR1	H/ACA ribonucleoprotein complex subunit 1 OS=Homo sapiens OX=	0	11.588	19.81567	4	10	4	217	22.3	10.92		0.931
High	P00491	'PNP	Purine nucleoside phosphorylase OS=Homo sapiens OX=9606 GN=P	0	11.582	12.11073	3	20	3	289	32.1	6.95		0.52
High	Q00535	'CDK5	Cyclin-dependent-like kinase 5 OS=Homo sapiens OX=9606 GN=CDK	0	11.56	15.75342	4	9	3	292	33.3	7.66		0.585
High	P23458	'JAK1	Tyrosine-protein kinase JAK1 OS=Homo sapiens OX=9606 GN=JAK1	0	11.559	5.372617	5	6	5	1154	133.2	7.55		0.168
High	P15121	'AKR1B1	Aldose reductase OS=Homo sapiens OX=9606 GN=AKR1B1 PE=1 SV=	0	11.554	20.88608	4	16	2	316	35.8	6.98		0.551
High	O95251	'KAT7	Histone acetyltransferase KAT7 OS=Homo sapiens OX=9606 GN=KA	0	11.549	7.692308	3	7	3	611	70.6	8.85		0.225
High	Q8N5A5	'ZGPAT	Zinc finger CCCH-type with G patch domain-containing protein OS=H	0	11.547	8.097928	3	5	3	531	57.3	5.43		0.233
High	P49005	'POLD2	DNA polymerase delta subunit 2 OS=Homo sapiens OX=9606 GN=PO	0	11.508	9.168443	3	10	3	469	51.3	5.58		0.35
High	P35611-3	'ADD1	Isoform 3 of Alpha-adducin OS=Homo sapiens OX=9606 GN=ADD1	0	11.498	7.8125	4	7	4	768	84.3	5.96		0.301
High	Q14624	'ITIH4	Inter-alpha-trypsin inhibitor heavy chain H4 OS=Homo sapiens OX=9	0	11.483	3.225806	3	8	3	930	103.3	6.98		0.148
High	Q7Z2T5	'TRMT1L	TRMT1-like protein OS=Homo sapiens OX=9606 GN=TRMT1L PE=1 S	0	11.467	10.50477	4	8	4	733	81.7	7.88		0.216
High	Q7RTP6	'MICAL3	[F-actin]-monooxygenase MICAL3 OS=Homo sapiens OX=9606 GN=M	0	11.462	2.947053	5	6	3	2002	224.2	5.55		0.096
High	O95816	'BAG2	BAG family molecular chaperone regulator 2 OS=Homo sapiens OX=	0	11.46	20.37915	4	7	4	211	23.8	6.7		0.778
High	Q6P1L8	'MRPL14	39S ribosomal protein L14, mitochondrial OS=Homo sapiens OX=96	0	11.455	29.65517	3	8	3	145	15.9	10.24		0.995
High	P49773	'HINT1	Histidine triad nucleotide-binding protein 1 OS=Homo sapiens OX=9	0	11.437	51.5873	4	6	4	126	13.8	6.95		3.217
High	Q3KQV9	'UAP1L1	UDP-N-acetylhexosamine pyrophosphorylase-like protein 1 OS=Hon	0	11.437	7.100592	3	4	3	507	57	6.32		0.233
High	P10599	'TXN	Thioredoxin OS=Homo sapiens OX=9606 GN=TXN PE=1 SV=3	0	11.434	29.52381	3	14	3	105	11.7	4.92		1.683
High	O43252	'PAPSS1	Bifunctional 3'-phosphoadenosine 5'-phosphosulfate synthase 1 OS	0	11.416	8.173077	3	5	3	624	70.8	6.86		0.205
High	O95394-4	'PGM3	Isoform 3 of Phosphoacetylglucosamine mutase OS=Homo sapiens	0	11.399	7.54386	5	8	5	570	62.9	6		0.433
High	Q9BVS4	'RIOK2	Serine/threonine-protein kinase RIO2 OS=Homo sapiens OX=9606 G	0	11.394	11.77536	4	8	4	552	63.2	5.94		0.359
High	Q71SY5-3	'MED25	Isoform 3 of Mediator of RNA polymerase II transcription subunit 25	0	11.39	8.040201	4	9	4	796	82.9	8.84		0.407
High	Q6P996	'PDXDC1	Pyridoxal-dependent decarboxylase domain-containing protein 1 OS	0	11.389	7.233503	4	10	4	788	86.7	5.38		0.227
High	Q9BW27	'NUP85	Nuclear pore complex protein Nup85 OS=Homo sapiens OX=9606 G	0	11.382	5.030488	2	7	2	656	75	5.55		0.225
High	P52434	'POLR2H	DNA-directed RNA polymerases I, II, and III subunit RPABC3 OS=Hon	0	11.381	26.66667	3	7	3	150	17.1	4.68		0.995
High	Q9ULC5-3	'ACSL5	Isoform 2 of Long-chain-fatty-acid--CoA ligase 5 OS=Homo sapiens C	0	11.381	5.548038	4	9	2	739	82.2	7.39		0.266
High	Q9NXW2-	'DNAJB12	Isoform 2 of DnaJ homolog subfamily B member 12 OS=Homo sapie	0	11.35	9.549072	3	9	3	377	42.2	8.53		0.389
High	P21912	'SDHB	Succinate dehydrogenase [ubiquinone] iron-sulfur subunit, mitocho	0	11.303	11.78571	3	7	3	280	31.6	8.76		0.369
High	Q6IAN0	'DHRS7B	Dehydrogenase/reductase SDR family member 7B OS=Homo sapien	0	11.302	14.46154	4	6	4	325	35.1	9.55		0.551
High	Q13177	'PAK2	Serine/threonine-protein kinase PAK 2 OS=Homo sapiens OX=9606	0	11.268	13.9313	5	8	5	524	58	5.96		0.532
High	Q14684	'RRP1B	Ribosomal RNA processing protein 1 homolog B OS=Homo sapiens C	0	11.261	7.519789	4	6	4	758	84.4	9.76		0.266
High	Q9BTT6	'LRRC1	Leucine-rich repeat-containing protein 1 OS=Homo sapiens OX=960	0	11.253	9.732824	3	8	2	524	59.2	5.02		0.25
High	O95202	'LETM1	Mitochondrial proton/calcium exchanger protein OS=Homo sapiens	0	11.197	7.44249	4	6	4	739	83.3	6.7		0.222
High	Q16850	'CYP51A1	Lanosterol 14-alpha demethylase OS=Homo sapiens OX=9606 GN=C	0	11.195	8.548708	4	7	4	503	56.8	8.53		0.359
High	P38432	'COIL	Coilin OS=Homo sapiens OX=9606 GN=COIL PE=1 SV=1	0	11.194	7.291667	4	9	4	576	62.6	9.07		0.301
High	Q92599	'SEPT8	Septin-8 OS=Homo sapiens OX=9606 GN=SEPT8 PE=1 SV=4	0	11.146	8.074534	5	23	1	483	55.7	6.28		0.532
High	Q96DH6	'MSI2	RNA-binding protein Musashi homolog 2 OS=Homo sapiens OX=960	0	11.121	12.5	3	8	3	328	35.2	8.48		0.701
High	Q13951-2	'CBFB	Isoform 2 of Core-binding factor subunit beta OS=Homo sapiens OX	0	11.115	17.64706	3	11	3	187	22	5.58		0.778
High	Q9NRX1	'PNO1	RNA-binding protein PNO1 OS=Homo sapiens OX=9606 GN=PNO1 P	0	11.104	19.04762	3	5	3	252	27.9	9.73		0.501
High	P63208	'SKP1	S-phase kinase-associated protein 1 OS=Homo sapiens OX=9606 GN	0	11.101	30.67485	3	5	3	163	18.6	4.54		0.778
High	Q96LW7	'CARD19	Caspase recruitment domain-containing protein 19 OS=Homo sapie	0	11.096	13.59649	3	7	3	228	25.6	9.2		0.874
High	Q8WVV4-	'POF1B	Isoform 1 of Protein POF1B OS=Homo sapiens OX=9606 GN=POF1B	0	11.076	8.907563	5	5	5	595	68.7	6.35		0.433
High	Q96B97	'SH3KBP1	SH3 domain-containing kinase-binding protein 1 OS=Homo sapiens	0	11.062	8.270677	4	6	4	665	73.1	6.62		0.252
High	Q14142	'TRIM14	Tripartite motif-containing protein 14 OS=Homo sapiens OX=9606 G	0	11.055	10.181	3	3	3	442	49.7	7.87		0.269
High	Q9NQG5	'RPRD1B	Regulation of nuclear pre-mRNA domain-containing protein 1B OS=	0	11.049	13.19018	3	5	3	326	36.9	5.97		0.468
High	Q8NF37	'LPCAT1	Lysophosphatidylcholine acyltransferase 1 OS=Homo sapiens OX=96	0	11.043	6.554307	3	3	3	534	59.1	6.02		0.292
High	P01619	'IGKV3-20	Immunoglobulin kappa variable 3-20 OS=Homo sapiens OX=9606 GN	0	11.038	21.55172	2	11	1	116	12.5	4.96		0.931
High	O15372	'EIF3H	Eukaryotic translation initiation factor 3 subunit H OS=Homo sapien	0	11.036	15.90909	4	7	4	352	39.9	6.54		0.468
High	Q9BY42	'RTFDC1	Protein RTF2 homolog OS=Homo sapiens OX=9606 GN=RTFDC1 PE=	0	11.025	14.05229	3	5	3	306	33.9	8.59		0.413
High	Q9NQL2	'RRAGD	Ras-related GTP-binding protein D OS=Homo sapiens OX=9606 GN=	0	10.988	7	2	4	2	400	45.6	4.92		0.259
High	Q9UBI6	'GNG12	Guanine nucleotide-binding protein G(I)/G(S)/G(O) subunit gamma-	0	10.966	44.44444	3	6	3	72	8	8.97		2.981
High	P52948	'NUP98	Nuclear pore complex protein Nup98-Nup96 OS=Homo sapiens OX=	0	10.959	3.357182	5	7	5	1817	197.5	6.4		0.153
High	Q9BZF1	'OSBPL8	Oxysterol-binding protein-related protein 8 OS=Homo sapiens OX=9	0	10.957	6.63667	4	5	4	889	101.1	6.96		0.179
High	Q9NUL3	'STAU2	Double-stranded RNA-binding protein Staufen homolog 2 OS=Homo	0	10.952	6.842105	4	12	2	570	62.6	9.61		0.266
High	P78524	'ST5	Suppression of tumorigenicity 5 protein OS=Homo sapiens OX=9606	0	10.937	6.508355	5	8	5	1137	126.4	9.25		0.204
High	Q9Y2R5	'MRPS17	28S ribosomal protein S17, mitochondrial OS=Homo sapiens OX=96	0	10.933	36.15385	3	9	3	130	14.5	9.85		1.683
High	Q9Y450	'HBS1L	HBS1-like protein OS=Homo sapiens OX=9606 GN=HBS1L PE=1 SV=1	0	10.932	5.701754	3	9	3	684	75.4	6.61		0.17
High	Q14145	'KEAP1	Kelch-like ECH-associated protein 1 OS=Homo sapiens OX=9606 GN	0	10.921	6.410256	4	10	4	624	69.6	6.44		0.266
High	P56134	'ATP5J2	ATP synthase subunit f, mitochondrial OS=Homo sapiens OX=9606 G	0	10.897	25.53191	2	10	2	94	10.9	9.67		2.162
High	P13928	'ANXA8	Annexin A8 OS=Homo sapiens OX=9606 GN=ANXA8 PE=1 SV=3	0	10.875	14.06728	4	6	4	327	36.9	5.78		0.492
High	Q12979	'ABR	Active breakpoint cluster region-related protein OS=Homo sapiens (	0	10.874	5.704307	4	7	3	859	97.5	6.55		0.198
High	Q9UBT2	'UBA2	SUMO-activating enzyme subunit 2 OS=Homo sapiens OX=9606 GN	0	10.87	5.9375	4	8	4	640	71.2	5.29		0.292
High	P15374	'UCHL3	Ubiquitin carboxyl-terminal hydrolase isozyme L3 OS=Homo sapiens	0	10.868	20	4	5	4	230	26.2	4.92		1.031
High	Q96F86	'EDC3	Enhancer of mRNA-decapping protein 3 OS=Homo sapiens OX=9606	0	10.855	10.62992	4	9	4	508	56	7.11		0.346
High	Q9NRG9	'AAAS	Aladin OS=Homo sapiens OX=9606 GN=AAAS PE=1 SV=1	0	10.852	6.776557	3	7	3	546	59.5	7.5		0.25
High	Q92643	'PIGK	GPI-anchor transamidase OS=Homo sapiens OX=9606 GN=PIGK PE=	0	10.848	10.63291	3	9	3	395	45.2	6.16		0.445
High	Q9NX24	'NHP2	H/ACA ribonucleoprotein complex subunit 2 OS=Homo sapiens OX=	0	10.807	35.94771	4	6	4	153	17.2	8.22		3.642
High	P07919	'UQCRH	Cytochrome b-c1 complex subunit 6, mitochondrial OS=Homo sapie	0	10.798	28.57143	2	11	2	91	10.7	4.44		0.931
High	Q2TB90	'HKDC1	Putative hexokinase HKDC1 OS=Homo sapiens OX=9606 GN=HKDC1	0	10.764	3.598691	4	19	1	917	102.5	7.12		0.175
High	Q9Y217	'MTMR6	Myotubularin-related protein 6 OS=Homo sapiens OX=9606 GN=MT	0	10.749	7.246377	4	5	4	621	71.9	7.66		0.245
High	Q8WVM8	'SCFD1	Sec1 family domain-containing protein 1 OS=Homo sapiens OX=960	0	10.727	7.320872	3	5	3	642	72.3	6.27		0.205
High	P47914	'RPL29	60S ribosomal protein L29 OS=Homo sapiens OX=9606 GN=RPL29 P	0	10.718	25.15723	4	24	4	159	17.7	11.66		3.642
High	P29966	'MARCKS	Myristoylated alanine-rich C-kinase substrate OS=Homo sapiens OX	0	10.695	15.36145	2	8	2	332	31.5	4.45		0.468
High	Q96GD4-5	'AURKB	Isoform 5 of Aurora kinase B OS=Homo sapiens OX=9606 GN=AURK	0	10.664	14.2029	4	9	4	345	39.4	9.36		0.52
High	P69891	'HBG1	Hemoglobin subunit gamma-1 OS=Homo sapiens OX=9606 GN=HBG	0	10.648	21.76871	3	27	3	147	16.1	7.2		0.778
High	Q92665	'MRPS31	28S ribosomal protein S31, mitochondrial OS=Homo sapiens OX=96	0	10.647	13.41772	3	4	3	395	45.3	9.29		0.292
High	Q9NQC3	'RTN4	Reticulon-4 OS=Homo sapiens OX=9606 GN=RTN4 PE=1 SV=2	0	10.643	5.285235	4	9	4	1192	129.9	4.5		0.179
High	Q13443	'ADAM9	Disintegrin and metalloproteinase domain-containing protein 9 OS=	0	10.626	4.273504	3	9	3	819	90.5	7.52		0.148
High	O14880	'MGST3	Microsomal glutathione S-transferase 3 OS=Homo sapiens OX=9606	0	10.593	18.42105	2	7	2	152	16.5	9.38		0.778

High	Q8IZQ5	'SELENOH	Selenoprotein H OS=Homo sapiens OX=9606 GN=SELENOH PE=1 SV	0	10.591	18.03279
High	Q5VT25-6	'CDC42BPA	Isoform 6 of Serine/threonine-protein kinase MRCK alpha OS=Homo	0	10.581	2.975856
High	Q8N3F8	'MICALL1	MICAL-like protein 1 OS=Homo sapiens OX=9606 GN=MICALL1 PE=1	0	10.573	6.257242
High	Q9H4G0	'EPB41L1	Band 4.1-like protein 1 OS=Homo sapiens OX=9606 GN=EPB41L1 PE	0	10.541	7.037457
High	Q96NE9	'FRMD6	FERM domain-containing protein 6 OS=Homo sapiens OX=9606 GN=	0	10.527	5.144695
High	Q5HYI8	'RABL3	Rab-like protein 3 OS=Homo sapiens OX=9606 GN=RABL3 PE=1 SV=1	0	10.526	26.27119
High	Q7Z4W1	'DCXR	L-xylulose reductase OS=Homo sapiens OX=9606 GN=DCXR PE=1 SV	0	10.524	13.52459
High	Q6P1X6	'C8orf82	UPF0598 protein C8orf82 OS=Homo sapiens OX=9606 GN=C8orf82 F	0	10.505	20.37037
High	Q9Y570-4	'PPME1	Isoform 4 of Protein phosphatase methylesterase 1 OS=Homo sapie	0	10.504	12.75
High	O94855-2	'SEC24D	Isoform 2 of Protein transport protein Sec24D OS=Homo sapiens OX	0	10.491	4.549855
High	Q13144	'EIF2B5	Translation initiation factor eIF-2B subunit epsilon OS=Homo sapien	0	10.484	9.431345
High	Q15477	'SKIV2L	Helicase SKI2W OS=Homo sapiens OX=9606 GN=SKIV2L PE=1 SV=3	0	10.444	5.617978
High	Q14914	'PTGR1	Prostaglandin reductase 1 OS=Homo sapiens OX=9606 GN=PTGR1 P	0	10.437	20.36474
High	O95834-3	'EML2	Isoform 3 of Echinoderm microtubule-associated protein-like 2 OS=I	0	10.436	5.176471
High	O15085-2	'ARHGEF11	Isoform 2 of Rho guanine nucleotide exchange factor 11 OS=Homo s	0	10.434	3.521127
High	Q9NSB2	'KRT84	Keratin, type II cuticular Hb4 OS=Homo sapiens OX=9606 GN=KRT84	0	10.426	6.5
High	Q99569	'PKP4	Plakophilin-4 OS=Homo sapiens OX=9606 GN=PKP4 PE=1 SV=2	0	10.419	6.291946
High	Q96F45	'ZNF503	Zinc finger protein 503 OS=Homo sapiens OX=9606 GN=ZNF503 PE=	0	10.388	7.585139
High	P0DME0	'SETSIP	Protein SETSIP OS=Homo sapiens OX=9606 GN=SETSIP PE=1 SV=1	0	10.385	12.25166
High	Q15628	'TRADD	Tumor necrosis factor receptor type 1-associated DEATH domain pr	0	10.372	8.974359
High	Q10471	'GALNT2	Polypeptide N-acetylgalactosaminyltransferase 2 OS=Homo sapiens	0	10.35	7.180385
High	P43351	'RAD52	DNA repair protein RAD52 homolog OS=Homo sapiens OX=9606 GN	0	10.34	16.98565
High	Q13610	'PWP1	Periodic tryptophan protein 1 homolog OS=Homo sapiens OX=9606	0	10.327	11.77645
High	Q15363	'TMED2	Transmembrane emp24 domain-containing protein 2 OS=Homo sap	0	10.326	18.40796
High	P32320	'CDA	Cytidine deaminase OS=Homo sapiens OX=9606 GN=CDA PE=1 SV=2	0	10.318	40.41096
High	Q9H9Q4	'NHEJ1	Non-homologous end-joining factor 1 OS=Homo sapiens OX=9606 G	0	10.318	13.71237
High	Q9HCE5	'METTL14	N6-adenosine-methyltransferase non-catalytic subunit OS=Homo sa	0	10.309	6.578947
High	P61966	'AP1S1	AP-1 complex subunit sigma-1A OS=Homo sapiens OX=9606 GN=AP	0	10.297	15.82278
High	Q9Y3B9	'RRP15	RRP15-like protein OS=Homo sapiens OX=9606 GN=RRP15 PE=1 SV=	0	10.293	10.99291
High	Q96L58	'B3GALT6	Beta-1,3-galactosyltransferase 6 OS=Homo sapiens OX=9606 GN=B3	0	10.288	9.422492
High	Q8N2M8	'CLASRP	CLK4-associating serine/arginine rich protein OS=Homo sapiens OX=	0	10.284	5.192878
High	Q8N163	'CCAR2	Cell cycle and apoptosis regulator protein 2 OS=Homo sapiens OX=9	0	10.27	4.983749
High	Q9BYX2	'TBC1D2	TBC1 domain family member 2A OS=Homo sapiens OX=9606 GN=TB	0	10.267	8.081897
High	O95861	'BPNT1	3'(2'),5'-bisphosphate nucleotidase 1 OS=Homo sapiens OX=9606 GI	0	10.211	12.33766
High	Q9H089	'LSG1	Large subunit GTPase 1 homolog OS=Homo sapiens OX=9606 GN=LS	0	10.193	8.054711
High	P21397	'MAOA	Amine oxidase [flavin-containing] A OS=Homo sapiens OX=9606 GN	0	10.183	7.020873
High	Q32MZ4	'LRRFIP1	Leucine-rich repeat flightless-interacting protein 1 OS=Homo sapien	0	10.175	6.930693
High	Q8WUP2-	'FBLIM1	Isoform 2 of Filamin-binding LIM protein 1 OS=Homo sapiens OX=96	0	10.161	9.893048
High	Q9BRK5	'SDF4	45 kDa calcium-binding protein OS=Homo sapiens OX=9606 GN=SDF	0	10.157	11.32597
High	Q96J01	'THOC3	THO complex subunit 3 OS=Homo sapiens OX=9606 GN=THOC3 PE=	0	10.155	10.25641
High	P11233	'RALA	Ras-related protein Ral-A OS=Homo sapiens OX=9606 GN=RALA PE=	0	10.119	16.01942
High	P67812-3	'SEC11A	Isoform 3 of Signal peptidase complex catalytic subunit SEC11A OS=	0	10.113	10.81081
High	Q9NYY8	'FASTKD2	FAST kinase domain-containing protein 2, mitochondrial OS=Homo s	0	10.111	6.901408
High	Q9H0U3	'MAGT1	Magnesium transporter protein 1 OS=Homo sapiens OX=9606 GN=M	0	10.077	8.059701
High	P30519	'HMOX2	Heme oxygenase 2 OS=Homo sapiens OX=9606 GN=HMOX2 PE=1 SV	0	10.059	16.77215
High	Q6GPH4	'XAF1	XIAP-associated factor 1 OS=Homo sapiens OX=9606 GN=XAF1 PE=1	0	10.041	14.28571
High	Q9BTY7	'HGH1	Protein HGH1 homolog OS=Homo sapiens OX=9606 GN=HGH1 PE=1	0	10.026	9.230769
High	Q96GD0	'PDXP	Pyridoxal phosphate phosphatase OS=Homo sapiens OX=9606 GN=F	0	10.025	14.18919
High	Q9H6T0	'ESRP2	Epithelial splicing regulatory protein 2 OS=Homo sapiens OX=9606 C	0	10.019	7.015131
High	Q9H5V8	'CDCP1	CUB domain-containing protein 1 OS=Homo sapiens OX=9606 GN=C	0	10.018	3.4689
High	Q8IWS0	'PHF6	PHD finger protein 6 OS=Homo sapiens OX=9606 GN=PHF6 PE=1 SV	0	10.006	10.68493
High	P09132	'SRP19	Signal recognition particle 19 kDa protein OS=Homo sapiens OX=960	0	10.005	18.75
High	P49959-3	'MRE11	Isoform 3 of Double-strand break repair protein MRE11 OS=Homo s	0	10.001	5.907173
High	Q53EL6	'PDCD4	Programmed cell death protein 4 OS=Homo sapiens OX=9606 GN=P	0	9.972	8.102345
High	P17174	'GOT1	Aspartate aminotransferase, cytoplasmic OS=Homo sapiens OX=960	0	9.955	9.200969
High	Q9H1K4	'SLC25A18	Mitochondrial glutamate carrier 2 OS=Homo sapiens OX=9606 GN=S	0	9.926	13.01587
High	O43399-7	'TPD52L2	Isoform 7 of Tumor protein D54 OS=Homo sapiens OX=9606 GN=TP	0	9.926	18.77729
High	P02810	'PRH1	Salivary acidic proline-rich phosphoprotein 1/2 OS=Homo sapiens O	0	9.914	26.50602
High	O96000	'NDUFB10	NADH dehydrogenase [ubiquinone] 1 beta subcomplex subunit 10 C	0	9.913	20.93023
High	Q9H2W6	'MRPL46	39S ribosomal protein L46, mitochondrial OS=Homo sapiens OX=960	0	9.899	13.26165
High	Q9NNW5	'WDR6	WD repeat-containing protein 6 OS=Homo sapiens OX=9606 GN=WI	0	9.893	3.835861
High	Q9Y4W6	'AFG3L2	AFG3-like protein 2 OS=Homo sapiens OX=9606 GN=AFG3L2 PE=1 S	0	9.89	4.76788
High	Q9UBB9	'TFIP11	Tuftelin-interacting protein 11 OS=Homo sapiens OX=9606 GN=TFIP	0	9.881	5.854241
High	Q9NP79	'VTA1	Vacuolar protein sorting-associated protein VTA1 homolog OS=Hom	0	9.879	15.30945
High	Q7Z2E3	'APTX	Aprataxin OS=Homo sapiens OX=9606 GN=APTX PE=1 SV=2	0	9.857	11.51685
High	Q5C9Z4	'NOM1	Nucleolar MIF4G domain-containing protein 1 OS=Homo sapiens OX	0	9.847	4.651163
High	P61769	'B2M	Beta-2-microglobulin OS=Homo sapiens OX=9606 GN=B2M PE=1 SV	0	9.84	35.29412
High	Q13643	'FHL3	Four and a half LIM domains protein 3 OS=Homo sapiens OX=9606 C	0	9.821	10
High	Q01415	'GALK2	N-acetylgalactosamine kinase OS=Homo sapiens OX=9606 GN=GALK	0	9.818	8.515284
High	Q6P9B6	'TLDC1	TLD domain-containing protein 1 OS=Homo sapiens OX=9606 GN=TI	0	9.797	10.74561
High	O14896	'IRF6	Interferon regulatory factor 6 OS=Homo sapiens OX=9606 GN=IRF6	0	9.79	11.1349
High	Q9C0D5	'TANC1	Protein TANC1 OS=Homo sapiens OX=9606 GN=TANC1 PE=1 SV=3	0	9.789	3.385277
High	Q8TAT6-2	'NPLOC4	Isoform 2 of Nuclear protein localization protein 4 homolog OS=Hor	0	9.76	4.700162
High	Q9BTZ2	'DHRS4	Dehydrogenase/reductase SDR family member 4 OS=Homo sapiens	0	9.746	11.15108
High	Q9NVX2	'NLE1	Notchless protein homolog 1 OS=Homo sapiens OX=9606 GN=NLE1	0	9.732	7.42268
High	Q9H299	'SH3BGRL3	SH3 domain-binding glutamic acid-rich-like protein 3 OS=Homo sapi	0	9.717	31.1828
High	Q96QR8	'PURB	Transcriptional activator protein Pur-beta OS=Homo sapiens OX=96	0	9.711	16.66667
High	Q9NYV4	'CDK12	Cyclin-dependent kinase 12 OS=Homo sapiens OX=9606 GN=CDK12	0	9.704	3.087248
High	Q9P2K8	'EIF2AK4	eIF-2-alpha kinase GCN2 OS=Homo sapiens OX=9606 GN=EIF2AK4 P	0	9.688	3.759854
High	Q9Y3A4	'RRP7A	Ribosomal RNA-processing protein 7 homolog A OS=Homo sapiens (	0	9.688	14.64286
High	Q9NS69	'TOMM22	Mitochondrial import receptor subunit TOM22 homolog OS=Homo s	0	9.679	26.05634
High	P51798	'CLCN7	H(+)/Cl(-) exchange transporter 7 OS=Homo sapiens OX=9606 GN=C	0	9.669	6.086957
High	Q9UJW0	'DCTN4	Dynactin subunit 4 OS=Homo sapiens OX=9606 GN=DCTN4 PE=1 SV	0	9.641	11.30435
High	Q66K14	'TBC1D9B	TBC1 domain family member 9B OS=Homo sapiens OX=9606 GN=TB	0	9.603	2.88
High	O43447	'PPIH	Peptidyl-prolyl cis-trans isomerase H OS=Homo sapiens OX=9606 GN	0	9.591	22.59887
High	P52565	'ARHGDIA	Rho GDP-dissociation inhibitor 1 OS=Homo sapiens OX=9606 GN=AF	0	9.579	22.54902
High	Q06323-2	'PSME1	Isoform 2 of Proteasome activator complex subunit 1 OS=Homo sap	0	9.518	13.2
High	P51970	'NDUFA8	NADH dehydrogenase [ubiquinone] 1 alpha subcomplex subunit 8 C	0	9.516	25
High	Q02388	'COL7A1	Collagen alpha-1(VII) chain OS=Homo sapiens OX=9606 GN=COL7A1	0	9.51	2.038043
High	Q9NQT8	'KIF13B	Kinesin-like protein KIF13B OS=Homo sapiens OX=9606 GN=KIF13B	0	9.496	2.135816
High	A0A0C4D	'IGKV3D-20	Immunoglobulin kappa variable 3D-20 OS=Homo sapiens OX=9606 C	0	9.482	21.55172
High	Q7L5N1	'COPS6	COP9 signalosome complex subunit 6 OS=Homo sapiens OX=9606 G	0	9.468	11.92661
High	Q15532	'SS18	Protein SSXT OS=Homo sapiens OX=9606 GN=SS18 PE=1 SV=3	0	9.466	7.177033
High	Q53F19	'NCBP3	Nuclear cap-binding protein subunit 3 OS=Homo sapiens OX=9606 C	0	9.46	6.290323
High	Q9BW04	'SARG	Specifically androgen-regulated gene protein OS=Homo sapiens OX=	0	9.431	9.650582
High	Q9BZL6-3	'PRKD2	Isoform 3 of Serine/threonine-protein kinase D2 OS=Homo sapiens (	0	9.425	6.306306
High	P52788	'SMS	Spermine synthase OS=Homo sapiens OX=9606 GN=SMS PE=1 SV=2	0	9.41	7.650273
High	P04233	'CD74	HLA class II histocompatibility antigen gamma chain OS=Homo sapie	0	9.409	9.459459
High	P46821	'MAP1B	Microtubule-associated protein 1B OS=Homo sapiens OX=9606 GN=	0	9.404	1.134522
High	Q9NXR1-	'NDE1	Isoform 2 of Nuclear distribution protein nudE homolog 1 OS=Homo	0	9.399	11.27168
High	O00186	'STXBP3	Syntaxin-binding protein 3 OS=Homo sapiens OX=9606 GN=STXBP3	0	9.376	6.587838
High	Q9Y508	'RNF114	E3 ubiquitin-protein ligase RNF114 OS=Homo sapiens OX=9606 GN=	0	9.374	26.31579
High	Q9Y3A2	'UTP11	Probable U3 small nucleolar RNA-associated protein 11 OS=Homo s	0	9.372	13.83399
High	Q13546	'RIPK1	Receptor-interacting serine/threonine-protein kinase 1 OS=Homo sa	0	9.359	7.898659
High	Q96GK7	'FAHD2A	Fumarylacetoacetate hydrolase domain-containing protein 2A OS=H	0	9.345	9.872611
High	Q9NSI2	'FAM207A	Protein FAM207A OS=Homo sapiens OX=9606 GN=FAM207A PE=1 S	0	9.302	16.08696
High	P41214	'EIF2D	Eukaryotic translation initiation factor 2D OS=Homo sapiens OX=960	0	9.28	6.678082
High	Q96ME1	'FBXL18	F-box/LRR-repeat protein 18 OS=Homo sapiens OX=9606 GN=FBXL1	0	9.262	8.447205
High	Q969U7	'PSMG2	Proteasome assembly chaperone 2 OS=Homo sapiens OX=9606 GN=	0	9.252	10.98485
High	A0AVT1	'UBA6	Ubiquitin-like modifier-activating enzyme 6 OS=Homo sapiens OX=9	0	9.245	3.041825
High	P62312	'LSM6	U6 snRNA-associated Sm-like protein LSm6 OS=Homo sapiens OX=9	0	9.217	33.75
High	Q9BRA2	'TXNDC17	Thioredoxin domain-containing protein 17 OS=Homo sapiens OX=96	0	9.216	26.82927
High	Q9BX40	'LSM14B	Protein LSM14 homolog B OS=Homo sapiens OX=9606 GN=LSM14B	0	9.209	12.72727
High	Q6XQN6	'NAPRT	Nicotinate phosphoribosyltransferase OS=Homo sapiens OX=9606 G	0	9.207	6.133829
High	Q6NUK1	'SLC25A24	Calcium-binding mitochondrial carrier protein SCaMC-1 OS=Homo s	0	9.204	9.433962
High	Q9Y5Q8-3	'GTF3C5	Isoform 3 of General transcription factor 3C polypeptide 5 OS=Hom	0	9.197	11.02662
High	P14550	'AKR1A1	Alcohol dehydrogenase [NADP(+)] OS=Homo sapiens OX=9606 GN=A	0	9.153	10.46154
High	P51553	'IDH3G	Isocitrate dehydrogenase [NAD] subunit gamma, mitochondrial OS=	0	9.139	12.9771
High	Q5RKV6	'EXOSC6	Exosome complex component MTR3 OS=Homo sapiens OX=9606 GN	0	9.135	14.70588
High	Q92552-2	'MRPS27	Isoform 2 of 28S ribosomal protein S27, mitochondrial OS=Homo sa	0	9.129	10.74766
High	P09758	'TACSTD2	Tumor-associated calcium signal transducer 2 OS=Homo sapiens OX	0	9.119	8.668731
High	Q8TAQ2	'SMARCC2	SWI/SNF complex subunit SMARCC2 OS=Homo sapiens OX=9606 GN	0	9.103	3.706755
High	P14854	'COX6B1	Cytochrome c oxidase subunit 6B1 OS=Homo sapiens OX=9606 GN=	0	9.088	26.74419
High	P30085	'CMPK1	UMP-CMP kinase OS=Homo sapiens OX=9606 GN=CMPK1 PE=1 SV=3	0	9.074	16.83673
High	Q96FS4	'SIPA1	Signal-induced proliferation-associated protein 1 OS=Homo sapiens	0	9.074	5.950096
High	O60563	'CCNT1	Cyclin-T1 OS=Homo sapiens OX=9606 GN=CCNT1 PE=1 SV=1	0	9.074	7.024793
High	Q9NVH1	'DNAJC11	DnaJ homolog subfamily C member 11 OS=Homo sapiens OX=9606 (	0	9.055	6.440072
High	P53985	'SLC16A1	Monocarboxylate transporter 1 OS=Homo sapiens OX=9606 GN=SLC	0	9.03	6.6
High	E9PRG8	'C11orf98	Uncharacterized protein C11orf98 OS=Homo sapiens OX=9606 GN=(	0	9.024	17.21311
High	Q9UPQ0	'LIMCH1	LIM and calponin homology domains-containing protein 1 OS=Homo	0	9.006	4.893813
High	O95486	'SEC24A	Protein transport protein Sec24A OS=Homo sapiens OX=9606 GN=S	0	8.977	2.561757
High	P50750-2	'CDK9	Isoform 2 of Cyclin-dependent kinase 9 OS=Homo sapiens OX=9606	0	8.969	8.384458
High	O14641	'DVL2	Segment polarity protein dishevelled homolog DVL-2 OS=Homo sapi	0	8.965	5.298913
High	Q9Y2K7	'KDM2A	Lysine-specific demethylase 2A OS=Homo sapiens OX=9606 GN=KDI	0	8.955	4.04475
High	Q96N66	'MBOAT7	Lysophospholipid acyltransferase 7 OS=Homo sapiens OX=9606 GN=	0	8.951	6.779661
High	P08579	'SNRPB2	U2 small nuclear ribonucleoprotein B'' OS=Homo sapiens OX=9606 (	0	8.946	12.88889
High	P06312	'IGKV4-1	Immunoglobulin kappa variable 4-1 OS=Homo sapiens OX=9606 GN	0	8.917	29.75207
High	Q99836-6	'MYD88	Isoform 6 of Myeloid differentiation primary response protein MyDl	0	8.916	11.84211
High	Q8WUY1	'THEM6	Protein THEM6 OS=Homo sapiens OX=9606 GN=THEM6 PE=1 SV=2	0	8.9	16.82692
High	Q16527	'CSRP2	Cysteine and glycine-rich protein 2 OS=Homo sapiens OX=9606 GN=	0	8.897	15.54404
High	P51911	'CNN1	Calponin-1 OS=Homo sapiens OX=9606 GN=CNN1 PE=1 SV=2	0	8.884	10.43771
High	P06400	'RB1	Retinoblastoma-associated protein OS=Homo sapiens OX=9606 GN=	0	8.875	4.741379
High	Q96T76-8	'MMS19	Isoform 5 of MMS19 nucleotide excision repair protein homolog OS	0	8.861	3.139867
High	P53004	'BLVRA	Biliverdin reductase A OS=Homo sapiens OX=9606 GN=BLVRA PE=1	0	8.839	15.87838
High	Q9NVV4-	'MTPAP	Isoform 2 of Poly(A) RNA polymerase, mitochondrial OS=Homo sapi	0	8.833	5.196629
High	O00505	'KPNA3	Importin subunit alpha-4 OS=Homo sapiens OX=9606 GN=KPNA3 PE	0	8.829	7.101727
High	Q9UHA4	'LAMTOR3	Ragulator complex protein LAMTOR3 OS=Homo sapiens OX=9606 G	0	8.828	30.64516
High	P61009	'SPCS3	Signal peptidase complex subunit 3 OS=Homo sapiens OX=9606 GN=	0	8.819	15.55556
High	Q8IVS2	'MCAT	Malonyl-CoA-acyl carrier protein transacylase, mitochondrial OS=Hc	0	8.799	5.641026
High	P56524	'HDAC4	Histone deacetylase 4 OS=Homo sapiens OX=9606 GN=HDAC4 PE=1	0	8.778	3.228782
High	Q9UDW1	'UQCR10	Cytochrome b-c1 complex subunit 9 OS=Homo sapiens OX=9606 GN	0	8.778	38.09524
High	P06703	'S100A6	Protein S100-A6 OS=Homo sapiens OX=9606 GN=S100A6 PE=1 SV=1	0	8.772	50
High	P01876	'IGHA1	Immunoglobulin heavy constant alpha 1 OS=Homo sapiens OX=960	0	8.762	7.932011
High	Q9NVJ2	'ARL8B	ADP-ribosylation factor-like protein 8B OS=Homo sapiens OX=9606	0	8.756	25.26882
High	Q93034	'CUL5	Cullin-5 OS=Homo sapiens OX=9606 GN=CUL5 PE=1 SV=4	0	8.752	5.897436
High	Q9HCC0	'MCCC2	Methylcrotonoyl-CoA carboxylase beta chain, mitochondrial OS=Hor	0	8.73	9.413854
High	P00966	'ASS1	Argininosuccinate synthase OS=Homo sapiens OX=9606 GN=ASS1 PI	0	8.729	9.223301
High	P11234-2	'RALB	Isoform 2 of Ras-related protein Ral-B OS=Homo sapiens OX=9606 G	0	8.722	14.91228
High	P54577	'YARS	Tyrosine--tRNA ligase, cytoplasmic OS=Homo sapiens OX=9606 GN=	0	8.719	6.628788
High	Q96IZ0	'PAWR	PRKC apoptosis WT1 regulator protein OS=Homo sapiens OX=9606 (	0	8.718	11.76471
High	Q9Y5L4	'TIMM13	Mitochondrial import inner membrane translocase subunit Tim13 O	0	8.686	25.26316
High	Q9H2M9	'RAB3GAP2	Rab3 GTPase-activating protein non-catalytic subunit OS=Homo sap	0	8.662	3.4458
High	Q8IY67-2	'RAVER1	Isoform 2 of Ribonucleoprotein PTB-binding 1 OS=Homo sapiens OX	0	8.652	7.44249
High	Q13868	'EXOSC2	Exosome complex component RRP4 OS=Homo sapiens OX=9606 GN	0	8.652	14.33447
High	Q9Y399	'MRPS2	28S ribosomal protein S2, mitochondrial OS=Homo sapiens OX=960	0	8.632	12.83784
High	P29558	'RBMS1	RNA-binding motif, single-stranded-interacting protein 1 OS=Homo	0	8.606	7.881773
High	Q05932	'FPGS	Folylpolyglutamate synthase, mitochondrial OS=Homo sapiens OX=9	0	8.593	5.110733
High	P23469	'PTPRE	Receptor-type tyrosine-protein phosphatase epsilon OS=Homo sapi	0	8.592	5.571429
High	P14406	'COX7A2	Cytochrome c oxidase subunit 7A2, mitochondrial OS=Homo sapiens	0	8.588	27.71084
High	Q5T3I0-3	'GPATCH4	Isoform 3 of G patch domain-containing protein 4 OS=Homo sapiens	0	8.571	11.08647
High	Q9UNS2	'COPS3	COP9 signalosome complex subunit 3 OS=Homo sapiens OX=9606 G	0	8.563	7.328605
High	P28161	'GSTM2	Glutathione S-transferase Mu 2 OS=Homo sapiens OX=9606 GN=GS	0	8.56	11.92661
High	P57735	'RAB25	Ras-related protein Rab-25 OS=Homo sapiens OX=9606 GN=RAB25	0	8.553	12.67606
High	O43172	'PRPF4	U4/U6 small nuclear ribonucleoprotein Prp4 OS=Homo sapiens OX=	0	8.553	9.195402
High	Q5VZE5	'NAA35	N-alpha-acetyltransferase 35, NatC auxiliary subunit OS=Homo sapi	0	8.54	4.275862
High	Q5VTL8	'PRPF38B	Pre-mRNA-splicing factor 38B OS=Homo sapiens OX=9606 GN=PRPF	0	8.537	7.142857
High	Q9BRR8	'GPATCH1	G patch domain-containing protein 1 OS=Homo sapiens OX=9606 GI	0	8.522	3.86681
High	O60306	'AQR	RNA helicase aquarius OS=Homo sapiens OX=9606 GN=AQR PE=1 SV	0	8.511	2.693603
High	O14874	'BCKDK	[3-methyl-2-oxobutanoate dehydrogenase [lipoamide]] kinase, mitc	0	8.502	7.281553
High	P04080	'CSTB	Cystatin-B OS=Homo sapiens OX=9606 GN=CSTB PE=1 SV=2	0	8.491	24.4898
High	Q01970	'PLCB3	1-phosphatidylinositol 4,5-bisphosphate phosphodiesterase beta-3	0	8.488	4.619125
High	Q14116	'IL18	Interleukin-18 OS=Homo sapiens OX=9606 GN=IL18 PE=1 SV=1	0	8.48	13.4715
High	Q9UI30	'TRMT112	Multifunctional methyltransferase subunit TRM112-like protein OS=	0	8.451	21.6
High	Q9H2G2	'SLK	STE20-like serine/threonine-protein kinase OS=Homo sapiens OX=9	0	8.434	2.267206
High	Q96P47-4	'AGAP3	Isoform 4 of Arf-GAP with GTPase, ANK repeat and PH domain-conta	0	8.409	4.17124
High	Q96LD4	'TRIM47	Tripartite motif-containing protein 47 OS=Homo sapiens OX=9606 G	0	8.406	8.934169
High	P15260	'IFNGR1	Interferon gamma receptor 1 OS=Homo sapiens OX=9606 GN=IFNG	0	8.406	9.406953
High	Q92973	'TNPO1	Transportin-1 OS=Homo sapiens OX=9606 GN=TNPO1 PE=1 SV=2	0	8.402	5.122494
High	Q99961	'SH3GL1	Endophilin-A2 OS=Homo sapiens OX=9606 GN=SH3GL1 PE=1 SV=1	0	8.384	8.695652
High	P11717	'IGF2R	Cation-independent mannose-6-phosphate receptor OS=Homo sapi	0	8.38	1.886792
High	P10155	'TROVE2	60 kDa SS-A/Ro ribonucleoprotein OS=Homo sapiens OX=9606 GN=	0	8.365	4.460967
High	Q9P2F8	'SIPA1L2	Signal-induced proliferation-associated 1-like protein 2 OS=Homo sa	0	8.36	1.800232
High	O15067	'PFAS	Phosphoribosylformylglycinamidine synthase OS=Homo sapiens OX=	0	8.337	3.139013
High	Q6PGP7	'TTC37	Tetratricopeptide repeat protein 37 OS=Homo sapiens OX=9606 GN	0	8.328	2.429668
High	P13995	'MTHFD2	Bifunctional methylenetetrahydrofolate dehydrogenase/cyclohydro	0	8.312	15.42857
High	P60900	'PSMA6	Proteasome subunit alpha type-6 OS=Homo sapiens OX=9606 GN=P	0	8.294	10.1626
High	Q9H3M7	'TXNIP	Thioredoxin-interacting protein OS=Homo sapiens OX=9606 GN=TXI	0	8.261	16.36829
High	Q92759	'GTF2H4	General transcription factor IIH subunit 4 OS=Homo sapiens OX=960	0	8.243	7.359307
High	P53990-5	'IST1	Isoform 5 of IST1 homolog OS=Homo sapiens OX=9606 GN=IST1	0	8.226	7.124011
High	Q92733	'PRCC	Proline-rich protein PRCC OS=Homo sapiens OX=9606 GN=PRCC PE=	0	8.221	14.86762
High	Q9P258	'RCC2	Protein RCC2 OS=Homo sapiens OX=9606 GN=RCC2 PE=1 SV=2	0	8.216	8.429119
High	Q9GZM7	'TINAGL1	Tubulointerstitial nephritis antigen-like OS=Homo sapiens OX=9606	0	8.212	8.351178
High	Q9NTJ3	'SMC4	Structural maintenance of chromosomes protein 4 OS=Homo sapien	0	8.205	2.406832
High	Q03164-3	'KMT2A	Isoform 3 of Histone-lysine N-methyltransferase 2A OS=Homo sapie	0	8.194	1.409869
High	O60237-6	'PPP1R12B	Isoform 6 of Protein phosphatase 1 regulatory subunit 12B OS=Hom	0	8.193	2.396932
High	P61764-2	'STXBP1	Isoform 2 of Syntaxin-binding protein 1 OS=Homo sapiens OX=9606	0	8.191	7.462687
High	P43353	'ALDH3B1	Aldehyde dehydrogenase family 3 member B1 OS=Homo sapiens OX	0	8.186	4.487179
High	Q9NUI1	'DECR2	Peroxisomal 2,4-dienoyl-CoA reductase OS=Homo sapiens OX=9606	0	8.186	10.27397
High	Q96EY7	'PTCD3	Pentatricopeptide repeat domain-containing protein 3, mitochondri	0	8.181	5.079826
High	Q96JP5	'ZFP91	E3 ubiquitin-protein ligase ZFP91 OS=Homo sapiens OX=9606 GN=ZI	0	8.177	8.070175
High	Q8WV41	'SNX33	Sorting nexin-33 OS=Homo sapiens OX=9606 GN=SNX33 PE=1 SV=1	0	8.122	6.968641
High	Q9NUQ7	'UFSP2	Ufm1-specific protease 2 OS=Homo sapiens OX=9606 GN=UFSP2 PE	0	8.117	9.808102
High	Q9BX68	'HINT2	Histidine triad nucleotide-binding protein 2, mitochondrial OS=Hom	0	8.075	24.53988
High	Q9HCN8	'SDF2L1	Stromal cell-derived factor 2-like protein 1 OS=Homo sapiens OX=96	0	8.051	13.57466
High	P06493	'CDK1	Cyclin-dependent kinase 1 OS=Homo sapiens OX=9606 GN=CDK1 PE	0	8.031	10.10101
High	Q96A26	'FAM162A	Protein FAM162A OS=Homo sapiens OX=9606 GN=FAM162A PE=1 S	0	8.019	12.33766
High	P04040	'CAT	Catalase OS=Homo sapiens OX=9606 GN=CAT PE=1 SV=3	0	8.015	8.159393
High	P49643	'PRIM2	DNA primase large subunit OS=Homo sapiens OX=9606 GN=PRIM2 I	0	8.014	7.465619
High	Q9HB19	'PLEKHA2	Pleckstrin homology domain-containing family A member 2 OS=Hon	0	8.009	8.235294
High	Q9NPF4	'OSGEP	Probable tRNA N6-adenosine threonylcarbamoyltransferase OS=Ho	0	7.997	15.8209
High	Q7L4I2	'RSRC2	Arginine/serine-rich coiled-coil protein 2 OS=Homo sapiens OX=960	0	7.985	10.13825
High	P16220	'CREB1	Cyclic AMP-responsive element-binding protein 1 OS=Homo sapiens	0	7.967	6.158358
High	O95248-4	'SBF1	Isoform 4 of Myotubularin-related protein 5 OS=Homo sapiens OX=	0	7.955	1.689546
High	P45973	'CBX5	Chromobox protein homolog 5 OS=Homo sapiens OX=9606 GN=CBX	0	7.945	14.13613
High	Q9H4L4	'SENP3	Sentrin-specific protease 3 OS=Homo sapiens OX=9606 GN=SENP3 F	0	7.942	8.536585
High	Q9H8Y8-3	'GORASP2	Isoform 3 of Golgi reassembly-stacking protein 2 OS=Homo sapiens	0	7.937	5.387931
High	O15118	'NPC1	NPC intracellular cholesterol transporter 1 OS=Homo sapiens OX=96	0	7.933	3.99061
High	A0A0C4D	'IGKV1-9	Immunoglobulin kappa variable 1-9 OS=Homo sapiens OX=9606 GN	0	7.932	29.05983
High	Q9BV44	'THUMPD3	THUMP domain-containing protein 3 OS=Homo sapiens OX=9606 GI	0	7.919	4.33925
High	Q9UNF1	'MAGED2	Melanoma-associated antigen D2 OS=Homo sapiens OX=9606 GN=M	0	7.917	11.71617
High	P22307	'SCP2	Non-specific lipid-transfer protein OS=Homo sapiens OX=9606 GN=S	0	7.913	5.667276
High	Q3YEC7-2	'RABL6	Isoform 2 of Rab-like protein 6 OS=Homo sapiens OX=9606 GN=RAB	0	7.907	6.986301
High	P25685	'DNAJB1	DnaJ homolog subfamily B member 1 OS=Homo sapiens OX=9606 G	0	7.882	9.705882
High	Q9Y6M7-	'SLC4A7	Isoform 7 of Sodium bicarbonate cotransporter 3 OS=Homo sapiens	0	7.868	1.826847
High	A6NFI3	'ZNF316	Zinc finger protein 316 OS=Homo sapiens OX=9606 GN=ZNF316 PE=	0	7.84	4.183267
High	Q9UHV9	'PFDN2	Prefoldin subunit 2 OS=Homo sapiens OX=9606 GN=PFDN2 PE=1 SV	0	7.833	13.63636
High	P08581-2	'MET	Isoform 2 of Hepatocyte growth factor receptor OS=Homo sapiens C	0	7.824	3.267045
High	Q9NP64	'ZCCHC17	Nucleolar protein of 40 kDa OS=Homo sapiens OX=9606 GN=ZCCHC	0	7.808	15.76763
High	P04049-2	'RAF1	Isoform 2 of RAF proto-oncogene serine/threonine-protein kinase C	0	7.806	6.736527
High	Q96S97	'MYADM	Myeloid-associated differentiation marker OS=Homo sapiens OX=96	0	7.804	7.142857
High	P10915	'HAPLN1	Hyaluronan and proteoglycan link protein 1 OS=Homo sapiens OX=9	0	7.802	10.45198
High	O14908	'GIPC1	PDZ domain-containing protein GIPC1 OS=Homo sapiens OX=9606 C	0	7.795	17.41742
High	O15381	'NVL	Nuclear valosin-containing protein-like OS=Homo sapiens OX=9606	0	7.788	5.490654
High	P52756	'RBM5	RNA-binding protein 5 OS=Homo sapiens OX=9606 GN=RBM5 PE=1	0	7.781	3.312883
High	Q9BWE0-	'REPIN1	Isoform 2 of Replication initiator 1 OS=Homo sapiens OX=9606 GN=	0	7.777	4.967949
High	P13693	'TPT1	Translationally-controlled tumor protein OS=Homo sapiens OX=960	0	7.775	15.69767
High	P48739-3	'PITPNB	Isoform 3 of Phosphatidylinositol transfer protein beta isoform OS=I	0	7.772	15.01832
High	P61077-3	'UBE2D3	Isoform 3 of Ubiquitin-conjugating enzyme E2 D3 OS=Homo sapiens	0	7.768	19.46309
High	P52701	'MSH6	DNA mismatch repair protein Msh6 OS=Homo sapiens OX=9606 GN	0	7.768	3.676471
High	P17676	'CEBPB	CCAAT/enhancer-binding protein beta OS=Homo sapiens OX=9606 (	0	7.75	9.275362
High	Q8NEJ9	'NGDN	Neuroguidin OS=Homo sapiens OX=9606 GN=NGDN PE=1 SV=1	0	7.713	8.888889
High	Q13190	'STX5	Syntaxin-5 OS=Homo sapiens OX=9606 GN=STX5 PE=1 SV=2	0	7.697	8.169014
High	Q08188	'TGM3	Protein-glutamine gamma-glutamyltransferase E OS=Homo sapiens	0	7.694	9.090909
High	Q70UQ0	'IKBIP	Inhibitor of nuclear factor kappa-B kinase-interacting protein OS=Hc	0	7.679	5.714286
High	Q7Z6I8	'C5orf24	UPF0461 protein C5orf24 OS=Homo sapiens OX=9606 GN=C5orf24 I	0	7.671	22.34043
High	Q9HCD6-	'TANC2	Isoform 2 of Protein TANC2 OS=Homo sapiens OX=9606 GN=TANC2	0	7.667	2.35
High	Q9Y2W2	'WBP11	WW domain-binding protein 11 OS=Homo sapiens OX=9606 GN=WI	0	7.661	7.020281
High	Q14344	'GNA13	Guanine nucleotide-binding protein subunit alpha-13 OS=Homo sap	0	7.657	6.896552
High	P30414	'NKTR	NK-tumor recognition protein OS=Homo sapiens OX=9606 GN=NKTF	0	7.652	3.214774
High	P53680	'AP2S1	AP-2 complex subunit sigma OS=Homo sapiens OX=9606 GN=AP2S1	0	7.637	19.01408
High	P34896	'SHMT1	Serine hydroxymethyltransferase, cytosolic OS=Homo sapiens OX=9	0	7.625	4.761905
High	Q8WUD4	'CCDC12	Coiled-coil domain-containing protein 12 OS=Homo sapiens OX=960	0	7.623	26.50602
High	P13164	'IFITM1	Interferon-induced transmembrane protein 1 OS=Homo sapiens OX	0	7.616	29.6
High	O94804	'STK10	Serine/threonine-protein kinase 10 OS=Homo sapiens OX=9606 GN=	0	7.615	2.68595
High	P35270	'SPR	Sepiapterin reductase OS=Homo sapiens OX=9606 GN=SPR PE=1 SV	0	7.614	21.0728
High	Q9HD45	'TM9SF3	Transmembrane 9 superfamily member 3 OS=Homo sapiens OX=96	0	7.603	3.225806
High	O14907	'TAX1BP3	Tax1-binding protein 3 OS=Homo sapiens OX=9606 GN=TAX1BP3 PE	0	7.593	28.22581
High	Q86UK7	'ZNF598	E3 ubiquitin-protein ligase ZNF598 OS=Homo sapiens OX=9606 GN=	0	7.592	5.862832
High	O00743-3	'PPP6C	Isoform 3 of Serine/threonine-protein phosphatase 6 catalytic subu	0	7.565	9.94152
High	Q5VT06	'CEP350	Centrosome-associated protein 350 OS=Homo sapiens OX=9606 GN	0	7.564	1.636189
High	P62306	'SNRPF	Small nuclear ribonucleoprotein F OS=Homo sapiens OX=9606 GN=S	0	7.558	24.4186
High	Q8WUQ7	'CACTIN	Isoform 2 of Cactin OS=Homo sapiens OX=9606 GN=CACTIN	0	7.523	2.566845
High	Q9Y6D5	'ARFGEF2	Brefeldin A-inhibited guanine nucleotide-exchange protein 2 OS=Ho	0	7.523	1.904762
High	P04156	'PRNP	Major prion protein OS=Homo sapiens OX=9606 GN=PRNP PE=1 SV=	0	7.516	7.905138
High	Q02252	'ALDH6A1	Methylmalonate-semialdehyde dehydrogenase [acylating], mitocho	0	7.502	5.607477
High	Q9HBH5	'RDH14	Retinol dehydrogenase 14 OS=Homo sapiens OX=9606 GN=RDH14 F	0	7.498	9.821429
High	Q9Y3D3	'MRPS16	28S ribosomal protein S16, mitochondrial OS=Homo sapiens OX=960	0	7.482	18.9781
High	Q9BQ52	'ELAC2	Zinc phosphodiesterase ELAC protein 2 OS=Homo sapiens OX=9606	0	7.452	3.1477
High	Q5TCZ1	'SH3PXD2A	SH3 and PX domain-containing protein 2A OS=Homo sapiens OX=96	0	7.447	3.618711
High	Q14331	'FRG1	Protein FRG1 OS=Homo sapiens OX=9606 GN=FRG1 PE=1 SV=1	0	7.435	9.689922
High	P49821	'NDUFV1	NADH dehydrogenase [ubiquinone] flavoprotein 1, mitochondrial O	0	7.435	10.77586
High	Q9NUQ8	'ABCF3	ATP-binding cassette sub-family F member 3 OS=Homo sapiens OX=	0	7.426	5.641749
High	Q99567	'NUP88	Nuclear pore complex protein Nup88 OS=Homo sapiens OX=9606 G	0	7.405	5.937922
High	Q96GY0	'ZC2HC1A	Zinc finger C2HC domain-containing protein 1A OS=Homo sapiens O	0	7.405	10.15385
High	Q14008-3	'CKAP5	Isoform 3 of Cytoskeleton-associated protein 5 OS=Homo sapiens O	0	7.405	1.961746
High	Q96EY4	'TMA16	Translation machinery-associated protein 16 OS=Homo sapiens OX=	0	7.403	17.24138
High	Q8TD19	'NEK9	Serine/threonine-protein kinase Nek9 OS=Homo sapiens OX=9606 G	0	7.392	3.575077
High	P48507	'GCLM	Glutamate--cysteine ligase regulatory subunit OS=Homo sapiens OX	0	7.369	10.21898
High	P04432	'IGKV1D-39	Immunoglobulin kappa variable 1D-39 OS=Homo sapiens OX=9606 (	0	7.368	29.05983
High	Q96H79	'ZC3HAV1L	Zinc finger CCCH-type antiviral protein 1-like OS=Homo sapiens OX=	0	7.36	14.66667
High	Q5EBL8-2	'PDZD11	Isoform 2 of PDZ domain-containing protein 11 OS=Homo sapiens O	0	7.353	19.88304
High	O15446-2	'CD3EAP	Isoform 2 of DNA-directed RNA polymerase I subunit RPA34 OS=Hor	0	7.325	9.375
High	P42696	'RBM34	RNA-binding protein 34 OS=Homo sapiens OX=9606 GN=RBM34 PE=	0	7.31	7.906977
High	P80404	'ABAT	4-aminobutyrate aminotransferase, mitochondrial OS=Homo sapien	0	7.309	10.8
High	Q9H0R4	'HDHD2	Haloacid dehalogenase-like hydrolase domain-containing protein 2 (	0	7.307	16.21622
High	Q9Y4X5	'ARIH1	E3 ubiquitin-protein ligase ARIH1 OS=Homo sapiens OX=9606 GN=A	0	7.276	4.48833
High	O95302-3	'FKBP9	Isoform 3 of Peptidyl-prolyl cis-trans isomerase FKBP9 OS=Homo sa	0	7.262	4.173355
High	Q8NHP8	'PLBD2	Putative phospholipase B-like 2 OS=Homo sapiens OX=9606 GN=PLE	0	7.261	4.923599
High	O95630	'STAMBP	STAM-binding protein OS=Homo sapiens OX=9606 GN=STAMBP PE=	0	7.243	8.254717

High	Q9NZM5	' NOP53	Ribosome biogenesis protein NOP53 OS=Homo sapiens OX=9606 GN	0	7.237	6.903766	2	2	2	478	54.4	10.32		0.245
High	Q03188	' CENPC	Centromere protein C OS=Homo sapiens OX=9606 GN=CENPC PE=1	0	7.235	3.711559	3	3	3	943	106.8	9.39		0.126
High	Q7Z4R8	' C6orf120	UPF0669 protein C6orf120 OS=Homo sapiens OX=9606 GN=C6orf12	0	7.21	14.13613	1	5	1	191	20.8	4.84		1.154
High	P62875	' POLR2L	DNA-directed RNA polymerases I, II, and III subunit RPABC5 OS=Hon	0	7.197	29.85075	2	8	2	67	7.6	7.77		1.512
High	P19634	' SLC9A1	Sodium/hydrogen exchanger 1 OS=Homo sapiens OX=9606 GN=SLC	0	7.194	3.435583	2	2	2	815	90.7	7.21		0.155
High	A8MWD9	' SNRPGP15	Putative small nuclear ribonucleoprotein G-like protein 15 OS=Hom	0	7.194	25	2	7	2	76	8.5	8.84		1.512
High	Q15738	' NSDHL	Sterol-4-alpha-carboxylate 3-dehydrogenase, decarboxylating OS=H	0	7.187	10.72386	3	5	3	373	41.9	8.06		0.304
High	Q5T0W9	' FAM83B	Protein FAM83B OS=Homo sapiens OX=9606 GN=FAM83B PE=1 SV=1	0	7.187	5.934718	4	6	4	1011	114.7	8.97		0.157
High	Q9Y305-4	' ACOT9	Isoform 4 of Acyl-coenzyme A thioesterase 9, mitochondrial OS=Hor	0	7.175	10.04464	4	4	4	448	50.8	8.53		0.374
High	P17693	' HLA-G	HLA class I histocompatibility antigen, alpha chain G OS=Homo sapie	0	7.172	13.90533	3	4	1	338	38.2	5.69		0.245
High	Q9P2R7	' SUCLA2	Succinate--CoA ligase [ADP-forming] subunit beta, mitochondrial OS	0	7.167	9.50324	3	3	3	463	50.3	7.42		0.269
High	P08651	' NFIC	Nuclear factor 1 C-type OS=Homo sapiens OX=9606 GN=NFIC PE=1 S	0	7.158	10.03937	3	8	3	508	55.6	8.38		0.318
High	Q99755	' PIP5K1A	Phosphatidylinositol 4-phosphate 5-kinase type-1 alpha OS=Homo s	0	7.142	8.718861	3	3	3	562	62.6	8.21		0.259
High	P57772	' EEFSEC	Selenocysteine-specific elongation factor OS=Homo sapiens OX=960	0	7.142	5.369128	2	3	2	596	65.3	8.35		0.141
High	Q7Z2K6	' ERMP1	Endoplasmic reticulum metallopeptidase 1 OS=Homo sapiens OX=9	0	7.061	3.761062	3	5	3	904	100.2	7.52		0.184
High	Q9Y2R4	' DDX52	Probable ATP-dependent RNA helicase DDX52 OS=Homo sapiens OX	0	7.045	4.507513	2	3	2	599	67.5	9.67		0.145
High	P25815	' S100P	Protein S100-P OS=Homo sapiens OX=9606 GN=S100P PE=1 SV=2	0	7.027	24.21053	2	5	2	95	10.4	4.88		2.162
High	Q96S44	' TP53RK	EKC/KEOPS complex subunit TP53RK OS=Homo sapiens OX=9606 GN	0	7.016	6.719368	2	4	2	253	28.1	9.54		0.389
High	P49841-2	' GSK3B	Isoform 2 of Glycogen synthase kinase-3 beta OS=Homo sapiens OX	0	7.012	12.70208	3	6	3	433	48	8.78		0.318
High	P60468	' SEC61B	Protein transport protein Sec61 subunit beta OS=Homo sapiens OX=	0	6.989	21.875	2	8	2	96	10	11.56		1.154
High	Q15843	' NEDD8	NEDD8 OS=Homo sapiens OX=9606 GN=NEDD8 PE=1 SV=1	0	6.98	17.28395	1	4	1	81	9.1	8.43		0.931
High	Q9NX02	' NLRP2	NACHT, LRR and PYD domains-containing protein 2 OS=Homo sapie	0	6.964	3.483992	3	7	3	1062	120.4	6.07		0.107
High	O15173-2	' PGRMC2	Isoform 2 of Membrane-associated progesterone receptor compone	0	6.962	12.55061	3	4	2	247	26.2	5.31		0.638
High	Q92905	' COPS5	COP9 signalosome complex subunit 5 OS=Homo sapiens OX=9606 G	0	6.946	5.389222	2	2	2	334	37.6	6.54		0.259
High	O75962	' TRIO	Triple functional domain protein OS=Homo sapiens OX=9606 GN=TF	0	6.943	1.162415	3	4	3	3097	346.7	6.37		0.039
High	P01703	' IGLV1-40	Immunoglobulin lambda variable 1-40 OS=Homo sapiens OX=9606 (	0	6.932	38.98305	2	4	2	118	12.3	5.74		4.623
High	P29992	' GNA11	Guanine nucleotide-binding protein subunit alpha-11 OS=Homo sap	0	6.929	9.470752	3	5	1	359	42.1	5.69		0.334
High	P21926	' CD9	CD9 antigen OS=Homo sapiens OX=9606 GN=CD9 PE=1 SV=4	0	6.919	7.45614	2	3	2	228	25.4	7.15		0.778
High	P08631	' HCK	Tyrosine-protein kinase HCK OS=Homo sapiens OX=9606 GN=HCK P	0	6.905	4.942966	3	9	1	526	59.6	6.7		0.241
High	Q96KP1	' EXOC2	Exocyst complex component 2 OS=Homo sapiens OX=9606 GN=EXO	0	6.9	3.896104	3	3	3	924	104	6.9		0.136
High	Q6P597-3	' KLC3	Isoform 3 of Kinesin light chain 3 OS=Homo sapiens OX=9606 GN=KI	0	6.895	7.528958	2	3	2	518	56.8	6.58		0.16
High	P28074	' PSMB5	Proteasome subunit beta type-5 OS=Homo sapiens OX=9606 GN=PS	0	6.891	10.26616	2	4	2	263	28.5	6.92		0.311
High	Q8N4V1-2	' MMGT1	Isoform 2 of Membrane magnesium transporter 1 OS=Homo sapien	0	6.891	12.2449	1	10	1	196	21.9	9.54		0.668
High	Q9UQR1	' ZNF148	Zinc finger protein 148 OS=Homo sapiens OX=9606 GN=ZNF148 PE=	0	6.874	2.770781	2	3	2	794	88.9	6.48		0.116
High	Q5J8M3	' EMC4	ER membrane protein complex subunit 4 OS=Homo sapiens OX=960	0	6.851	15.84699	2	4	2	183	20.1	8.62		0.585
High	P20936	' RASA1	Ras GTPase-activating protein 1 OS=Homo sapiens OX=9606 GN=RA	0	6.85	3.91595	3	5	3	1047	116.3	6.54		0.131
High	Q9UKF6	' CPSF3	Cleavage and polyadenylation specificity factor subunit 3 OS=Homo	0	6.844	3.947368	2	6	2	684	77.4	5.6		0.129
High	O43150	' ASAP2	Arf-GAP with SH3 domain, ANK repeat and PH domain-containing pr	0	6.834	3.677932	3	13	3	1006	111.6	6.68		0.124
High	O43633	' CHMP2A	Charged multivesicular body protein 2a OS=Homo sapiens OX=9606	0	6.823	12.16216	2	4	2	222	25.1	5.97		0.468
High	P09488	' GSTM1	Glutathione S-transferase Mu 1 OS=Homo sapiens OX=9606 GN=GS	0	6.814	7.798165	2	4	2	218	25.7	6.7		0.292
High	Q9Y676	' MRPS18B	28S ribosomal protein S18b, mitochondrial OS=Homo sapiens OX=9	0	6.796	16.27907	3	4	3	258	29.4	9.38		0.585
High	Q13625-3	' TP53BP2	Isoform 3 of Apoptosis-stimulating of p53 protein 2 OS=Homo sapie	0	6.787	3.35097	3	5	3	1134	126.2	6.21		0.129
High	P61088	' UBE2N	Ubiquitin-conjugating enzyme E2 N OS=Homo sapiens OX=9606 GN=	0	6.768	11.84211	2	7	2	152	17.1	6.57		0.52
High	Q5H9R7-5	' PPP6R3	Isoform 5 of Serine/threonine-protein phosphatase 6 regulatory sub	0	6.758	2.730375	2	4	2	879	98.4	4.64		0.096
High	Q8IVG5	' SAMD9L	Sterile alpha motif domain-containing protein 9-like OS=Homo sapie	0	6.745	2.146465	3	4	2	1584	184.4	8.02		0.075
High	Q92922	' SMARCC1	SWI/SNF complex subunit SMARCC1 OS=Homo sapiens OX=9606 GN	0	6.712	2.986425	3	9	1	1105	122.8	5.76		0.118
High	O15438-4	' ABCC3	Isoform 4 of Canalicular multispecific organic anion transporter 2 OS	0	6.65	1.837769	2	2	2	1578	174.8	7.64		0.059
High	P01583	' IL1A	Interleukin-1 alpha OS=Homo sapiens OX=9606 GN=IL1A PE=1 SV=1	0	6.647	10.3321	2	6	2	271	30.6	5.2		0.359
High	Q9Y2Q9	' MRPS28	28S ribosomal protein S28, mitochondrial OS=Homo sapiens OX=96	0	6.633	16.04278	3	5	3	187	20.8	9.1		0.585
High	Q13404-1	' UBE2V1	Isoform 1 of Ubiquitin-conjugating enzyme E2 variant 1 OS=Homo s	0	6.629	13.12217	3	5	3	221	25.8	8.32		0.585
High	Q08257	' CRYZ	Quinone oxidoreductase OS=Homo sapiens OX=9606 GN=CRYZ PE=1	0	6.617	5.775076	2	7	2	329	35.2	8.44		0.245
High	P01599	' IGKV1-17	Immunoglobulin kappa variable 1-17 OS=Homo sapiens OX=9606 GI	0	6.603	23.07692	2	3	2	117	12.8	8.68		0.931
High	Q9HAV4	' XPO5	Exportin-5 OS=Homo sapiens OX=9606 GN=XPO5 PE=1 SV=1	0	6.581	1.993355	2	2	2	1204	136.2	5.8		0.069
High	P31350-2	' RRM2	Isoform 2 of Ribonucleoside-diphosphate reductase subunit M2 OS=	0	6.58	7.7951	3	3	3	449	51.1	6.57		0.25
High	Q9NSI6	' BRWD1	Bromodomain and WD repeat-containing protein 1 OS=Homo sapie	0	6.572	0.991379	2	6	1	2320	262.8	8.46		0.033
High	P19447	' ERCC3	General transcription and DNA repair factor IIH helicase subunit XPE	0	6.57	4.859335	3	3	3	782	89.2	7.23		0.151
High	Q9P2Q2	' FRMD4A	FERM domain-containing protein 4A OS=Homo sapiens OX=9606 GN	0	6.557	2.598653	2	2	2	1039	115.4	8.87		0.076
High	P17600	' SYN1	Synapsin-1 OS=Homo sapiens OX=9606 GN=SYN1 PE=1 SV=3	0	6.546	2.269504	1	2	1	705	74.1	9.83		0.066
High	Q9H1E3	' NUCKS1	Nuclear ubiquitous casein and cyclin-dependent kinase substrate 1 (	0	6.539	11.93416	2	4	2	243	27.3	5.08		0.468
High	Q9NZC4-3	' EHF	Isoform 3 of ETS homologous factor OS=Homo sapiens OX=9606 GN	0	6.535	9.627329	2	3	2	322	37.3	7.53		0.389
High	Q5T750	' XP32	Skin-specific protein 32 OS=Homo sapiens OX=9606 GN=XP32 PE=1	0	6.534	8	2	13	2	250	26.2	7.97		0.425
High	Q86UU0	' BCL9L	B-cell CLL/lymphoma 9-like protein OS=Homo sapiens OX=9606 GN=	0	6.496	3.135424	3	6	3	1499	157	8.63		0.145
High	O75525	' KHDRBS3	KH domain-containing, RNA-binding, signal transduction-associated	0	6.483	8.381503	3	11	1	346	38.8	7.61		0.438
High	O96008	' TOMM40	Mitochondrial import receptor subunit TOM40 homolog OS=Homo	0	6.477	9.418283	3	3	3	361	37.9	7.25		0.54
High	Q96RQ3	' MCCC1	Methylcrotonoyl-CoA carboxylase subunit alpha, mitochondrial OS=	0	6.477	4.275862	2	2	2	725	80.4	7.78		0.108
High	Q15797	' SMAD1	Mothers against decapentaplegic homolog 1 OS=Homo sapiens OX=	0	6.476	5.376344	2	6	1	465	52.2	7.31		0.222
High	Q15382	' RHEB	GTP-binding protein Rheb OS=Homo sapiens OX=9606 GN=RHEB PE	0	6.476	13.58696	3	4	3	184	20.5	5.92		0.778
High	Q15800	' MSMO1	Methylsterol monooxygenase 1 OS=Homo sapiens OX=9606 GN=MS	0	6.468	4.778157	1	6	1	293	35.2	7.23		0.259
High	P57729	' RAB38	Ras-related protein Rab-38 OS=Homo sapiens OX=9606 GN=RAB38	0	6.464	13.27014	2	4	2	211	23.7	7.77		0.334
High	Q9NXE4-4	' SMPD4	Isoform 4 of Sphingomyelin phosphodiesterase 4 OS=Homo sapiens	0	6.429	2.960526	3	5	3	912	102.5	8.53		0.136
High	P05549	' TFAP2A	Transcription factor AP-2-alpha OS=Homo sapiens OX=9606 GN=TFA	0	6.39	6.17849	3	5	3	437	48	8.02		0.438
High	Q6ZNB6	' NFXL1	NF-X1-type zinc finger protein NFXL1 OS=Homo sapiens OX=9606 GI	0	6.385	4.829857	2	3	2	911	101.3	8.41		0.084
High	Q9BRX9	' WDR83	WD repeat domain-containing protein 83 OS=Homo sapiens OX=960	0	6.379	13.65079	3	4	3	315	34.3	5.58		0.501
High	O00483	' NDUFA4	Cytochrome c oxidase subunit NDUFA4 OS=Homo sapiens OX=9606	0	6.377	22.22222	2	7	2	81	9.4	9.38		1.154
High	P05156	' CFI	Complement factor I OS=Homo sapiens OX=9606 GN=CFI PE=1 SV=2	0	6.37	3.602058	2	7	2	583	65.7	7.5		0.141
High	Q9P2P6	' STARD9	StAR-related lipid transfer protein 9 OS=Homo sapiens OX=9606 GN	0	6.367	0.87234	3	6	1	4700	516	6.32	Phospho	0.027
High	P55210-3	' CASP7	Isoform Alpha' of Caspase-7 OS=Homo sapiens OX=9606 GN=CASP7	0	6.357	9.821429	2	4	2	336	37.8	6.37		0.245
High	P37198	' NUP62	Nuclear pore glycoprotein p62 OS=Homo sapiens OX=9606 GN=NUF	0	6.348	5.363985	2	5	2	522	53.2	5.31		0.425
High	O43592	' XPOT	Exportin-T OS=Homo sapiens OX=9606 GN=XPOT PE=1 SV=2	0	6.341	2.286902	2	6	2	962	109.9	5.39		0.103
High	O43823	' AKAP8	A-kinase anchor protein 8 OS=Homo sapiens OX=9606 GN=AKAP8 P	0	6.331	6.069364	3	5	3	692	76.1	5.15		0.25
High	P27338	' MAOB	Amine oxidase [flavin-containing] B OS=Homo sapiens OX=9606 GN=	0	6.329	4.423077	2	3	2	520	58.7	7.5		0.155
High	Q9P0J0-2	' NDUFA13	Isoform 2 of NADH dehydrogenase [ubiquinone] 1 alpha subcomple	0	6.321	16.21622	3	6	3	222	24.9	9.7		0.701
High	P62328	' TMSB4X	Thymosin beta-4 OS=Homo sapiens OX=9606 GN=TMSB4X PE=1 SV=	0	6.317	45.45455	3	11	3	44	5.1	5.06		2.162
High	Q9P003	' CNIH4	Protein cornichon homolog 4 OS=Homo sapiens OX=9606 GN=CNIH	0	6.316	14.38849	1	6	1	139	16.1	6.65		0.778
High	Q9NXH8	' TOR4A	Torsin-4A OS=Homo sapiens OX=9606 GN=TOR4A PE=1 SV=2	0	6.312	7.801418	3	3	3	423	46.9	9.94		0.28
High	Q7Z7H5	' TMED4	Transmembrane emp24 domain-containing protein 4 OS=Homo sap	0	6.304	7.929515	2	4	2	227	25.9	8.28		0.359
High	Q9Y4K3	' TRAF6	TNF receptor-associated factor 6 OS=Homo sapiens OX=9606 GN=TI	0	6.302	6.51341	2	2	2	522	59.5	6.44		0.166
High	P25686	' DNAJB2	DnaJ homolog subfamily B member 2 OS=Homo sapiens OX=9606 G	0	6.286	6.790123	2	6	1	324	35.6	5.95		0.292
High	O15226-2	' NKRF	Isoform 2 of NF-kappa-B-repressing factor OS=Homo sapiens OX=96	0	6.282	4.397163	3	5	3	705	79.4	8.88		0.099
High	P28347	' TEAD1	Transcriptional enhancer factor TEF-1 OS=Homo sapiens OX=9606 G	0	6.267	4.225352	2	3	2	426	47.9	8.15		0.311
High	Q96PU4	' UHRF2	E3 ubiquitin-protein ligase UHRF2 OS=Homo sapiens OX=9606 GN=L	0	6.261	2.369077	2	5	1	802	89.9	8.21		0.103
High	P47974	' ZFP36L2	mRNA decay activator protein ZFP36L2 OS=Homo sapiens OX=9606	0	6.259	6.680162	2	4	1	494	51	8.16		0.245
High	Q14894	' CRYM	Ketimine reductase mu-crystallin OS=Homo sapiens OX=9606 GN=C	0	6.257	5.732484	2	6	2	314	33.8	5.14		0.359
High	Q9UBU8	' MORF4L1	Mortality factor 4-like protein 1 OS=Homo sapiens OX=9606 GN=M(	0	6.257	7.458564	2	3	2	362	41.4	9.28		0.233
High	Q13670	' PMS2P11	Putative postmeiotic segregation increased 2-like protein 11 OS=Ho	0	6.252	5.185185	1	3	1	270	28.5	6.7		0.194
High	Q16540	' MRPL23	39S ribosomal protein L23, mitochondrial OS=Homo sapiens OX=96	0	6.235	15.03268	2	5	2	153	17.8	9.69		0.585
High	Q9BQE5	' APOL2	Apolipoprotein L2 OS=Homo sapiens OX=9606 GN=APOL2 PE=1 SV=	0	6.223	14.24332	3	7	3	337	37.1	6.74		0.468
High	Q14563	' SEMA3A	Semaphorin-3A OS=Homo sapiens OX=9606 GN=SEMA3A PE=1 SV=1	0	6.222	3.891051	2	3	2	771	88.8	7.42		0.089
High	Q8WUA2	' PPIL4	Peptidyl-prolyl cis-trans isomerase-like 4 OS=Homo sapiens OX=960	0	6.193	6.300813	2	7	2	492	57.2	5.92		0.172
High	O76054	' SEC14L2	SEC14-like protein 2 OS=Homo sapiens OX=9606 GN=SEC14L2 PE=1	0	6.159	7.692308	2	4	2	403	46.1	7.84		0.245
High	P11279	' LAMP1	Lysosome-associated membrane glycoprotein 1 OS=Homo sapiens (	0	6.157	5.995204	2	4	2	417	44.9	8.75		0.222
High	P50148	' GNAQ	Guanine nucleotide-binding protein G(q) subunit alpha OS=Homo sa	0	6.155	10.58496	3	4	1	359	42.1	5.68		0.334
High	Q9ULR0-1	' ISY1	Isoform 2 of Pre-mRNA-splicing factor ISY1 homolog OS=Homo sapi	0	6.152	9.667674	2	4	2	331	37.5	5.78		0.202
High	Q9H8H2	' DDX31	Probable ATP-dependent RNA helicase DDX31 OS=Homo sapiens OX	0	6.145	2.350176	2	2	2	851	94	9.99		0.093
High	Q15058	' KIF14	Kinesin-like protein KIF14 OS=Homo sapiens OX=9606 GN=KIF14 PE=	0	6.138	1.63835	3	6	3	1648	186.4	7.91		0.04
High	Q8N7H5	' PAF1	RNA polymerase II-associated factor 1 homolog OS=Homo sapiens C	0	6.138	5.084746	2	2	2	531	59.9	4.63		0.133
High	Q14956	' GPNMB	Transmembrane glycoprotein NMB OS=Homo sapiens OX=9606 GN=	0	6.136	4.72028	2	4	2	572	63.9	6.64		0.222
High	Q9UIS9-1	' MBD1	Isoform 12 of Methyl-CpG-binding domain protein 1 OS=Homo sapi	0	6.128	3.206107	2	4	2	655	72.1	8.78		0.145
High	Q92743	' HTRA1	Serine protease HTRA1 OS=Homo sapiens OX=9606 GN=HTRA1 PE=1	0	6.126	6.875	3	3	3	480	51.3	7.83		0.35
High	P49721	' PSMB2	Proteasome subunit beta type-2 OS=Homo sapiens OX=9606 GN=PS	0	6.115	12.43781	2	2	2	201	22.8	7.02		0.425
High	Q06481	' APLP2	Amyloid-like protein 2 OS=Homo sapiens OX=9606 GN=APLP2 PE=1	0	6.076	4.849279	2	3	2	763	86.9	4.79		0.105
High	P61244	' MAX	Protein max OS=Homo sapiens OX=9606 GN=MAX PE=1 SV=1	0	6.076	11.875	2	5	2	160	18.3	6.3		0.52
High	Q15404	' RSU1	Ras suppressor protein 1 OS=Homo sapiens OX=9606 GN=RSU1 PE=	0	6.055	8.66426	3	8	3	277	31.5	8.65		0.501
High	Q8IVH8	' MAP4K3	Mitogen-activated protein kinase kinase kinase kinase 3 OS=Homo s	0	6.028	3.691275	2	4	2	894	101.3	7.56		0.089
High	Q13459	' MYO9B	Unconventional myosin-IXb OS=Homo sapiens OX=9606 GN=MYO9E	0	6.025	1.576263	3	4	3	2157	243.2	8.75		0.05
High	O00170	' AIP	AH receptor-interacting protein OS=Homo sapiens OX=9606 GN=AIF	0	6.024	8.787879	2	3	2	330	37.6	6.29		0.222
High	Q9NRP0-2	' OSTC	Isoform 2 of Oligosaccharyltransferase complex subunit OSTC OS=H	0	6.019	7.017544	1	7	1	171	19.2	9.19		0.334
High	Q9NPF5	' DMAP1	DNA methyltransferase 1-associated protein 1 OS=Homo sapiens OX	0	6.008	6.638116	2	6	2	467	53	9.5		0.155
High	Q99615	' DNAJC7	DnaJ homolog subfamily C member 7 OS=Homo sapiens OX=9606 G	0	6.005	4.048583	2	2	2	494	56.4	6.96		0.133
High	Q9NX04	' C1orf109	Uncharacterized protein C1orf109 OS=Homo sapiens OX=9606 GN=	0	6.002	15.27094	2	3	2	203	23.4	5.67		0.52
High	O95777	' LSM8	U6 snRNA-associated Sm-like protein LSm8 OS=Homo sapiens OX=9	0	5.999	26.04167	2	3	2	96	10.4	4.48		0.931
High	Q9UBQ5	' EIF3K	Eukaryotic translation initiation factor 3 subunit K OS=Homo sapien	0	5.991	11.46789	2	6	2	218	25	4.93		0.468
High	O43920	' NDUFS5	NADH dehydrogenase [ubiquinone] iron-sulfur protein 5 OS=Homo	0	5.988	17.92453	2	5	2	106	12.5	9.14		0.931
High	P35030	' PRSS3	Trypsin-3 OS=Homo sapiens OX=9606 GN=PRSS3 PE=1 SV=2	0	5.973	4.276316	1	6	1	304	32.5	7.49		0.136
High	O60502	' MGEA5	Protein O-GlcNAcase OS=Homo sapiens OX=9606 GN=MGEA5 PE=1	0	5.969	2.074236	2	4	2	916	102.8	4.91		0.089
High	P17612	' PRKACA	cAMP-dependent protein kinase catalytic subunit alpha OS=Homo s	0	5.963	12.25071	3	4	1	351	40.6	8.79		0.389
High	Q86X95	' CIR1	Corepressor interacting with RBPJ 1 OS=Homo sapiens OX=9606 GN	0	5.959	11.33333	3	7	3	450	52.3	9.86		0.369
High	Q63HN8-4	' RNF213	Isoform 2 of E3 ubiquitin-protein ligase RNF213 OS=Homo sapiens (	0	5.947	0.64688	3	4	3	5256	596.1	6.43		0.022
High	P40763	' STAT3	Signal transducer and activator of transcription 3 OS=Homo sapiens	0	5.939	5.194805	2	6	2	770	88	6.3		0.103
High	P30048	' PRDX3	Thioredoxin-dependent peroxide reductase, mitochondrial OS=Hom	0	5.939	7.421875	2	5	2	256	27.7	7.78		0.389
High	Q9UQ10	' DHDH	Trans-1,2-dihydrobenzene-1,2-diol dehydrogenase OS=Homo sapier	0	5.926	6.886228	2	7	2	334	36.4	7.2		0.259
High	O75251	' NDUFS7	NADH dehydrogenase [ubiquinone] iron-sulfur protein 7, mitochonc	0	5.923	10.79812	2	5	2	213	23.5	9.99		0.389
High	Q15750	' TAB1	TGF-beta-activated kinase 1 and MAP3K7-binding protein 1 OS=Hon	0	5.912	3.769841	2	4	2	504	54.6	5.52		0.202
High	Q9NW82	' WDR70	WD repeat-containing protein 70 OS=Homo sapiens OX=9606 GN=W	0	5.906	3.82263	2	4	2	654	73.2	6.33		0.129
High	P41223-2	' BUD31	Isoform 2 of Protein BUD31 homolog OS=Homo sapiens OX=9606 GI	0	5.901	13.10345	2	2	2	145	17.3	8.87		0.668
High	Q5JPH6-2	' EARS2	Isoform 2 of Probable glutamate--tRNA ligase, mitochondrial OS=Ho	0	5.894	4.681648	2	4	2	534	60	8.07		0.141
High	Q16740	' CLPP	ATP-dependent Clp protease proteolytic subunit, mitochondrial OS=	0	5.885	5.415162	1	1	1	277	30.2	8.09		0.155
High	Q16718	' NDUFA5	NADH dehydrogenase [ubiquinone] 1 alpha subcomplex subunit 5 C	0	5.869	16.37931	2	3	2	116	13.5	5.99		0.931
High	Q9H7N4	' SCAF1	Splicing factor, arginine/serine-rich 19 OS=Homo sapiens OX=9606 (	0	5.847	1.295732	2	3	1	1312	139.2	9.25		0.084
High	Q14919-2	' DRAP1	Isoform 2 of Dr1-associated corepressor OS=Homo sapiens OX=960	0	5.846	10.42654	2	4	2	211	23.1	5.27		0.585
High	P28066	' PSMA5	Proteasome subunit alpha type-5 OS=Homo sapiens OX=9606 GN=P	0	5.844	7.883817	1	7	1	241	26.4	4.79		0.233
High	P50336	' PPOX	Protoporphyrinogen oxidase OS=Homo sapiens OX=9606 GN=PPOX	0	5.827	8.385744	2	4	2	477	50.7	8.16		0.166
High	Q15517	' CDSN	Corneodesmosin OS=Homo sapiens OX=9606 GN=CDSN PE=1 SV=3	0	5.808	5.10397	2	7	2	529	51.5	8.35		0.389
High	Q9BT92	' TCHP	Trichoplein keratin filament-binding protein OS=Homo sapiens OX=9	0	5.801	4.216867	2	3	2	498	61	6.54		0.15
High	P0DOX2	' NA	Immunoglobulin alpha-2 heavy chain OS=Homo sapiens OX=9606 PI	0	5.795	4.615385	2	3	1	455	48.9	6.67		0.202
High	O60256	' PRPSAP2	Phosphoribosyl pyrophosphate synthase-associated protein 2 OS=H	0	5.793	7.588076	2	3	1	369	40.9	7.44		0.194
High	Q99627	' COPS8	COP9 signalosome complex subunit 8 OS=Homo sapiens OX=9606 G	0	5.79	13.8756	2	4	2	209	23.2	5.38		0.585
High	Q92783	' STAM	Signal transducing adapter molecule 1 OS=Homo sapiens OX=9606 (	0	5.771	3.703704	2	2	2	540	59.1	4.82		0.233
High	Q9HDC9	' APMAP	Adipocyte plasma membrane-associated protein OS=Homo sapiens	0	5.757	13.22115	3	4	3	416	46.5	6.16		0.334
High	Q9ULX9	' MAFF	Transcription factor MafF OS=Homo sapiens OX=9606 GN=MAFF PE	0	5.743	19.5122	3	3	2	164	17.7	9.8		0.995
High	Q9BXK1	' KLF16	Krueppel-like factor 16 OS=Homo sapiens OX=9606 GN=KLF16 PE=1	0	5.741	17.06349	2	2	2	252	25.4	9.88		0.425
High	P55042	' RRAD	GTP-binding protein RAD OS=Homo sapiens OX=9606 GN=RRAD PE=	0	5.738	7.792208	2	3	2	308	33.2	8.88		0.359
High	Q92466	' DDB2	DNA damage-binding protein 2 OS=Homo sapiens OX=9606 GN=DDI	0	5.728	6.323185	2	5	2	427	47.8	9.47		0.179
High	P13798	' APEH	Acylamino-acid-releasing enzyme OS=Homo sapiens OX=9606 GN=A	0	5.705	3.278689	2	2	2	732	81.2	5.48		0.136
High	P41240	' CSK	Tyrosine-protein kinase CSK OS=Homo sapiens OX=9606 GN=CSK PE	0	5.682	5.555556	2	5	2	450	50.7	7.06		0.172
High	Q8WUH1	' CHURC1	Isoform 2 of Protein Churchill OS=Homo sapiens OX=9606 GN=CHUF	0	5.679	15	2	3	2	140	16.2	5.19		0.585
High	Q15125	' EBP	3-beta-hydroxysteroid-Delta(8),Delta(7)-isomerase OS=Homo sapier	0	5.679	5.217391	1	3	1	230	26.3	7.9		0.778
High	O75891-3	' ALDH1L1	Isoform 3 of Cytosolic 10-formyltetrahydrofolate dehydrogenase OS	0	5.669	2.083333	2	8	2	912	99.7	5.94		0.038
High	P62304	' SNRPE	Small nuclear ribonucleoprotein E OS=Homo sapiens OX=9606 GN=S	0	5.666	25	2	7	2	92	10.8	9.44		2.162
High	Q5VZM2	' RRAGB	Ras-related GTP-binding protein B OS=Homo sapiens OX=9606 GN=I	0	5.656	5.614973	2	2	2	374	43.2	6.21		0.233
High	Q9Y324	' FCF1	rRNA-processing protein FCF1 homolog OS=Homo sapiens OX=9606	0	5.654	10.10101	2	2	2	198	23.4	9.7		0.668
High	Q99549-2	' MPHOSPH8	Isoform 2 of M-phase phosphoprotein 8 OS=Homo sapiens OX=9606	0	5.64	4.844291	2	3	2	867	97.6	6.06		0.113
High	O95639	' CPSF4	Cleavage and polyadenylation specificity factor subunit 4 OS=Homo	0	5.639	12.26766	2	4	2	269	30.2	8.31		0.274
High	P47989	' XDH	Xanthine dehydrogenase/oxidase OS=Homo sapiens OX=9606 GN=X	0	5.634	1.650413	2	4	2	1333	146.3	7.66		0.066
High	Q96QD8	' SLC38A2	Sodium-coupled neutral amino acid transporter 2 OS=Homo sapiens	0	5.63	4.150198	1	2	1	506	56	8		0.166
High	P61803	' DAD1	Dolichyl-diphosphooligosaccharide--protein glycosyltransferase sub	0	5.622	19.46903	2	2	2	113	12.5	7.08		1.512
High	O95147	' DUSP14	Dual specificity protein phosphatase 14 OS=Homo sapiens OX=9606	0	5.616	18.18182	2	6	2	198	22.2	9.57		0.359
High	Q6P3X3	' TTC27	Tetratricopeptide repeat protein 27 OS=Homo sapiens OX=9606 GN	0	5.608	2.846975	2	3	2	843	96.6	5.59		0.101
High	P61927	' RPL37	60S ribosomal protein L37 OS=Homo sapiens OX=9606 GN=RPL37 P	0	5.599	23.71134	3	13	3	97	11.1	11.74		1.371
High	P28340	' POLD1	DNA polymerase delta catalytic subunit OS=Homo sapiens OX=9606	0	5.597	3.252033	3	3	2	1107	123.6	7.03		0.096
High	Q6ZTI6	' RFLNA	Refilin-A OS=Homo sapiens OX=9606 GN=RFLNA PE=1 SV=3	0	5.594	19.44444	3	6	3	216	23.6	8.48		0.778
High	P09467	' FBP1	Fructose-1,6-bisphosphatase 1 OS=Homo sapiens OX=9606 GN=FBP	0	5.584	6.804734	2	2	2	338	36.8	6.99		0.245
High	Q9H501	' ESF1	ESF1 homolog OS=Homo sapiens OX=9606 GN=ESF1 PE=1 SV=1	0	5.575	3.525264	2	2	2	851	98.7	5.11		0.103
High	P20585	' MSH3	DNA mismatch repair protein Msh3 OS=Homo sapiens OX=9606 GN	0	5.571	2.198769	2	6	2	1137	127.3	8.02		0.068
High	Q5JRX3-2	' PITRM1	Isoform 2 of Presequence protease, mitochondrial OS=Homo sapien	0	5.57	2.697495	2	2	2	1038	117.4	6.92		0.073
High	O75420	' GIGYF1	GRB10-interacting GYF protein 1 OS=Homo sapiens OX=9606 GN=GI	0	5.57	2.801932	2	4	2	1035	114.5	5.39		0.087
High	Q13227	' GPS2	G protein pathway suppressor 2 OS=Homo sapiens OX=9606 GN=GF	0	5.57	4.892966	2	2	2	327	36.7	9.52		0.389
High	A6NCE7	' MAP1LC3B2	Microtubule-associated proteins 1A/1B light chain 3 beta 2 OS=Hom	0	5.568	16.8	2	4	2	125	14.6	8.68		1.154
High	P0DMM9	' SULT1A3	Isoform 3 of Sulfotransferase 1A3 OS=Homo sapiens OX=9606 GN=S	0	5.564	8.9701	2	2	2	301	34.8	5.83		0.245
High	O75427	' LRCH4	Leucine-rich repeat and calponin homology domain-containing prot	0	5.546	4.831625	2	2	2	683	73.4	8.22		0.136
High	P0CG08	' GPR89B	Golgi pH regulator B OS=Homo sapiens OX=9606 GN=GPR89B PE=1	0	5.538	8.351648	2	2	2	455	52.9	9.28		0.222
High	Q12972	' PPP1R8	Nuclear inhibitor of protein phosphatase 1 OS=Homo sapiens OX=9	0	5.537	10.82621	2	2	2	351	38.5	7.37		0.334
High	O95274	' LYPD3	Ly6/PLAUR domain-containing protein 3 OS=Homo sapiens OX=960	0	5.533	10.98266	3	5	3	346	35.9	7.75		0.389
High	Q0ZGT2	' NEXN	Nexilin OS=Homo sapiens OX=9606 GN=NEXN PE=1 SV=1	0	5.526	4.740741	3	3	3	675	80.6	5.33		0.184
High	O00410-3	' IPO5	Isoform 3 of Importin-5 OS=Homo sapiens OX=9606 GN=IPO5	0	5.524	2.421525	2	2	2	1115	125.5	4.92		0.078
High	Q9H019	' MTFR1L	Mitochondrial fission regulator 1-like OS=Homo sapiens OX=9606 G	0	5.518	7.534247	2	5	2	292	31.9	6.1		0.179
High	Q5SY16	' NOL9	Polynucleotide 5'-hydroxyl-kinase NOL9 OS=Homo sapiens OX=9606	0	5.511	4.273504	2	5	2	702	79.3	9.13		0.179
High	Q9HCM4	' EPB41L5	Band 4.1-like protein 5 OS=Homo sapiens OX=9606 GN=EPB41L5 PE	0	5.508	3.001364	2	3	1	733	81.8	6.58		0.129
High	O60508	' CDC40	Pre-mRNA-processing factor 17 OS=Homo sapiens OX=9606 GN=CD	0	5.495	6.217617	2	2	2	579	65.5	7.06		0.125
High	Q9GZS1	' POLR1E	DNA-directed RNA polymerase I subunit RPA49 OS=Homo sapiens O	0	5.494	4.573805	2	3	2	481	53.9	8.56		0.202
High	A0A087W	' IGKV2-40	Immunoglobulin kappa variable 2-40 OS=Homo sapiens OX=9606 GI	0	5.482	10.7438	1	3	1	121	13.3	4.61		0.585
High	Q9NTX5	' ECHDC1	Ethylmalonyl-CoA decarboxylase OS=Homo sapiens OX=9606 GN=E(	0	5.477	7.166124	2	3	2	307	33.7	8.21		0.274
High	Q9BTD8	' RBM42	RNA-binding protein 42 OS=Homo sapiens OX=9606 GN=RBM42 PE=	0	5.472	3.541667	1	2	1	480	50.4	9.63		0.136
High	P17029	' ZKSCAN1	Zinc finger protein with KRAB and SCAN domains 1 OS=Homo sapier	0	5.471	4.795737	2	4	2	563	63.6	7.05		0.166
High	Q969M3-	' YIPF5	Isoform 3 of Protein YIPF5 OS=Homo sapiens OX=9606 GN=YIPF5	0	5.457	7.924528	2	7	2	265	29.1	4.46		1.512
High	O43292	' GPAA1	Glycosylphosphatidylinositol anchor attachment 1 protein OS=Hom	0	5.452	3.703704	2	3	2	621	67.6	8.06		0.194
High	O43402	' EMC8	ER membrane protein complex subunit 8 OS=Homo sapiens OX=960	0	5.449	11.90476	2	3	2	210	23.8	6.4		0.425
High	Q15758	' SLC1A5	Neutral amino acid transporter B(0) OS=Homo sapiens OX=9606 GN	0	5.441	2.218115	1	3	1	541	56.6	5.48		0.11
High	Q92925	' SMARCD2	SWI/SNF-related matrix-associated actin-dependent regulator of ch	0	5.44	6.214689	2	5	2	531	58.9	9.64		0.155
High	Q96G23	' CERS2	Ceramide synthase 2 OS=Homo sapiens OX=9606 GN=CERS2 PE=1 S	0	5.437	4.210526	1	5	1	380	44.8	8.98		0.145
High	Q7Z4F1	' LRP10	Low-density lipoprotein receptor-related protein 10 OS=Homo sapie	0	5.431	4.628331	2	3	2	713	76.1	5.52		0.166
High	Q9BTE6-3	' AARSD1	Isoform 3 of Alanyl-tRNA editing protein Aarsd1 OS=Homo sapiens (	0	5.428	8.191126	3	4	3	586	65.7	6.51		0.218
High	P23193	' TCEA1	Transcription elongation factor A protein 1 OS=Homo sapiens OX=9	0	5.427	9.966777	3	3	3	301	33.9	8.38		0.438
High	Q9H3D4	' TP63	Tumor protein 63 OS=Homo sapiens OX=9606 GN=TP63 PE=1 SV=1	0	5.412	4.558824	2	4	2	680	76.7	6.57		0.136
High	P55854-2	' SUMO3	Isoform 2 of Small ubiquitin-related modifier 3 OS=Homo sapiens O:	0	5.41	8.510638	1	5	1	141	15.8	6.92		0.389
High	P08174-7	' CD55	Isoform 7 of Complement decay-accelerating factor OS=Homo sapie	0	5.408	9.255898	3	5	3	551	59	8.78		0.225
High	Q1ED39	' KNOP1	Lysine-rich nucleolar protein 1 OS=Homo sapiens OX=9606 GN=KNC	0	5.406	5.676856	2	3	2	458	51.6	9.86		0.186
High	P48506	' GCLC	Glutamate--cysteine ligase catalytic subunit OS=Homo sapiens OX=9	0	5.398	2.825746	2	2	2	637	72.7	6.09		0.133
High	Q9H2U1	' DHX36	ATP-dependent RNA helicase DHX36 OS=Homo sapiens OX=9606 GI	0	5.39	1.984127	2	4	2	1008	114.7	7.68		0.081
High	Q9H7B4	' SMYD3	Histone-lysine N-methyltransferase SMYD3 OS=Homo sapiens OX=9	0	5.386	5.373832	2	6	2	428	49.1	7.25		0.222
High	O60216	' RAD21	Double-strand-break repair protein rad21 homolog OS=Homo sapie	0	5.379	3.486529	2	4	2	631	71.6	4.65		0.179
High	P36954	' POLR2I	DNA-directed RNA polymerase II subunit RPB9 OS=Homo sapiens O	0	5.371	18.4	1	1	1	125	14.5	5.14		0.292
High	P21283	' ATP6V1C1	V-type proton ATPase subunit C 1 OS=Homo sapiens OX=9606 GN=A	0	5.37	6.020942	2	6	2	382	43.9	7.46		0.172
High	Q15024	' EXOSC7	Exosome complex component RRP42 OS=Homo sapiens OX=9606 G	0	5.369	9.965636	2	2	2	291	31.8	5.19		0.259
High	Q9BSJ2-4	' TUBGCP2	Isoform 3 of Gamma-tubulin complex component 2 OS=Homo sapie	0	5.368	4.086022	2	2	2	930	105.6	7.08		0.08
High	O60551	' NMT2	Glycylpeptide N-tetradecanoyltransferase 2 OS=Homo sapiens OX=9	0	5.36	2.811245	1	4	1	498	56.9	7.58		0.075
High	Q9NV56	' MRGBP	MRG/MORF4L-binding protein OS=Homo sapiens OX=9606 GN=MR(	0	5.358	13.72549	2	5	2	204	22.4	5.83		0.585
High	P11274	' BCR	Breakpoint cluster region protein OS=Homo sapiens OX=9606 GN=B	0	5.356	1.494886	2	3	1	1271	142.7	7.03		0.072
High	P52435	' POLR2J	DNA-directed RNA polymerase II subunit RPB11-a OS=Homo sapien	0	5.349	12.82051	1	3	1	117	13.3	5.86		0.468
High	P35080	' PFN2	Profilin-2 OS=Homo sapiens OX=9606 GN=PFN2 PE=1 SV=3	0	5.345	20.71429	2	3	2	140	15	6.99		0.778
High	Q96H55	' MYO19	Unconventional myosin-XIX OS=Homo sapiens OX=9606 GN=MYO19	0	5.345	3.195876	2	3	2	970	109.1	7.71		0.096
High	P52630	' STAT2	Signal transducer and activator of transcription 2 OS=Homo sapiens	0	5.331	5.405405	3	3	3	851	97.9	5.49		0.158
High	O95347	' SMC2	Structural maintenance of chromosomes protein 2 OS=Homo sapier	0	5.328	1.670844	2	3	2	1197	135.6	8.43		0.061
High	Q9NRX2	' MRPL17	39S ribosomal protein L17, mitochondrial OS=Homo sapiens OX=96	0	5.326	9.714286	2	2	2	175	20	10.11		0.389
High	P30260-2	' CDC27	Isoform 2 of Cell division cycle protein 27 homolog OS=Homo sapier	0	5.317	2.409639	2	2	2	830	92.6	7.12		0.108
High	P55039	' DRG2	Developmentally-regulated GTP-binding protein 2 OS=Homo sapien	0	5.307	4.67033	2	4	2	364	40.7	8.88		0.202
High	P09417	' QDPR	Dihydropteridine reductase OS=Homo sapiens OX=9606 GN=QDPR I	0	5.296	6.557377	1	4	1	244	25.8	7.37		0.145
High	Q9UNF0	' PACSIN2	Protein kinase C and casein kinase substrate in neurons protein 2 O	0	5.288	4.526749	2	4	2	486	55.7	5.2		0.194
High	P30043	' BLVRB	Flavin reductase (NADPH) OS=Homo sapiens OX=9606 GN=BLVRB PI	0	5.286	7.281553	1	2	1	206	22.1	7.65		0.233
High	O60828	' PQBP1	Polyglutamine-binding protein 1 OS=Homo sapiens OX=9606 GN=P(	0	5.285	9.433962	1	3	1	265	30.5	6.33		0.585
High	Q86VP1	' TAX1BP1	Tax1-binding protein 1 OS=Homo sapiens OX=9606 GN=TAX1BP1 PE	0	5.284	3.168568	2	2	2	789	90.8	5.43		0.045
High	P36507	' MAP2K2	Dual specificity mitogen-activated protein kinase kinase 2 OS=Homo	0	5.27	5	2	3	1	400	44.4	6.55		0.245
High	Q8N6R0	' METTL13	Methyltransferase-like protein 13 OS=Homo sapiens OX=9606 GN=I	0	5.266	4.005722	2	2	2	699	78.7	6.73		0.11
High	Q99470	' SDF2	Stromal cell-derived factor 2 OS=Homo sapiens OX=9606 GN=SDF2 I	0	5.249	17.53555	2	4	2	211	23	7.33		0.52
High	Q96P11-4	' NSUN5	Isoform 4 of Probable 28S rRNA (cytosine-C(5))-methyltransferase C	0	5.249	7.021277	2	2	2	470	51	8.65		0.172
High	O75925-2	' PIAS1	Isoform 2 of E3 SUMO-protein ligase PIAS1 OS=Homo sapiens OX=9	0	5.245	2.29709	1	1	1	653	72.2	7.4		0.07
High	Q14119	' VEZF1	Vascular endothelial zinc finger 1 OS=Homo sapiens OX=9606 GN=V	0	5.24	9.788868	2	2	2	521	56.9	9.52		0.212
High	Q9BYE4	' SPRR2G	Small proline-rich protein 2G OS=Homo sapiens OX=9606 GN=SPRR2	0	5.233	30.13699	2	8	2	73	8.2	7.96		1.512
High	O95785	' WIZ	Protein Wiz OS=Homo sapiens OX=9606 GN=WIZ PE=1 SV=2	0	5.193	1.574803	2	2	2	1651	178.6	6.86		0.053
High	Q8N122	' RPTOR	Regulatory-associated protein of mTOR OS=Homo sapiens OX=9606	0	5.175	2.097378	2	2	2	1335	148.9	6.89		0.066
High	Q9Y3C6	' PPIL1	Peptidyl-prolyl cis-trans isomerase-like 1 OS=Homo sapiens OX=960	0	5.162	10.24096	1	4	1	166	18.2	7.99		0.259
High	Q13098-7	' GPS1	Isoform 2 of COP9 signalosome complex subunit 1 OS=Homo sapien	0	5.156	3.41556	2	3	2	527	59	6.32		0.179
High	P51159	' RAB27A	Ras-related protein Rab-27A OS=Homo sapiens OX=9606 GN=RAB27	0	5.14	8.597285	2	3	1	221	24.9	5.22		0.389
High	Q8WV92	' MITD1	MIT domain-containing protein 1 OS=Homo sapiens OX=9606 GN=M	0	5.139	11.64659	2	2	2	249	29.3	8		0.389
High	Q9NV92	' NDFIP2	NEDD4 family-interacting protein 2 OS=Homo sapiens OX=9606 GN=	0	5.138	4.166667	1	1	1	336	36.4	5.08		0.166
High	Q96B26	' EXOSC8	Exosome complex component RRP43 OS=Homo sapiens OX=9606 G	0	5.135	13.04348	2	2	2	276	30	5.3		0.311
High	P41208	' CETN2	Centrin-2 OS=Homo sapiens OX=9606 GN=CETN2 PE=1 SV=1	0	5.133	21.51163	3	3	3	172	19.7	5		0.995
High	O95168	' NDUFB4	NADH dehydrogenase [ubiquinone] 1 beta subcomplex subunit 4 OS	0	5.125	17.82946	2	6	2	129	15.2	9.85		0.778
High	P20930	' FLG	Filaggrin OS=Homo sapiens OX=9606 GN=FLG PE=1 SV=3	0	5.122	3.792169	2	6	2	4061	434.9	9.25		0.018
High	Q99442	' SEC62	Translocation protein SEC62 OS=Homo sapiens OX=9606 GN=SEC62	0	5.111	4.260652	2	4	2	399	45.8	7.12		0.274
High	Q15070	' OXA1L	Mitochondrial inner membrane protein OXA1L OS=Homo sapiens O.	0	5.111	4.137931	2	4	2	435	48.5	9.45		0.311
High	Q6ZRS2	' SRCAP	Helicase SRCAP OS=Homo sapiens OX=9606 GN=SRCAP PE=1 SV=3	0	5.11	1.052632	3	3	3	3230	343.3	5.96		0.064
High	O00327	' ARNTL	Aryl hydrocarbon receptor nuclear translocator-like protein 1 OS=H	0	5.11	2.875399	2	3	2	626	68.7	6.86		0.15
High	Q13867	' BLMH	Bleomycin hydrolase OS=Homo sapiens OX=9606 GN=BLMH PE=1 S	0	5.1	6.813187	2	5	2	455	52.5	6.27		0.179
High	P57678	' GEMIN4	Gem-associated protein 4 OS=Homo sapiens OX=9606 GN=GEMIN4	0	5.094	2.457467	2	2	2	1058	120	6.04		0.077
High	Q96CT7	' CCDC124	Coiled-coil domain-containing protein 124 OS=Homo sapiens OX=96	0	5.088	5.829596	1	1	1	223	25.8	9.54		0.166
High	O15235	' MRPS12	28S ribosomal protein S12, mitochondrial OS=Homo sapiens OX=96	0	5.083	12.31884	2	2	2	138	15.2	10.29		0.778
High	Q8WTT2	' NOC3L	Nucleolar complex protein 3 homolog OS=Homo sapiens OX=9606 (	0	5.072	3.25	2	3	2	800	92.5	9.17		0.113
High	O14684	' PTGES	Prostaglandin E synthase OS=Homo sapiens OX=9606 GN=PTGES PE	0	5.069	11.84211	2	10	2	152	17.1	9.5		0.778
High	Q02833	' RASSF7	Ras association domain-containing protein 7 OS=Homo sapiens OX=	0	5.067	7.506702	2	2	2	373	39.9	5.62		0.245
High	O60825	' PFKFB2	6-phosphofructo-2-kinase/fructose-2,6-bisphosphatase 2 OS=Homo	0	5.06	3.564356	2	4	2	505	58.4	8.38		0.125
High	Q13405	' MRPL49	39S ribosomal protein L49, mitochondrial OS=Homo sapiens OX=96	0	5.051	8.433735	1	3	1	166	19.2	9.45		0.166
High	O75190	' DNAJB6	DnaJ homolog subfamily B member 6 OS=Homo sapiens OX=9606 G	0	5.046	5.214724	2	6	1	326	36.1	9.16		0.389
High	O15258	' RER1	Protein RER1 OS=Homo sapiens OX=9606 GN=RER1 PE=1 SV=1	0	5.042	9.183673	1	5	1	196	22.9	9.54		0.259
High	Q75N03	' CBLL1	E3 ubiquitin-protein ligase Hakai OS=Homo sapiens OX=9606 GN=CE	0	5.04	7.535642	3	3	3	491	54.5	8.29		0.334
High	P30049	' ATP5F1D	ATP synthase subunit delta, mitochondrial OS=Homo sapiens OX=96	0	5.035	8.333333	1	4	1	168	17.5	5.49		0.334
High	Q96JJ7	' TMX3	Protein disulfide-isomerase TMX3 OS=Homo sapiens OX=9606 GN=T	0	5.032	5.947137	3	4	3	454	51.8	4.91		0.304
High	Q8WUM9	' SLC20A1	Sodium-dependent phosphate transporter 1 OS=Homo sapiens OX=	0	5.026	2.650957	2	3	2	679	73.7	7.08		0.166
High	P16870	' CPE	Carboxypeptidase E OS=Homo sapiens OX=9606 GN=CPE PE=1 SV=1	0	5.026	5.042017	2	2	2	476	53.1	5.14		0.16
High	P05067	' APP	Amyloid-beta A4 protein OS=Homo sapiens OX=9606 GN=APP PE=1	0	5.021	3.246753	2	3	2	770	86.9	4.82		0.113
High	P25490	' YY1	Transcriptional repressor protein YY1 OS=Homo sapiens OX=9606 G	0	5.018	8.454106	3	5	3	414	44.7	6.25		0.438
High	Q8N9Q2	' SREK1IP1	Protein SREK1IP1 OS=Homo sapiens OX=9606 GN=SREK1IP1 PE=1 S\	0	5.015	27.74194	2	5	2	155	18.2	9.85		0.931
High	P56556	' NDUFA6	NADH dehydrogenase [ubiquinone] 1 alpha subcomplex subunit 6 C	0	5.009	14.84375	2	8	2	128	15.1	9.98		0.668
High	O75506	' HSBP1	Heat shock factor-binding protein 1 OS=Homo sapiens OX=9606 GN	0	5.001	57.89474	2	2	2	76	8.5	4.36		0.931
High	O14602	' EIF1AY	Eukaryotic translation initiation factor 1A, Y-chromosomal OS=Hom	0	5	24.30556	3	5	3	144	16.4	5.24		0.995
High	Q9H2U2-3	' PPA2	Isoform 2 of Inorganic pyrophosphatase 2, mitochondrial OS=Homo	0	4.995	6.017192	2	2	2	349	39.6	6.92		0.194
High	Q9BZE1	' MRPL37	39S ribosomal protein L37, mitochondrial OS=Homo sapiens OX=96	0	4.992	5.673759	2	3	2	423	48.1	8.59		0.155
High	Q969G3	' SMARCE1	SWI/SNF-related matrix-associated actin-dependent regulator of ch	0	4.989	4.136253	2	5	1	411	46.6	4.88		0.202
High	Q9H4H8	' FAM83D	Protein FAM83D OS=Homo sapiens OX=9606 GN=FAM83D PE=1 SV=	0	4.968	3.589744	2	2	2	585	64.4	6.54		0.15
High	Q9Y3D6	' FIS1	Mitochondrial fission 1 protein OS=Homo sapiens OX=9606 GN=FIS1	0	4.964	15.78947	2	4	2	152	16.9	8.79		0.778
High	O15516	' CLOCK	Circadian locomoter output cycles protein kaput OS=Homo sapiens	0	4.959	2.364066	2	3	2	846	95.2	7.01		0.136
High	Q9NPD3	' EXOSC4	Exosome complex component RRP41 OS=Homo sapiens OX=9606 G	0	4.958	11.02041	2	3	2	245	26.4	6.52		0.425
High	Q9NP66	' HMG20A	High mobility group protein 20A OS=Homo sapiens OX=9606 GN=HI	0	4.957	4.899135	1	1	1	347	40.1	6.49		0.136
High	Q9H7E9-2	' C8orf33	Isoform 2 of UPF0488 protein C8orf33 OS=Homo sapiens OX=9606 (	0	4.951	11.15702	2	3	2	242	26.4	10.13		0.389
High	P00505	' GOT2	Aspartate aminotransferase, mitochondrial OS=Homo sapiens OX=9	0	4.936	7.44186	3	3	3	430	47.5	9.01		0.269
High	P30086	' PEBP1	Phosphatidylethanolamine-binding protein 1 OS=Homo sapiens OX=	0	4.936	7.486631	1	3	1	187	21	7.53		0.212
High	Q86U86	' PBRM1	Protein polybromo-1 OS=Homo sapiens OX=9606 GN=PBRM1 PE=1	0	4.934	1.598579	2	3	2	1689	192.8	6.89		0.047
High	Q9UNZ5	' C19orf53	Leydig cell tumor 10 kDa protein homolog OS=Homo sapiens OX=96	0	4.917	16.16162	2	3	2	99	10.6	11.55		0.778
High	P58335	' ANTXR2	Anthrax toxin receptor 2 OS=Homo sapiens OX=9606 GN=ANTXR2 P	0	4.914	2.862986	1	1	1	489	53.6	7.46		0.086
High	Q99519	' NEU1	Sialidase-1 OS=Homo sapiens OX=9606 GN=NEU1 PE=1 SV=1	0	4.911	5.301205	2	3	2	415	45.4	5.88		0.222
High	Q9BUW7	' C9orf16	UPF0184 protein C9orf16 OS=Homo sapiens OX=9606 GN=C9orf16 I	0	4.91	30.12048	2	5	2	83	9	4.22		3.642
High	Q96PM9	' ZNF385A	Zinc finger protein 385A OS=Homo sapiens OX=9606 GN=ZNF385A F	0	4.908	5.440415	2	3	2	386	40.4	9.86		0.233
High	Q9HD26	' GOPC	Golgi-associated PDZ and coiled-coil motif-containing protein OS=H	0	4.901	5.627706	2	2	2	462	50.5	5.92		0.172
High	P49736	' MCM2	DNA replication licensing factor MCM2 OS=Homo sapiens OX=9606	0	4.898	2.765487	2	2	2	904	101.8	5.52		0.093
High	Q9P2B4	' CTTNBP2NL	CTTNBP2 N-terminal-like protein OS=Homo sapiens OX=9606 GN=C	0	4.889	4.85133	2	3	2	639	70.1	8.06		0.129
High	Q9Y5Q9	' GTF3C3	General transcription factor 3C polypeptide 3 OS=Homo sapiens OX	0	4.888	3.047404	2	3	2	886	101.2	5.07		0.103
High	Q9UKL0	' RCOR1	REST corepressor 1 OS=Homo sapiens OX=9606 GN=RCOR1 PE=1 SV	0	4.879	5.773196	2	4	2	485	53.3	7.03		0.16
High	Q9Y448	' KNSTRN	Small kinetochore-associated protein OS=Homo sapiens OX=9606 G	0	4.876	4.43038	1	4	1	316	35.4	6.3		0.101
High	Q96BN8	' OTULIN	Ubiquitin thioesterase otulin OS=Homo sapiens OX=9606 GN=OTUL	0	4.876	4.829545	2	2	2	352	40.2	5.47		0.212

High	Q5JTZ9	' AARS2	Alanine--tRNA ligase, mitochondrial OS=Homo sapiens OX=9606 GN	0	4.864	3.147208	2	3	2	985	107.3	6.27		0.083		3350513.91				0				2				2			Q5JTZ9	NA	NA	' AARS2
High	P26447	' S100A4	Protein S100-A4 OS=Homo sapiens OX=9606 GN=S100A4 PE=1 SV=1	0	4.862	19.80198	2	5	2	101	11.7	6.11		1.154			2647893.06	14792313.3			2.24	2.79			1	2			1	2	P26447	NA	2.481931	' S100A4
High	C9JRZ8-2	' AKR1B15	Isoform 2 of Aldo-keto reductase family 1 member B15 OS=Homo sa	0	4.847	4.651163	2	7	1	344	39.4	6.47		0.259	2464645.69				4.32	2.24	2.16	2.21	2	1	1	1	2	1	1	1	C9JRZ8-2	NA	NA	' AKR1B15
High	Q9NSU2-1	' TREX1	Isoform 1 of Three-prime repair exonuclease 1 OS=Homo sapiens O	0	4.824	5.691057	2	2	2	369	38.9	8.41		0.292		950345.938				2.25				1				1			Q9NSU2-1	NA	NA	' TREX1
High	Q8TBK6	' ZCCHC10	Zinc finger CCHC domain-containing protein 10 OS=Homo sapiens O	0	4.823	7.291667	1	3	1	192	21	8.53		0.468	11239153	13069094			2.97	2.86			1	1			1	1			Q8TBK6	0.217626	NA	' ZCCHC10
High	P51617	' IRAK1	Interleukin-1 receptor-associated kinase 1 OS=Homo sapiens OX=96	0	4.821	3.230337	2	4	2	712	76.5	6.62		0.16			1729851.56	1673758.31			0	2.06			1	1			1	1	P51617	NA	-0.04756	' IRAK1
High	O00767	' SCD	Acyl-CoA desaturase OS=Homo sapiens OX=9606 GN=SCD PE=1 SV=	0	4.818	3.62117	1	3	1	359	41.5	9		0.129		26020513		8224068.63		2.98		2.16		1		1		1		1	O00767	NA	NA	' SCD
High	P46100	' ATRX	Transcriptional regulator ATRX OS=Homo sapiens OX=9606 GN=ATR	0	4.798	0.76244	2	2	2	2492	282.4	6.58		0.035																	P46100	NA	NA	' ATRX
High	P09429	' HMGB1	High mobility group protein B1 OS=Homo sapiens OX=9606 GN=HM	0	4.795	13.95349	2	5	2	215	24.9	5.74		0.52		8374436.39				5.75				2				2			P09429	NA	NA	' HMGB1
High	Q8NE86	' MCU	Calcium uniporter protein, mitochondrial OS=Homo sapiens OX=960	0	4.794	7.407407	2	4	2	351	39.8	8.65		0.274		4286635.25			2.13	1.67			1	1			1	1			Q8NE86	NA	NA	' MCU
High	P56385	' ATP5ME	ATP synthase subunit e, mitochondrial OS=Homo sapiens OX=9606	0	4.794	24.63768	2	2	2	69	7.9	9.35		1.512	4917222.47		8816483.03		2.21		2.39		1		1		1		1		P56385	NA	NA	' ATP5ME
High	Q04760	' GLO1	Lactoylglutathione lyase OS=Homo sapiens OX=9606 GN=GLO1 PE=	0	4.792	13.04348	2	3	2	184	20.8	5.31		0.468						2.31				1				1			Q04760	NA	NA	' GLO1
High	O14730	' RIOK3	Serine/threonine-protein kinase RIO3 OS=Homo sapiens OX=9606 G	0	4.777	3.853565	2	3	2	519	59.1	5.76		0.083		4324023.75				1.95				1				1			O14730	NA	NA	' RIOK3
High	Q99426	' TBCB	Tubulin-folding cofactor B OS=Homo sapiens OX=9606 GN=TBCB PE	0	4.77	8.196721	2	2	2	244	27.3	5.15		0.334			1883128.44				0				1				1		Q99426	NA	NA	' TBCB
High	Q9BZG1	' RAB34	Ras-related protein Rab-34 OS=Homo sapiens OX=9606 GN=RAB34	0	4.763	7.722008	2	2	2	259	29	7.88		0.259																	Q9BZG1	NA	NA	' RAB34
High	Q9BZI1	' IRX2	Iroquois-class homeodomain protein IRX-2 OS=Homo sapiens OX=9	0	4.761	3.609342	1	1	1	471	49.1	5.29		0.11			10417739.3				3.09				1				1		Q9BZI1	NA	NA	' IRX2
High	P59665	' DEFA1	Neutrophil defensin 1 OS=Homo sapiens OX=9606 GN=DEFA1 PE=1	0	4.76	19.14894	2	6	2	94	10.2	6.99		1.154	9569602			7553202.44	1.89		1.83	1.6	1		1	1	1		1	1	P59665	NA	NA	' DEFA1
High	Q9Y5K8	' ATP6V1D	V-type proton ATPase subunit D OS=Homo sapiens OX=9606 GN=AT	0	4.741	4.453441	1	2	1	247	28.2	9.36		0.179				1849912.5				2.11				1				1	Q9Y5K8	NA	NA	' ATP6V1D
High	Q9UHQ4-	' BCAP29	Isoform 2 of B-cell receptor-associated protein 29 OS=Homo sapien	0	4.731	6.896552	2	4	2	348	40.8	9.72		0.222				2520272.45				2.18				2				2	Q9UHQ4-	NA	NA	' BCAP29
High	Q9NUQ2	' AGPAT5	1-acyl-sn-glycerol-3-phosphate acyltransferase epsilon OS=Homo sa	0	4.714	9.065934	2	4	2	364	42	9.1		0.233	4727655.22	4217649.28			2.22	2.76			2	1			2	1			Q9NUQ2	-0.16469	NA	' AGPAT5
High	Q9BVT8	' TMUB1	Transmembrane and ubiquitin-like domain-containing protein 1 OS=	0	4.713	12.60163	2	3	2	246	26.2	5.72		2.162					0				1				1				Q9BVT8	NA	NA	' TMUB1
High	P01701	' IGLV1-51	Immunoglobulin lambda variable 1-51 OS=Homo sapiens OX=9606	0	4.71	13.67521	2	6	2	117	12.2	7.03		1.512																	P01701	NA	NA	' IGLV1-51
High	Q00536-2	' CDK16	Isoform 2 of Cyclin-dependent kinase 16 OS=Homo sapiens OX=960	0	4.704	2.807018	2	6	1	570	63.4	8.13		0.133			1141347.22		2.88		2.12		1		2		1		2		Q00536-2	NA	NA	' CDK16
High	P51809-2	' VAMP7	Isoform 2 of Vesicle-associated membrane protein 7 OS=Homo sapi	0	4.701	8.076923	2	2	2	260	30.2	8.37		0.274				5089667.31				1.65				1				1	P51809-2	NA	NA	' VAMP7
High	O43293	' DAPK3	Death-associated protein kinase 3 OS=Homo sapiens OX=9606 GN=	0	4.697	2.863436	1	1	1	454	52.5	6.89		0.101																	O43293	NA	NA	' DAPK3
High	Q14004	' CDK13	Cyclin-dependent kinase 13 OS=Homo sapiens OX=9606 GN=CDK13	0	4.696	1.455026	2	6	1	1512	164.8	9.69		0.06					2.88		2.12		1		1		1		1		Q14004	NA	NA	' CDK13
High	Q6PJ61	' FBXO46	F-box only protein 46 OS=Homo sapiens OX=9606 GN=FBXO46 PE=1	0	4.682	6.799337	2	3	2	603	64.6	7.4		0.166	3745933.5		748149.125		0		0		1		1		1		1		Q6PJ61	NA	NA	' FBXO46
High	Q99805	' TM9SF2	Transmembrane 9 superfamily member 2 OS=Homo sapiens OX=96	0	4.676	2.111614	1	3	1	663	75.7	7.44		0.089		6953609				3.26				1				1			Q99805	NA	NA	' TM9SF2
High	Q96CU9	' FOXRED1	FAD-dependent oxidoreductase domain-containing protein 1 OS=H	0	4.663	4.73251	2	2	2	486	53.8	7.78		0.194			1864927.81			2.07	0			1	1			1	1		Q96CU9	NA	NA	' FOXRED1
High	P22694-2	' PRKACB	Isoform 2 of cAMP-dependent protein kinase catalytic subunit beta	0	4.662	10.80402	3	3	1	398	46.2	8.69		0.35	3120600.63				4.39				3				3				P22694-2	NA	NA	' PRKACB
High	O00479	' HMGN4	High mobility group nucleosome-binding domain-containing protein	0	4.661	17.77778	2	9	1	90	9.5	10.48		2.162	41254572.8	38453515.4	1140379.05		4.53	3.92	0		2	2	1		2	2	1		O00479	-0.10144	NA	' HMGN4
High	P56937	' HSD17B7	3-keto-steroid reductase OS=Homo sapiens OX=9606 GN=HSD17B7	0	4.638	3.519062	1	1	1	341	38.2	8.1		0.129																	P56937	NA	NA	' HSD17B7
High	Q03169	' TNFAIP2	Tumor necrosis factor alpha-induced protein 2 OS=Homo sapiens O	0	4.638	3.51682	2	2	2	654	72.6	6.46		0.122				2541189.25				0				1				1	Q03169	NA	NA	' TNFAIP2
High	P04150-3	' NR3C1	Isoform Alpha-2 of Glucocorticoid receptor OS=Homo sapiens OX=9	0	4.631	3.984576	2	3	2	778	85.8	6.49		0.108		3640077.88				0				1				1			P04150-3	NA	NA	' NR3C1
High	Q12894-2	' IFRD2	Isoform 2 of Interferon-related developmental regulator 2 OS=Hom	0	4.622	4.934211	2	5	2	608	65.6	8.59		0.172	5047610.79	2260522.22			1.73	2.07			1	1			1	1			Q12894-2	-1.15894	NA	' IFRD2
High	Q8NHH9	' ATL2	Atlastin-2 OS=Homo sapiens OX=9606 GN=ATL2 PE=1 SV=2	0	4.617	3.259005	1	2	1	583	66.2	5.48		0.083	1744332.56				0				1				1				Q8NHH9	NA	NA	' ATL2
High	Q9NWV4	' C1orf123	UPF0587 protein C1orf123 OS=Homo sapiens OX=9606 GN=C1orf12	0	4.613	15	2	2	2	160	18	5.01		0.585			4972010.38				0				1				1		Q9NWV4	NA	NA	' C1orf123
High	Q9NQS7	' INCENP	Inner centromere protein OS=Homo sapiens OX=9606 GN=INCENP	0	4.595	2.505447	2	6	2	918	105.4	9.44		0.101	3523653.02	535543.316			0	0			2	1			2	1			Q9NQS7	-2.718	NA	' INCENP
High	P62891	' RPL39	60S ribosomal protein L39 OS=Homo sapiens OX=9606 GN=RPL39 P	0	4.592	23.52941	2	8	2	51	6.4	12.56		99	6275793.25	254426833	53094252.8	61555901	0	3.43	2.48	3.02	1	1	1	1	1	1	1	1	P62891	5.341309	0.213341	' RPL39
High	Q9UI09	' NDUFA12	NADH dehydrogenase [ubiquinone] 1 alpha subcomplex subunit 12	0	4.59	20	2	2	2	145	17.1	9.63		0.585	4360409.29				0				1				1				Q9UI09	NA	NA	' NDUFA12
High	P98172	' EFNB1	Ephrin-B1 OS=Homo sapiens OX=9606 GN=EFNB1 PE=1 SV=1	0	4.583	8.959538	2	3	2	346	38	8.94		0.233		2661703.94				2.46				1				1			P98172	NA	NA	' EFNB1
High	Q92567	' FAM168A	Protein FAM168A OS=Homo sapiens OX=9606 GN=FAM168A PE=1 S	0	4.574	7.377049	2	3	2	244	26.2	8.98		0.931			5633236.38	2068532.22			2.59	1.7			1	1			1	1	Q92567	NA	-1.44536	' FAM168A
High	Q14574	' DSC3	Desmocollin-3 OS=Homo sapiens OX=9606 GN=DSC3 PE=1 SV=3	0	4.56	2.455357	2	3	2	896	99.9	6.1		0.083		5323039.38				2.59				1				1			Q14574	NA	NA	' DSC3
High	Q5T5U3	' ARHGAP21	Rho GTPase-activating protein 21 OS=Homo sapiens OX=9606 GN=A	0.001	4.555	1.788452	2	3	2	1957	217.2	7.8		0.037		1835262.56				0				1				1			Q5T5U3	NA	NA	' ARHGAP21
High	P07099	' EPHX1	Epoxide hydrolase 1 OS=Homo sapiens OX=9606 GN=EPHX1 PE=1 S	0.001	4.554	3.956044	1	1	1	455	52.9	7.25		0.093																	P07099	NA	NA	' EPHX1
High	Q9P287-2	' BCCIP	Isoform 2 of BRCA2 and CDKN1A-interacting protein OS=Homo sapi	0.001	4.552	7.453416	2	2	2	322	36.2	4.69		0.259		6310888.81				2.65				1				1			Q9P287-2	NA	NA	' BCCIP
High	Q92785	' DPF2	Zinc finger protein ubi-d4 OS=Homo sapiens OX=9606 GN=DPF2 PE=	0.001	4.55	4.092072	1	1	1	391	44.1	6.33		0.101					2.94				1				1				Q92785	NA	NA	' DPF2
High	Q7Z333-4	' SETX	Isoform 4 of Probable helicase senataxin OS=Homo sapiens OX=960	0.001	4.549	1.330377	2	2	2	2706	306.1	7.24		0.028					2.36				2				2				Q7Z333-4	NA	NA	' SETX
High	P51649-2	' ALDH5A1	Isoform 2 of Succinate-semialdehyde dehydrogenase, mitochondria	0.001	4.547	2.554745	1	4	1	548	58.6	8.09		0.086	20927483.4	12357337.1			2.51	2.22			1	1			1	1			P51649-2	-0.76003	NA	' ALDH5A1
High	Q8N448	' LNX2	Ligand of Numb protein X 2 OS=Homo sapiens OX=9606 GN=LNX2 P	0.001	4.546	5.942029	2	4	2	690	76	6.64		0.16							0	0			1	1			3	1	Q8N448	NA	NA	' LNX2
High	Q9BVL4	' SELENOO	Selenoprotein O OS=Homo sapiens OX=9606 GN=SELENOO PE=1 SV	0.001	4.543	3.736921	2	2	2	669	73.4	5.97		0.129			1081929.16				1.62				1				1		Q9BVL4	NA	NA	' SELENOO
High	Q92609-2	' TBC1D5	Isoform 2 of TBC1 domain family member 5 OS=Homo sapiens OX=	0.001	4.534	3.79437	2	4	2	817	91.2	6.33		0.108						0				1				1			Q92609-2	NA	NA	' TBC1D5
High	Q05397-5	' PTK2	Isoform 5 of Focal adhesion kinase 1 OS=Homo sapiens OX=9606 GN	0.001	4.532	1.877934	2	2	1	1065	120.8	6.74		0.036				671847.781				0				1				1	Q05397-5	NA	NA	' PTK2
High	P22528	' SPRR1B	Cornifin-B OS=Homo sapiens OX=9606 GN=SPRR1B PE=1 SV=2	0.001	4.528	28.08989	2	7	2	89	9.9	8.48		0.778	18509748.6	4782910.88		6551637.96	2.04	0		0	1	1		2	1	1		2	P22528	-1.95232	NA	' SPRR1B
High	Q9BVC6	' TMEM109	Transmembrane protein 109 OS=Homo sapiens OX=9606 GN=TMEM	0.001	4.527	4.938272	1	4	1	243	26.2	10.48		0.233		7585800.5				2.02				1				1			Q9BVC6	NA	NA	' TMEM109
High	P36404	' ARL2	ADP-ribosylation factor-like protein 2 OS=Homo sapiens OX=9606 G	0.001	4.526	13.04348	2	3	2	184	20.9	6.34		0.52				4869358.09				2.4				2				2	P36404	NA	NA	' ARL2
High	P41227	' NAA10	N-alpha-acetyltransferase 10 OS=Homo sapiens OX=9606 GN=NAA1	0.001	4.522	12.76596	3	4	3	235	26.4	5.64		0.54						0				1				1			P41227	NA	NA	' NAA10
High	P01700	' IGLV1-47	Immunoglobulin lambda variable 1-47 OS=Homo sapiens OX=9606	0.001	4.522	24.78632	2	4	2	117	12.3	5.91		2.981																	P01700	NA	NA	' IGLV1-47
High	Q92879-4	' CELF1	Isoform 4 of CUGBP Elav-like family member 1 OS=Homo sapiens O	0.001	4.513	4.296875	2	4	2	512	55	8.38		0.245																	Q92879-4	NA	NA	' CELF1
High	P57105	' SYNJ2BP	Synaptojanin-2-binding protein OS=Homo sapiens OX=9606 GN=SYN	0.001	4.508	8.275862	1	1	1	145	15.9	6.3		0.292																	P57105	NA	NA	' SYNJ2BP
High	Q96D71	' REPS1	RalBP1-associated Eps domain-containing protein 1 OS=Homo sapie	0.001	4.508	1.256281	1	2	1	796	86.6	5.69		0.055				2040728.09				2.1				1				1	Q96D71	NA	NA	' REPS1
High	O95391	' SLU7	Pre-mRNA-splicing factor SLU7 OS=Homo sapiens OX=9606 GN=SLU	0.001	4.499	5.290102	3	3	3	586	68.3	7.14		0.189																	O95391	NA	NA	' SLU7
High	O75717	' WDHD1	WD repeat and HMG-box DNA-binding protein 1 OS=Homo sapiens	0.001	4.498	1.771479	2	2	2	1129	125.9	5.62		0.075	1512908.16				1.86				2				2				O75717	NA	NA	' WDHD1
High	P51610-4	' HCFC1	Isoform 4 of Host cell factor 1 OS=Homo sapiens OX=9606 GN=HCF	0.001	4.484	0.577201	1	3	1	2079	213.3	7.33		0.031		2755267.75			1.9	1.79			1	1			1	1			P51610-4	NA	NA	' HCFC1
High	Q8N4X5-4	' AFAP1L2	Isoform 4 of Actin filament-associated protein 1-like 2 OS=Homo sa	0.001	4.476	4.15677	2	2	2	842	93.8	5.35		0.099	6927507.97				3.11				1				1				Q8N4X5-4	NA	NA	' AFAP1L2
High	Q9UK45	' LSM7	U6 snRNA-associated Sm-like protein LSm7 OS=Homo sapiens OX=9	0.001	4.474	32.03883	2	2	2	103	11.6	5.27		0.931				2030338.34				1.7				1				1	Q9UK45	NA	NA	' LSM7
High	O75935	' DCTN3	Dynactin subunit 3 OS=Homo sapiens OX=9606 GN=DCTN3 PE=1 SV	0.001	4.473	8.064516	2	3	2	186	21.1	5.47		0.179		3642967.06				2.3				1				1			O75935	NA	NA	' DCTN3
High	O43251-8	' RBFOX2	Isoform 8 of RNA binding protein fox-1 homolog 2 OS=Homo sapien	0.001	4.472	5.764967	1	1	1	451	47.3	6.62		0.155					3.74				1				1				O43251-8	NA	NA	' RBFOX2
High	O00217	' NDUFS8	NADH dehydrogenase [ubiquinone] iron-sulfur protein 8, mitochon	0.001	4.463	13.80952	2	9	2	210	23.7	6.34		0.334	2859375	3778018.44		3974220.14	1.88	2.09		0	1	1		2	1	1		2	O00217	0.40193	NA	' NDUFS8
High	Q8N9M1	' C19orf47	Uncharacterized protein C19orf47 OS=Homo sapiens OX=9606 GN=	0.001	4.462	3.317536	1	1	1	422	44.7	10.11		0.086																	Q8N9M1	NA	NA	' C19orf47
High	Q9H9T3	' ELP3	Elongator complex protein 3 OS=Homo sapiens OX=9606 GN=ELP3	0.001	4.456	2.925046	2	5	2	547	62.2	8.88		0.145		1674442.44	1861482.31	3767991.47		0	0	0		1	1	1		1	1	1	Q9H9T3	NA	1.017344	' ELP3
High	Q9H6Y2	' WDR55	WD repeat-containing protein 55 OS=Homo sapiens OX=9606 GN=W	0.001	4.45	6.005222	1	1	1	383	42	4.92		0.11																	Q9H6Y2	NA	NA	' WDR55
High	Q06587	' RING1	E3 ubiquitin-protein ligase RING1 OS=Homo sapiens OX=9606 GN=R	0.001	4.442	3.448276	1	5	1	406	42.4	5.62		0.129			3175133.28	2383980.45			2.46	2.04			1	1			1	1	Q06587	NA	-0.41344	' RING1
High	P25789	' PSMA4	Proteasome subunit alpha type-4 OS=Homo sapiens OX=9606 GN=P	0.001	4.44	6.896552	2	4	2	261	29.5	7.72		0.359			1517501.13	7189633.38			0	2.54			1	1			1	1	P25789	NA	2.244221	' PSMA4
High	Q8NFH5	' NUP35	Nucleoporin NUP35 OS=Homo sapiens OX=9606 GN=NUP35 PE=1 S	0.001	4.429	9.815951	2	2	2	326	34.8	9.09		0.389																	Q8NFH5	NA	NA	' NUP35
High	Q8NC54	' KCT2	Keratinocyte-associated transmembrane protein 2 OS=Homo sapier	0.001	4.428	4.90566	1	1	1	265	29.2	5.08		0.179				10111312.5				2.73				1				1	Q8NC54	NA	NA	' KCT2
High	Q14728	' MFSD10	Major facilitator superfamily domain-containing protein 10 OS=Hon	0.001	4.427	2.857143	1	4	1	455	48.3	9.6		0.11	2225772.69	5627148.63			2.49	3.05			1	1			1	1			Q14728	1.338098	NA	' MFSD10
High	Q7Z5K2-3	' WAPL	Isoform 3 of Wings apart-like protein homolog OS=Homo sapiens O	0.001	4.422	2.352941	2	2	2	1275	142.4	6.06		0.056																	Q7Z5K2-3	NA	NA	' WAPL
High	O00220	' TNFRSF10A	Tumor necrosis factor receptor superfamily member 10A OS=Homo	0.001	4.416	2.777778	1	1	1	468	50.1	7.03		0.105								2.69				1				1	O00220	NA	NA	' TNFRSF10A
High	O75607	' NPM3	Nucleoplasmin-3 OS=Homo sapiens OX=9606 GN=NPM3 PE=1 SV=3	0.001	4.415	8.426966	1	2	1	178	19.3	4.63		0.292	17787597.1				3.29				1				1				O75607	NA	NA	' NPM3
High	Q00587	' CDC42EP1	Cdc42 effector protein 1 OS=Homo sapiens OX=9606 GN=CDC42EP1	0.001	4.414	9.462916	2	2	2	391	40.3	7.15		0.122	2306382				2.62				1				1				Q00587	NA	NA	' CDC42EP1
High	P46937-9	' YAP1	Isoform 9 of Transcriptional coactivator YAP1 OS=Homo sapiens OX	0.001	4.414	4.92126	2	3	2	508	54.9	5.24		0.233				2446115.63				1.74				1				1	P46937-9	NA	NA	' YAP1
High	O60313-1	' OPA1	Isoform 4 of Dynamin-like 120 kDa protein, mitochondrial OS=Homo	0.001	4.411	2.561576	2	2	2	1015	117.7	7.77		0.068				1352252.63				0				1				1	O60313-1	NA	NA	' OPA1
High	O76071	' CIAO1	Probable cytosolic iron-sulfur protein assembly protein CIAO1 OS=H	0.001	4.405	7.374631	2	6	2	339	37.8	4.97		0.292	3175035.09		1793880.59	1884534.19	1.69		0	0	2		1	1	2		1	1	O76071	NA	0.071124	' CIAO1
High	Q9Y3D0	' FAM96B	Mitotic spindle-associated MMXD complex subunit MIP18 OS=Hom	0.001	4.392	13.49693	1	1	1	163	17.7	5.19		0.389		5595297.38				3.01				1				1			Q9Y3D0	NA	NA	' FAM96B
High	Q9BQB6	' VKORC1	Vitamin K epoxide reductase complex subunit 1 OS=Homo sapiens	0.001	4.391	7.97546	1	4	1	163	18.2	9.36		0.585			6731873.5				1.91				1				1		Q9BQB6	NA	NA	' VKORC1
High	Q9NPE3	' NOP10	H/ACA ribonucleoprotein complex subunit 3 OS=Homo sapiens OX=	0.001	4.384	20.3125	1	3	1	64	7.7	9.99		0.778		9031340.81			2.05	2.53			1	1			1	1			Q9NPE3	NA	NA	' NOP10
High	Q9UJK0	' TSR3	Ribosome biogenesis protein TSR3 homolog OS=Homo sapiens OX=	0.001	4.382	3.525641	1	3	1	312	33.6	6.87		0.166		3960782.88				2.26				1				1			Q9UJK0	NA	NA	' TSR3
High	Q9BRT2	' UQCC2	Ubiquinol-cytochrome-c reductase complex assembly factor 2 OS=H	0.001	4.382	10.31746	1	1	1	126	14.9	7.37		0.334			4677832.63				3.13				1				1		Q9BRT2	NA	NA	' UQCC2
High	Q9UNL2-2	' SSR3	Isoform 2 of Translocon-associated protein subunit gamma OS=Hon	0.001	4.373	7.070707	1	1	1	198	22.6	9.63		0.334																	Q9UNL2-2	NA	NA	' SSR3
High	P48651	' PTDSS1	Phosphatidylserine synthase 1 OS=Homo sapiens OX=9606 GN=PTD	0.001	4.336	2.748414	1	2	1	473	55.5	8.43		0.105			5419369.63		1.66		2.7		1		1		1		1		P48651	NA	NA	' PTDSS1
High	Q9H9J2	' MRPL44	39S ribosomal protein L44, mitochondrial OS=Homo sapiens OX=96	0.001	4.333	6.325301	2	2	2	332	37.5	8.4		0.233																	Q9H9J2	NA	NA	' MRPL44
High	Q96KP4	' CNDP2	Cytosolic non-specific dipeptidase OS=Homo sapiens OX=9606 GN=	0.001	4.328	4.421053	2	3	2	475	52.8	5.97		0.172				1060270.52				1.77				1				1	Q96KP4	NA	NA	' CNDP2
High	O60220	' TIMM8A	Mitochondrial import inner membrane translocase subunit Tim8 A	0.001	4.319	11.34021	1	6	1	97	11	5.16		0.389	11474953.8	11250955.6		13483348.6	2.44	2.35	2.78	2.32	1	1	1	1	1	1	1	1	O60220	-0.02844	NA	' TIMM8A
High	P12273	' PIP	Prolactin-inducible protein OS=Homo sapiens OX=9606 GN=PIP PE=	0.001	4.319	7.534247	1	4	1	146	16.6	8.05		0.212	4903789.16			1253683.28	2.37			0	1			1	1			1	P12273	NA	NA	' PIP
High	Q13887	' KLF5	Krueppel-like factor 5 OS=Homo sapiens OX=9606 GN=KLF5 PE=1 S	0.001	4.316	6.564551	1	3	1	457	50.8	8.6		0.292																	Q13887	NA	NA	' KLF5
High	Q5SWX8	' ODR4	Protein odr-4 homolog OS=Homo sapiens OX=9606 GN=ODR4 PE=1	0.001	4.308	5.947137	2	4	2	454	51.1	5.92		0.194		3275625.5	1597196.5	1587708.2		0	0	0		1	1	1		1	1	1	Q5SWX8	NA	-0.0086	' ODR4
High	Q14807	' KIF22	Kinesin-like protein KIF22 OS=Homo sapiens OX=9606 GN=KIF22 PE	0.001	4.307	4.210526	2	2	2	665	73.2	9.45		0.119																	Q14807	NA	NA	' KIF22
High	Q9H4A6	' GOLPH3	Golgi phosphoprotein 3 OS=Homo sapiens OX=9606 GN=GOLPH3 P	0.001	4.3	5.033557	1	1	1	298	33.8	6.44		0.136	5791603				3.08				1				1				Q9H4A6	NA	NA	' GOLPH3
High	Q96IX5	' USMG5	Up-regulated during skeletal muscle growth protein 5 OS=Homo sa	0.001	4.295	17.24138	1	2	1	58	6.5	9.76		1.154		8389669.13				2.22				1				1			Q96IX5	NA	NA	' USMG5
High	Q6PIU2-2	' NCEH1	Isoform 2 of Neutral cholesterol ester hydrolase 1 OS=Homo sapien	0.001	4.282	5.769231	2	2	2	416	46.6	6.62		0.222		884495.5				0				1				1			Q6PIU2-2	NA	NA	' NCEH1
High	Q6UW68	' TMEM205	Transmembrane protein 205 OS=Homo sapiens OX=9606 GN=TMEM	0.001	4.274	8.994709	1	2	1	189	21.2	8.62		0.468				25286341.9				2.98				1				1	Q6UW68	NA	NA	' TMEM205
High	P10644	' PRKAR1A	cAMP-dependent protein kinase type I-alpha regulatory subunit OS	0.001	4.271	6.824147	2	3	2	381	43	5.35		0.259				5401996.63				1.62				1				1	P10644	NA	NA	' PRKAR1A
High	Q16186	' ADRM1	Proteasomal ubiquitin receptor ADRM1 OS=Homo sapiens OX=9606	0.001	4.268	5.405405	2	8	2	407	42.1	5.07		0.468	5716896.69	5134052.03			0	1.72			1	2			1	2			Q16186	-0.15513	NA	' ADRM1
High	O43824	' GTPBP6	Putative GTP-binding protein 6 OS=Homo sapiens OX=9606 GN=GTF	0.001	4.253	3.488372	2	2	2	516	56.9	9.42		0.166					1.96				1				1				O43824	NA	NA	' GTPBP6
High	P12694-2	' BCKDHA	Isoform 2 of 2-oxoisovalerate dehydrogenase subunit alpha, mitoch	0.001	4.248	5.357143	2	2	2	448	50.8	7.15		0.194			1802717.08				0				1				1		P12694-2	NA	NA	' BCKDHA
High	P25311	' AZGP1	Zinc-alpha-2-glycoprotein OS=Homo sapiens OX=9606 GN=AZGP1 P	0.001	4.248	7.38255	2	3	2	298	34.2	6.05		0.274	2449963.13				1.62				1				1				P25311	NA	NA	' AZGP1
High	O15525	' MAFG	Transcription factor MafG OS=Homo sapiens OX=9606 GN=MAFG P	0.001	4.243	11.11111	2	3	1	162	17.8	10.04		0.468																	O15525	NA	NA	' MAFG
High	Q8NI27	' THOC2	THO complex subunit 2 OS=Homo sapiens OX=9606 GN=THOC2 PE	0.001	4.239	1.443817	2	2	2	1593	182.7	8.44		0.053	4740065.75				1.8				1				1				Q8NI27	NA	NA	' THOC2
High	Q9Y3D9	' MRPS23	28S ribosomal protein S23, mitochondrial OS=Homo sapiens OX=96	0.001	4.22	10.52632	2	2	2	190	21.8	8.9		0.425	4681021.75				2.19				1				1				Q9Y3D9	NA	NA	' MRPS23
High	P01308	' INS	Insulin OS=Homo sapiens OX=9606 GN=INS PE=1 SV=1	0.001	4.212	25.45455	2	5	2	110	12	5.3		1.512	59949623	58130127.3			2.38	2.05			2	1			2	1			P01308	-0.04446	NA	' INS
High	Q8N6T3-2	' ARFGAP1	Isoform 2 of ADP-ribosylation factor GTPase-activating protein 1 OS	0.001	4.208	7.004831	2	3	2	414	45.6	5.78		0.212				2914590.38				0				1				1	Q8N6T3-2	NA	NA	' ARFGAP1
High	Q9BT40	' INPP5K	Inositol polyphosphate 5-phosphatase K OS=Homo sapiens OX=960	0.001	4.202	2.232143	1	3	1	448	51.1	6.54		0.11			1245378.94	3277264.48			2.26	2.23			1	1			1	1	Q9BT40	NA	1.395907	' INPP5K
High	A0A075B6	' IGLV8-61	Immunoglobulin lambda variable 8-61 OS=Homo sapiens OX=9606	0.001	4.198	7.377049	1	3	1	122	12.8	4.55		2.162																	A0A075B6	NA	NA	' IGLV8-61
High	P54756	' EPHA5	Ephrin type-A receptor 5 OS=Homo sapiens OX=9606 GN=EPHA5 PE	0.001	4.179	2.314368	2	4	1	1037	114.7	6.93		0.08		7410703				2.92				1				1			P54756	NA	NA	' EPHA5
High	Q9NUY8	' TBC1D23	TBC1 domain family member 23 OS=Homo sapiens OX=9606 GN=TB	0.001	4.168	3.576538	2	2	2	699	78.3	5.41		0.133						0				1				1			Q9NUY8	NA	NA	' TBC1D23
High	P98170	' XIAP	E3 ubiquitin-protein ligase XIAP OS=Homo sapiens OX=9606 GN=XIA	0.001	4.166	5.432596	2	3	2	497	56.6	6.65		0.133		6489122.56				2.2				2				2			P98170	NA	NA	' XIAP
High	P07196	' NEFL	Neurofilament light polypeptide OS=Homo sapiens OX=9606 GN=NE	0.001	4.155	2.946593	2	5	1	543	61.5	4.65		0.119			4403705.63	7846849.88			4.19	4.01			2	2			2	2	P07196	NA	0.833396	' NEFL
High	Q5VYK3	' ECPAS	Proteasome adapter and scaffold protein ECM29 OS=Homo sapiens	0.001	4.151	1.626016	2	2	2	1845	204.2	7.12		0.042		1341568.27				0		2.08		1		1		1		1	Q5VYK3	NA	NA	' ECPAS
High	Q9BTE1	' DCTN5	Dynactin subunit 5 OS=Homo sapiens OX=9606 GN=DCTN5 PE=1 SV	0.001	4.151	6.043956	1	5	1	182	20.1	8.02		0.259		3939307	4968257.06	6912185.75		1.99	2.18	2.14		1	1	1		1	1	1	Q9BTE1	NA	0.476402	' DCTN5
High	Q9H967	' WDR76	WD repeat-containing protein 76 OS=Homo sapiens OX=9606 GN=W	0.001	4.15	4.472843	2	2	2	626	69.7	9.25		0.133																	Q9H967	NA	NA	' WDR76
High	Q9H3K2	' GHITM	Growth hormone-inducible transmembrane protein OS=Homo sapie	0.001	4.149	2.898551	1	3	1	345	37.2	9.94		0.145	7314083.25		3540593.88		2.73		0		1		1		1		1		Q9H3K2	NA	NA	' GHITM
High	Q9UK58	' CCNL1	Cyclin-L1 OS=Homo sapiens OX=9606 GN=CCNL1 PE=1 SV=1	0.001	4.144	6.653992	2	6	2	526	59.6	10.7		0.202	5481206.69				0				2				2				Q9UK58	NA	NA	' CCNL1
High	P63165	' SUMO1	Small ubiquitin-related modifier 1 OS=Homo sapiens OX=9606 GN=	0.001	4.142	20.79208	2	5	2	101	11.6	5.52		0.668		7605811.81				2.34				1				1			P63165	NA	NA	' SUMO1
High	O60869	' EDF1	Endothelial differentiation-related factor 1 OS=Homo sapiens OX=9	0.001	4.131	10.13514	1	1	1	148	16.4	9.95		0.212																	O60869	NA	NA	' EDF1
High	Q8NBS9	' TXNDC5	Thioredoxin domain-containing protein 5 OS=Homo sapiens OX=96	0.001	4.129	4.398148	2	3	2	432	47.6	5.97		0.186			1543090.06				0				1				1		Q8NBS9	NA	NA	' TXNDC5
High	P82932	' MRPS6	28S ribosomal protein S6, mitochondrial OS=Homo sapiens OX=960	0.001	4.128	12.8	2	2	2	125	14.2	9.26		0.668	4669435.5				1.96				1				1				P82932	NA	NA	' MRPS6
High	Q9BZL1	' UBL5	Ubiquitin-like protein 5 OS=Homo sapiens OX=9606 GN=UBL5 PE=1	0.001	4.127	20.54795	2	9	2	73	8.5	8.44		1.512	18083703.3	8517470	10133744.9	9534791.85	2.18	2.08	2.01	2.14	2	1	1	1	2	1	1	1	Q9BZL1	-1.08619	-0.08789	' UBL5
High	P12270	' TPR	Nucleoprotein TPR OS=Homo sapiens OX=9606 GN=TPR PE=1 SV=3	0.002	4.119	1.184934	2	6	2	2363	267.1	5.02		0.033	761669.219	1409419.05	846021.813		0	0	0		1	1	1		1	1	1		P12270	0.887864	NA	' TPR
High	Q13888	' GTF2H2	General transcription factor IIH subunit 2 OS=Homo sapiens OX=96	0.002	4.115	2.278481	1	3	1	395	44.4	6.76		0.093		5160213.88				2.11				1				1			Q13888	NA	NA	' GTF2H2
High	Q9UN81	' L1RE1	LINE-1 retrotransposable element ORF1 protein OS=Homo sapiens	0.002	4.112	3.846154	1	2	1	338	40	9.51		0.116	2609672.25				2.32				1				1				Q9UN81	NA	NA	' L1RE1
High	P62273-2	' RPS29	Isoform 2 of 40S ribosomal protein S29 OS=Homo sapiens OX=9606	0.002	4.104	11.9403	2	7	2	67	8.1	10.02		1.512	92605277.9			11235112.3	2.18	2.16		2.25	1	1		1	1	1		1	P62273-2	NA	NA	' RPS29
High	Q9BTW9-	' TBCD	Isoform 4 of Tubulin-specific chaperone D OS=Homo sapiens OX=96	0.002	4.093	2.24359	2	2	2	1248	138.6	6.37		0.069																	Q9BTW9-	NA	NA	' TBCD
High	Q9UKM7	' MAN1B1	Endoplasmic reticulum mannosyl-oligosaccharide 1,2-alpha-mannos	0.002	4.093	4.005722	2	5	2	699	79.5	7.72		0.122	88444346.5	13183110.1			0	2.01			1	1			1	1			Q9UKM7	-2.74608	NA	' MAN1B1
High	Q8WUK0	' PTPMT1	Phosphatidylglycerophosphatase and protein-tyrosine phosphatase	0.002	4.079	6.467662	1	1	1	201	22.8	9.77		0.179																	Q8WUK0	NA	NA	' PTPMT1
High	Q13596-3	' SNX1	Isoform 3 of Sorting nexin-1 OS=Homo sapiens OX=9606 GN=SNX1	0.002	4.079	1.974865	1	1	1	557	63	5.48		0.072				4616462.66				2.36				1				1	Q13596-3	NA	NA	' SNX1
High	Q12765-2	' SCRN1	Isoform 2 of Secernin-1 OS=Homo sapiens OX=9606 GN=SCRN1	0.002	4.075	4.83871	2	2	2	434	48.7	4.78		0.194				1159235.94				1.8				1				1	Q12765-2	NA	NA	' SCRN1
High	Q9NZQ3	' NCKIPSD	NCK-interacting protein with SH3 domain OS=Homo sapiens OX=96	0.002	4.063	3.601108	1	3	1	722	78.9	6.38		0.068			3794666.31	2056243.56			3.43	3.49			1	1			1	1	Q9NZQ3	NA	-0.88396	' NCKIPSD
High	Q9NZJ7	' MTCH1	Mitochondrial carrier homolog 1 OS=Homo sapiens OX=9606 GN=M	0.002	4.06	2.827763	1	4	1	389	41.5	9.32		0.122		7919430.41				2.73		1.94		1		1		1		1	Q9NZJ7	NA	NA	' MTCH1
High	A0A0A0M	' IGKV3D-11	Immunoglobulin kappa variable 3D-11 OS=Homo sapiens OX=9606	0.002	4.059	7.826087	1	8	1	115	12.6	5.29		1.154		5555219.38	6443932.91			2.24	2.02			1	1			1	1		A0A0A0M	NA	NA	' IGKV3D-11
High	Q9UBN7	' HDAC6	Histone deacetylase 6 OS=Homo sapiens OX=9606 GN=HDAC6 PE=1	0.002	4.055	2.304527	2	3	2	1215	131.3	5.3		0.125		3841869.75	1630124.94			1.81	0			1	1			1	1		Q9UBN7	NA	NA	' HDAC6
High	Q06828	' FMOD	Fibromodulin OS=Homo sapiens OX=9606 GN=FMOD PE=1 SV=2	0.002	4.05	5.319149	2	3	2	376	43.2	6.04		0.311	4264865.25				3.48				2				2				Q06828	NA	NA	' FMOD
High	Q92581-2	' SLC9A6	Isoform 2 of Sodium/hydrogen exchanger 6 OS=Homo sapiens OX=	0.002	4.05	1.71184	1	1	1	701	77.9	6.44		0.096				1846311.72				2.4				1				1	Q92581-2	NA	NA	' SLC9A6
High	Q2M2I8	' AAK1	AP2-associated protein kinase 1 OS=Homo sapiens OX=9606 GN=AA	0.002	4.048	4.162331	3	3	3	961	103.8	6.6		0.158																	Q2M2I8	NA	NA	' AAK1
High	P01116	' KRAS	GTPase KRas OS=Homo sapiens OX=9606 GN=KRAS PE=1 SV=1	0.002	4.043	13.75661	2	4	2	189	21.6	6.77		0.52		1384410.38	2003243.56			2.14	1.89			1	1			1	1		P01116	NA	NA	' KRAS
High	Q709F0	' ACAD11	Acyl-CoA dehydrogenase family member 11 OS=Homo sapiens OX=	0.002	4.04	1.538462	1	1	1	780	87.2	8.12		0.054		2463095.63				2.14				1				1			Q709F0	NA	NA	' ACAD11
High	P25788	' PSMA3	Proteasome subunit alpha type-3 OS=Homo sapiens OX=9606 GN=P	0.002	4.036	4.705882	1	1	1	255	28.4	5.33		0.179																	P25788	NA	NA	' PSMA3
High	Q5BJD5	' TMEM41B	Transmembrane protein 41B OS=Homo sapiens OX=9606 GN=TMEM	0.002	4.025	6.52921	1	1	1	291	32.5	9.58		0.233		2139674.97				2.44				1				1			Q5BJD5	NA	NA	' TMEM41B
High	Q13257	' MAD2L1	Mitotic spindle assembly checkpoint protein MAD2A OS=Homo sapi	0.002	4.022	8.780488	2	2	2	205	23.5	5.08		0.359	6768380.45	4424021.22			0	1.88			1	1			1	1			Q13257	-0.61345	NA	' MAD2L1
High	Q9H0C8	' ILKAP	Integrin-linked kinase-associated serine/threonine phosphatase 2C	0.002	4.003	5.102041	2	4	2	392	42.9	7.09		0.179	3390772.73	6348844.75			4.48	2.96			2	1			2	1			Q9H0C8	0.90488	NA	' ILKAP
High	Q9BV68	' RNF126	E3 ubiquitin-protein ligase RNF126 OS=Homo sapiens OX=9606 GN=	0.002	4.003	9.815951	1	2	1	326	35.6	5.72		0.931	12076495.4	3897888.5			2.58	0			1	1			1	1			Q9BV68	-1.63144	NA	' RNF126
High	Q9UKN8	' GTF3C4	General transcription factor 3C polypeptide 4 OS=Homo sapiens OX	0.002	3.982	3.406326	2	2	2	822	91.9	6.65		0.081					2.13				1				1				Q9UKN8	NA	NA	' GTF3C4
High	Q13322	' GRB10	Growth factor receptor-bound protein 10 OS=Homo sapiens OX=96	0.002	3.975	5.050505	2	2	2	594	67.2	7.87		0.16		2348062.63				0				1				1			Q13322	NA	NA	' GRB10
High	O00194	' RAB27B	Ras-related protein Rab-27B OS=Homo sapiens OX=9606 GN=RAB27	0.002	3.956	11.92661	2	2	2	218	24.6	5.52		0.389								1.68				1				1	O00194	NA	NA	' RAB27B
High	P61962	' DCAF7	DDB1- and CUL4-associated factor 7 OS=Homo sapiens OX=9606 GN	0.002	3.952	6.140351	2	2	2	342	38.9	5.52		0.274	6496712.13				1.88				1				1				P61962	NA	NA	' DCAF7
High	P01593	' IGKV1D-33	Immunoglobulin kappa variable 1D-33 OS=Homo sapiens OX=9606	0.002	3.941	13.67521	1	1	1	117	12.8	4.78		0.585																	P01593	NA	NA	' IGKV1D-33
High	P35573	' AGL	Glycogen debranching enzyme OS=Homo sapiens OX=9606 GN=AGL	0.002	3.937	2.023499	3	3	3	1532	174.7	6.76		0.071					0				1				1				P35573	NA	NA	' AGL
High	Q9Y5J9	' TIMM8B	Mitochondrial import inner membrane translocase subunit Tim8 B	0.002	3.936	13.25301	1	3	1	83	9.3	5.12		0.389			4514429.34	5516427.88			0	2.42			1	1			1	1	Q9Y5J9	NA	0.289191	' TIMM8B
High	P20226	' TBP	TATA-box-binding protein OS=Homo sapiens OX=9606 GN=TBP PE=	0.002	3.933	6.19469	2	3	2	339	37.7	9.79		0.52																	P20226	NA	NA	' TBP
High	Q13033	' STRN3	Striatin-3 OS=Homo sapiens OX=9606 GN=STRN3 PE=1 SV=3	0.002	3.927	2.885822	2	4	2	797	87.2	5.36		0.07			11876142.4				0				1				1		Q13033	NA	NA	' STRN3
High	P37837	' TALDO1	Transaldolase OS=Homo sapiens OX=9606 GN=TALDO1 PE=1 SV=2	0.002	3.927	5.04451	2	2	2	337	37.5	6.81		0.101																	P37837	NA	NA	' TALDO1
High	O60664	' PLIN3	Perilipin-3 OS=Homo sapiens OX=9606 GN=PLIN3 PE=1 SV=3	0.002	3.924	7.603687	2	3	2	434	47	5.44		0.166				5026638.34				2.26				1				1	O60664	NA	NA	' PLIN3
High	P11474	' ESRRA	Steroid hormone receptor ERR1 OS=Homo sapiens OX=9606 GN=ES	0.002	3.919	3.782506	1	2	1	423	45.5	6.38		0.105																	P11474	NA	NA	' ESRRA
High	O14763	' TNFRSF10B	Tumor necrosis factor receptor superfamily member 10B OS=Homo	0.002	3.913	7.272727	2	4	2	440	47.8	5.55		0.222		2530368.38		12451812.3		1.62		2.22		1		2		1		2	O14763	NA	NA	' TNFRSF10B
High	P50897	' PPT1	Palmitoyl-protein thioesterase 1 OS=Homo sapiens OX=9606 GN=PP	0.002	3.902	4.248366	1	1	1	306	34.2	6.52		0.166																	P50897	NA	NA	' PPT1
High	Q8N5I2	' ARRDC1	Arrestin domain-containing protein 1 OS=Homo sapiens OX=9606 G	0.002	3.886	5.773672	2	3	2	433	46	7.02		0.359	50834486.9			2147089.03	2.13			0	1			1	1			1	Q8N5I2	NA	NA	' ARRDC1
High	Q9BRD0	' BUD13	BUD13 homolog OS=Homo sapiens OX=9606 GN=BUD13 PE=1 SV=1	0.002	3.873	3.392593	2	2	2	619	70.5	9.86		0.105		1296818.81				0				1				1			Q9BRD0	NA	NA	' BUD13
High	Q8IYL3	' C1orf174	UPF0688 protein C1orf174 OS=Homo sapiens OX=9606 GN=C1orf17	0.002	3.873	5.761317	1	2	1	243	26	6.9		0.212																	Q8IYL3	NA	NA	' C1orf174
High	Q8IY22	' CMIP	C-Maf-inducing protein OS=Homo sapiens OX=9606 GN=CMIP PE=1	0.002	3.867	2.457956	1	2	1	773	86.3	6.7		0.059				3053925.25				2.3				1				1	Q8IY22	NA	NA	' CMIP
High	Q9NQ48	' LZTFL1	Leucine zipper transcription factor-like protein 1 OS=Homo sapiens	0.002	3.867	7.35786	2	4	2	299	34.6	5.36		0.311		1255843.88				1.75				1				1			Q9NQ48	NA	NA	' LZTFL1
High	Q9BRT6	' LLPH	Protein LLP homolog OS=Homo sapiens OX=9606 GN=LLPH PE=1 SV	0.002	3.846	12.4031	1	2	1	129	15.2	10.37		0.585		11569805.3				3.16				1				1			Q9BRT6	NA	NA	' LLPH
High	Q15398	' DLGAP5	Disks large-associated protein 5 OS=Homo sapiens OX=9606 GN=DL	0.002	3.846	3.073286	2	2	2	846	95.1	9		0.086	3839328.25				0				1				1				Q15398	NA	NA	' DLGAP5
High	P36941	' LTBR	Tumor necrosis factor receptor superfamily member 3 OS=Homo sa	0.002	3.84	10.8046	1	1	1	435	46.7	5.83		0.155				5543973				3.89				1				1	P36941	NA	NA	' LTBR
High	Q9Y2A9	' B3GNT3	N-acetyllactosaminide beta-1,3-N-acetylglucosaminyltransferase 3	0.002	3.839	4.032258	1	1	1	372	42.5	8.68		0.155																	Q9Y2A9	NA	NA	' B3GNT3
High	Q9H2C0	' GAN	Gigaxonin OS=Homo sapiens OX=9606 GN=GAN PE=1 SV=1	0.002	3.838	3.517588	2	2	2	597	67.6	5.85		0.122			3611914.63				2.16				1				1		Q9H2C0	NA	NA	' GAN
High	O15498	' YKT6	Synaptobrevin homolog YKT6 OS=Homo sapiens OX=9606 GN=YKT6	0.002	3.836	12.12121	2	2	2	198	22.4	6.92		0.389		2292318.38				0				1				1			O15498	NA	NA	' YKT6
High	Q04941	' PLP2	Proteolipid protein 2 OS=Homo sapiens OX=9606 GN=PLP2 PE=1 SV	0.002	3.835	18.42105	2	8	2	152	16.7	7.24		1.154	30519682.2		12086744.8	50298920.5	2.57		1.84	2.69	1		2	1	1		2	1	Q04941	NA	2.057102	' PLP2
High	Q9HAF1-3	' MEAF6	Isoform 3 of Chromatin modification-related protein MEAF6 OS=Ho	0.002	3.834	7.462687	1	2	1	201	22.7	8.28		0.259	643631.543				0				1				1				Q9HAF1-3	NA	NA	' MEAF6
High	O75608	' LYPLA1	Acyl-protein thioesterase 1 OS=Homo sapiens OX=9606 GN=LYPLA1	0.002	3.832	8.26087	2	4	2	230	24.7	6.77		0.425	4682259.16		7888032.75	5513363.31	1.65		0	0	1		1	1	1		1	1	O75608	NA	-0.51673	' LYPLA1
High	Q53GA4	' PHLDA2	Pleckstrin homology-like domain family A member 2 OS=Homo sapi	0.002	3.82	5.921053	1	2	1	152	17.1	9.17		0.233		5732859.72				1.86				1				1			Q53GA4	NA	NA	' PHLDA2
High	P01624	' IGKV3-15	Immunoglobulin kappa variable 3-15 OS=Homo sapiens OX=9606 G	0.002	3.814	26.08696	2	8	1	115	12.5	5.19		1.154		5092536.38	5637463.88			2.1	1.67	1.79		1	1	1		1	1	1	P01624	NA	NA	' IGKV3-15
High	O95249	' GOSR1	Golgi SNAP receptor complex member 1 OS=Homo sapiens OX=960	0.002	3.813	7.2	2	2	2	250	28.6	9.42		0.259		2664288.88				1.7				1				1			O95249	NA	NA	' GOSR1
High	P34949	' MPI	Mannose-6-phosphate isomerase OS=Homo sapiens OX=9606 GN=I	0.002	3.809	3.546099	1	2	1	423	46.6	5.95		0.129	5814220				0				1				1				P34949	NA	NA	' MPI
High	Q9Y6A9	' SPCS1	Signal peptidase complex subunit 1 OS=Homo sapiens OX=9606 GN	0.002	3.795	14.70588	1	5	1	102	11.8	9.31		2.162	8157249.63			12416804	2.89			3.57	1			1	1			1	Q9Y6A9	NA	NA	' SPCS1
High	Q5SRE5	' NUP188	Nucleoporin NUP188 homolog OS=Homo sapiens OX=9606 GN=NUF	0.003	3.767	1.429388	2	2	2	1749	195.9	6.73		0.053																	Q5SRE5	NA	NA	' NUP188
High	Q5SNT2	' TMEM201	Transmembrane protein 201 OS=Homo sapiens OX=9606 GN=TMEN	0.003	3.766	2.552553	2	3	2	666	72.2	9.22		0.16		2765037.13				0				1				1			Q5SNT2	NA	NA	' TMEM201
High	Q9P015	' MRPL15	39S ribosomal protein L15, mitochondrial OS=Homo sapiens OX=96	0.003	3.763	7.094595	2	2	2	296	33.4	10.01		0.274																	Q9P015	NA	NA	' MRPL15
High	Q9Y3X0	' CCDC9	Coiled-coil domain-containing protein 9 OS=Homo sapiens OX=9606	0.003	3.763	3.013183	1	1	1	531	59.7	5.4		0.08																	Q9Y3X0	NA	NA	' CCDC9
High	Q96II8-2	' LRCH3	Isoform 2 of Leucine-rich repeat and calponin homology domain-co	0.003	3.76	2.49066	2	2	2	803	89.1	6.7		0.129	901849.25				0				1				1				Q96II8-2	NA	NA	' LRCH3
High	Q9UBR2	' CTSZ	Cathepsin Z OS=Homo sapiens OX=9606 GN=CTSZ PE=1 SV=1	0.003	3.759	3.30033	1	3	1	303	33.8	7.11		0.155				19237436.9				2.68				1				1	Q9UBR2	NA	NA	' CTSZ
High	Q99417	' MYCBP	c-Myc-binding protein OS=Homo sapiens OX=9606 GN=MYCBP PE=1	0.003	3.752	16.50485	1	3	1	103	12	5.91		0.468		2782113.13			0	0			1	1			1	1			Q99417	NA	NA	' MYCBP
High	Q5T2T1	' MPP7	MAGUK p55 subfamily member 7 OS=Homo sapiens OX=9606 GN=N	0.003	3.748	2.604167	1	1	1	576	65.5	7.14		0.068	2519143.88				2.86				1				1				Q5T2T1	NA	NA	' MPP7
High	Q15003	' NCAPH	Condensin complex subunit 2 OS=Homo sapiens OX=9606 GN=NCAF	0.003	3.729	3.238866	2	2	2	741	82.5	5.06		0.129		4270202.03				2.1				1				1			Q15003	NA	NA	' NCAPH
High	Q8WVM0	' TFB1M	Dimethyladenosine transferase 1, mitochondrial OS=Homo sapiens	0.003	3.729	4.046243	1	1	1	346	39.5	9.26		0.101																	Q8WVM0	NA	NA	' TFB1M
High	Q7Z7M9	' GALNT5	Polypeptide N-acetylgalactosaminyltransferase 5 OS=Homo sapiens	0.003	3.721	2.553191	2	3	2	940	106.2	9.47		0.081	4620977.69				2.33	0			2	1			2	1			Q7Z7M9	NA	NA	' GALNT5
High	P20645	' M6PR	Cation-dependent mannose-6-phosphate receptor OS=Homo sapier	0.003	3.715	5.054152	1	2	1	277	31	5.83		0.136		2071426.56				2.22				1				1			P20645	NA	NA	' M6PR
High	Q9BTP6	' ZBED2	Zinc finger BED domain-containing protein 2 OS=Homo sapiens OX=	0.003	3.714	6.422018	1	1	1	218	25.1	7.06		0.212																	Q9BTP6	NA	NA	' ZBED2
High	O00178	' GTPBP1	GTP-binding protein 1 OS=Homo sapiens OX=9606 GN=GTPBP1 PE=	0.003	3.709	1.494768	1	1	1	669	72.4	8.34		0.072																	O00178	NA	NA	' GTPBP1
High	Q9UEE5	' STK17A	Serine/threonine-protein kinase 17A OS=Homo sapiens OX=9606 G	0.003	3.707	5.31401	1	2	1	414	46.5	5.15		0.179	6121018.34				2.4				1				1				Q9UEE5	NA	NA	' STK17A
High	P13640	' MT1G	Metallothionein-1G OS=Homo sapiens OX=9606 GN=MT1G PE=1 SV	0.003	3.687	19.35484	1	8	1	62	6.1	7.96		1.154	8733455.77	12446629.1	6093769.94	7929156.44	0	1.86	0	1.97	1	1	1	1	1	1	1	1	P13640	0.511131	0.379832	' MT1G
High	Q6UWP2	' DHRS11	Dehydrogenase/reductase SDR family member 11 OS=Homo sapien	0.003	3.687	5	1	3	1	260	28.3	6.64		0.145	7127477.81	4157955.25			2.12	1.76			1	1			1	1			Q6UWP2	-0.77752	NA	' DHRS11
High	Q8NEY1	' NAV1	Neuron navigator 1 OS=Homo sapiens OX=9606 GN=NAV1 PE=1 SV=	0.003	3.674	0.745871	1	1	1	1877	202.3	8.07		0.021																	Q8NEY1	NA	NA	' NAV1
High	Q06265-2	' EXOSC9	Isoform 2 of Exosome complex component RRP45 OS=Homo sapien	0.003	3.667	2.192982	1	1	1	456	50.8	5.3		0.129																	Q06265-2	NA	NA	' EXOSC9
High	O14744	' PRMT5	Protein arginine N-methyltransferase 5 OS=Homo sapiens OX=9606	0.003	3.667	4.081633	2	2	2	637	72.6	6.29		0.129		2199757.09				1.95				2				2			O14744	NA	NA	' PRMT5
High	Q9Y5X9	' LIPG	Endothelial lipase OS=Homo sapiens OX=9606 GN=LIPG PE=1 SV=1	0.003	3.666	3.6	1	1	1	500	56.8	7.93		0.096																	Q9Y5X9	NA	NA	' LIPG
High	Q9UMW8	' USP18	Ubl carboxyl-terminal hydrolase 18 OS=Homo sapiens OX=9606 GN=	0.003	3.66	3.225806	1	1	1	372	43	7.8		0.122				3506435.5				2.53				1				1	Q9UMW8	NA	NA	' USP18
High	Q9H0A8	' COMMD4	COMM domain-containing protein 4 OS=Homo sapiens OX=9606 GN	0.003	3.653	9.547739	1	1	1	199	21.8	7.31		0.179	4276328.41				2.93				1				1				Q9H0A8	NA	NA	' COMMD4
High	P60604	' UBE2G2	Ubiquitin-conjugating enzyme E2 G2 OS=Homo sapiens OX=9606 GN	0.003	3.638	13.33333	1	4	1	165	18.6	4.7		0.468		4403180.56			2.28	0			1	1			1	1			P60604	NA	NA	' UBE2G2
High	Q7Z434	' MAVS	Mitochondrial antiviral-signaling protein OS=Homo sapiens OX=960	0.003	3.637	2.592593	1	1	1	540	56.5	5.52		0.105																	Q7Z434	NA	NA	' MAVS
High	Q9NRR3	' CDC42SE2	CDC42 small effector protein 2 OS=Homo sapiens OX=9606 GN=CDC	0.003	3.628	44.04762	1	1	1	84	9.2	8.35		1.154	2737229.13				3.28				1				1				Q9NRR3	NA	NA	' CDC42SE2
High	Q8NB37-2	' GATD1	Isoform 2 of Glutamine amidotransferase-like class 1 domain-conta	0.003	3.625	4	1	1	1	250	26.8	6.4		0.166							2.62				1				1		Q8NB37-2	NA	NA	' GATD1
High	O43504	' LAMTOR5	Ragulator complex protein LAMTOR5 OS=Homo sapiens OX=9606 G	0.003	3.621	21.97802	1	1	1	91	9.6	4.87		0.585	6628881.75				3.14				1				1				O43504	NA	NA	' LAMTOR5
High	Q99759-2	' MAP3K3	Isoform 2 of Mitogen-activated protein kinase kinase kinase 3 OS=H	0.003	3.62	3.957382	2	3	1	657	74	9.06		0.103		3168784.48			1.66	2.01			1	1			1	1			Q99759-2	NA	NA	' MAP3K3
High	O15379-2	' HDAC3	Isoform 2 of Histone deacetylase 3 OS=Homo sapiens OX=9606 GN=	0.003	3.609	4.662005	2	4	1	429	49.1	5.33		0.186						0	1.6			1	1			1	1		O15379-2	NA	NA	' HDAC3
High	Q5TAQ9	' DCAF8	DDB1- and CUL4-associated factor 8 OS=Homo sapiens OX=9606 GN	0.003	3.601	3.350084	2	2	2	597	66.8	5.39		0.145							0				1				1		Q5TAQ9	NA	NA	' DCAF8
High	P02792	' FTL	Ferritin light chain OS=Homo sapiens OX=9606 GN=FTL PE=1 SV=2	0.003	3.594	8.571429	1	1	1	175	20	5.78		0.212	5890894.63				2.41				1				1				P02792	NA	NA	' FTL
High	O43813	' LANCL1	LanC-like protein 1 OS=Homo sapiens OX=9606 GN=LANCL1 PE=1 S	0.003	3.576	2.756892	1	1	1	399	45.3	7.75		0.11	22945115				1.98				1				1				O43813	NA	NA	' LANCL1
High	Q9H4G4	' GLIPR2	Golgi-associated plant pathogenesis-related protein 1 OS=Homo sa	0.003	3.573	8.441558	1	2	1	154	17.2	9.41		0.259				2666458.5				0				1				1	Q9H4G4	NA	NA	' GLIPR2
High	O60524	' NEMF	Nuclear export mediator factor NEMF OS=Homo sapiens OX=9606	0.003	3.565	0.929368	1	1	1	1076	122.9	6.35		0.038																	O60524	NA	NA	' NEMF
High	Q9UHA3	' RSL24D1	Probable ribosome biogenesis protein RLP24 OS=Homo sapiens OX=	0.003	3.564	7.361963	1	2	1	163	19.6	9.98		0.468		5242060.06				2.33				1				1			Q9UHA3	NA	NA	' RSL24D1
High	Q9P0J1-2	' PDP1	Isoform 2 of [Pyruvate dehydrogenase [acetyl-transferring]]-phosph	0.003	3.563	4.982206	2	2	2	562	63.7	6.81		0.133			2506036				4.39				2				2		Q9P0J1-2	NA	NA	' PDP1
High	P02679	' FGG	Fibrinogen gamma chain OS=Homo sapiens OX=9606 GN=FGG PE=1	0.003	3.562	1.986755	1	2	1	453	51.5	5.62		0.083	2294212.06				2.13				1				1				P02679	NA	NA	' FGG
High	Q9BXS4	' TMEM59	Transmembrane protein 59 OS=Homo sapiens OX=9606 GN=TMEM	0.003	3.56	8.668731	2	3	2	323	36.2	5.1		0.129				5424028.13				2.21				2				2	Q9BXS4	NA	NA	' TMEM59
High	Q6L8Q7	' PDE12	2',5'-phosphodiesterase 12 OS=Homo sapiens OX=9606 GN=PDE12	0.003	3.557	1.970443	1	4	1	609	67.3	6.57		0.089	3254163.94				1.75	1.64			1	1			1	1			Q6L8Q7	NA	NA	' PDE12
High	Q8IXK0-5	' PHC2	Isoform 5 of Polyhomeotic-like protein 2 OS=Homo sapiens OX=960	0.003	3.541	1.862631	1	2	1	859	90.8	8.69		0.077				3700867.13				2.27				1				1	Q8IXK0-5	NA	NA	' PHC2
High	O75976	' CPD	Carboxypeptidase D OS=Homo sapiens OX=9606 GN=CPD PE=1 SV=2	0.003	3.539	0.869565	1	1	1	1380	152.8	6.05		0.029																	O75976	NA	NA	' CPD
High	O75165	' DNAJC13	DnaJ homolog subfamily C member 13 OS=Homo sapiens OX=9606	0.003	3.539	1.604993	2	2	2	2243	254.3	6.74		0.036				1890239.66	0			0	1			1	1			1	O75165	NA	NA	' DNAJC13
High	Q9NRX4	' PHPT1	14 kDa phosphohistidine phosphatase OS=Homo sapiens OX=9606	0.003	3.531	8.8	1	2	1	125	13.8	6.07		0.292																	Q9NRX4	NA	NA	' PHPT1
High	Q9H061	' TMEM126A	Transmembrane protein 126A OS=Homo sapiens OX=9606 GN=TME	0.003	3.528	10.25641	1	2	1	195	21.5	9.26		0.212																	Q9H061	NA	NA	' TMEM126A
High	P30536	' TSPO	Translocator protein OS=Homo sapiens OX=9606 GN=TSPO PE=1 SV	0.003	3.523	9.467456	2	3	2	169	18.8	9.36		0.778		5911284.31		2309562.75		1.78		0		1		1		1		1	P30536	NA	NA	' TSPO
High	O95994	' AGR2	Anterior gradient protein 2 homolog OS=Homo sapiens OX=9606 GN	0.003	3.514	10.28571	2	2	2	175	20	9		0.52	1687809.97	38438734			0	0			1	1			1	1			O95994	4.509336	NA	' AGR2
High	Q9Y232	' CDYL	Chromodomain Y-like protein OS=Homo sapiens OX=9606 GN=CDYL	0.003	3.512	2.508361	1	1	1	598	66.4	9.45		0.07	1520937.31				1.7				1				1				Q9Y232	NA	NA	' CDYL
High	Q15154	' PCM1	Pericentriolar material 1 protein OS=Homo sapiens OX=9606 GN=P	0.003	3.504	0.444664	1	1	1	2024	228.4	5.02		0.023				1927617.88				0				1				1	Q15154	NA	NA	' PCM1
High	Q5T280	' SPOUT1	Putative methyltransferase C9orf114 OS=Homo sapiens OX=9606 G	0.003	3.489	3.723404	1	1	1	376	42	7.43		0.122																	Q5T280	NA	NA	' SPOUT1
High	P63218	' GNG5	Guanine nucleotide-binding protein G(I)/G(S)/G(O) subunit gamma-	0.003	3.458	13.23529	1	4	1	68	7.3	9.85		0.585		3671962.13			0	1.83			1	1			1	1			P63218	NA	NA	' GNG5
High	Q9BZZ5	' API5	Apoptosis inhibitor 5 OS=Homo sapiens OX=9606 GN=API5 PE=1 SV	0.003	3.456	2.480916	1	2	1	524	59	7.34		0.068		3435826.06				2.24				1				1			Q9BZZ5	NA	NA	' API5
High	Q3ZAQ7	' VMA21	Vacuolar ATPase assembly integral membrane protein VMA21 OS=H	0.003	3.438	11.88119	1	2	1	101	11.3	7.24		0.468				4393581.13				2.26				1				1	Q3ZAQ7	NA	NA	' VMA21
High	P00846	' MT-ATP6	ATP synthase subunit a OS=Homo sapiens OX=9606 GN=MT-ATP6 P	0.003	3.432	4.424779	1	1	1	226	24.8	10.1		0.585		11102343.3				2.87				1				1			P00846	NA	NA	' MT-ATP6
High	P18827	' SDC1	Syndecan-1 OS=Homo sapiens OX=9606 GN=SDC1 PE=1 SV=3	0.003	3.421	5.483871	1	3	1	310	32.4	4.63		0.233	23243790.5	8287830.06			3.27	3.19			1	1			1	1			P18827	-1.48778	NA	' SDC1
High	Q03426	' MVK	Mevalonate kinase OS=Homo sapiens OX=9606 GN=MVK PE=1 SV=1	0.003	3.414	3.030303	1	1	1	396	42.4	6.46		0.122				3951209.56				2.31				1				1	Q03426	NA	NA	' MVK
High	Q9HD42	' CHMP1A	Charged multivesicular body protein 1a OS=Homo sapiens OX=9606	0.003	3.405	8.163265	2	3	2	196	21.7	8.06		0.585		2427945.03		1681798.59		0		0		1		1		1		1	Q9HD42	NA	NA	' CHMP1A
High	Q7Z3B4	' NUP54	Nucleoporin p54 OS=Homo sapiens OX=9606 GN=NUP54 PE=1 SV=2	0.003	3.402	4.33925	2	2	2	507	55.4	7.02		0.179																	Q7Z3B4	NA	NA	' NUP54
High	P37173-2	' TGFBR2	Isoform 2 of TGF-beta receptor type-2 OS=Homo sapiens OX=9606	0.003	3.402	3.378378	1	1	1	592	67.4	5.88		0.061				4696505.63				2.67				1				1	P37173-2	NA	NA	' TGFBR2
High	Q8IZ83	' ALDH16A1	Aldehyde dehydrogenase family 16 member A1 OS=Homo sapiens	0.003	3.399	1.745636	1	1	1	802	85.1	6.79		0.072	2014410.38				2.88				1				1				Q8IZ83	NA	NA	' ALDH16A1
High	Q13126-2	' MTAP	Isoform 2 of S-methyl-5'-thioadenosine phosphorylase OS=Homo sa	0.003	3.395	6.358382	2	2	2	346	38.3	8.12		0.233			1641570.59				1.7				2				2		Q13126-2	NA	NA	' MTAP
High	P14174	' MIF	Macrophage migration inhibitory factor OS=Homo sapiens OX=9606	0.003	3.392	7.826087	1	7	1	115	12.5	7.88		0.778	14652604.4	24351638.7	32987685.5	51326702.3	2.47	2.82	2.59	2.49	1	1	1	1	1	1	1	1	P14174	0.732862	0.637782	' MIF
High	O75348	' ATP6V1G1	V-type proton ATPase subunit G 1 OS=Homo sapiens OX=9606 GN=	0.003	3.392	9.322034	1	4	1	118	13.7	8.79		0.468	3974622.06	3144954.28			2.44	1.66			1	1			1	1			O75348	-0.33778	NA	' ATP6V1G1
High	Q9H8H3	' METTL7A	Methyltransferase-like protein 7A OS=Homo sapiens OX=9606 GN=	0.003	3.389	6.147541	1	1	1	244	28.3	8.38		0.212																	Q9H8H3	NA	NA	' METTL7A
High	Q5T160	' RARS2	Probable arginine--tRNA ligase, mitochondrial OS=Homo sapiens OX	0.003	3.389	2.076125	1	1	1	578	65.5	8.21		0.059				2625692.13				2.08				1				1	Q5T160	NA	NA	' RARS2
High	P08047	' SP1	Transcription factor Sp1 OS=Homo sapiens OX=9606 GN=SP1 PE=1	0.003	3.386	3.566879	1	2	1	785	80.6	7.34		0.166	2784736.56				0				1				1				P08047	NA	NA	' SP1
High	O15321	' TM9SF1	Transmembrane 9 superfamily member 1 OS=Homo sapiens OX=96	0.003	3.384	1.485149	1	1	1	606	68.8	7.17		0.089					2.2				1				1				O15321	NA	NA	' TM9SF1
High	Q9NTZ6	' RBM12	RNA-binding protein 12 OS=Homo sapiens OX=9606 GN=RBM12 PE	0.003	3.37	1.072961	1	1	1	932	97.3	8.63		0.066						1.8				1				1			Q9NTZ6	NA	NA	' RBM12
High	Q13415	' ORC1	Origin recognition complex subunit 1 OS=Homo sapiens OX=9606 G	0.003	3.366	1.393728	1	1	1	861	97.3	9.22		0.045																	Q13415	NA	NA	' ORC1
High	O14929	' HAT1	Histone acetyltransferase type B catalytic subunit OS=Homo sapiens	0.003	3.363	3.102625	1	1	1	419	49.5	5.69		0.105						1.86				1				1			O14929	NA	NA	' HAT1
High	O15533-3	' TAPBP	Isoform 3 of Tapasin OS=Homo sapiens OX=9606 GN=TAPBP	0.003	3.358	4.365079	2	3	2	504	53.9	7.23		0.259		2622327				0				1				1			O15533-3	NA	NA	' TAPBP
High	Q6Y1H2	' HACD2	Very-long-chain (3R)-3-hydroxyacyl-CoA dehydratase 2 OS=Homo sa	0.003	3.352	3.937008	1	4	1	254	28.4	9.55		0.194	3507093.23		986196.125		2.89		2.27		1		1		1		1		Q6Y1H2	NA	NA	' HACD2
High	P42345	' MTOR	Serine/threonine-protein kinase mTOR OS=Homo sapiens OX=9606	0.003	3.351	0.902315	2	3	2	2549	288.7	7.17		0.015	779599.516	5211013.13			0	0			1	1			1	1			P42345	2.740759	NA	' MTOR
High	Q9Y241-2	' HIGD1A	Isoform 2 of HIG1 domain family member 1A, mitochondrial OS=Ho	0.003	3.35	20.56075	1	1	1	107	11.8	9.44		0.585		2425333.06				0				1				1			Q9Y241-2	NA	NA	' HIGD1A
High	Q6P1X5	' TAF2	Transcription initiation factor TFIID subunit 2 OS=Homo sapiens OX=	0.003	3.348	1.918265	2	3	2	1199	136.9	8.19		0.065				1410790.59				2.02				2				2	Q6P1X5	NA	NA	' TAF2
High	Q15043	' SLC39A14	Zinc transporter ZIP14 OS=Homo sapiens OX=9606 GN=SLC39A14 P	0.003	3.347	2.642276	1	3	1	492	54.2	5.33		0.166		3070045.75		5267206.63		1.99		2.23		1		1		1		1	Q15043	NA	NA	' SLC39A14
High	Q01151	' CD83	CD83 antigen OS=Homo sapiens OX=9606 GN=CD83 PE=1 SV=1	0.003	3.343	4.878049	1	2	1	205	23	8.18		0.194				4558466.13				2.29				1				1	Q01151	NA	NA	' CD83
High	Q9UHD1	' CHORDC1	Cysteine and histidine-rich domain-containing protein 1 OS=Homo s	0.003	3.341	9.638554	2	2	2	332	37.5	7.87		0.212		1679387.56			0	2.28			1	1			1	1			Q9UHD1	NA	NA	' CHORDC1
High	P42226	' STAT6	Signal transducer and activator of transcription 6 OS=Homo sapiens	0.003	3.333	1.180638	1	2	1	847	94.1	6.23		0.059				1195526.88				0				1				1	P42226	NA	NA	' STAT6
High	Q9UHR5	' SAP30BP	SAP30-binding protein OS=Homo sapiens OX=9606 GN=SAP30BP PE	0.003	3.333	4.87013	1	1	1	308	33.9	4.84		0.145	3933886.38				2.56				1				1				Q9UHR5	NA	NA	' SAP30BP
High	P15954	' COX7C	Cytochrome c oxidase subunit 7C, mitochondrial OS=Homo sapiens	0.003	3.333	14.28571	1	4	1	63	7.2	10.27		0.468			5042001.91	6728104.46			1.83	1.87			1	1			1	1	P15954	NA	0.416203	' COX7C
High	O95817	' BAG3	BAG family molecular chaperone regulator 3 OS=Homo sapiens OX=	0.003	3.332	6.086957	2	2	2	575	61.6	6.95		0.068																	O95817	NA	NA	' BAG3
High	Q6NZ67	' MZT2B	Mitotic-spindle organizing protein 2B OS=Homo sapiens OX=9606 G	0.003	3.33	12.65823	2	2	2	158	16.2	10.15		0.585								0				1				1	Q6NZ67	NA	NA	' MZT2B
High	P05120	' SERPINB2	Plasminogen activator inhibitor 2 OS=Homo sapiens OX=9606 GN=S	0.003	3.324	3.614458	1	4	1	415	46.6	5.63		0.105	3812251.91	4535885.88			0	1.82			1	1			1	1			P05120	0.250741	NA	' SERPINB2
High	Q7L5N7	' LPCAT2	Lysophosphatidylcholine acyltransferase 2 OS=Homo sapiens OX=96	0.003	3.324	1.654412	1	1	1	544	60.2	6.55		0.075																	Q7L5N7	NA	NA	' LPCAT2
High	Q9P2X0-2	' DPM3	Isoform 2 of Dolichol-phosphate mannosyltransferase subunit 3 OS=	0.003	3.304	8.196721	1	3	1	122	13.3	8.35		0.585	3390289.25	3906656.5			1.77	2.34			1	1			1	1			Q9P2X0-2	0.204526	NA	' DPM3
High	Q9NPA0	' EMC7	ER membrane protein complex subunit 7 OS=Homo sapiens OX=960	0.003	3.303	5.371901	1	2	1	242	26.5	9.25		0.194		6061433.63				2.04				1				1			Q9NPA0	NA	NA	' EMC7
High	Q9NZN8	' CNOT2	CCR4-NOT transcription complex subunit 2 OS=Homo sapiens OX=9	0.003	3.302	1.481481	1	1	1	540	59.7	7.66		0.086																	Q9NZN8	NA	NA	' CNOT2
High	O95456	' PSMG1	Proteasome assembly chaperone 1 OS=Homo sapiens OX=9606 GN=	0.003	3.299	4.861111	1	5	1	288	32.8	7.17		0.145		2046147.19	6607922	8908637.25		2.1	2.46	2.33		1	1	1		1	1	1	O95456	NA	0.431008	' PSMG1
High	Q9UPT8	' ZC3H4	Zinc finger CCCH domain-containing protein 4 OS=Homo sapiens OX	0.003	3.296	1.765157	2	5	1	1303	140.2	6.27		0.045	2123825.53				2.13		1.86		2		1		2		1		Q9UPT8	NA	NA	' ZC3H4
High	Q8NFH3	' NUP43	Nucleoporin Nup43 OS=Homo sapiens OX=9606 GN=NUP43 PE=1 S	0.003	3.295	4.473684	1	2	1	380	42.1	5.63		0.194	2232971.56	2232613.94			0	2.04			1	1			1	1			Q8NFH3	-0.00023	NA	' NUP43
High	Q9UGP4	' LIMD1	LIM domain-containing protein 1 OS=Homo sapiens OX=9606 GN=LI	0.003	3.292	2.366864	1	1	1	676	72.1	6.65		0.064				1517894.75				0				1				1	Q9UGP4	NA	NA	' LIMD1
High	Q6UXV4	' APOOL	MICOS complex subunit MIC27 OS=Homo sapiens OX=9606 GN=APO	0.003	3.29	4.477612	1	1	1	268	29.1	9.52		0.155				586354.469				1.65				1				1	Q6UXV4	NA	NA	' APOOL
High	Q9UEW8	' STK39	STE20/SPS1-related proline-alanine-rich protein kinase OS=Homo sa	0.003	3.288	3.853211	2	2	1	545	59.4	6.29		0.186																	Q9UEW8	NA	NA	' STK39
High	Q05707	' COL14A1	Collagen alpha-1(XIV) chain OS=Homo sapiens OX=9606 GN=COL14	0.003	3.276	2.282851	2	12	2	1796	193.4	5.3		0.053	2322026.39		552426530		3.59		4.17		2		1		7		1		Q05707	NA	NA	' COL14A1
High	P00441	' SOD1	Superoxide dismutase [Cu-Zn] OS=Homo sapiens OX=9606 GN=SOD	0.003	3.273	9.090909	1	5	1	154	15.9	6.13		0.212		5970274.91	6635168.94	6602557.88		1.68	1.78	2.54		1	1	1		1	1	1	P00441	NA	-0.00711	' SOD1
High	Q9H9A6	' LRRC40	Leucine-rich repeat-containing protein 40 OS=Homo sapiens OX=96	0.003	3.269	1.66113	1	1	1	602	68.2	6.43		0.061		1230150.56				1.94				1				1			Q9H9A6	NA	NA	' LRRC40
High	Q8NDD1-	' C1orf131	Isoform 2 of Uncharacterized protein C1orf131 OS=Homo sapiens O	0.003	3.269	3.741497	1	1	1	294	32.7	9.92		0.145																	Q8NDD1-	NA	NA	' C1orf131
High	Q9C005	' DPY30	Protein dpy-30 homolog OS=Homo sapiens OX=9606 GN=DPY30 PE=	0.003	3.264	16.16162	1	2	1	99	11.2	4.88		0.468	1223884.06				0				1				1				Q9C005	NA	NA	' DPY30
High	A6NK75	' ZNF98	Zinc finger protein 98 OS=Homo sapiens OX=9606 GN=ZNF98 PE=2	0.003	3.262	9.965035	2	2	1	572	65.8	9.17	Phospho	0.047				5597376.2				0				1				1	A6NK75	NA	NA	' ZNF98
High	Q9NZJ0	' DTL	Denticleless protein homolog OS=Homo sapiens OX=9606 GN=DTL	0.003	3.259	2.876712	2	2	2	730	79.4	8.87		0.122			697626.422	2380282.81			0	2.07			1	1			1	1	Q9NZJ0	NA	1.770606	' DTL
High	Q7Z5J4	' RAI1	Retinoic acid-induced protein 1 OS=Homo sapiens OX=9606 GN=RAI	0.003	3.245	1.731375	2	2	2	1906	203.2	8.79		0.051					0				1				1				Q7Z5J4	NA	NA	' RAI1
High	Q9UH17	' APOBEC3B	DNA dC->dU-editing enzyme APOBEC-3B OS=Homo sapiens OX=960	0.004	3.231	6.282723	2	3	2	382	45.9	6.06		0.222																	Q9UH17	NA	NA	' APOBEC3B
High	Q8NC42	' RNF149	E3 ubiquitin-protein ligase RNF149 OS=Homo sapiens OX=9606 GN=	0.004	3.229	2.75	1	1	1	400	43.1	6.54		0.105								2.59				1				1	Q8NC42	NA	NA	' RNF149
High	P46109	' CRKL	Crk-like protein OS=Homo sapiens OX=9606 GN=CRKL PE=1 SV=1	0.004	3.224	4.950495	1	2	1	303	33.8	6.74		0.129				4852485.88				3.06				1				1	P46109	NA	NA	' CRKL
High	Q6PK04	' CCDC137	Coiled-coil domain-containing protein 137 OS=Homo sapiens OX=96	0.004	3.217	6.228374	1	2	1	289	33.2	10.93		0.122		3945186.75				2.17				1				1			Q6PK04	NA	NA	' CCDC137
High	P58546	' MTPN	Myotrophin OS=Homo sapiens OX=9606 GN=MTPN PE=1 SV=2	0.004	3.204	14.40678	1	5	1	118	12.9	5.52		0.334	7257717.13	7184991.5	2288799.34		0	0	0		1	1	1		1	1	1		P58546	-0.01453	NA	' MTPN
High	P30084	' ECHS1	Enoyl-CoA hydratase, mitochondrial OS=Homo sapiens OX=9606 GN	0.004	3.2	4.137931	1	2	1	290	31.4	8.07		0.122		5918251.25				2.23				1				1			P30084	NA	NA	' ECHS1
High	Q6PJT7	' ZC3H14	Zinc finger CCCH domain-containing protein 14 OS=Homo sapiens O	0.004	3.198	3.668478	2	3	2	736	82.8	7.31		0.105			1108652.25		0		0		1		1		1		1		Q6PJT7	NA	NA	' ZC3H14
High	Q6UY14-3	' ADAMTSL4	Isoform 3 of ADAMTS-like protein 4 OS=Homo sapiens OX=9606 GN	0.004	3.185	1.549681	1	1	1	1097	118.7	8.34		0.038																	Q6UY14-3	NA	NA	' ADAMTSL4
High	Q9Y5K3	' PCYT1B	Choline-phosphate cytidylyltransferase B OS=Homo sapiens OX=960	0.004	3.179	3.252033	1	2	1	369	41.9	6.4		0.096	2451097.34				1.99				1				1				Q9Y5K3	NA	NA	' PCYT1B
High	Q9BUN8	' DERL1	Derlin-1 OS=Homo sapiens OX=9606 GN=DERL1 PE=1 SV=1	0.004	3.167	4.38247	1	5	1	251	28.8	9.51		0.212	5216510.75	13168914.6	7785261	40697326.5	1.93	2.27	1.79	2.51	1	1	1	1	1	1	1	1	Q9BUN8	1.335979	2.386117	' DERL1
High	Q14680	' MELK	Maternal embryonic leucine zipper kinase OS=Homo sapiens OX=96	0.004	3.165	2.150538	1	1	1	651	74.6	8.72		0.061	3662633.38				2.86				1				1				Q14680	NA	NA	' MELK
High	Q7Z407	' CSMD3	CUB and sushi domain-containing protein 3 OS=Homo sapiens OX=9	0.004	3.165	0.701376	2	2	2	3707	405.7	6	Phospho	0.015		5245452.41				2.35				1				1			Q7Z407	NA	NA	' CSMD3
High	Q99501	' GAS2L1	GAS2-like protein 1 OS=Homo sapiens OX=9606 GN=GAS2L1 PE=1 S	0.004	3.163	6.167401	2	2	2	681	72.7	10.05		0.105			1914140.22				0				2				2		Q99501	NA	NA	' GAS2L1

High	Q12933-2	' TRAF2	Isoform 2 of TNF receptor-associated factor 2 OS=Homo sapiens OX	0.004	3.161	2.169982	1	1	1	553	61.3	7.21		0.066																	Q12933-2	NA	NA	' TRAF2	Isoform 2 of TNF receptor-associated factor 2 OS=Homo sapiens OX=9606 GN=TRAF2
High	Q8NBU5	' ATAD1	ATPase family AAA domain-containing protein 1 OS=Homo sapiens (	0.004	3.156	5.540166	1	1	1	361	40.7	6.9		0.116				7699050.13				4.07				1				1	Q8NBU5	NA	NA	' ATAD1	ATPase family AAA domain-containing protein 1 OS=Homo sapiens OX=9606 GN=ATAD1 PE=1 SV=1
High	P30530	' AXL	Tyrosine-protein kinase receptor UFO OS=Homo sapiens OX=9606 G	0.004	3.153	1.230425	1	1	1	894	98.3	5.39		0.056				2769144.13				2.25				1				1	P30530	NA	NA	' AXL	Tyrosine-protein kinase receptor UFO OS=Homo sapiens OX=9606 GN=AXL PE=1 SV=4
High	Q9BX10	' GTPBP2	GTP-binding protein 2 OS=Homo sapiens OX=9606 GN=GTPBP2 PE=1	0.004	3.147	1.827243	1	2	1	602	65.7	8.05		0.072																	Q9BX10	NA	NA	' GTPBP2	GTP-binding protein 2 OS=Homo sapiens OX=9606 GN=GTPBP2 PE=1 SV=1
High	Q96EU6	' RRP36	Ribosomal RNA processing protein 36 homolog OS=Homo sapiens O	0.004	3.146	5.791506	1	2	1	259	29.8	10.17		0.136	5174759.5				0				1				1				Q96EU6	NA	NA	' RRP36	Ribosomal RNA processing protein 36 homolog OS=Homo sapiens OX=9606 GN=RRP36 PE=1 SV=1
High	O00233	' PSMD9	26S proteasome non-ATPase regulatory subunit 9 OS=Homo sapien	0.004	3.145	9.41704	2	2	2	223	24.7	6.95		0.52																	O00233	NA	NA	' PSMD9	26S proteasome non-ATPase regulatory subunit 9 OS=Homo sapiens OX=9606 GN=PSMD9 PE=1 SV=3
High	Q9NQG1	' MANBAL	Protein MANBAL OS=Homo sapiens OX=9606 GN=MANBAL PE=1 SV	0.005	3.138	14.11765	1	1	1	85	9.5	9.16		0.585																	Q9NQG1	NA	NA	' MANBAL	Protein MANBAL OS=Homo sapiens OX=9606 GN=MANBAL PE=1 SV=1
High	P63098	' PPP3R1	Calcineurin subunit B type 1 OS=Homo sapiens OX=9606 GN=PPP3R	0.005	3.129	5.882353	1	2	1	170	19.3	4.81		0.259	5564549.31				2.25				1				1				P63098	NA	NA	' PPP3R1	Calcineurin subunit B type 1 OS=Homo sapiens OX=9606 GN=PPP3R1 PE=1 SV=2
High	Q9P270	' SLAIN2	SLAIN motif-containing protein 2 OS=Homo sapiens OX=9606 GN=SI	0.005	3.128	2.92599	1	4	1	581	62.5	9.45		0.066			2109371.19	3161675.75			0	2.07			1	1			1	1	Q9P270	NA	0.583876	' SLAIN2	SLAIN motif-containing protein 2 OS=Homo sapiens OX=9606 GN=SLAIN2 PE=1 SV=2
High	Q9BXB5	' OSBPL10	Oxysterol-binding protein-related protein 10 OS=Homo sapiens OX=	0.005	3.128	3.926702	2	2	2	764	83.9	8.31		0.054		2787393			2.13	1.66			1	1			1	1			Q9BXB5	NA	NA	' OSBPL10	Oxysterol-binding protein-related protein 10 OS=Homo sapiens OX=9606 GN=OSBPL10 PE=1 SV=2
High	P27482	' CALML3	Calmodulin-like protein 3 OS=Homo sapiens OX=9606 GN=CALML3 I	0.005	3.114	18.12081	2	7	1	149	16.9	4.42		0.52							2.24	2.06			1	1			1	1	P27482	NA	NA	' CALML3	Calmodulin-like protein 3 OS=Homo sapiens OX=9606 GN=CALML3 PE=1 SV=2
High	Q9BYM8	' RBCK1	RanBP-type and C3HC4-type zinc finger-containing protein 1 OS=Ho	0.005	3.108	3.529412	1	1	1	510	57.5	5.67		0.07																	Q9BYM8	NA	NA	' RBCK1	RanBP-type and C3HC4-type zinc finger-containing protein 1 OS=Homo sapiens OX=9606 GN=RBCK1 PE=1 SV=2
High	O95154	' AKR7A3	Aflatoxin B1 aldehyde reductase member 3 OS=Homo sapiens OX=9	0.005	3.107	5.740181	1	4	1	331	37.2	7.15		0.166	16523000	11655104.3			2.43	2.04			1	1			1	1			O95154	-0.50351	NA	' AKR7A3	Aflatoxin B1 aldehyde reductase member 3 OS=Homo sapiens OX=9606 GN=AKR7A3 PE=1 SV=2
High	Q6UWP8	' SBSN	Suprabasin OS=Homo sapiens OX=9606 GN=SBSN PE=1 SV=2	0.005	3.102	9.152542	1	1	1	590	60.5	7.01		0.072				1629482.03				0				1				1	Q6UWP8	NA	NA	' SBSN	Suprabasin OS=Homo sapiens OX=9606 GN=SBSN PE=1 SV=2
High	P12081	' HARS	Histidine--tRNA ligase, cytoplasmic OS=Homo sapiens OX=9606 GN=	0.005	3.094	3.732809	2	2	2	509	57.4	5.88		0.141																	P12081	NA	NA	' HARS	Histidine--tRNA ligase, cytoplasmic OS=Homo sapiens OX=9606 GN=HARS PE=1 SV=2
High	Q96EL2	' MRPS24	28S ribosomal protein S24, mitochondrial OS=Homo sapiens OX=96	0.005	3.091	4.790419	1	4	1	167	19	9.38		0.212	6338480.29	5729594			0	1.96			1	1			1	1			Q96EL2	-0.1457	NA	' MRPS24	28S ribosomal protein S24, mitochondrial OS=Homo sapiens OX=9606 GN=MRPS24 PE=1 SV=1
High	Q10589	' BST2	Bone marrow stromal antigen 2 OS=Homo sapiens OX=9606 GN=BST	0.005	3.083	8.333333	1	1	1	180	19.8	5.6		0.233																	Q10589	NA	NA	' BST2	Bone marrow stromal antigen 2 OS=Homo sapiens OX=9606 GN=BST2 PE=1 SV=1
High	Q13637	' RAB32	Ras-related protein Rab-32 OS=Homo sapiens OX=9606 GN=RAB32 I	0.005	3.082	7.555556	1	1	1	225	25	6.54		0.155				1476612.75				2.74				1				1	Q13637	NA	NA	' RAB32	Ras-related protein Rab-32 OS=Homo sapiens OX=9606 GN=RAB32 PE=1 SV=3
High	Q9Y530	' OARD1	O-acetyl-ADP-ribose deacetylase 1 OS=Homo sapiens OX=9606 GN=	0.005	3.072	5.263158	1	1	1	152	17	8.31		0.194	2244814.56				1.92				1				1				Q9Y530	NA	NA	' OARD1	O-acetyl-ADP-ribose deacetylase 1 OS=Homo sapiens OX=9606 GN=OARD1 PE=1 SV=2
High	Q9BU23	' LMF2	Lipase maturation factor 2 OS=Homo sapiens OX=9606 GN=LMF2 PE	0.005	3.071	4.101839	2	3	2	707	79.6	10.1		0.179			1860608.04	2849704.39		0	0	1.69		1	1	1		1	1	1	Q9BU23	NA	0.615038	' LMF2	Lipase maturation factor 2 OS=Homo sapiens OX=9606 GN=LMF2 PE=1 SV=2
High	Q96DU7	' ITPKC	Inositol-trisphosphate 3-kinase C OS=Homo sapiens OX=9606 GN=IT	0.005	3.068	3.367496	1	1	1	683	75.2	5.14		0.068	4198802.69				2.82				1				1				Q96DU7	NA	NA	' ITPKC	Inositol-trisphosphate 3-kinase C OS=Homo sapiens OX=9606 GN=ITPKC PE=1 SV=1
High	Q16877	' PFKFB4	6-phosphofructo-2-kinase/fructose-2,6-bisphosphatase 4 OS=Homo	0.005	3.067	5.33049	2	2	2	469	54	6.62		0.15																	Q16877	NA	NA	' PFKFB4	6-phosphofructo-2-kinase/fructose-2,6-bisphosphatase 4 OS=Homo sapiens OX=9606 GN=PFKFB4 PE=2 SV=6
High	Q8N1B4	' VPS52	Vacuolar protein sorting-associated protein 52 homolog OS=Homo s	0.005	3.062	1.521438	1	1	1	723	82.2	5.99		0.054				2705412.38				1.89				1				1	Q8N1B4	NA	NA	' VPS52	Vacuolar protein sorting-associated protein 52 homolog OS=Homo sapiens OX=9606 GN=VPS52 PE=1 SV=1
High	Q9UKS6	' PACSIN3	Protein kinase C and casein kinase substrate in neurons protein 3 O:	0.005	3.056	3.773585	1	2	1	424	48.5	6.18		0.089				2185337.25				2.86				1				1	Q9UKS6	NA	NA	' PACSIN3	Protein kinase C and casein kinase substrate in neurons protein 3 OS=Homo sapiens OX=9606 GN=PACSIN3 PE=1 SV=2
High	Q15021	' NCAPD2	Condensin complex subunit 1 OS=Homo sapiens OX=9606 GN=NCAF	0.005	3.049	1.356174	1	2	1	1401	157.1	6.61		0.026		2923431.5				0				1				1			Q15021	NA	NA	' NCAPD2	Condensin complex subunit 1 OS=Homo sapiens OX=9606 GN=NCAPD2 PE=1 SV=3
High	Q9HBH0	' RHOF	Rho-related GTP-binding protein RhoF OS=Homo sapiens OX=9606 (	0.005	3.045	9.952607	2	2	2	211	23.6	8.65		0.468																	Q9HBH0	NA	NA	' RHOF	Rho-related GTP-binding protein RhoF OS=Homo sapiens OX=9606 GN=RHOF PE=1 SV=1
High	Q16563	' SYPL1	Synaptophysin-like protein 1 OS=Homo sapiens OX=9606 GN=SYPL1	0.005	3.043	4.247104	1	6	1	259	28.5	8.43		0.212	11272784.4		3664878.38	5762912.34	2.31	2.22	2.14	2.41	1	1	1	1	1	1	1	1	Q16563	NA	0.653033	' SYPL1	Synaptophysin-like protein 1 OS=Homo sapiens OX=9606 GN=SYPL1 PE=1 SV=1
High	P54709	' ATP1B3	Sodium/potassium-transporting ATPase subunit beta-3 OS=Homo sa	0.005	3.04	5.376344	1	1	1	279	31.5	8.35		0.166																	P54709	NA	NA	' ATP1B3	Sodium/potassium-transporting ATPase subunit beta-3 OS=Homo sapiens OX=9606 GN=ATP1B3 PE=1 SV=1
High	O43324	' EEF1E1	Eukaryotic translation elongation factor 1 epsilon-1 OS=Homo sapie	0.005	3.038	5.747126	1	1	1	174	19.8	8.54		0.166																	O43324	NA	NA	' EEF1E1	Eukaryotic translation elongation factor 1 epsilon-1 OS=Homo sapiens OX=9606 GN=EEF1E1 PE=1 SV=1
High	Q9UKV0-7	' HDAC9	Isoform 7 of Histone deacetylase 9 OS=Homo sapiens OX=9606 GN=	0.005	3.032	1.683817	1	1	1	1069	117.5	6.6		0.043																	Q9UKV0-7	NA	NA	' HDAC9	Isoform 7 of Histone deacetylase 9 OS=Homo sapiens OX=9606 GN=HDAC9
High	Q96JM3	' CHAMP1	Chromosome alignment-maintaining phosphoprotein 1 OS=Homo sa	0.005	3.028	3.325123	2	2	2	812	89	8.44		0.083		2787657.63				0				1				1			Q96JM3	NA	NA	' CHAMP1	Chromosome alignment-maintaining phosphoprotein 1 OS=Homo sapiens OX=9606 GN=CHAMP1 PE=1 SV=2
High	P20618	' PSMB1	Proteasome subunit beta type-1 OS=Homo sapiens OX=9606 GN=PS	0.005	3.023	5.809129	1	5	1	241	26.5	8.13		0.166		4943087.47	5846727.13	4492786.25		0	0	0		1	1	1		1	1	1	P20618	NA	-0.38002	' PSMB1	Proteasome subunit beta type-1 OS=Homo sapiens OX=9606 GN=PSMB1 PE=1 SV=2
High	Q99707	' MTR	Methionine synthase OS=Homo sapiens OX=9606 GN=MTR PE=1 SV	0.005	3.02	0.948617	1	1	1	1265	140.4	5.58		0.03								0				1				1	Q99707	NA	NA	' MTR	Methionine synthase OS=Homo sapiens OX=9606 GN=MTR PE=1 SV=2
High	Q9NXE8	' CWC25	Pre-mRNA-splicing factor CWC25 homolog OS=Homo sapiens OX=96	0.005	3.02	4.941176	2	2	2	425	49.6	10.18		0.212			1703305.72				0				2				2		Q9NXE8	NA	NA	' CWC25	Pre-mRNA-splicing factor CWC25 homolog OS=Homo sapiens OX=9606 GN=CWC25 PE=1 SV=1
High	P46199	' MTIF2	Translation initiation factor IF-2, mitochondrial OS=Homo sapiens O	0.005	3.019	1.925722	1	1	1	727	81.3	7.15		0.061		3094882.81				0				1				1			P46199	NA	NA	' MTIF2	Translation initiation factor IF-2, mitochondrial OS=Homo sapiens OX=9606 GN=MTIF2 PE=1 SV=2
[illegible]																																			

Medium	Q13084	' MRPL28	39S ribosomal protein L28, mitochondrial OS=Homo sapiens OX=96	0.024	2.04	8.984375	1	2	1	256	30.1	8.29		0.136
Medium	Q8N983-7	' MRPL43	Isoform 6 of 39S ribosomal protein L43, mitochondrial OS=Homo sa	0.025	2.034	4.597701	1	1	1	261	28.7	8.29		0.166
Medium	Q8WYA6	' CTNNBL1	Beta-catenin-like protein 1 OS=Homo sapiens OX=9606 GN=CTNNBL	0.026	2.029	1.420959	1	4	1	563	65.1	5.05		0.089
Medium	Q9HAN9	' NMNAT1	Nicotinamide/nicotinic acid mononucleotide adenylyltransferase 1 (	0.026	2.028	3.942652	1	1	1	279	31.9	8.87		0.129
Medium	Q6P587-3	' FAHD1	Isoform 3 of Acylpyruvase FAHD1, mitochondrial OS=Homo sapiens	0.026	2.027	5.645161	1	1	1	248	27.1	7.69		0.136
Medium	Q9NVA1	' UQCC1	Ubiquinol-cytochrome-c reductase complex assembly factor 1 OS=H	0.026	2.022	6.020067	1	1	1	299	34.6	8.91		0.145
Medium	Q8TF68	' ZNF384	Zinc finger protein 384 OS=Homo sapiens OX=9606 GN=ZNF384 PE=	0.027	2.018	2.079723	1	1	1	577	63.2	8.95		0.083
Medium	Q9BSR8	' YIPF4	Protein YIPF4 OS=Homo sapiens OX=9606 GN=YIPF4 PE=1 SV=1	0.029	2.011	3.688525	1	1	1	244	27.1	4.65		0.259
Medium	O43688-2	' PLPP2	Isoform 2 of Phospholipid phosphatase 2 OS=Homo sapiens OX=960	0.029	2.01	3.236246	1	1	1	309	34.4	8.62		0.155
Medium	Q12768	' WASHC5	WASH complex subunit 5 OS=Homo sapiens OX=9606 GN=WASHC5	0.029	2.001	1.121657	1	1	1	1159	134.2	6.98		0.031
Medium	Q01968	' OCRL	Inositol polyphosphate 5-phosphatase OCRL-1 OS=Homo sapiens O	0.03	1.997	1.109878	1	1	1	901	104.1	6.55		0.046
Medium	Q9Y697	' NFS1	Cysteine desulfurase, mitochondrial OS=Homo sapiens OX=9606 GN	0.03	1.99	3.501094	1	2	1	457	50.2	8.31		0.093
Medium	Q9UIW2	' PLXNA1	Plexin-A1 OS=Homo sapiens OX=9606 GN=PLXNA1 PE=1 SV=3	0.03	1.987	0.474684	1	1	1	1896	210.9	6.92		0.021
Medium	Q96HY6	' DDRGK1	DDRGK domain-containing protein 1 OS=Homo sapiens OX=9606 GN	0.03	1.981	4.140127	1	1	1	314	35.6	5.12		0.155
Medium	Q92890-1	' UFD1	Isoform Long of Ubiquitin recognition factor in ER-associated degrad	0.03	1.98	4.373178	1	1	1	343	38.7	6.34		0.136
Medium	Q2TB10	' ZNF800	Zinc finger protein 800 OS=Homo sapiens OX=9606 GN=ZNF800 PE=	0.03	1.972	3.463855	1	1	1	664	75.2	9.47		0.07
Medium	Q9Y6Y0	' IVNS1ABP	Influenza virus NS1A-binding protein OS=Homo sapiens OX=9606 GN	0.03	1.971	1.401869	1	2	1	642	71.7	5.53		0.072
Medium	Q9BRJ2	' MRPL45	39S ribosomal protein L45, mitochondrial OS=Homo sapiens OX=96	0.031	1.963	9.150327	1	4	1	306	35.3	9.03		0.116
Medium	Q9Y5J5	' PHLDA3	Pleckstrin homology-like domain family A member 3 OS=Homo sapi	0.031	1.962	6.299213	1	2	1	127	13.9	9.67		0.212
Medium	P32519	' ELF1	ETS-related transcription factor Elf-1 OS=Homo sapiens OX=9606 GN	0.031	1.958	2.746365	1	1	1	619	67.5	5.21		0.101
Medium	Q13907-2	' IDI1	Isoform 2 of Isopentenyl-diphosphate Delta-isomerase 1 OS=Homo	0.031	1.956	4.225352	1	2	1	284	32.5	7.88		0.116
Medium	Q9Y2T3-3	' GDA	Isoform 3 of Guanine deaminase OS=Homo sapiens OX=9606 GN=G	0.032	1.944	2.547771	1	1	1	471	52.8	5.85		0.08
Medium	O14949	' UQCRQ	Cytochrome b-c1 complex subunit 8 OS=Homo sapiens OX=9606 GN	0.032	1.941	15.85366	1	2	1	82	9.9	10.08		0.468
Medium	P48556	' PSMD8	26S proteasome non-ATPase regulatory subunit 8 OS=Homo sapien	0.032	1.94	2.285714	1	2	1	350	39.6	9.7		0.101
Medium	O75600-2	' GCAT	Isoform 2 of 2-amino-3-ketobutyrate coenzyme A ligase, mitochond	0.032	1.94	3.146067	1	1	1	445	47.9	7.97		0.11
Medium	Q9Y5K6	' CD2AP	CD2-associated protein OS=Homo sapiens OX=9606 GN=CD2AP PE=	0.032	1.939	1.564945	1	1	1	639	71.4	6.4		0.054
Medium	P98160	' HSPG2	Basement membrane-specific heparan sulfate proteoglycan core pr	0.032	1.939	0.318834	1	1	1	4391	468.5	6.51		0.011
Medium	Q15633	' TARBP2	RISC-loading complex subunit TARBP2 OS=Homo sapiens OX=9606	0.033	1.936	3.278689	1	1	1	366	39	6.54		0.136
Medium	Q5SRD1	' TIMM23B	Putative mitochondrial import inner membrane translocase subunit	0.033	1.936	6.614786	1	2	1	257	28	9.39		0.194
Medium	Q5VZ89-7	' DENND4C	Isoform 2 of DENN domain-containing protein 4C OS=Homo sapiens	0.033	1.936	0.561798	1	1	1	1958	217.7	6.64		0.022
Medium	Q8NBI5-2	' SLC43A3	Isoform 2 of Solute carrier family 43 member 3 OS=Homo sapiens O	0.033	1.924	1.388889	1	1	1	504	55.8	8.47		0.129
Medium	O43148-2	' RNMT	Isoform 2 of mRNA cap guanine-N7 methyltransferase OS=Homo sa	0.033	1.922	1.587302	1	1	1	504	57.7	7.69		0.07
Medium	Q96CB8	' INTS12	Integrator complex subunit 12 OS=Homo sapiens OX=9606 GN=INTS	0.033	1.922	3.030303	1	1	1	462	48.8	9.69		0.096
Medium	Q9C0F1-2	' CEP44	Isoform 2 of Centrosomal protein of 44 kDa OS=Homo sapiens OX=9	0.033	1.92	6.265664	1	1	1	399	44.7	5.24		0.105
Medium	O14828	' SCAMP3	Secretory carrier-associated membrane protein 3 OS=Homo sapiens	0.033	1.918	4.610951	1	1	1	347	38.3	7.64		0.166
Medium	Q9NZT1	' CALML5	Calmodulin-like protein 5 OS=Homo sapiens OX=9606 GN=CALML5	0.033	1.913	5.479452	1	2	1	146	15.9	4.44		0.292
Medium	P53355-3	' DAPK1	Isoform 3 of Death-associated protein kinase 1 OS=Homo sapiens O	0.033	1.913	1.111111	1	1	1	1440	161.1	6.87		0.028
Medium	Q86YT6	' MIB1	E3 ubiquitin-protein ligase MIB1 OS=Homo sapiens OX=9606 GN=M	0.034	1.91	0.994036	1	1	1	1006	110.1	6.92		0.04
Medium	O94826	' TOMM70	Mitochondrial import receptor subunit TOM70 OS=Homo sapiens O	0.035	1.907	1.480263	1	1	1	608	67.4	7.12		0.064
Medium	Q9NWW5	' CLN6	Isoform 2 of Ceroid-lipofuscinosis neuronal protein 6 OS=Homo sapi	0.035	1.907	3.206997	1	1	1	343	39.5	9.52		0.259
Medium	Q14554	' PDIA5	Protein disulfide-isomerase A5 OS=Homo sapiens OX=9606 GN=PDIA	0.035	1.905	1.926782	1	1	1	519	59.6	7.91		0.075
Medium	Q8NDA2	' HMCN2	Hemicentin-2 OS=Homo sapiens OX=9606 GN=HMCN2 PE=2 SV=3	0.035	1.9	0.138367	1	1	1	5059	541.6	5.87		0.009
Medium	O14732	' IMPA2	Inositol monophosphatase 2 OS=Homo sapiens OX=9606 GN=IMPA2	0.035	1.899	3.472222	1	1	1	288	31.3	6.61		0.145
Medium	Q9UK59	' DBR1	Lariat debranching enzyme OS=Homo sapiens OX=9606 GN=DBR1 P	0.035	1.897	2.022059	1	1	1	544	61.5	5.47		0.086
Medium	P37235	' HPCAL1	Hippocalcin-like protein 1 OS=Homo sapiens OX=9606 GN=HPCAL1	0.035	1.897	6.735751	1	2	1	193	22.3	5.35		0.166
Medium	Q9Y6K0	' CEPT1	Choline/ethanolaminephosphotransferase 1 OS=Homo sapiens OX=	0.035	1.893	2.644231	1	4	1	416	46.5	8.21		0.194
Medium	Q16851	' UGP2	UTP--glucose-1-phosphate uridylyltransferase OS=Homo sapiens OX	0.037	1.89	1.968504	1	1	1	508	56.9	8.15		0.075
Medium	Q9NYK5-2	' MRPL39	Isoform 2 of 39S ribosomal protein L39, mitochondrial OS=Homo sa	0.037	1.888	3.399433	1	1	1	353	40.4	7.91		0.105
Medium	Q86X83	' COMMD2	COMM domain-containing protein 2 OS=Homo sapiens OX=9606 GN	0.037	1.882	3.517588	1	1	1	199	22.7	6.73		0.212
Medium	Q9NQX1-	' PRDM5	Isoform 3 of PR domain zinc finger protein 5 OS=Homo sapiens OX=	0.037	1.882	7.207207	1	6	1	111	12.9	9.04		0.259
Medium	A8MWX3	' WASH4P	Putative WAS protein family homolog 4 OS=Homo sapiens OX=9606	0.037	1.88	1.886792	1	2	1	477	51.6	6.67		0.101
Medium	P15104	' GLUL	Glutamine synthetase OS=Homo sapiens OX=9606 GN=GLUL PE=1 S	0.038	1.879	5.36193	1	1	1	373	42	6.89		0.122
Medium	Q9NPJ3	' ACOT13	Acyl-coenzyme A thioesterase 13 OS=Homo sapiens OX=9606 GN=A	0.038	1.875	7.142857	1	1	1	140	15	9.14		0.334
Medium	Q92917	' GPKOW	G-patch domain and KOW motifs-containing protein OS=Homo sapi	0.038	1.868	6.932773	1	2	1	476	52.2	6.15		0.075
Medium	P02774-3	' GC	Isoform 3 of Vitamin D-binding protein OS=Homo sapiens OX=9606	0.038	1.868	4.462475	1	1	1	493	55.1	5.74		0.08
Medium	A0A0B4J2	' GATD3B	Glutamine amidotransferase-like class 1 domain-containing protein	0.038	1.867	9.328358	1	1	1	268	28.1	8.27		0.166
Medium	P62341	' SELENOT	Thioredoxin reductase-like selenoprotein T OS=Homo sapiens OX=9	0.038	1.865	5.128205	1	1	1	195	22.3	8.6		0.212
Medium	P53384	' NUBP1	Cytosolic Fe-S cluster assembly factor NUBP1 OS=Homo sapiens OX	0.038	1.861	2.8125	1	1	1	320	34.5	5.33		0.166
Medium	O75616	' ERAL1	GTPase Era, mitochondrial OS=Homo sapiens OX=9606 GN=ERAL1 P	0.038	1.859	3.661327	1	1	1	437	48.3	8.84		0.089
Medium	Q96CX2	' KCTD12	BTB/POZ domain-containing protein KCTD12 OS=Homo sapiens OX=	0.038	1.856	3.692308	1	1	1	325	35.7	5.64		0.122
Medium	P01764	' IGHV3-23	Immunoglobulin heavy variable 3-23 OS=Homo sapiens OX=9606 GN	0.038	1.852	9.401709	1	1	1	117	12.6	8.28		0.468
Medium	O75352	' MPDU1	Mannose-P-dolichol utilization defect 1 protein OS=Homo sapiens O	0.038	1.852	4.048583	1	1	1	247	26.6	8.94		0.334
Medium	Q9H6R3	' ACSS3	Acyl-CoA synthetase short-chain family member 3, mitochondrial O	0.038	1.848	1.895044	1	1	1	686	74.7	8.63		0.053
Medium	Q96RQ1	' ERGIC2	Endoplasmic reticulum-Golgi intermediate compartment protein 2 (	0.038	1.847	3.448276	1	1	1	377	42.5	6.77		0.11
Medium	Q9H7X3	' ZNF696	Zinc finger protein 696 OS=Homo sapiens OX=9606 GN=ZNF696 PE=	0.039	1.843	6.684492	1	1	1	374	40.5	8.92		0.093
Medium	P01714	' IGLV3-19	Immunoglobulin lambda variable 3-19 OS=Homo sapiens OX=9606 (	0.039	1.835	8.035714	1	1	1	112	12	4.96		0.585
Medium	Q14BN4	' SLMAP	Sarcolemmal membrane-associated protein OS=Homo sapiens OX=9	0.039	1.835	1.207729	1	1	1	828	95.1	5.47		0.039
Medium	A7KAX9	' ARHGAP32	Rho GTPase-activating protein 32 OS=Homo sapiens OX=9606 GN=A	0.039	1.834	0.479157	1	1	1	2087	230.4	6.74		0.021
Medium	Q9Y388	' RBMX2	RNA-binding motif protein, X-linked 2 OS=Homo sapiens OX=9606 G	0.039	1.834	2.484472	1	3	1	322	37.3	9.83		0.166
Medium	P23368	' ME2	NAD-dependent malic enzyme, mitochondrial OS=Homo sapiens OX	0.039	1.83	1.369863	1	1	1	584	65.4	7.61		0.072
Medium	P58557	' YBEY	Endoribonuclease YbeY OS=Homo sapiens OX=9606 GN=YBEY PE=1	0.039	1.828	9.580838	1	2	1	167	19.3	7.55		0.259
Medium	Q8TAK6	' OLIG1	Oligodendrocyte transcription factor 1 OS=Homo sapiens OX=9606	0.039	1.828	2.95203	1	1	1	271	27.9	9.66		0.194
Medium	Q9NP78	' ABCB9	ATP-binding cassette sub-family B member 9 OS=Homo sapiens OX=	0.04	1.822	0.913838	1	1	1	766	84.4	7.93		0.068
Medium	P49914	' MTHFS	5-formyltetrahydrofolate cyclo-ligase OS=Homo sapiens OX=9606 G	0.04	1.821	4.926108	1	1	1	203	23.2	7.88		0.194
Medium	O43681	' ASNA1	ATPase ASNA1 OS=Homo sapiens OX=9606 GN=ASNA1 PE=1 SV=2	0.04	1.821	2.873563	1	1	1	348	38.8	4.91		0.145
Medium	Q8N6H7	' ARFGAP2	ADP-ribosylation factor GTPase-activating protein 2 OS=Homo sapie	0.041	1.819	2.879079	1	1	1	521	56.7	7.99		0.072
Medium	Q9BZE7	' C22orf23	UPF0193 protein EVG1 OS=Homo sapiens OX=9606 GN=C22orf23 P	0.041	1.816	3.225806	1	4	1	217	24.9	9.8		0.194
Medium	P19174-2	' PLCG1	Isoform 2 of 1-phosphatidylinositol 4,5-bisphosphate phosphodieste	0.041	1.816	0.542215	1	4	1	1291	148.6	6.05		0.033
Medium	P62487	' POLR2G	DNA-directed RNA polymerase II subunit RPB7 OS=Homo sapiens O	0.041	1.814	7.55814	1	1	1	172	19.3	5.54		0.233
Medium	Q9UKV5	' AMFR	E3 ubiquitin-protein ligase AMFR OS=Homo sapiens OX=9606 GN=A	0.042	1.809	2.021773	1	1	1	643	72.9	6.39		0.086
Medium	Q9Y2G8	' DNAJC16	DnaJ homolog subfamily C member 16 OS=Homo sapiens OX=9606	0.042	1.808	1.023018	1	1	1	782	90.5	7.12		0.046
Medium	Q5BKT4	' ALG10	Dol-P-Glc:Glc(2)Man(9)GlcNAc(2)-PP-Dol alpha-1,2-glucosyltransfera	0.042	1.808	3.805497	1	1	1	473	55.6	9.29		0.11
Medium	Q15054	' POLD3	DNA polymerase delta subunit 3 OS=Homo sapiens OX=9606 GN=P	0.042	1.804	2.7897	1	1	1	466	51.4	9.35		0.086
Medium	Q14232	' EIF2B1	Translation initiation factor eIF-2B subunit alpha OS=Homo sapiens	0.042	1.799	3.278689	1	1	1	305	33.7	7.33		0.136
Medium	F5H4B4	' FAM227A	Protein FAM227A OS=Homo sapiens OX=9606 GN=FAM227A PE=1 S	0.042	1.796	1.22807	1	2	1	570	66.1	9.31		0.068
Medium	Q7Z5L9	' IRF2BP2	Interferon regulatory factor 2-binding protein 2 OS=Homo sapiens O	0.042	1.79	3.407155	1	1	1	587	61	8.69		0.072
Medium	Q9BRK3-2	' MXRA8	Isoform 2 of Matrix remodeling-associated protein 8 OS=Homo sapi	0.042	1.79	4.444444	1	1	1	450	49.6	6.92		0.083
Medium	Q9UL03	' INTS6	Integrator complex subunit 6 OS=Homo sapiens OX=9606 GN=INTS6	0.042	1.789	1.916573	1	1	1	887	100.3	8.62		0.044
Medium	Q08AE8	' SPIRE1	Protein spire homolog 1 OS=Homo sapiens OX=9606 GN=SPIRE1 PE=	0.042	1.788	1.322751	1	19	1	756	85.5	8.62		0.054
Medium	Q8WWL2	' SPIRE2	Protein spire homolog 2 OS=Homo sapiens OX=9606 GN=SPIRE2 PE=	0.042	1.788	1.40056	1	19	1	714	79.6	7.62		0.059
Medium	Q8TEW0	' PARD3	Partitioning defective 3 homolog OS=Homo sapiens OX=9606 GN=PA	0.042	1.785	0.737463	1	1	1	1356	151.3	7.68		0.028
Medium	A0A0B4J1	' IGLV9-49	Immunoglobulin lambda variable 9-49 OS=Homo sapiens OX=9606 (	0.042	1.785	8.130081	1	1	1	123	13	7.28		0.585
Medium	P53567	' CEBPG	CCAAT/enhancer-binding protein gamma OS=Homo sapiens OX=960	0.042	1.78	20.66667	1	2	1	150	16.4	9.77		0.334
Medium	P01704	' IGLV2-14	Immunoglobulin lambda variable 2-14 OS=Homo sapiens OX=9606 (	0.042	1.779	13.33333	1	2	1	120	12.6	6.49		2.162
Medium	Q9NZ32	' ACTR10	Actin-related protein 10 OS=Homo sapiens OX=9606 GN=ACTR10 PE	0.042	1.777	1.678657	1	1	1	417	46.3	7.37		0.093
Medium	Q9Y5T5	' USP16	Ubiquitin carboxyl-terminal hydrolase 16 OS=Homo sapiens OX=960	0.042	1.772	1.215067	1	1	1	823	93.5	6.93		0.047
Medium	O95336	' PGLS	6-phosphogluconolactonase OS=Homo sapiens OX=9606 GN=PGLS	0.042	1.772	6.20155	1	1	1	258	27.5	6.05		0.166
Medium	Q8N357	' SLC35F6	Solute carrier family 35 member F6 OS=Homo sapiens OX=9606 GN	0.042	1.771	3.773585	1	1	1	371	40.2	6.93		0.155
Medium	Q9C002	' NMES1	Normal mucosa of esophagus-specific gene 1 protein OS=Homo sap	0.043	1.761	8.433735	1	1	1	83	9.6	9.47		0.585
Medium	Q15124	' PGM5	Phosphoglucomutase-like protein 5 OS=Homo sapiens OX=9606 GN	0.044	1.757	2.645503	1	1	1	567	62.2	7.21		0.068
Medium	Q9BX66-1	' SORBS1	Isoform 12 of Sorbin and SH3 domain-containing protein 1 OS=Hom	0.044	1.756	0.927357	1	1	1	1294	143.7	8.6	Phospho (	0.027
Medium	P54819	' AK2	Adenylate kinase 2, mitochondrial OS=Homo sapiens OX=9606 GN=	0.044	1.755	6.694561	1	1	1	239	26.5	7.81		0.145
Medium	O95935	' TBX18	T-box transcription factor TBX18 OS=Homo sapiens OX=9606 GN=TE	0.044	1.749	1.153213	1	4	1	607	64.7	8.78		0.08
Medium	O75417	' POLQ	DNA polymerase theta OS=Homo sapiens OX=9606 GN=POLQ PE=1	0.045	1.743	0.772201	1	1	1	2590	289.4	7.36		0.015
Medium	P04179	' SOD2	Superoxide dismutase [Mn], mitochondrial OS=Homo sapiens OX=9	0.045	1.743	6.306306	1	1	1	222	24.7	8.25		0.194
Medium	P27658	' COL8A1	Collagen alpha-1(VIII) chain OS=Homo sapiens OX=9606 GN=COL8A	0.045	1.742	1.075269	1	4	1	744	73.3	9.61		0.077
Medium	Q03111	' MLLT1	Protein ENL OS=Homo sapiens OX=9606 GN=MLLT1 PE=1 SV=2	0.045	1.741	1.9678	1	1	1	559	62	8.59		0.093
Medium	Q13617-2	' CUL2	Isoform 2 of Cullin-2 OS=Homo sapiens OX=9606 GN=CUL2	0.045	1.741	1.308901	1	1	1	764	89.4	7.11		0.046
Medium	Q9BYF1	' ACE2	Angiotensin-converting enzyme 2 OS=Homo sapiens OX=9606 GN=A	0.045	1.74	3.850932	1	1	1	805	92.4	5.54		0.061
Medium	Q96CW5	' TUBGCP3	Gamma-tubulin complex component 3 OS=Homo sapiens OX=9606	0.045	1.738	2.425579	1	1	1	907	103.5	8.12		0.044
Medium	Q9UIA0	' CYTH4	Cytohesin-4 OS=Homo sapiens OX=9606 GN=CYTH4 PE=1 SV=1	0.045	1.733	2.030457	1	2	1	394	45.6	6.47		0.089
Medium	Q9UBV8	' PEF1	Peflin OS=Homo sapiens OX=9606 GN=PEF1 PE=1 SV=1	0.045	1.732	3.521127	1	1	1	284	30.4	6.54		0.259
Medium	O75362	' ZNF217	Zinc finger protein 217 OS=Homo sapiens OX=9606 GN=ZNF217 PE=	0.045	1.731	1.717557	1	1	1	1048	115.2	8.48		0.036
Medium	Q9NZT2	' OGFR	Opioid growth factor receptor OS=Homo sapiens OX=9606 GN=OGF	0.045	1.728	2.511078	1	1	1	677	73.3	4.84		0.072
Medium	Q86U37	' LINC01551	Uncharacterized protein encoded by LINC01551 OS=Homo sapiens (	0.045	1.728	11.97605	1	1	1	167	18.8	9.7		0.212
Medium	A0A0J9YX	' IGHV3-64D	Immunoglobulin heavy variable 3-64D OS=Homo sapiens OX=9606 (	0.045	1.727	9.401709	1	1	1	117	12.8	7.85		0.468
Medium	O94964-2	' SOGA1	Isoform 2 of Protein SOGA1 OS=Homo sapiens OX=9606 GN=SOGA1	0.045	1.726	0.662252	1	1	1	1661	183.7	6.52		0.02
Medium	Q08379	' GOLGA2	Golgin subfamily A member 2 OS=Homo sapiens OX=9606 GN=GOL	0.045	1.722	1.197605	1	2	1	1002	113	5.02		0.038
Medium	Q5U623-2	' ATF7IP2	Isoform 2 of Activating transcription factor 7-interacting protein 2 O	0.046	1.714	1.841621	1	1	1	543	60.9	8.28	Phospho (	0.064
Medium	O43300	' LRRTM2	Leucine-rich repeat transmembrane neuronal protein 2 OS=Homo s	0.046	1.714	4.457364	1	1	1	516	59	8	Phospho (	0.07
Medium	P05121	' SERPINE1	Plasminogen activator inhibitor 1 OS=Homo sapiens OX=9606 GN=S	0.046	1.712	4.975124	1	1	1	402	45	7.2		0.105
Medium	Q9Y5B8	' NME7	Nucleoside diphosphate kinase 7 OS=Homo sapiens OX=9606 GN=N	0.046	1.706	2.393617	1	1	1	376	42.5	6.47		0.096
Medium	Q9NX40	' OCIAD1	OCIA domain-containing protein 1 OS=Homo sapiens OX=9606 GN=	0.046	1.703	3.265306	1	1	1	245	27.6	7.49		0.212
Medium	Q8TD30	' GPT2	Alanine aminotransferase 2 OS=Homo sapiens OX=9606 GN=GPT2 P	0.046	1.7	4.397706	1	1	1	523	57.9	7.71		0.075
Medium	Q9HBA9	' FOLH1B	Putative N-acetylated-alpha-linked acidic dipeptidase OS=Homo sap	0.046	1.699	1.58371	1	7	1	442	50	7.02		0.068
Medium	P86790	' CCZ1B	Vacuolar fusion protein CCZ1 homolog B OS=Homo sapiens OX=960	0.046	1.697	2.904564	1	1	1	482	55.8	6.48		0.068
Medium	P52746	' ZNF142	Zinc finger protein 142 OS=Homo sapiens OX=9606 GN=ZNF142 PE=	0.046	1.689	1.244813	1	2	1	1687	187.8	7.91		0.027
Medium	Q8IYA6	' CKAP2L	Cytoskeleton-associated protein 2-like OS=Homo sapiens OX=9606 (	0.046	1.686	1.879195	1	1	1	745	83.5	9.83		0.049
Medium	Q8NF91	' SYNE1	Nesprin-1 OS=Homo sapiens OX=9606 GN=SYNE1 PE=1 SV=4	0.046	1.683	0.125043	1	6	1	8797	1010.5	5.53		0.004
Medium	Q86TJ2	' TADA2B	Transcriptional adapter 2-beta OS=Homo sapiens OX=9606 GN=TAD	0.046	1.682	1.666667	1	1	1	420	48.4	7.83		0.116
Medium	Q9HC52	' CBX8	Chromobox protein homolog 8 OS=Homo sapiens OX=9606 GN=CBX	0.046	1.68	2.570694	1	1	1	389	43.4	9.91		0.11
Medium	P49281-3	' SLC11A2	Isoform 3 of Natural resistance-associated macrophage protein 2 O	0.046	1.679	1.525424	1	1	1	590	64.7	7.2		0.136
Medium	Q96Q89-2	' KIF20B	Isoform 2 of Kinesin-like protein KIF20B OS=Homo sapiens OX=9606	0.046	1.678	0.755532	1	1	1	1853	214.1	5.91		0.018
Medium	Q96BZ4	' PLD4	Phospholipase D4 OS=Homo sapiens OX=9606 GN=PLD4 PE=2 SV=2	0.048	1.669	3.359684	1	1	1	506	55.6	8.35		0.093
Medium	O43617	' TRAPPC3	Trafficking protein particle complex subunit 3 OS=Homo sapiens OX	0.048	1.666	4.444444	1	3	1	180	20.3	4.96		0.292
Low	Q9NQC1	' JADE2	E3 ubiquitin-protein ligase Jade-2 OS=Homo sapiens OX=9606 GN=J	0.05	1.659	2.151899	1	1	1	790	87.4	5.26		0.055
Low	P53801	' PTTG1IP	Pituitary tumor-transforming gene 1 protein-interacting protein OS=	0.051	1.658	3.888889	1	1	1	180	20.3	8.79		0.334
Low	Q9Y312	' AAR2	Protein AAR2 homolog OS=Homo sapiens OX=9606 GN=AAR2 PE=1	0.051	1.657	3.90625	1	1	1	384	43.4	5.96		0.105
Low	Q8ND83	' SLAIN1	SLAIN motif-containing protein 1 OS=Homo sapiens OX=9606 GN=SL	0.051	1.651	5.985915	1	2	1	568	60.6	7.64		0.083
Low	Q9NWF9-	' RNF216	Isoform 2 of E3 ubiquitin-protein ligase RNF216 OS=Homo sapiens O	0.051	1.65	2.058505	1	5	1	923	105.8	5.01		0.049
Low	O75815	' BCAR3	Breast cancer anti-estrogen resistance protein 3 OS=Homo sapiens (	0.051	1.641	0.848485	1	1	1	825	92.5	7.96		0.038
Low	P14921-3	' ETS1	Isoform 3 of Protein C-ets-1 OS=Homo sapiens OX=9606 GN=ETS1	0.052	1.638	1.85567	1	1	1	485	55.4	5.15		0.122
Low	Q09MP3	' RAD51AP2	RAD51-associated protein 2 OS=Homo sapiens OX=9606 GN=RAD51	0.052	1.636	1.553063	1	1	1	1159	133.8	7.34		0.031
Low	Q9UMY4	' SNX12	Sorting nexin-12 OS=Homo sapiens OX=9606 GN=SNX12 PE=1 SV=3	0.052	1.633	5.232558	1	1	1	172	19.7	7.87		0.212
Low	Q9GZP8	' IMUP	Immortalization up-regulated protein OS=Homo sapiens OX=9606 G	0.052	1.629	9.433962	1	1	1	106	10.9	9.73		0.585
Low	O15127	' SCAMP2	Secretory carrier-associated membrane protein 2 OS=Homo sapiens	0.052	1.627	6.079027	1	1	1	329	36.6	6.1		0.292
Low	Q00653	' NFKB2	Nuclear factor NF-kappa-B p100 subunit OS=Homo sapiens OX=960	0.053	1.625	2.333333	1	1	1	900	96.7	6.25		0.05
Low	Q8WUH6	' TMEM263	Transmembrane protein 263 OS=Homo sapiens OX=9606 GN=TMEM	0.053	1.619	8.62069	1	1	1	116	11.7	9.32		0.292
Low	Q66K74	' MAP1S	Microtubule-associated protein 1S OS=Homo sapiens OX=9606 GN=	0.055	1.608	0.944287	1	1	1	1059	112.1	7.3		0.049
Low	P21953	' BCKDHB	2-oxoisovalerate dehydrogenase subunit beta, mitochondrial OS=Ho	0.055	1.607	3.316327	1	1	1	392	43.1	6.29		0.116
Low	Q8TEX9-2	' IPO4	Isoform 2 of Importin-4 OS=Homo sapiens OX=9606 GN=IPO4	0.055	1.603	1.015697	1	2	1	1083	118.8	4.94		0.041
Low	Q9UH65	' SWAP70	Switch-associated protein 70 OS=Homo sapiens OX=9606 GN=SWAP	0.055	1.6	1.196581	1	1	1	585	69	5.87		0.058
Low	O15212	' PFDN6	Prefoldin subunit 6 OS=Homo sapiens OX=9606 GN=PFDN6 PE=1 SV	0.055	1.597	9.302326	1	1	1	129	14.6	8.88		0.212
Low	Q8NAP3	' ZBTB38	Zinc finger and BTB domain-containing protein 38 OS=Homo sapien	0.055	1.597	0.669456	1	1	1	1195	134.2	8.03		0.035
Low	P13196	' ALAS1	5-aminolevulinate synthase, nonspecific, mitochondrial OS=Homo s	0.055	1.595	3.90625	1	2	1	640	70.5	8.46		0.054
Low	Q15833-3	' STXBP2	Isoform 3 of Syntaxin-binding protein 2 OS=Homo sapiens OX=9606	0.055	1.594	3.311258	1	2	1	604	67.7	6.68		0.058
Low	O60287	' URB1	Nucleolar pre-ribosomal-associated protein 1 OS=Homo sapiens OX	0.056	1.591	0.484368	1	1	1	2271	254.2	6.47		0.019
Low	Q8WXH0-	' SYNE2	Isoform 2 of Nesprin-2 OS=Homo sapiens OX=9606 GN=SYNE2	0.056	1.591	0.231649	1	1	1	6907	798.4	5.36		0.005
Low	Q04721	' NOTCH2	Neurogenic locus notch homolog protein 2 OS=Homo sapiens OX=9	0.056	1.59	0.526103	1	1	1	2471	265.2	5.14		0.024
Low	Q9Y6E2	' BZW2	Basic leucine zipper and W2 domain-containing protein 2 OS=Homo	0.056	1.59	2.147971	1	3	1	419	48.1	6.68		0.077
Low	O60271	' SPAG9	C-Jun-amino-terminal kinase-interacting protein 4 OS=Homo sapien	0.056	1.589	0.681302	1	1	1	1321	146.1	5.15		0.03
Low	P21359	' NF1	Neurofibromin OS=Homo sapiens OX=9606 GN=NF1 PE=1 SV=2	0.056	1.588	0.457908	1	1	1	2839	319.2	7.39		0.014
Low	Q96CN5	' LRRC45	Leucine-rich repeat-containing protein 45 OS=Homo sapiens OX=96	0.056	1.585	1.044776	1	1	1	670	75.9	6.23		0.045
Low	Q9BWU0	' SLC4A1AP	Kanadaptin OS=Homo sapiens OX=9606 GN=SLC4A1AP PE=1 SV=1	0.056	1.583	1.130653	1	1	1	796	88.8	5.19		0.047
Low	Q01668-2	' CACNA1D	Isoform Beta-cell-type of Voltage-dependent L-type calcium channe	0.057	1.58	1.650619	1	1	1	2181	247.4	6.98	Phospho (	0.023
Low	P00414	' MT-CO3	Cytochrome c oxidase subunit 3 OS=Homo sapiens OX=9606 GN=MT	0.057	1.569	5.363985	1	1	1	261	29.9	7.31		1.154
Low	Q13111	' CHAF1A	Chromatin assembly factor 1 subunit A OS=Homo sapiens OX=9606	0.057	1.569	0.732218	1	1	1	956	106.8	5.94		0.05
Low	Q9H9C1	' VIPAS39	Spermatogenesis-defective protein 39 homolog OS=Homo sapiens (	0.057	1.569	3.042596	1	1	1	493	57	7.4		0.075
Low	Q7Z4H9	' FAM220A	Protein FAM220A OS=Homo sapiens OX=9606 GN=FAM220A PE=2 S	0.057	1.567	10.42471	1	4	1	259	28	8.56		0.194
Low	Q8IUH4	' ZDHHC13	Palmitoyltransferase ZDHHC13 OS=Homo sapiens OX=9606 GN=ZDH	0.057	1.566	3.376206	1	1	1	622	70.8	8.07		0.086
Low	O14633	' LCE2B	Late cornified envelope protein 2B OS=Homo sapiens OX=9606 GN=	0.057	1.565	22.72727	1	1	1	110	11.2	8.09		1.154
Low	Q92556	' ELMO1	Engulfment and cell motility protein 1 OS=Homo sapiens OX=9606 (	0.057	1.565	1.100413	1	1	1	727	83.8	6.28		0.05
Low	Q9H1Y0	' ATG5	Autophagy protein 5 OS=Homo sapiens OX=9606 GN=ATG5 PE=1 SV	0.058	1.563	4.727273	1	1	1	275	32.4	5.77		0.145
Low	P33947	' KDELR2	ER lumen protein-retaining receptor 2 OS=Homo sapiens OX=9606 (	0.058	1.562	8.018868	1	1	1	212	24.4	8.72		0.292
Low	Q9BYD1	' MRPL13	39S ribosomal protein L13, mitochondrial OS=Homo sapiens OX=96	0.058	1.561	5.617978	1	2	1	178	20.7	9.16		0.166
Low	Q5VVP1	' SPATA31A6	Spermatogenesis-associated protein 31A6 OS=Homo sapiens OX=96	0.058	1.561	1.265823	1	1	1	1343	147.7	8.7	Phospho (	0.033
Low	Q16836-2	' HADH	Isoform 2 of Hydroxyacyl-coenzyme A dehydrogenase, mitochondri	0.059	1.559	2.307692	1	1	1	390	42.1	9.26		0.101
Low	Q9HBE1	' PATZ1	POZ-, AT hook-, and zinc finger-containing protein 1 OS=Homo sapie	0.059	1.558	2.328967	1	1	1	687	74	8.35		0.064
Low	Q96CB9	' NSUN4	5-methylcytosine rRNA methyltransferase NSUN4 OS=Homo sapiens	0.059	1.557	2.083333	1	1	1	384	43.1	8.18		0.129
Low	O60645	' EXOC3	Exocyst complex component 3 OS=Homo sapiens OX=9606 GN=EXO	0.059	1.557	0.925926	1	1	1	756	86.8	6.11		0.054
Low	O14745	' SLC9A3R1	Na(+)/H(+) exchange regulatory cofactor NHE-RF1 OS=Homo sapien	0.059	1.556	4.748603	1	1	1	358	38.8	5.77		0.089
Low	O94762	' RECQL5	ATP-dependent DNA helicase Q5 OS=Homo sapiens OX=9606 GN=R	0.059	1.553	1.009082	1	1	1	991	108.8	8.56		0.036
Low	P63279	' UBE2I	SUMO-conjugating enzyme UBC9 OS=Homo sapiens OX=9606 GN=U	0.059	1.552	6.329114	1	1	1	158	18	8.66		0.334
Low	Q155Q3	' DIXDC1	Dixin OS=Homo sapiens OX=9606 GN=DIXDC1 PE=1 SV=2	0.059	1.552	1.317716	1	1	1	683	77.4	6.24		0.054
Low	Q9H1E1	' RNASE7	Ribonuclease 7 OS=Homo sapiens OX=9606 GN=RNASE7 PE=1 SV=2	0.059	1.552	8.974359	1	1	1	156	17.4	9.69		0.389
Low	O60285	' NUAK1	NUAK family SNF1-like kinase 1 OS=Homo sapiens OX=9606 GN=NU	0.059	1.552	1.966717	1	1	1	661	74.3	8.82		0.066
Low	Q9H1A4	' ANAPC1	Anaphase-promoting complex subunit 1 OS=Homo sapiens OX=960	0.06	1.539	1.131687	1	1	1	1944	216.4	6.3		0.022
Low	O60393	' NOBOX	Homeobox protein NOBOX OS=Homo sapiens OX=9606 GN=NOBOX	0.063	1.532	2.894356	1	2	1	691	73.9	6.13	Phospho (	0.096
Low	O95298	' NDUFC2	NADH dehydrogenase [ubiquinone] 1 subunit C2 OS=Homo sapiens	0.063	1.531	5.882353	1	1	1	119	14.2	8.98		0.259
Low	Q13564-4	' NAE1	Isoform 4 of NEDD8-activating enzyme E1 regulatory subunit OS=Ho	0.063	1.524	2.793296	1	3	1	537	60.5	5.4		0.083
Low	Q9NS86	' LANCL2	LanC-like protein 2 OS=Homo sapiens OX=9606 GN=LANCL2 PE=1 SV	0.063	1.523	3.111111	1	2	1	450	50.8	7.43		0.101
Low	A8K0R7-5	' ZNF839	Isoform 3 of Zinc finger protein 839 OS=Homo sapiens OX=9606 GN	0.063	1.52	1.941748	1	1	1	927	99.4	6.61	Phospho (	0.047
Low	Q9H7Z7	' PTGES2	Prostaglandin E synthase 2 OS=Homo sapiens OX=9606 GN=PTGES2	0.063	1.52	2.387268	1	1	1	377	41.9	9.16		0.093
Low	Q9BYR9	' KRTAP2-4	Keratin-associated protein 2-4 OS=Homo sapiens OX=9606 GN=KRT	0.063	1.514	17.96875	1	1	1	128	13.5	7.87		1.154
Low	Q9Y3B2	' EXOSC1	Exosome complex component CSL4 OS=Homo sapiens OX=9606 GN	0.063	1.512	5.641026	1	2	1	195	21.4	8.24		0.233
Low	P01024	' C3	Complement C3 OS=Homo sapiens OX=9606 GN=C3 PE=1 SV=2	0.064	1.506	0.661455	1	1	1	1663	187	6.4		0.021
Low	Q16650	' TBR1	T-box brain protein 1 OS=Homo sapiens OX=9606 GN=TBR1 PE=1 SV	0.065	1.504	1.759531	1	5	1	682	74	7.33		0.066
Low	Q92545	' TMEM131	Transmembrane protein 131 OS=Homo sapiens OX=9606 GN=TMEM	0.069	1.49	1.009028	1	1	1	1883	205	8.59		0.027
Low	P35398-1	' RORA	Isoform 2 of Nuclear receptor ROR-alpha OS=Homo sapiens OX=960	0.07	1.487	1.079137	1	3	1	556	63	6.38		0.083
Low	A0A075B	' IGLV2-18	Immunoglobulin lambda variable 2-18 OS=Homo sapiens OX=9606 (	0.071	1.481	13.55932	1	1	1	118	12.4	4.82		1.154
Low	Q96I51	' RCC1L	RCC1-like G exchanging factor-like protein OS=Homo sapiens OX=96	0.071	1.479	4.741379	1	1	1	464	50	8.4		0.089
Low	Q8IYT8	' ULK2	Serine/threonine-protein kinase ULK2 OS=Homo sapiens OX=9606 G	0.071	1.477	3.571429	1	2	1	1036	112.6	8.53	Phospho (	0.044
Low	Q13042	' CDC16	Cell division cycle protein 16 homolog OS=Homo sapiens OX=9606 (	0.071	1.476	2.903226	1	1	1	620	71.6	5.85		0.064
Low	Q96F85	' CNRIP1	CB1 cannabinoid receptor-interacting protein 1 OS=Homo sapiens C	0.071	1.473	6.707317	1	1	1	164	18.6	7.94		0.233
Low	Q13423	' NNT	NAD(P) transhydrogenase, mitochondrial OS=Homo sapiens OX=960	0.071	1.471	1.381215	1	1	1	1086	113.8	8.09		0.045
Low	O43826-2	' SLC37A4	Isoform 2 of Glucose-6-phosphate exchanger SLC37A4 OS=Homo sa	0.071	1.466	7.760532	1	2	1	451	48.8	7.93	Phospho (	0.155
Low	Q13574	' DGKZ	Diacylglycerol kinase zeta OS=Homo sapiens OX=9606 GN=DGKZ PE	0.071	1.465	1.342883	1	1	1	1117	124.1	9.04		0.037
Low	Q13107	' USP4	Ubiquitin carboxyl-terminal hydrolase 4 OS=Homo sapiens OX=9606	0.071	1.464	2.38837	1	1	1	963	108.5	5.71		0.042
Low	Q12888-2	' TP53BP1	Isoform 2 of TP53-binding protein 1 OS=Homo sapiens OX=9606 GN	0.071	1.461	0.708144	1	1	1	1977	214	4.7		0.022
Low	Q6PEW0	' PRSS54	Inactive serine protease 54 OS=Homo sapiens OX=9606 GN=PRSS54	0.073	1.449	3.544304	1	1	1	395	43.8	6.7		0.11
Low	O75376	' NCOR1	Nuclear receptor corepressor 1 OS=Homo sapiens OX=9606 GN=NC	0.073	1.448	0.901639	1	1	1	2440	270	7.11		0.017
Low	P24311	' COX7B	Cytochrome c oxidase subunit 7B, mitochondrial OS=Homo sapiens	0.073	1.444	8.75	1	1	1	80	9.2	10.27		0.778
Low	Q92538	' GBF1	Golgi-specific brefeldin A-resistance guanine nucleotide exchange fa	0.074	1.442	0.968263	1	2	1	1859	206.3	5.73		0.021
Low	Q13445	' TMED1	Transmembrane emp24 domain-containing protein 1 OS=Homo sap	0.074	1.436	5.726872	1	1	1	227	25.2	4.48		0.292
Low	Q06124	' PTPN11	Tyrosine-protein phosphatase non-receptor type 11 OS=Homo sapie	0.074	1.435	1.842546	1	1	1	597	68.4	7.3		0.056
Low	Q96BT7-4	' ALKBH8	Isoform 4 of Alkylated DNA repair protein alkB homolog 8 OS=Homo	0.074	1.43	1.1994	1	2	1	667	75.5	7.99		0.055
Low	Q7Z7H8-2	' MRPL10	Isoform 2 of 39S ribosomal protein L10, mitochondrial OS=Homo sa	0.074	1.429	7.01107	1	2	1	271	30.3	9.25		0.212
Low	Q6IAA8	' LAMTOR1	Ragulator complex protein LAMTOR1 OS=Homo sapiens OX=9606 G	0.075	1.425	8.074534	1	2	1	161	17.7	5.15		0.292
Low	P27986-4	' PIK3R1	Isoform 4 of Phosphatidylinositol 3-kinase regulatory subunit alpha	0.075	1.424	4.234973	1	3	1	732	84.4	6.16		0.051
Low	Q9NXR7-	' BABAM2	Isoform 1 of BRISC and BRCA1-A complex member 2 OS=Homo sapi	0.076	1.423	3.373494	1	1	1	415	46.9	6.14		0.101
Low	Q14642	' INPP5A	Type I inositol 1,4,5-trisphosphate 5-phosphatase OS=Homo sapiens	0.076	1.42	2.184466	1	1	1	412	47.8	7.03		0.089
Low	A0A075B	' IGLV3-10	Immunoglobulin lambda variable 3-10 OS=Homo sapiens OX=9606 (	0.076	1.419	6.956522	1	1	1	115	12.4	4.83		0.468
Low	Q9UHD2	' TBK1	Serine/threonine-protein kinase TBK1 OS=Homo sapiens OX=9606 G	0.076	1.418	1.783265	1	1	1	729	83.6	6.79		0.051
Low	Q9BPX3	' NCAPG	Condensin complex subunit 3 OS=Homo sapiens OX=9606 GN=NCAP	0.076	1.416	1.576355	1	1	1	1015	114.3	5.59		0.042
Low	A0A0J9YX	' IGHV5-10-1	Immunoglobulin heavy variable 5-10-1 OS=Homo sapiens OX=9606 (	0.077	1.411	5.982906	1	1	1	117	12.8	8.28		0.389
Low	O60942	' RNGTT	mRNA-capping enzyme OS=Homo sapiens OX=9606 GN=RNGTT PE=	0.077	1.408	1.675042	1	1	1	597	68.5	8.13		0.061
Low	O43181	' NDUFS4	NADH dehydrogenase [ubiquinone] iron-sulfur protein 4, mitochond	0.079	1.404	8.571429	1	1	1	175	20.1	10.3		0.233
Low	Q14517	' FAT1	Protocadherin Fat 1 OS=Homo sapiens OX=9606 GN=FAT1 PE=1 SV=	0.079	1.403	0.457716	1	1	1	4588	506	5		0.011
Low	Q8N4Q0	' ZADH2	Prostaglandin reductase 3 OS=Homo sapiens OX=9606 GN=ZADH2 P	0.079	1.401	2.387268	1	1	1	377	40.1	8.18		0.116
Low	Q9NYU2	' UGGT1	UDP-glucose:glycoprotein glucosyltransferase 1 OS=Homo sapiens (	0.079	1.399	0.514469	1	1	1	1555	177.1	5.63		0.027
Low	Q1MSJ5	' CSPP1	Centrosome and spindle pole-associated protein 1 OS=Homo sapien	0.079	1.398	1.11465	1	1	1	1256	145.4	6.8		0.033
Low	P57737-3	' CORO7	Isoform 3 of Coronin-7 OS=Homo sapiens OX=9606 GN=CORO7	0.079	1.395	1.240458	1	1	1	1048	114.1	6.33		0.047
Low	P15514	' AREG	Amphiregulin OS=Homo sapiens OX=9606 GN=AREG PE=1 SV=2	0.081	1.384	4.365079	1	1	1	252	27.9	7.4		0.233
Low	Q9Y566	' SHANK1	SH3 and multiple ankyrin repeat domains protein 1 OS=Homo sapie	0.081	1.382	1.619621	1	1	1	2161	224.8	8.15		0.024
Low	P01584	' IL1B	Interleukin-1 beta OS=Homo sapiens OX=9606 GN=IL1B PE=1 SV=2	0.081	1.381	4.089219	1	1	1	269	30.7	4.83		0.194
Low	Q9UIJ7	' AK3	GTP:AMP phosphotransferase AK3, mitochondrial OS=Homo sapien	0.082	1.379	4.405286	1	1	1	227	25.6	9.16		0.129
Low	Q9H9B1	' EHMT1	Histone-lysine N-methyltransferase EHMT1 OS=Homo sapiens OX=9	0.082	1.377	1.155624	1	1	1	1298	141.4	5.76		0.032
Low	Q9ULJ6	' ZMIZ1	Zinc finger MIZ domain-containing protein 1 OS=Homo sapiens OX=	0.082	1.375	0.749766	1	1	1	1067	115.4	7.46		0.08
Low	O75691	' UTP20	Small subunit processome component 20 homolog OS=Homo sapien	0.082	1.372	0.43088	1	1	1	2785	318.2	7.39		0.014
Low	Q9Y297	' BTRC	F-box/WD repeat-containing protein 1A OS=Homo sapiens OX=9606	0.082	1.372	1.983471	1	1	1	605	68.8	7.96		0.056
Low	Q9NVS2-2	' MRPS18A	Isoform 2 of 39S ribosomal protein S18a, mitochondrial OS=Homo s	0.082	1.372	3.333333	1	1	1	270	30.5	10.64		0.122
Low	Q9BXF6	' RAB11FIP5	Rab11 family-interacting protein 5 OS=Homo sapiens OX=9606 GN=	0.082	1.368	3.522205	1	1	1	653	70.4	9.23		0.064
Low	Q9NNX1	' TUFT1	Tuftelin OS=Homo sapiens OX=9606 GN=TUFT1 PE=1 SV=1	0.082	1.367	2.820513	1	1	1	390	44.2	6		0.093
Low	A2RRP1	' NBAS	Neuroblastoma-amplified sequence OS=Homo sapiens OX=9606 GN	0.082	1.367	0.716997	1	1	1	2371	268.4	5.96		0.016
Low	Q96NB2	' SFXN2	Sideroflexin-2 OS=Homo sapiens OX=9606 GN=SFXN2 PE=1 SV=2	0.082	1.367	3.726708	1	1	1	322	36.2	9.41		0.11
Low	Q6NSJ5	' LRRC8E	Volume-regulated anion channel subunit LRRC8E OS=Homo sapiens	0.083	1.36	3.015075	1	2	1	796	90.2	6.96		0.051
Low	Q08AM6-	' VAC14	Isoform 2 of Protein VAC14 homolog OS=Homo sapiens OX=9606 GN	0.083	1.354	3.271028	1	1	1	214	24.8	6.93		0.194
Low	Q96L73	' NSD1	Histone-lysine N-methyltransferase, H3 lysine-36 and H4 lysine-20 s	0.084	1.353	0.296736	1	2	1	2696	296.5	8.03		0.014
Low	Q9P031	' CCDC59	Thyroid transcription factor 1-associated protein 26 OS=Homo sapie	0.084	1.352	4.149378	1	1	1	241	28.7	9.88		0.212
Low	Q9NQ34	' TMEM9B	Transmembrane protein 9B OS=Homo sapiens OX=9606 GN=TMEM	0.084	1.351	3.535354	1	1	1	198	22.5	8.06		0.194
Low	P56270-2	' MAZ	Isoform 2 of Myc-associated zinc finger protein OS=Homo sapiens O	0.085	1.35	2.434077	1	1	1	493	51	9.1		0.105
Low	Q96JE7	' SEC16B	Protein transport protein Sec16B OS=Homo sapiens OX=9606 GN=S	0.085	1.346	2.358491	1	1	1	1060	116.5	6.09	Phospho (	0.049
Low	O75665	' OFD1	Oral-facial-digital syndrome 1 protein OS=Homo sapiens OX=9606 G	0.085	1.339	1.185771	1	1	1	1012	116.6	6.1		0.032
Low	Q13614	' MTMR2	Myotubularin-related protein 2 OS=Homo sapiens OX=9606 GN=MT	0.086	1.335	1.244168	1	1	1	643	73.3	7.4		0.066
Low	Q9H560	' ANKRD19P	Putative ankyrin repeat domain-containing protein 19 OS=Homo sa	0.086	1.333	4.545455	1	1	1	264	30.4	8.95		0.194
Low	Q499Z4	' ZNF672	Zinc finger protein 672 OS=Homo sapiens OX=9606 GN=ZNF672 PE=	0.086	1.332	3.539823	1	1	1	452	50.2	9.89		0.07
Low	P29372	' MPG	DNA-3-methyladenine glycosylase OS=Homo sapiens OX=9606 GN=	0.087	1.331	4.026846	1	1	1	298	32.8	9.57		0.145
Low	Q16864-2	' ATP6V1F	Isoform 2 of V-type proton ATPase subunit F OS=Homo sapiens OX=	0.087	1.33	6.122449	1	1	1	147	16.4	5.08		0.292
Low	Q9UIV1	' CNOT7	CCR4-NOT transcription complex subunit 7 OS=Homo sapiens OX=9	0.087	1.328	7.017544	1	1	1	285	32.7	4.84		0.166
Low	Q9H8M5	' CNNM2	Metal transporter CNNM2 OS=Homo sapiens OX=9606 GN=CNNM2	0.088	1.32	1.371429	1	2	1	875	96.6	6.38		0.047
Low	Q03181	' PPARD	Peroxisome proliferator-activated receptor delta OS=Homo sapiens	0.088	1.32	1.587302	1	3	1	441	49.9	7.59		0.096
Low	Q96T58	' SPEN	Msx2-interacting protein OS=Homo sapiens OX=9606 GN=SPEN PE=	0.088	1.319	0.382096	1	1	1	3664	402	7.64		0.011
Low	Q9P253	' VPS18	Vacuolar protein sorting-associated protein 18 homolog OS=Homo s	0.088	1.319	1.541624	1	3	1	973	110.1	6.07		0.042
Low	Q8WWN8	' ARAP3	Arf-GAP with Rho-GAP domain, ANK repeat and PH domain-containi	0.088	1.318	1.036269	1	1	1	1544	169.7	7.08		0.028
Low	O60725	' ICMT	Protein-S-isoprenylcysteine O-methyltransferase OS=Homo sapiens	0.088	1.317	3.169014	1	1	1	284	31.9	7.96		0.194
Low	Q9UMZ3	' PTPRQ	Phosphatidylinositol phosphatase PTPRQ OS=Homo sapiens OX=960	0.088	1.316	0.900515	1	1	1	2332	260.8	5.81		0.02
Low	Q6DT37	' CDC42BPG	Serine/threonine-protein kinase MRCK gamma OS=Homo sapiens O	0.089	1.309	0.70922	1	1	1	1551	172.4	6.28		0.027
Low	A6NL88	' SHISA7	Protein shisa-7 OS=Homo sapiens OX=9606 GN=SHISA7 PE=2 SV=3	0.089	1.308	6.319703	1	1	1	538	56.2	10.02		0.072
Low	Q13049	' TRIM32	E3 ubiquitin-protein ligase TRIM32 OS=Homo sapiens OX=9606 GN=	0.09	1.301	2.143951	1	2	1	653	71.9	6.98		0.058
Low	Q6NXE6	' ARMC6	Armadillo repeat-containing protein 6 OS=Homo sapiens OX=9606 (	0.09	1.299	3.193613	1	1	1	501	54.1	6.24		0.089
Low	Q9P266	' JCAD	Junctional protein associated with coronary artery disease OS=Hom	0.09	1.289	0.883002	1	1	1	1359	148.3	7.09		0.028
Low	Q2KHT4-8	' GSG1	Isoform 8 of Germ cell-specific gene 1 protein OS=Homo sapiens OX	0.09	1.288	4.956268	1	2	1	343	38.1	7.33		0.145
Low	P00749	' PLAU	Urokinase-type plasminogen activator OS=Homo sapiens OX=9606 (	0.09	1.287	2.320186	1	2	1	431	48.5	8.41		0.093
Low	Q00765	' REEP5	Receptor expression-enhancing protein 5 OS=Homo sapiens OX=960	0.091	1.283	4.761905	1	1	1	189	21.5	8.1		0.259
Low	Q8IVU3	' HERC6	Probable E3 ubiquitin-protein ligase HERC6 OS=Homo sapiens OX=9	0.091	1.28	2.641879	1	1	1	1022	115.1	7.96		0.04
Low	Q07075	' ENPEP	Glutamyl aminopeptidase OS=Homo sapiens OX=9606 GN=ENPEP P	0.092	1.276	5.015674	1	1	1	957	109.2	5.47		0.04
Low	Q8WW38	' ZFPM2	Zinc finger protein ZFPM2 OS=Homo sapiens OX=9606 GN=ZFPM2 P	0.092	1.274	1.476977	1	2	1	1151	128.1	6.47		0.033
Low	Q8NI35	' PATJ	InaD-like protein OS=Homo sapiens OX=9606 GN=PATJ PE=1 SV=3	0.092	1.273	1.054969	1	1	1	1801	196.2	4.94		0.024
Low	P19823	' ITIH2	Inter-alpha-trypsin inhibitor heavy chain H2 OS=Homo sapiens OX=9	0.094	1.263	1.585624	1	1	1	946	106.4	6.86		0.041
Low	Q8N335	' GPD1L	Glycerol-3-phosphate dehydrogenase 1-like protein OS=Homo sapie	0.094	1.262	2.279202	1	1	1	351	38.4	7.02		0.093
Low	Q9BYD3	' MRPL4	39S ribosomal protein L4, mitochondrial OS=Homo sapiens OX=960	0.094	1.255	6.109325	1	1	1	311	34.9	9.72		0.136
Low	A0A075B	' IGLV4-60	Immunoglobulin lambda variable 4-60 OS=Homo sapiens OX=9606 (	0.094	1.254	8.333333	1	1	1	120	13	6.25		0.468
Low	Q8NE62	' CHDH	Choline dehydrogenase, mitochondrial OS=Homo sapiens OX=9606	0.095	1.251	2.020202	1	1	1	594	65.3	8.28		0.058
Low	Q9Y2Q5-2	' LAMTOR2	Isoform 2 of Ragulator complex protein LAMTOR2 OS=Homo sapien	0.095	1.251	6.666667	1	1	1	150	15.6	7.77		0.389
Low	O00399	' DCTN6	Dynactin subunit 6 OS=Homo sapiens OX=9606 GN=DCTN6 PE=1 SV	0.095	1.247	5.263158	1	1	1	190	20.7	6.32		0.334
Low	P04035-3	' HMGCR	Isoform 3 of 3-hydroxy-3-methylglutaryl-coenzyme A reductase OS=	0.095	1.242	1.211454	1	1	1	908	99.7	6.77		0.049
Low	Q5BJE1	' CCDC178	Coiled-coil domain-containing protein 178 OS=Homo sapiens OX=96	0.095	1.241	1.614764	1	1	1	867	101.9	6.77		0.037
Low	Q92839	' HAS1	Hyaluronan synthase 1 OS=Homo sapiens OX=9606 GN=HAS1 PE=2	0.095	1.231	1.384083	1	2	1	578	64.8	9.19		0.062
Low	P52888	' THOP1	Thimet oligopeptidase OS=Homo sapiens OX=9606 GN=THOP1 PE=1	0.095	1.229	1.161103	1	1	1	689	78.8	6.05		0.058
Low	A0A0B4J2	' IGHV1-69D	Immunoglobulin heavy variable 1-69D OS=Homo sapiens OX=9606 (	0.096	1.225	9.401709	1	1	1	117	12.7	8.47		0.389
Low	Q9GZS3	' WDR61	WD repeat-containing protein 61 OS=Homo sapiens OX=9606 GN=W	0.096	1.225	5.245902	1	1	1	305	33.6	5.47		0.145
Low	O95169	' NDUFB8	NADH dehydrogenase [ubiquinone] 1 beta subcomplex subunit 8, m	0.096	1.223	4.83871	1	1	1	186	21.8	6.8		0.233
Low	Q14191	' WRN	Werner syndrome ATP-dependent helicase OS=Homo sapiens OX=9	0.096	1.214	0.768156	1	1	1	1432	162.4	6.34		0.027
Low	Q9NQT5	' EXOSC3	Exosome complex component RRP40 OS=Homo sapiens OX=9606 G	0.096	1.204	7.636364	1	1	1	275	29.6	8.1		0.179
Low	Q9Y619	' SLC25A15	Mitochondrial ornithine transporter 1 OS=Homo sapiens OX=9606 G	0.098	1.182	5.647841	1	1	1	301	32.7	9.13		0.129
Low	P0DOX6	' NA	Immunoglobulin mu heavy chain OS=Homo sapiens OX=9606 PE=1 S	0.098	1.172	3.819444	1	1	1	576	63.4	7.87		0.068
Low	Q96DT6	' ATG4C	Cysteine protease ATG4C OS=Homo sapiens OX=9606 GN=ATG4C PE	0.099	1.168	1.528384	1	1	1	458	52.5	5.97		0.08
Low	P38398-3	' BRCA1	Isoform 3 of Breast cancer type 1 susceptibility protein OS=Homo sa	0.099	1.161	4.479578	1	2	1	759	84.9	5.08		0.054
Low	Q5VIR6-4	' VPS53	Isoform 4 of Vacuolar protein sorting-associated protein 53 homolo	0.099	1.148	2.884615	1	2	1	832	94.3	6.7		0.048

Low	C9JPN9	' USP17L12	Ubiquitin carboxyl-terminal hydrolase 17-like protein 12 OS=Homo s	0.099	1.146	6.226415	1	1	1	530	59.6	8.07		0.08	C9JPN9	NA	NA	' USP17L1	Ubiquitin carboxyl-terminal hydrolase 17-like protein 12 OS=Homo sapiens OX=9606 GN=USP17L12 PE=3 SV=1
Low	Q5TCS8	' AK9	Adenylate kinase 9 OS=Homo sapiens OX=9606 GN=AK9 PE=1 SV=2	0.101	1.119	0.994244	1	1	1	1911	221.3	5.01		0.021	Q5TCS8	NA	NA	' AK9	Adenylate kinase 9 OS=Homo sapiens OX=9606 GN=AK9 PE=1 SV=2
Low	Q92851-4	' CASP10	Isoform D of Caspase-10 OS=Homo sapiens OX=9606 GN=CASP10	0.101	1.102	3.065134	1	1	1	522	59	6.62		0.093	Q92851-4	NA	NA	' CASP10	Isoform D of Caspase-10 OS=Homo sapiens OX=9606 GN=CASP10
Low	P04430	' IGKV1-16	Immunoglobulin kappa variable 1-16 OS=Homo sapiens OX=9606 GI	0.101	1.046	13.67521	1	1	1	117	12.6	8.16		0.468	P04430	NA	NA	' IGKV1-16	Immunoglobulin kappa variable 1-16 OS=Homo sapiens OX=9606 GN=IGKV1-16 PE=1 SV=2
Low	Q9Y4H2	' IRS2	Insulin receptor substrate 2 OS=Homo sapiens OX=9606 GN=IRS2 PE	0.101	1.03	1.046338	1	1	1	1338	137.2	8.65		0.038	Q9Y4H2	NA	NA	' IRS2	Insulin receptor substrate 2 OS=Homo sapiens OX=9606 GN=IRS2 PE=1 SV=2
Low	A0A075B	' IGLV3-16	Immunoglobulin lambda variable 3-16 OS=Homo sapiens OX=9606 (	0.101	1.013	9.565217	1	1	1	115	12.5	4.65		0.585	A0A075B6	NA	NA	' IGLV3-16	Immunoglobulin lambda variable 3-16 OS=Homo sapiens OX=9606 GN=IGLV3-16 PE=3 SV=2
