# Supplementary material for: ISG15/GRAIL1/CD3 axis influences survival of patients with esophageal adenocarcinoma
Source: JCI Insight. 2024 May 23;9(13):e179315. doi: 10.1172/jci.insight.179315 (PMC11383178; doi:10.1172/jci.insight.179315)

Figure 3A

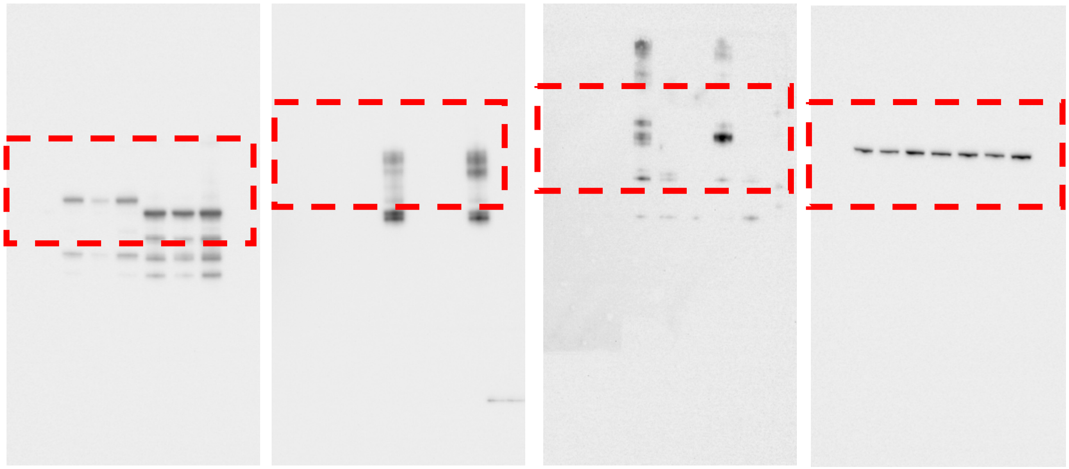

Figure 3B

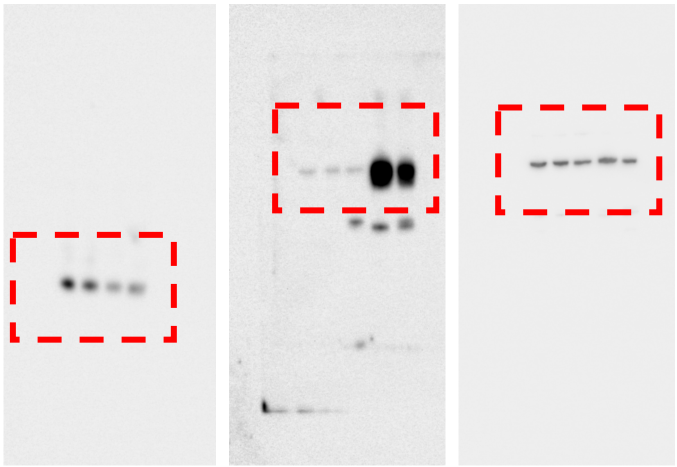

Figure 3C

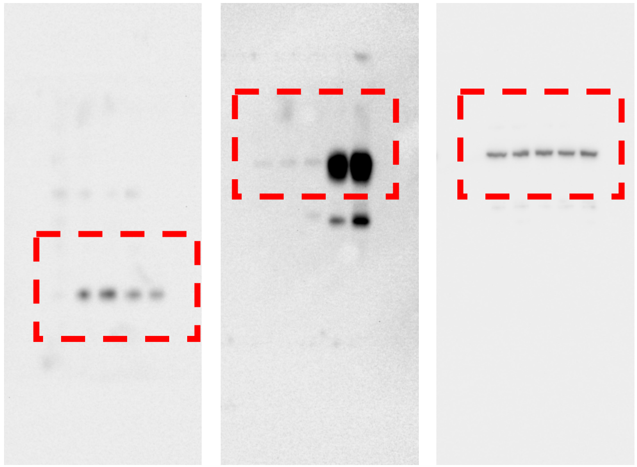

Figure 3D

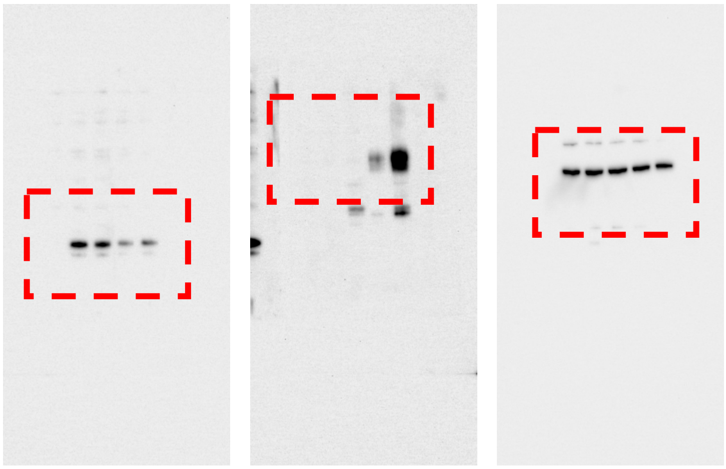

Figure 3E

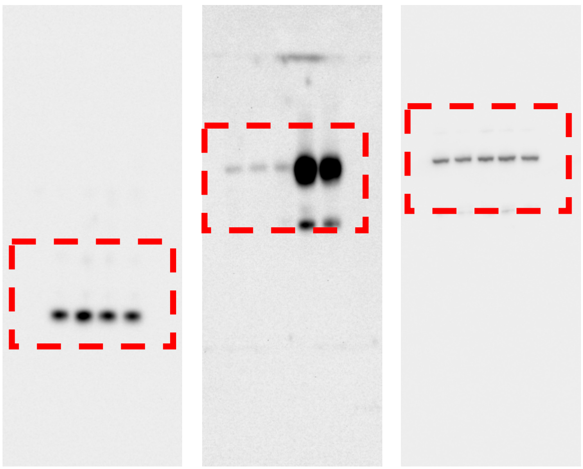

Figure 3G

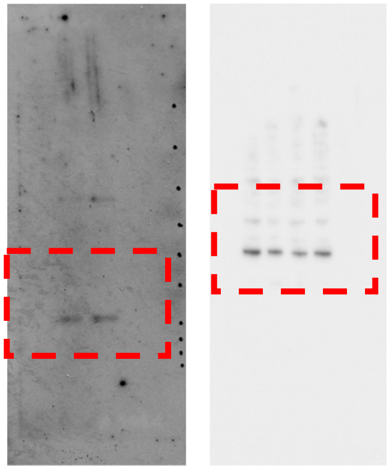

Figure 3H

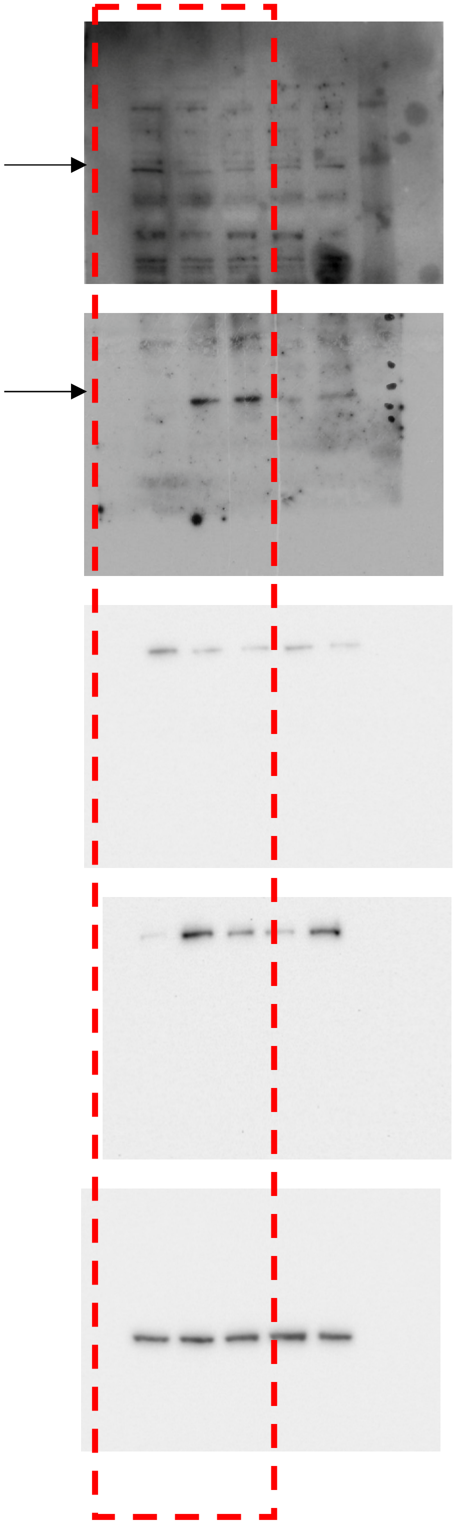

Figure 4A

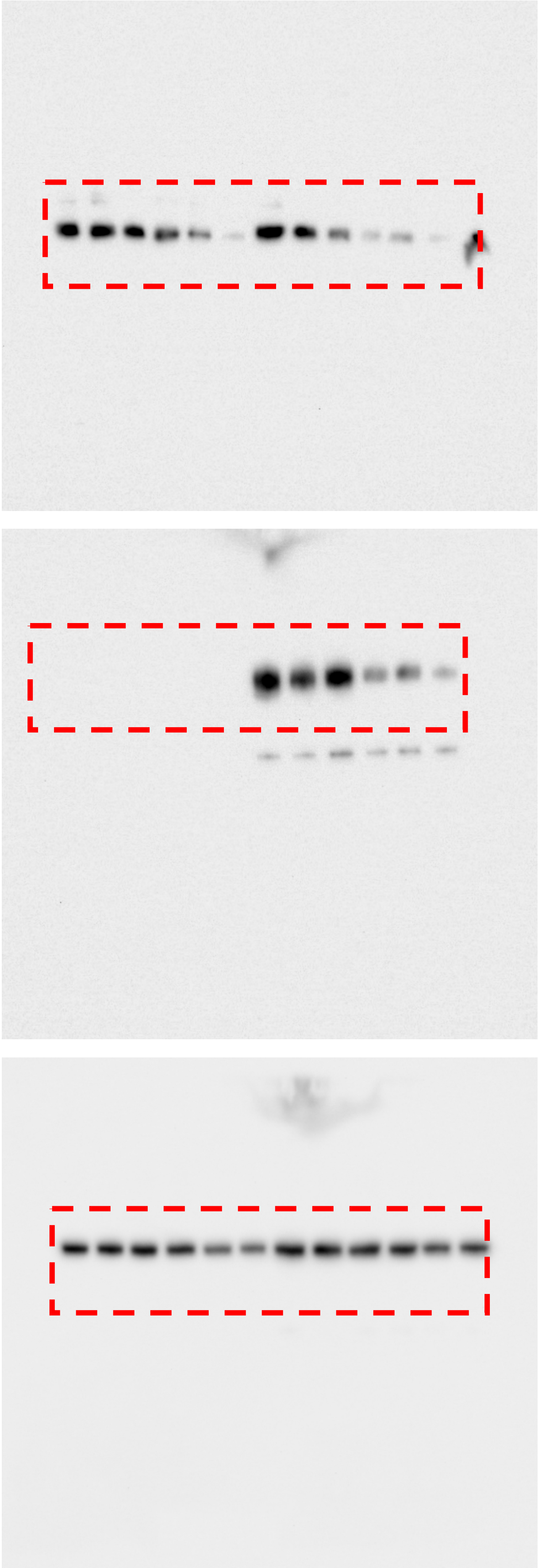

Figure 4C

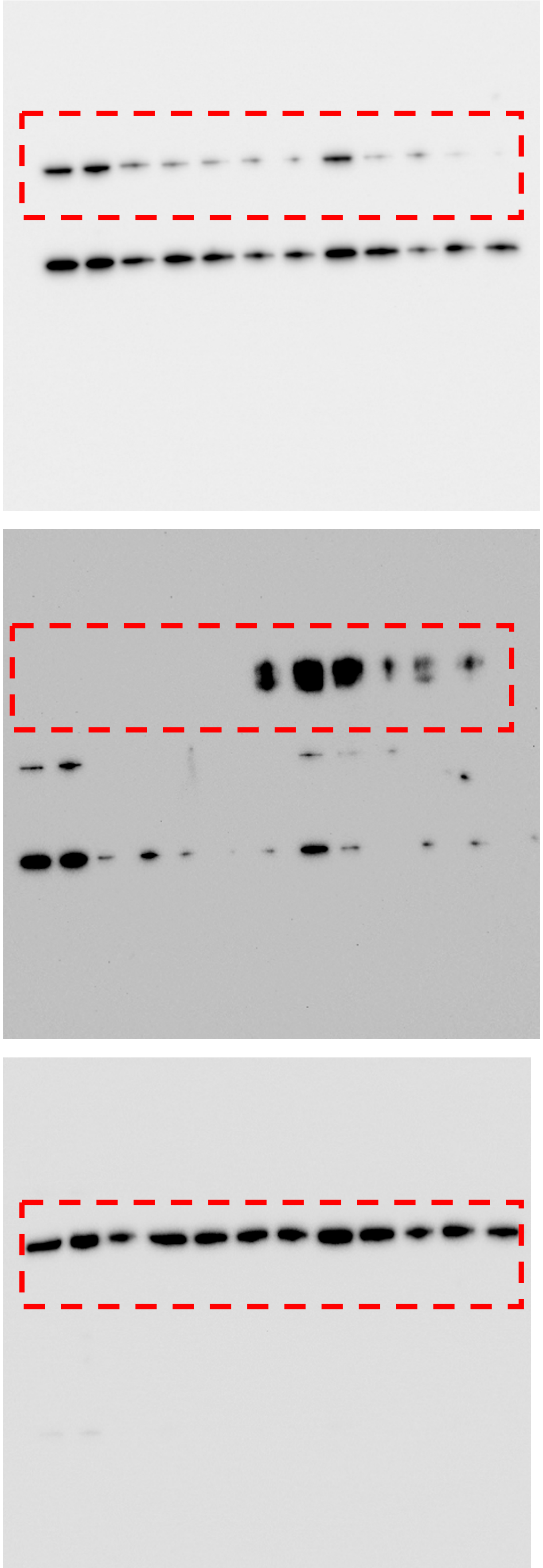

Figure 4E

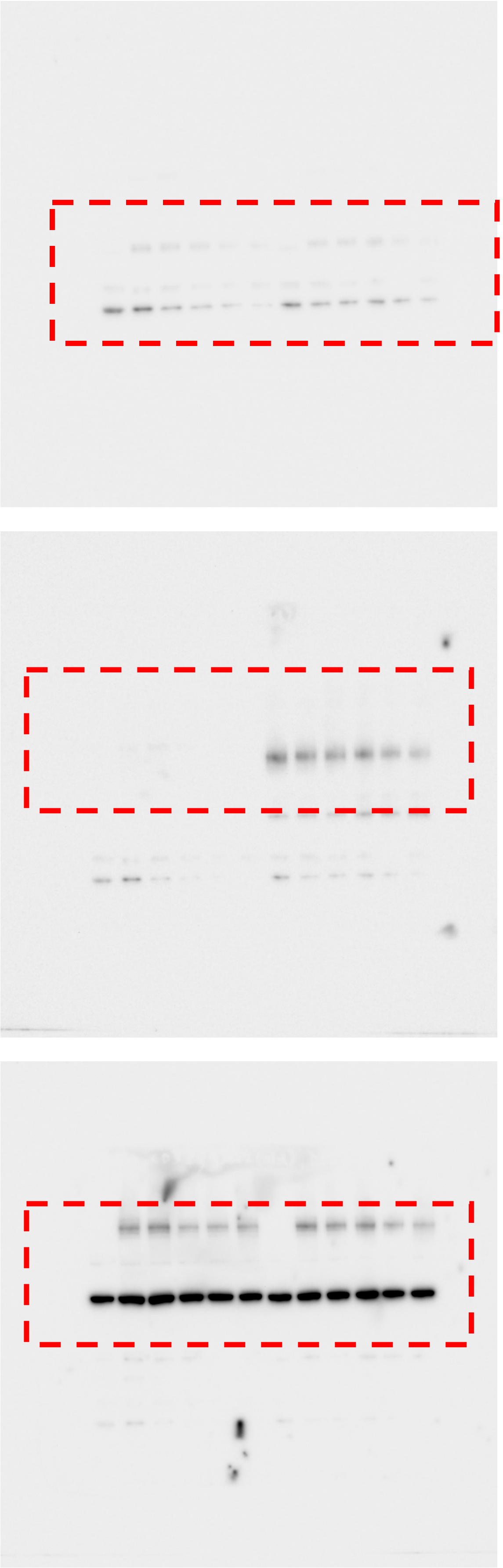

Figure 4G

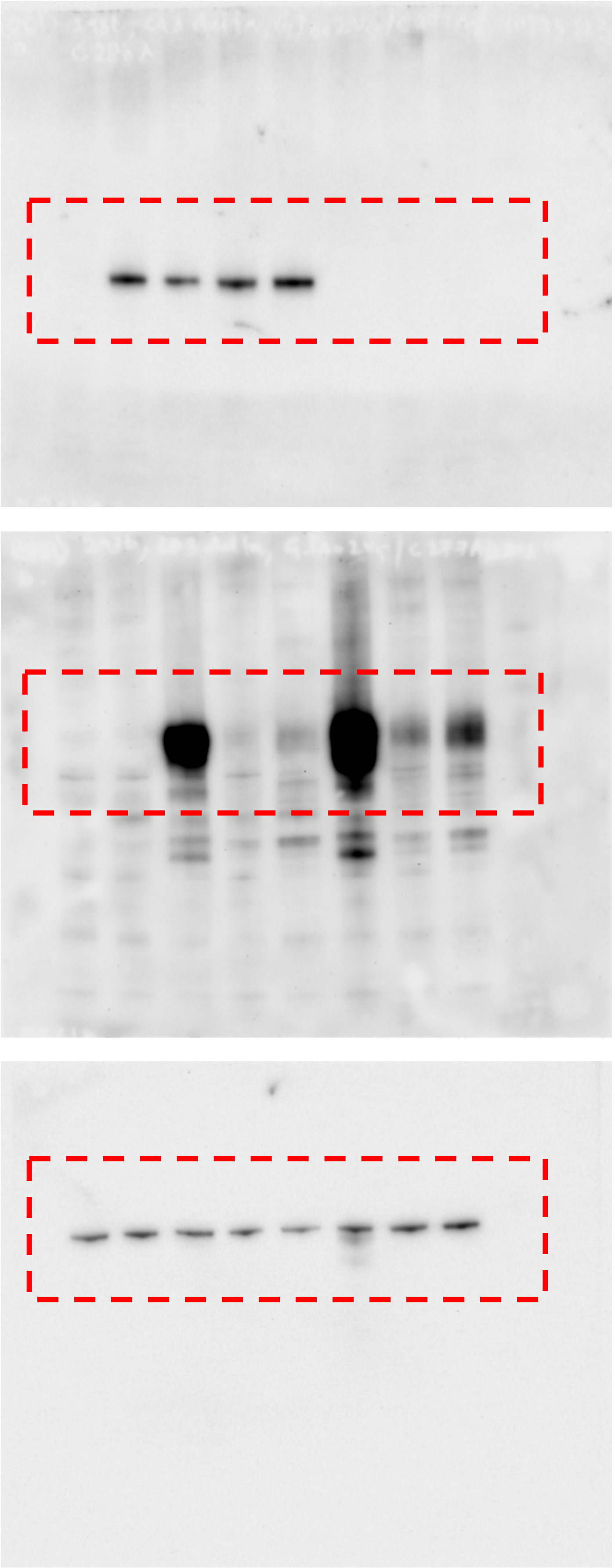

Figure 5A

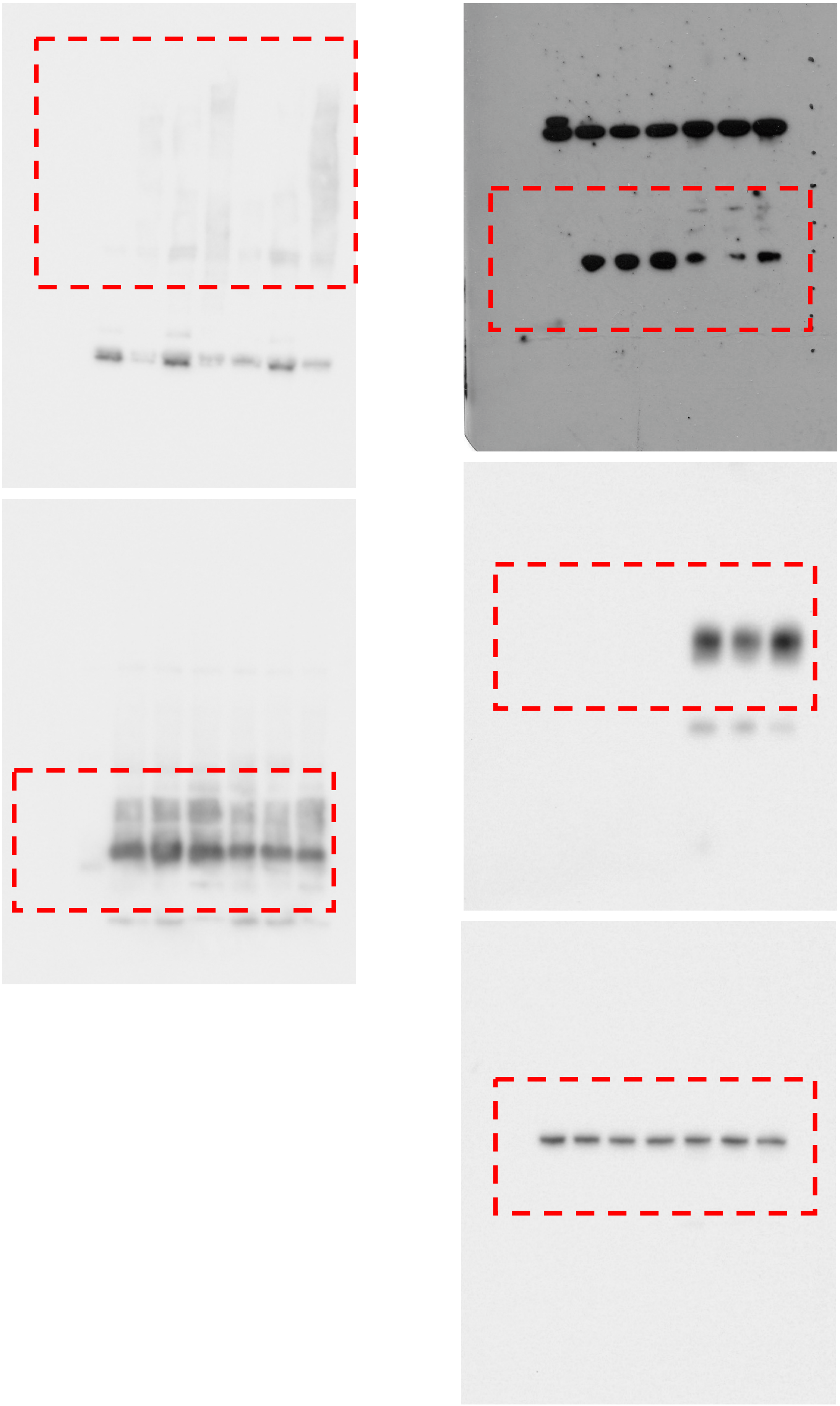

Figure 5B

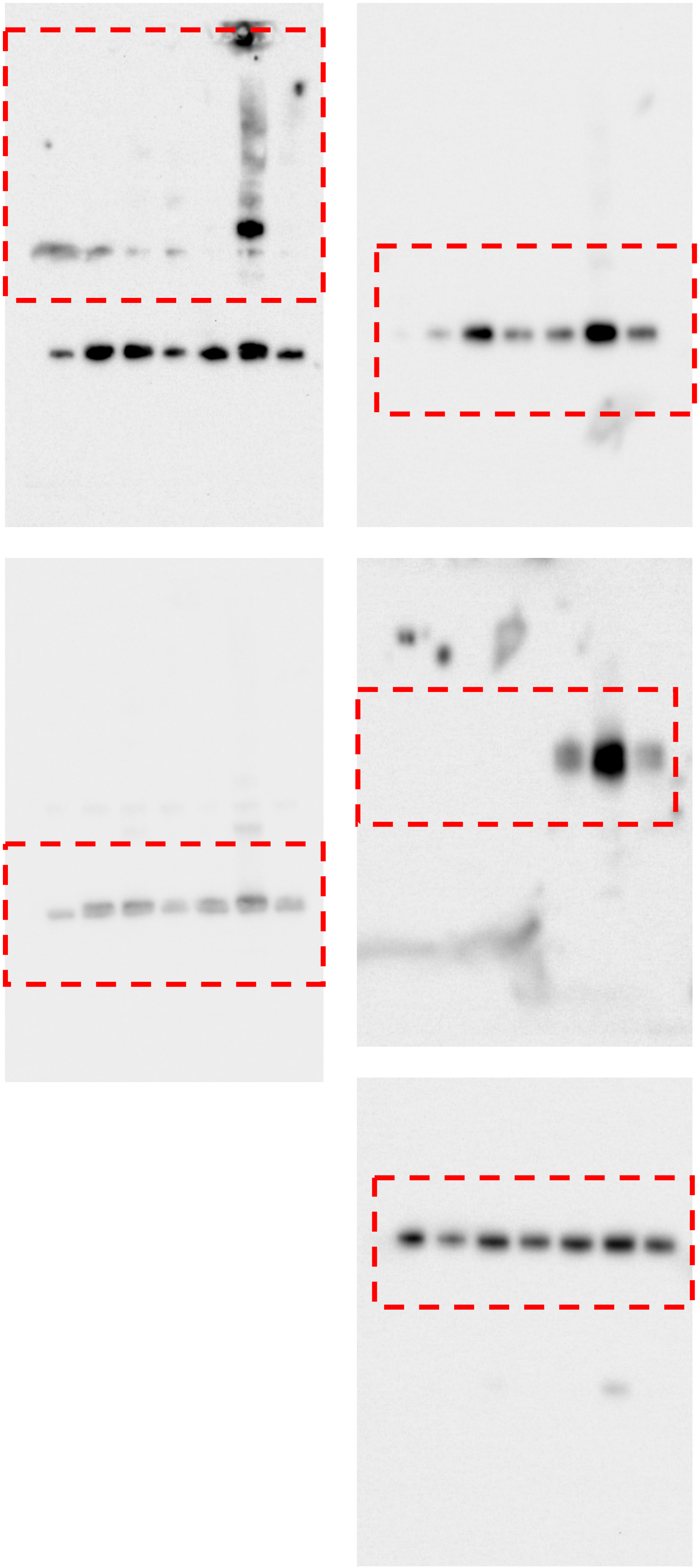

Figure 5C

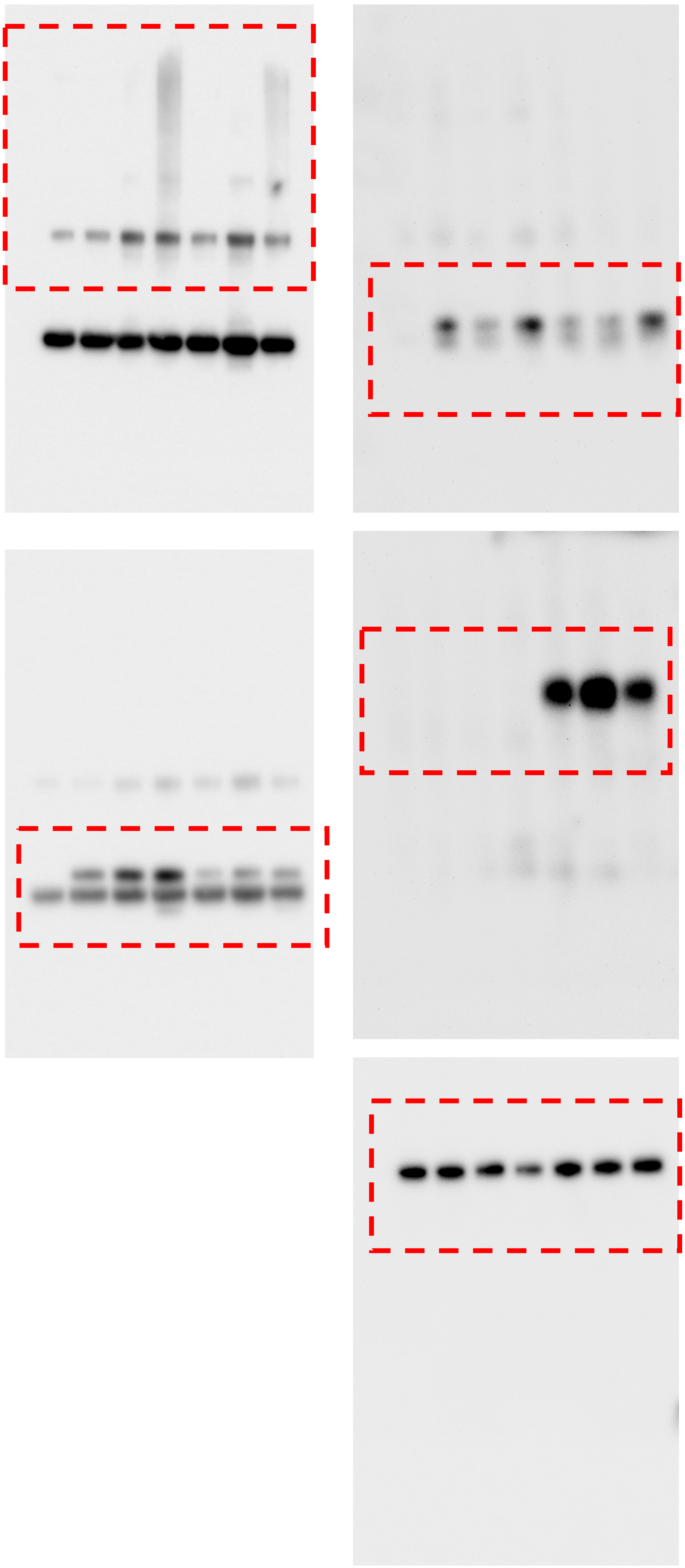

Figure 5D

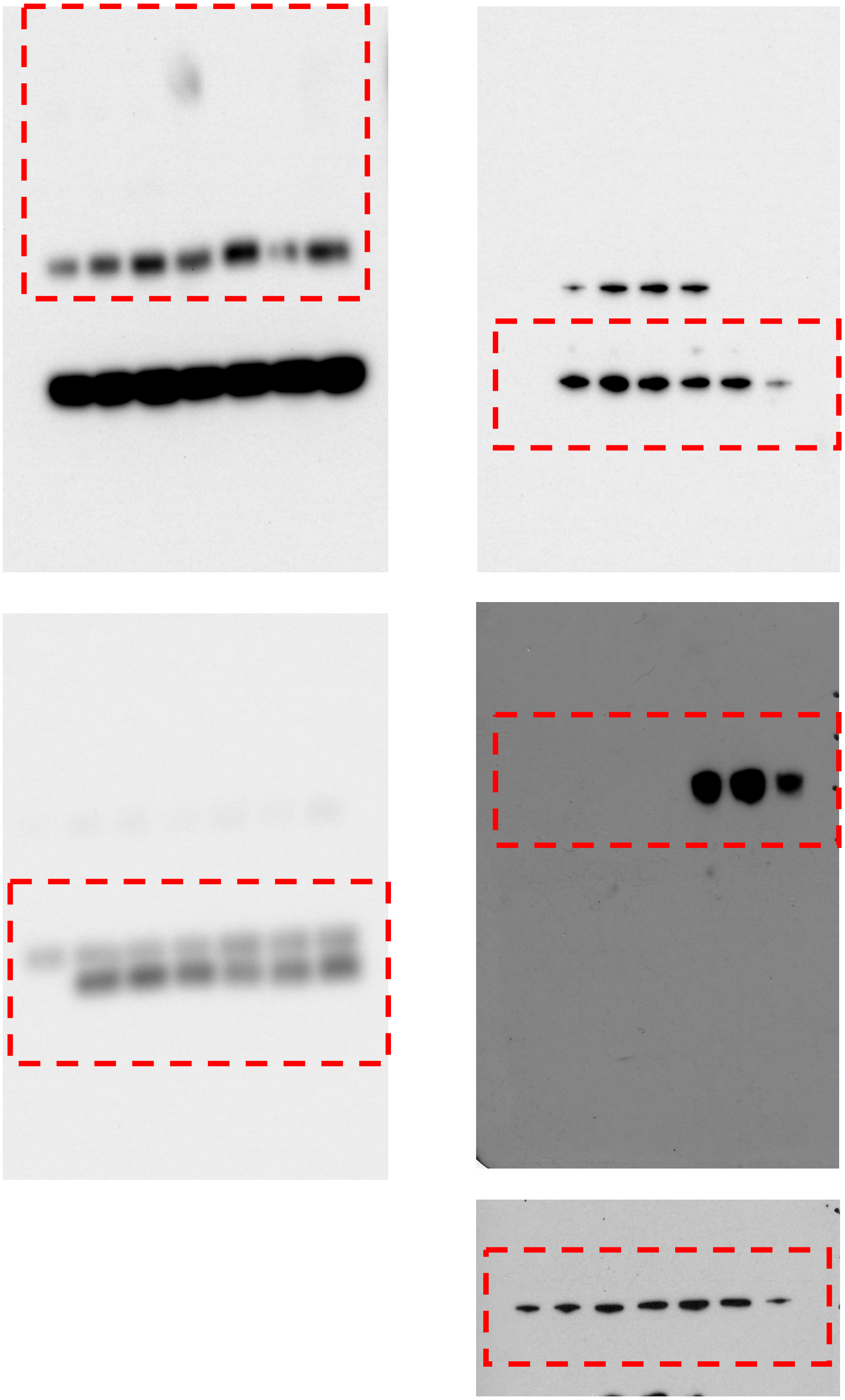

Figure 6A

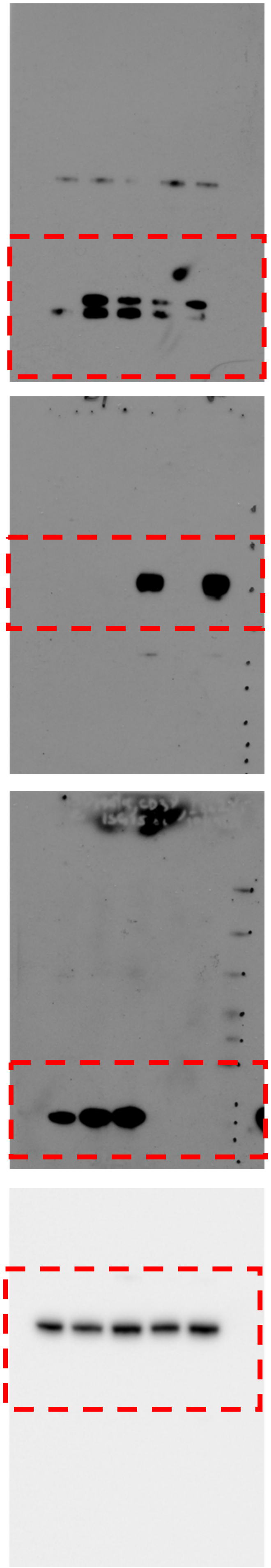

Figure 6B

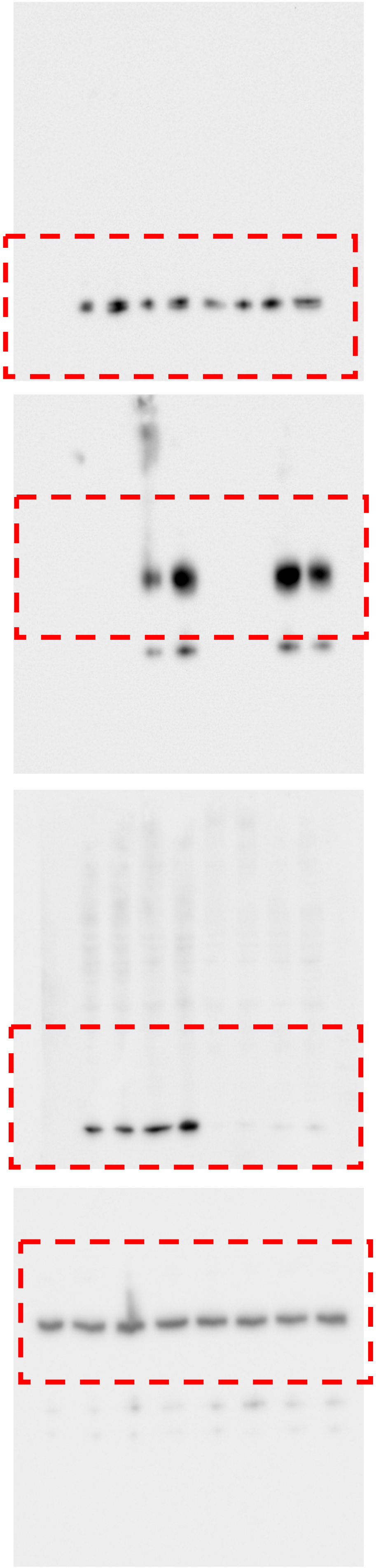

Figure 6C

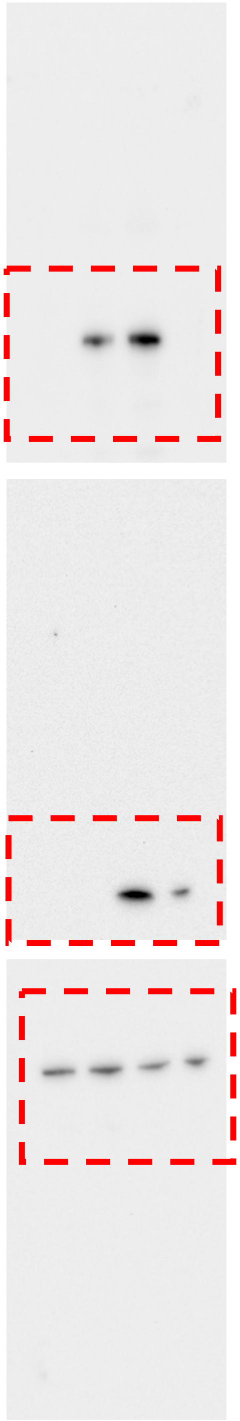

Figure 6D

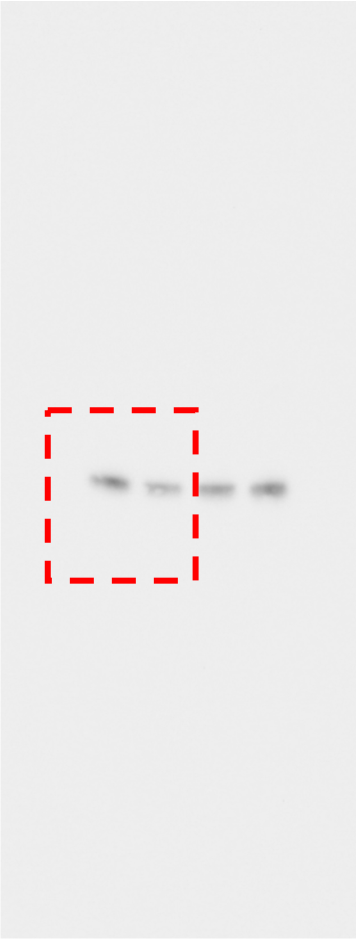

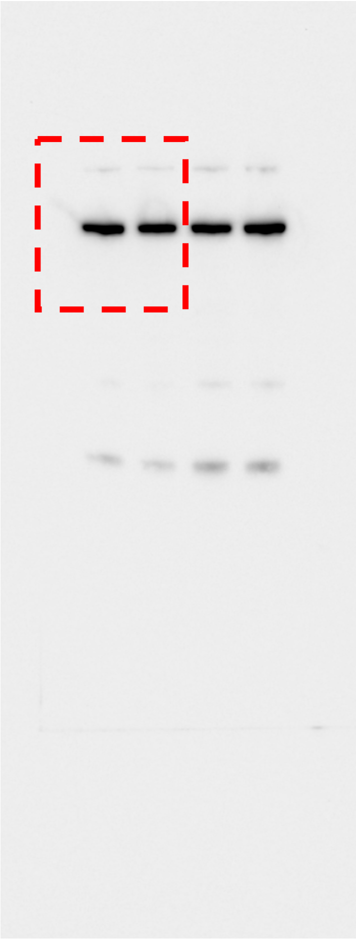

Figure 7A

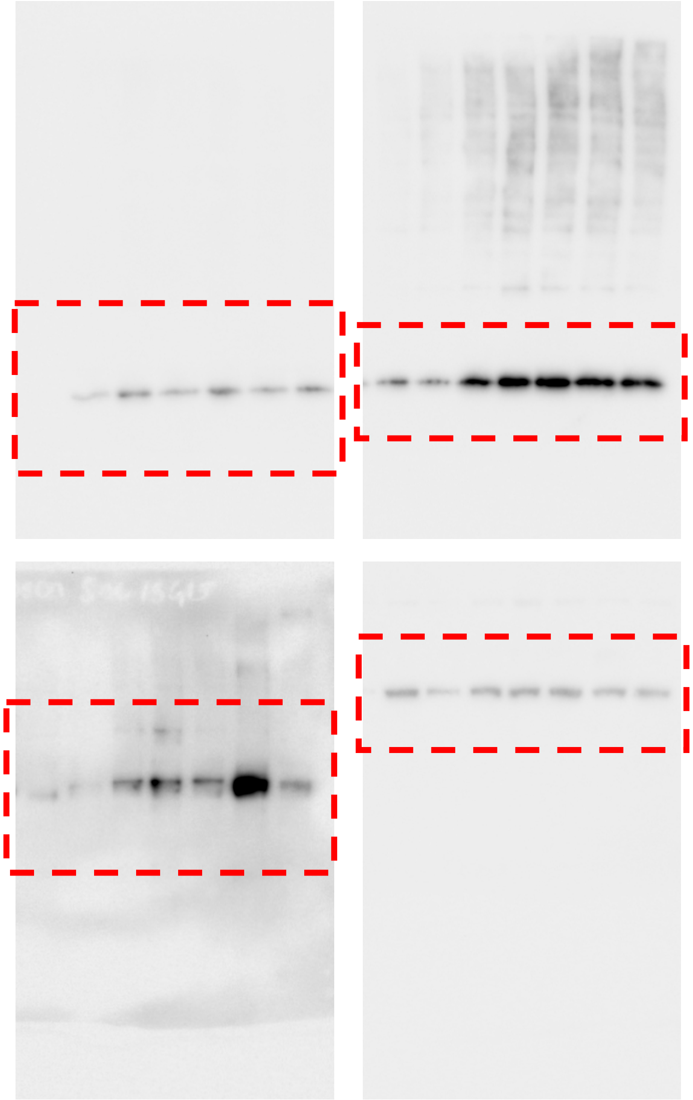

Figure 7B

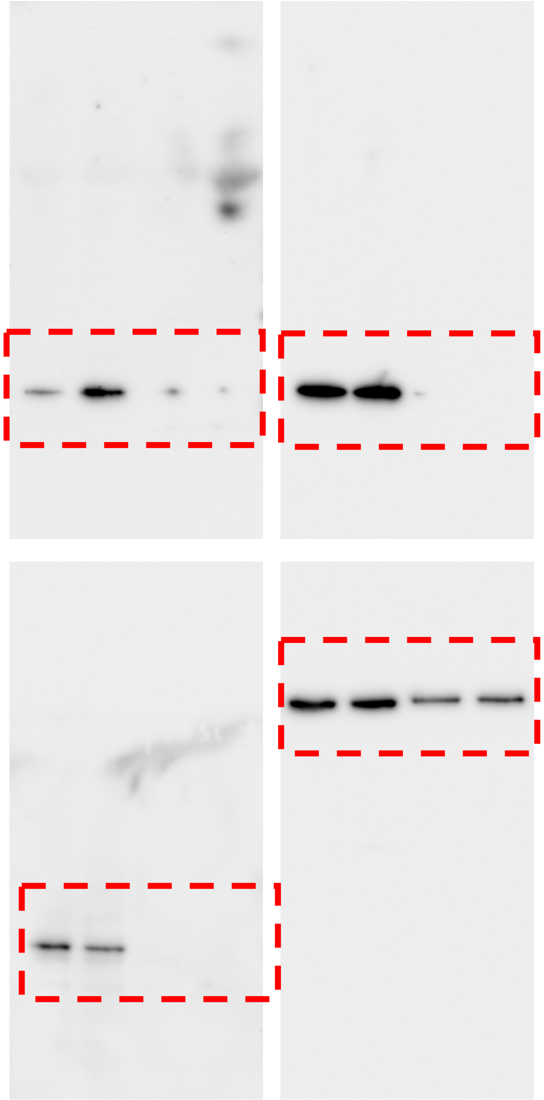

Figure 7C

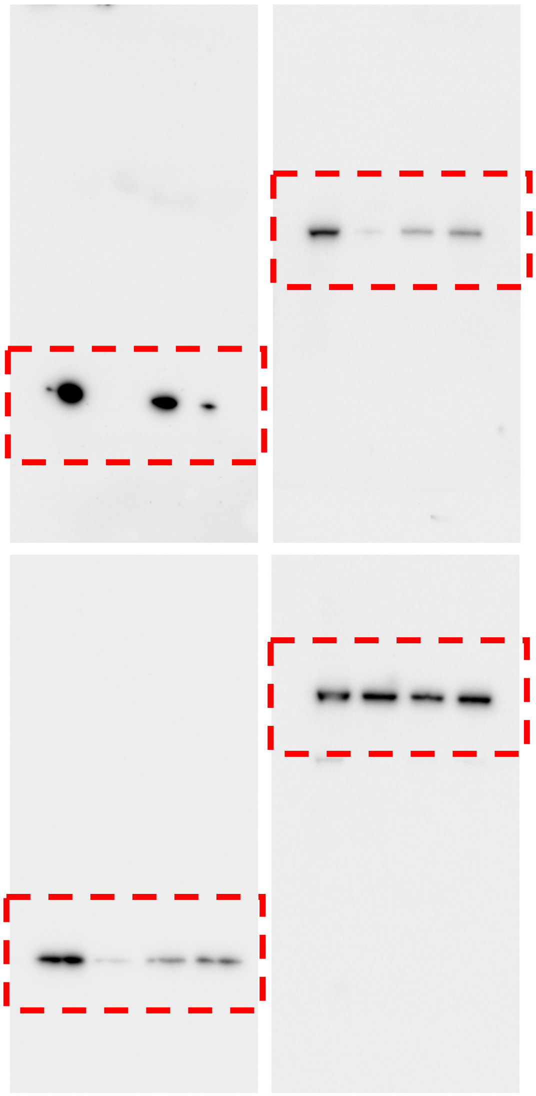

Figure 7D

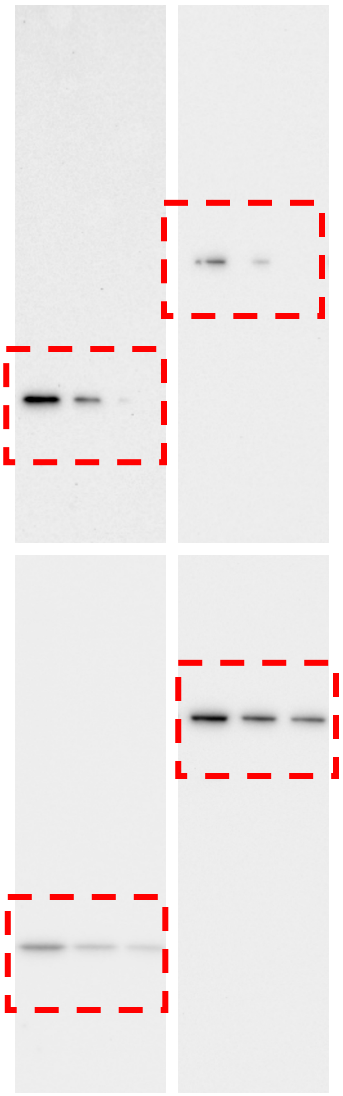

Supplemental Figure 9D

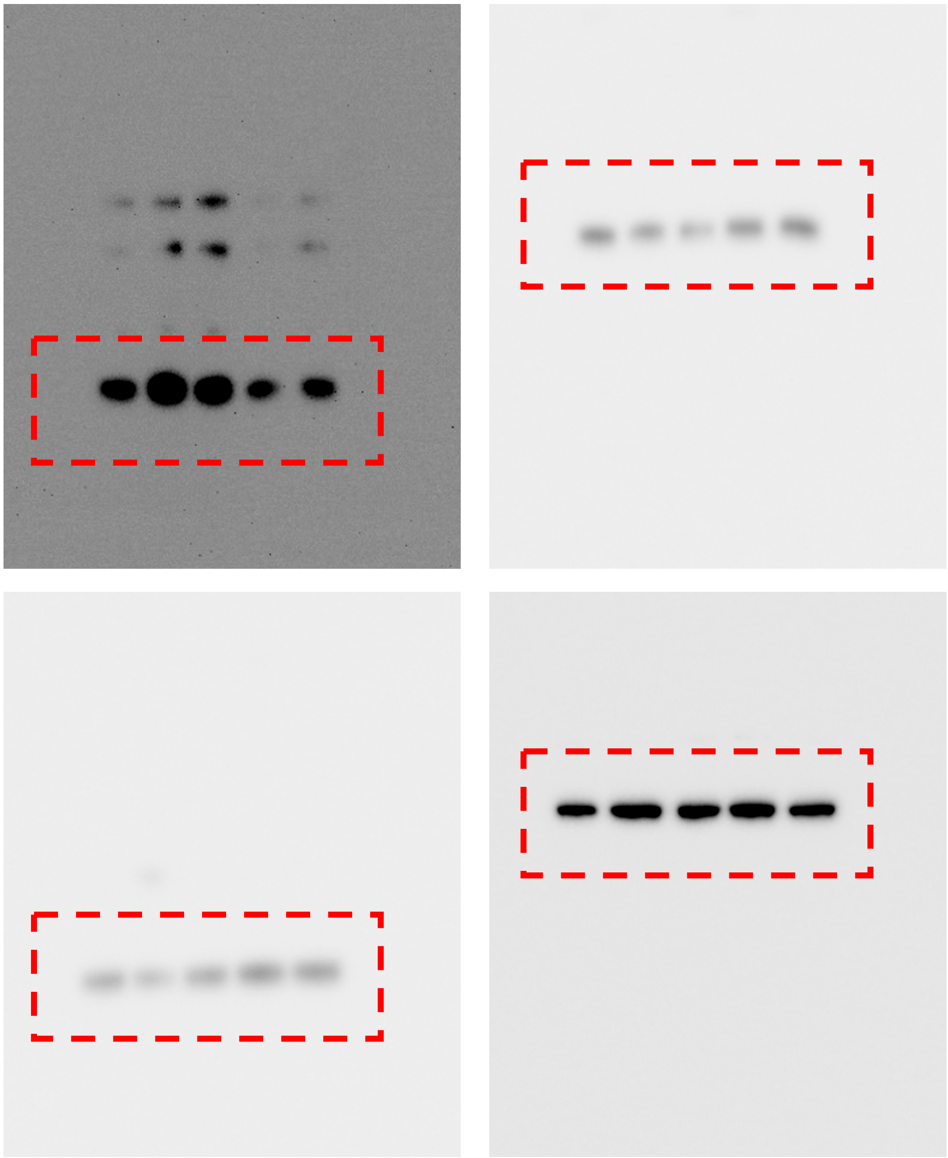

Supplement: Unedited blot and gel images [file jciinsight-9-179315-s077.pdf]
